# Gaps in current methods to detect polymorphic CpGs from Illumina Infinium human methylation microarrays and exploring their potential impact in multi-EWAS analyses

**DOI:** 10.1080/15592294.2023.2281153

**Published:** 2023-11-20

**Authors:** Basharat Bhat, Gregory T Jones

**Affiliations:** Departments of Surgical Sciences, University of Otago, Dunedin, New Zealand

**Keywords:** DNA methylation, Epigenetics, EWAS, CpGs polymorphism, probe polymorphism, genetic polymorphism, EWAS trans-ancestry

## Abstract

DNA methylation (DNAm) epigenome-wide association studies (EWAS) have been performed on diverse ethnicities to discover novel biomarkers associated with various diseases, such as cancers, autoimmune diseases, and neurological disorders. However, genetic polymorphisms can influence DNAm levels resulting in methylation quantitative trait loci (meQTL). These can be either direct effects, by altering the sequence of the methylation (CpG) site itself, or, in the case of array-based measures, indirectly altering the detection probe-binding site interaction. Given that genetic variant frequencies associated with meQTL can differ between population groups, these have the potential to confound EWAS observations, particularly in multi-ethnic populations. In this study, we analysed publicly available DNA methylation profiles (*450K array*), consisting of 1342 individuals from 6 distinct ancestral groups. We investigate two distinct tools (*GapHunter* and *MethylToSNP)* specifically designed to identify CpG sites that may be influenced by genetic variation. Results from this aggregated trans-ancestral epigenome-wide dataset suggest that both tools fail to consistently identify not only rarer (MAF < 0.05) genetic variant effects but also more than half of sites predicted to be associated with variants with much higher allele frequencies (MAF >0.2). In addition, there is a relatively low concordance in the detection of polymorphic CpGs between *GapHunter* and *MethylToSNP*. Screening of CpG site associations from EWAS using either of these tools is unlikely to be a robust or comprehensive means of identifying all genetic variant confounding effects.

## Introduction

Epigenetic studies have increasingly been used to investigate a wide range of biological processes, from basic cell cycle regulation to the multidisciplinary investigations of complex societal exposures, such as psychological stress [1]. The most commonly investigated epigenetic mechanism being DNAm forming 5-methylcytosine (5-mC), due to its alteration by environmental exposures, relative stability, and ability to alter gene expression. As such, changes in specific 5-mC DNAm (CpG) sites have been proposed as useful biomarkers to study environmental influences on biological processes and pathobiology [1].

The Illumina Human Methylation EPIC and 450K arrays have provided the most accessible platform to study genome-wide DNA methylation in humans [2,3]. These arrays cover approximately 99% of the genes in the RefSeq database and are considered to provide robust and accurate assessment of methylation levels in genic and intergenic regions. It is important to note that the assessment of DNAm using these arrays can be influenced by genetic variation [4]. Genetic variation can occur at or near CpG sites, which can either directly ablate the methylation site or influence the binding efficiency or specificity of the probes used in these assays. This can result in misleading DNAm values being reported. Although lists of CpG sites that are potentially prone to these problems are publicly available, these are problematic. For example, they could be used to exclude potentially polymorphic sites, but factors such as population variable allele frequencies, and, in the case of probe-binding region SNPs, variable effects make this difficult and run the risk of over excluding potentially epigenetically relevant sites. It is more preferable to directly screen DNAm results for patterns suggestive of genetic effects, especially in populations in which genetic variation is not as well mapped as in Europeans.

**Figure 1.**
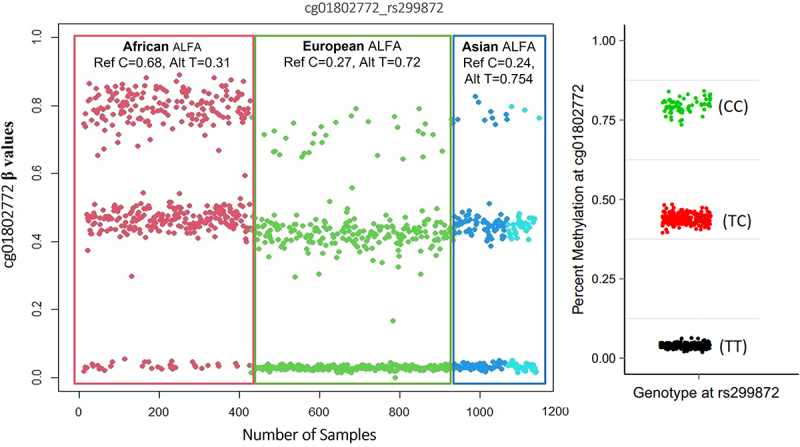
A ß-value plot showing effects of a genetic (SNP) variant on the interrogated CpG site. this site was characterized by having three clusters (upper, intermediate, and lower). The ‘TT’ SNP genotype is associated with significantly lower/no methylation. The ‘CC’ genotype is associated with full methylation. Individuals who are heterozygous for the SNP display intermediate methylation levels. The right-hand distribution plot shows β-values with genotype information as reported by Andrews, Shan V., et al. 2016^5^.

Here, we report our findings regarding the detection of genetic polymorphic effects on DNAm levels assessed using Illumina microarrays. Currently, the most widely used tools for this purpose, as reported in the literature, are *GapHunter* and *MethylToSNP* [5,6]. These are based on the premise that a polymorphic CpG site returns three discrete levels of DNAm, (1) full methylation would correspond with both allelic copies of the CpG site being intact, (2) partial methylation would correspond to a heterozygote single nucleotide polymorphism (SNP) at either the cytosine or guanine nucleotide of the CpG site or (3) the effective absence of methylation due to being homozygotic for a CpG altering SNP. In these cases, β-values will tend to cluster into three discrete levels, with gaps in between, when the methylation levels (β-values) from different individuals are plotted together (Figure 1). The effects of SNPs located in the probe-binding regions are more subtle and need to be considered separately to that of CpG site SNPs. Here, we evaluate the efficiency of existing methods for the identification of apparent polymorphic effects on CpG site β-values generated using Illumina microarrays in ethnically diverse populations.

## Methods

### Methylation profiling

**Figure 2.**
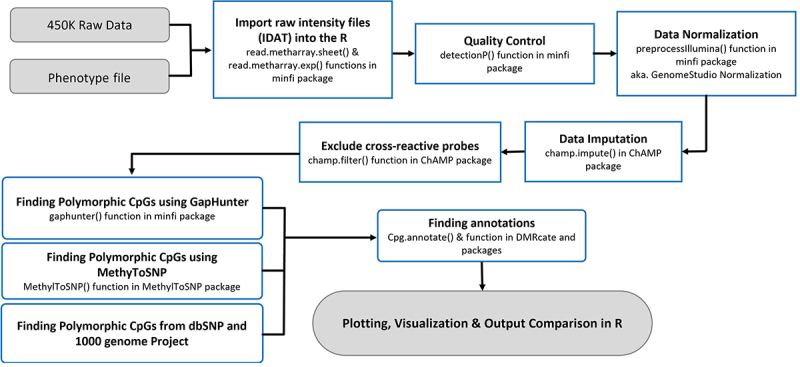
Flowchart of all the steps performed in data analysis.

In this study, we utilized publicly available Infinium® HumanMethylation450 BeadChip datasets from the NCBI GEO database (Table 1). The NCBI datasets were selected to ensure representation from a wide range of ancestral populations. These datasets encompassed samples from six distinct population groups, both male and female individuals, and included a range of age groups. Samples consist of white blood cell-derived DNA from whole or cord blood (Supplementary File 1). All the steps and analyses performed are summarized in Figure 2. Briefly, the raw intensity files (IDAT files) were imported to the R program using the minfi package [7]. Samples with an assigned detection p-value <0.05 were used for the analysis. Intensity files were normalized by the GenomeStudio method using Illumina internal normalization probes with background corrections. The β-value matrix was then imputed to correct for any missing values using the *champ.impute()* function in the ChAMP package [8]. The CpG sites with more than 1% missing values across all samples were discarded. The cross-reactive probes, predicted to hybridize multiple genomic regions, were filtered out [8]. After data quality filtering and pre-processing 472,123 CpGs from 1342 individuals were taken forwards for further analysis.

**Table 1.** GEO accession ID and population structure of the 8 Illumina 450k datasets (*n* = 1342 individuals) examined in this study.

S. No	GEO ID	Number of Samples	Sample IDs
1	*GSE210256*	African Americans: *418*	GSM6424736 to GSM6425153
2	*GSE201287*	Han Chinese: *80*	GSM6057811 to GSM6057890
3	*GSE114935*	White: *21* Hispanic: *18* African: *4*	GSM3155928 to GSM3156489
4	*GSE104812*	Chinese: *48*	GSM2808239 to GSM2808286
5	*GSE73412*	Chinese: *69* African: *5*	GSM1893070 to GSM1893143
6	*GSE111629*	Caucasian: *508* Hispanic: *63*	GSM3035401 to GSM3035900
7	*GSE65638*	Chinese: *16*	GSM1602316 to GSM1602331
8	*GSE69633*	Mexican: *92*	GSM1704992 to GSM1705083

### Detection of potentially polymorphic CpGs

We utilized the *GapHunter* and *MethylToSNP* packages to identify polymorphic CpGs. Both tools were implemented using the Bioconductor (https://www.bioconductor.org/) bioinformatics software platform. These two methods are based on different polymorphic CpGs detection approaches. In *GapHunter,* the methylation levels of candidate CpG sites are sorted and grouped into clusters. In *MethylToSNP,* the methylation levels of each site are typically grouped into three clusters with defined thresholds. Probe annotation information was obtained from the BioConductor package Illumina Human Methylation 450 kanno. ilmn 12.hg19 version 0.6.1. We interrogated the 2020-Jan release of the *1000 genome project* and *NCBI dbSNP* Build 155 (release date: 16 June 2021) to generate a list of potentially relevant genetic polymorphisms [9,10]. A CpG site is deemed potentially polymorphic if an SNP was documented with either C or G residue on either strand.

A separate list of SNPs located within probe-binding regions was also generated. The potentially polymorphic CpG sites identified by *GapHunter* and *MethylToSNP* were compared against known polymorphic CpGs.

## Results & discussion

In this study, we utilized *GapHunter* and *MethyToSNP* to investigate their ability to identify potentially polymorphic CpGs across a multi-ethnic genome-wide dataset.

**Figure 3.**
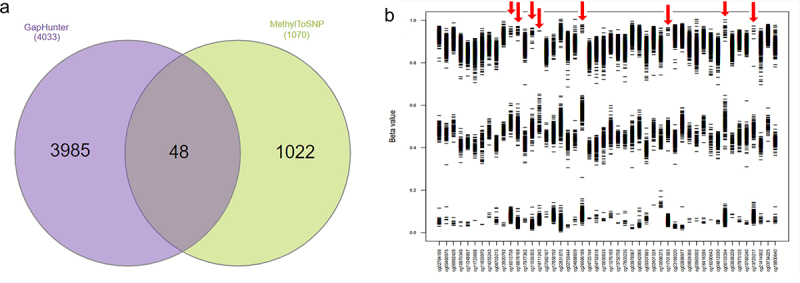
(a) venn diagram represents the common and specific predicted polymorphic CpGs identified by GapHunter and MethylToSNP tools. (b) Β-value distribution of the 48 polymorphic CpGs identified by both the GapHunter and MethylToSNP tools.

The raw data were pre-processed using the pipeline described in Figure 2. *GapHunter* and *MethyToSNP* were applied on the normalized and prefiltered datasets. A total of *4033* and *1070* CpG sites were identified as being potentially polymorphic using *GapHunter* and *MethylToSNP,* respectively (Supplementary Files 2, 3). Only 48 potentially polymorphic CpGs were consistently detected by both tools (Figure 3a). Subsequently, we plotted the β-value distributions for these 48 sites and observed that they each formed three distinct cluster patterns (as described above, Figure 1). While over 80% (40/48) of sites had most of their values in the upper cluster, likely suggesting that the lower two clusters were associated with SNP minor alleles, which resulted in CpG ablation, a minority (8 sites) had an inverse effect. In these eight sites (shown by red arrows in Figure 3b) a majority of samples were associated with the lower (near zero) β-value cluster, with the upper two clusters therefore likely being explained by an SNP creating a CpG site in those individuals. Similar effects for these eight sites were observed in the Simons Genome Diversity Project (SGDP) [11].

Interestingly, none of these 48 sites displayed a hypomethylated pattern (mean upper cluster β-values in the range 0–0.5) which appears inconsistent with the prevalence of hypomethyated sites (typically approximately 40%) at the genome-wide level. To investigate this further, we conducted a thorough examination of the β-value distributions for all 4033 potentially polymorphic CpGs identified by GapHunter. For this analysis, potential meQTLs were stratified based on the known allele frequencies of the predicted associated SNP. As with the previous analysis, we were unable to observe patterns suggestive of polymorphic hypomethylated sites (Supplementary Figure S1). As a sensitivity analysis, we also adjusted for study participant age and sex, and where able to confirm that the observed potential meQTL β-value distributions where no substantivity is confounded by these factors (Supplementary Figure S2).

We suggest that the absence of detection of potentially polymorphic hypomethylated sites may be due to the methodology used by these tools. Firstly, MethylToSNP detects clusters based on a set of predetermined thresholds for methylation levels [6]. As such, this essentially excludes the possibility of identifying hypomethylated polymorphic CpGs. Secondly, GapHunter aims to identify discrete clusters of β-values by detecting ‘gaps’ between them. These ‘gaps’ will be compressed together, and therefore more difficult to discretely resolve, in CpG sites in which the mean β-values (of an intact CpG site) are less than 0.5.

**Figure 4.**
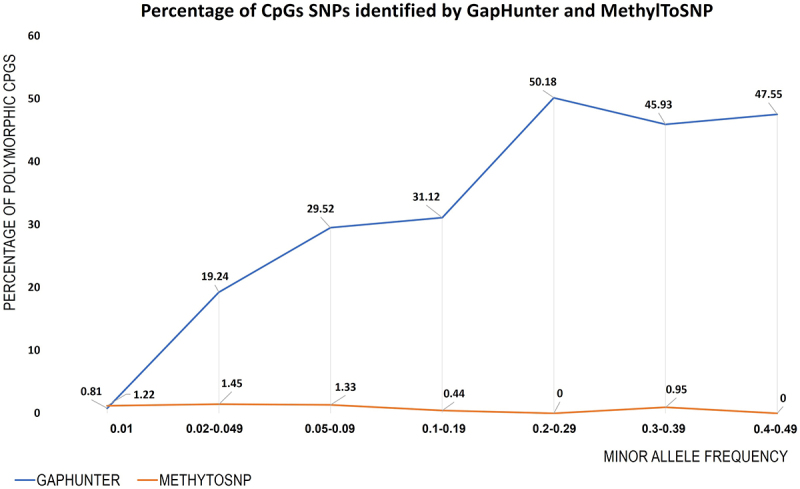
Percentage of polymorphic CpGs identified from *GapHunter* and *MethylTosnp* compared to associated SNP allele frequencies. CpG sites were binned into seven groups based on the MAF of the associated SNP. The percentage of potentially polymorphic CpGs identified by the respective tools are provided for each MAF sub-group.

To estimate the likely efficiency of both tools to identify polymorphic CpGs associated with SNPs of differing MAFs, we examined *GapHunter* and *MethylToSNP* predictions and compared them to the reported allele frequency of the associated SNP (Figure 4). Across all stratified MAF groups, we observed a very low concordance between the predicted polymorphic CpGs identified by *MethylToSNP* (Figure 4). In contrast, *GapHunter* showed a stronger relationship between the rate of polymorphic site detection and MAF. At the CpG sites containing SNPs with MAF > 0.20 *GapHunter* identified 40% to 50% of the sites as being polymorphic (Figure 4). This rate appeared to fall in a linear fashion when the MAF was below 0.2. With rare SNPs (MAF < 0.02) *MethylToSNP* and *GapHunter* both identified only 1% of the predicted polymorphic sites.

We then examined CpG sites predicted to be associated with SNPs but not flagged as meQTL by either of the tools (Supplementary Figure S3). Of concern, we observed numerous examples of unflagged CpG sites that had 3-cluster distributions, whose sample distributions between each cluster were generally consistent with Hardy – Weinberg equilibrium predictions, further supporting the suggestion of a polymorphic effect. Supplementary Figure S3 illustrates numerous examples of polymorphic CpGs showing three distinct clusters of methylation levels but which were not flagged as potential meQTLs by GapHunter. These findings suggest that GapHunter may not be sensitive enough to detect all polymorphic CpG sites, especially those with lower MAF. In particular, with rare SNPs (MAF < 0.02), both tools only identified a very small percentage of the predicted potentially polymorphic sites, which raises concerns about their accuracy and reliability in detecting DNA methylation effects due to such rare polymorphisms.

**Figure 5.**
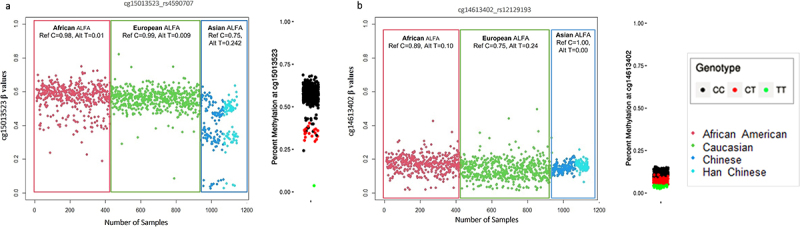
Represents the varied impacts of probe-site polymorphisms on β-value levels in different ethnicities. the left-hand plot for each CpG site shows individuals examined in this study (grouped by three major ethnicities) with associated SNP ALFA MAF from *dbSNP*. (a) a CpG site with a probe region SNP that appears to produce discrete β-values clusters, (b) a separate example where the SNP appears to alter β-value distributions patterns without producing noticeable cluster gaps. The right-hand distribution plot for each site shows β-values with genotype information as reported by Andrews, Shan V., et al. 2016^5^.

In array-based DNAm studies, it is important to consider not only CpGs polymorphism but also the potential influence of probe-specific polymorphic sites. Probe site polymorphisms refer to genetic variations occurring within specific probe-binding regions. These variations can include SNPs or other genomic variations that could impact upon binding efficiency or stability of the probe–DNA interaction. The potential for such effects to alter the interpretation of differential DNAm analysis in EWAS is of substantial concern because, in contrast to CpG site SNPs, these variants may result in smaller percentage changes to the reported β-values. As shown in Figure 5, in which two separate examples of probe SNPs (rs4590707 and rs12129193) are shown that appear to influence the CpG site reported β-values but do not consistently result in detectable ‘gaps’ between signal clusters [5]. Without access to genotype information, distinguishing these signals from true associations represents a considerable challenge.

To mitigate against such probe SNP effects, Illumina has provided a comprehensive list of underlying SNPs, including minor allele frequencies and their probe location relative to the CpGs [8,12]. Many researchers choose to exclude all polymorphic sites from their analysis to avoid potential confounding effects on their results. However, this approach will substantially reduce the number of sites being analysed. To avoid this loss of detection power, researchers may choose to selectively exclude polymorphic sites that are likely to have a strong impact on the results, while retaining those that are less likely to be confounding. For example, one approach is to exclude CpGs associated with SNPs with a minor allele frequency above a certain threshold. While this may retain sites that are less likely to be impacted by a polymorphic effect, it is difficult to be sure that the strategy will be consistent across different ancestry sub-groups. Another approach is to use statistical methods that are robust to measurement error or that explicitly model the effect of genetic variation on DNAm. However, such methods should be both sensitive and specific to ensure that only true polymorphic sites are identified and to avoid false positives or false negatives.

In order to evaluate GapHunter’s performance for the identification of probe SNPs (as distinct from CpG site SNPs), we performed a comparative analysis by juxtaposing the results obtained from GapHunter with a list of potentially polymorphic probe sites reported by Illumina. We specifically focus on the identification of common SNPs, which are more likely to have a confounding effect in EWAS. To achieve this, we selected a subset from Illumina’s list consisting of 25,877 potential probe site SNPs with a MAF > 0.1. From this subset, GapHunter identified 1305 potential meQTLs, which is only 5% of the predicted SNP variable sites. Interestingly, in this multi-ethnic study, GapHunter failed to detect the likely polymorphic nature of certain sites, even when distinct gaps were present in a particular population (Figure 5a). These findings suggest the need for a thorough evaluation of GapHunter’s effectiveness in accurately capturing all genetically intricate sites.

## Future directions

Based on our observations, there is a clear need to develop more resilient and precise tools to accurately detect genetic variant confounding of DNAm observations. This is proving to be a formidable challenge which, even with refinements, may be beyond the capacity of current tools. Future tools will need to have the ability to accommodate a wide spectrum of allele frequencies and methylation patterns, including hypomethylated sites. In the interim, we would suggest that researchers consider careful integration of their DNAm data with appropriate genotype reference data. This integration can help flag potential associations between known genetic variations and DNA methylation levels, however, this may not address issues of cryptic meQTL in less well genetically characterized populations. The future most likely lies in approaches utilizing machine learning algorithms, to identify DNAm patterns suggestive of genetic variant effects. Such algorithms will need to be carefully trained on genetically diverse datasets to improve their accuracy in detecting polymorphic CpG sites and SNP effects within probe-binding regions.

## Conclusion

There is no doubt that polymorphic CpGs can confound downstream methylation analysis, with the presence of genetic variations leading to spurious associations between DNA methylation levels and the phenotype of interest. In addition, if a polymorphic CpG is associated with a disease phenotype, but its methylation state is also associated with genetic variation at that site, it may be difficult to determine whether the association is directly associated with differential methylation or is acting as an indirect surrogate of a genetic effect [13]. It is important to note that, polymorphic CpGs can vary in frequency across different populations; therefore, studies that aim to compare DNAm levels across ancestrally diverse populations should be particularly careful to investigate the potential influence of polymorphic CpGs on their analysis and interpretation. Moreover, these findings suggest that there may also be limitations in the current methods with regard to identifying hypomethylated polymorphic CpGs and SNP effects within probe-binding regions.

In conclusion, while tools such as GapHunter manage to find a proportion of meQTLs, our analysis suggests that many genetically confounded sites will escape identification. Therefore, a thorough interrogation of EWAS top hits also needs to be performed for the comprehensive identification of potentially genetically confounded CpG sites.

## Supplementary Material

Supplementary Figure 2.jpg

Supplementary_File_3.docx

Supplementary_File_2.docx

Supplementary_File_1.docx

Supplementary Figure 1.jpg

Supplementary Figure 3.jpeg

## Data Availability

Publicly available datasets from the NCBI GEO database were utilized in this study.
