# Supplementary figures and images for: Gaps in current methods to detect polymorphic CpGs from Illumina Infinium human methylation microarrays and exploring their potential impact in multi-EWAS analyses
Source: Epigenetics. 2023 Nov 20;18(1):2281153. doi: 10.1080/15592294.2023.2281153 (PMC10732615; doi:10.1080/15592294.2023.2281153)

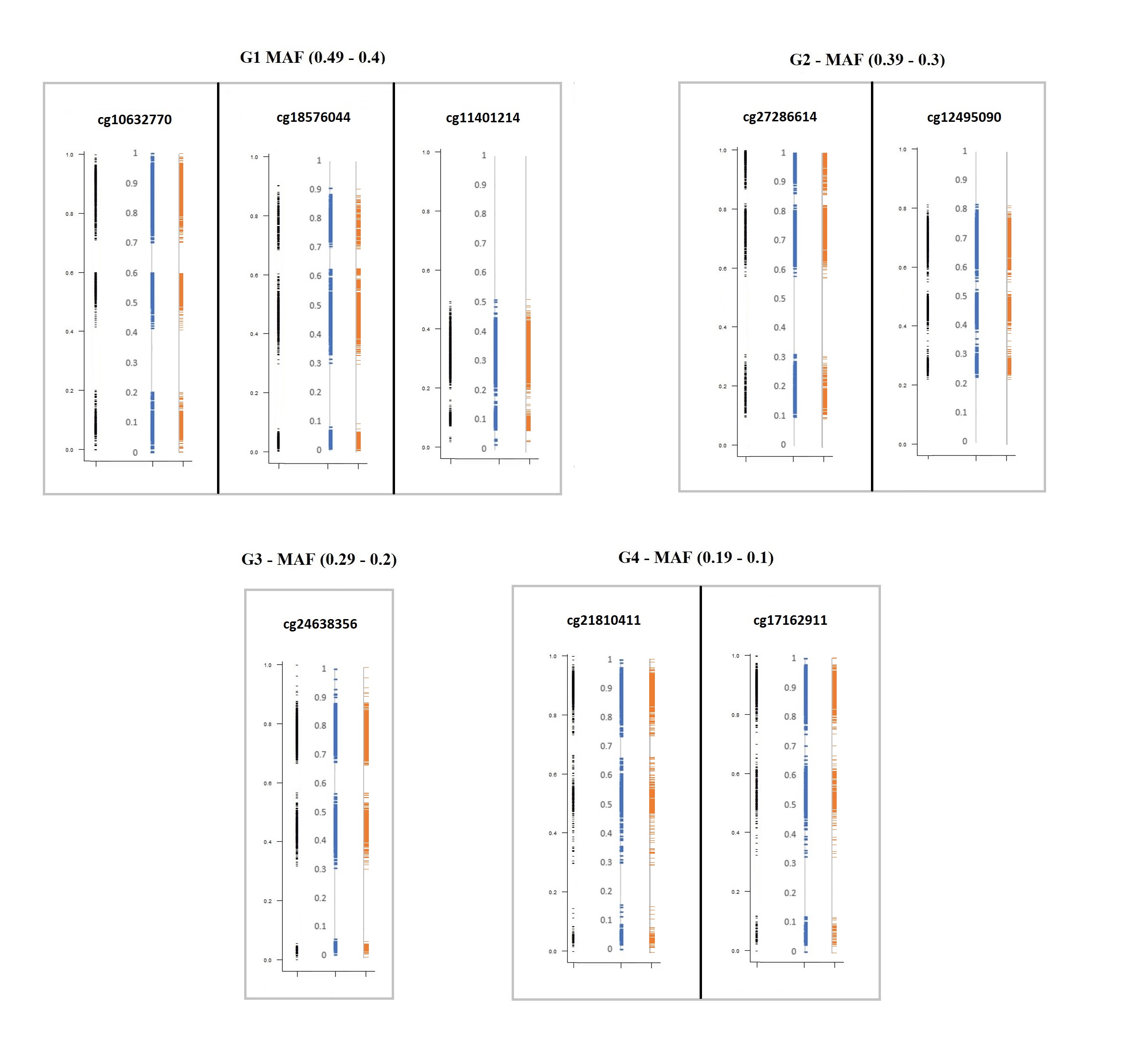

Supplement: Supplementary Figure 2.jpg [file KEPI_A_2281153_SM9092.jpg]

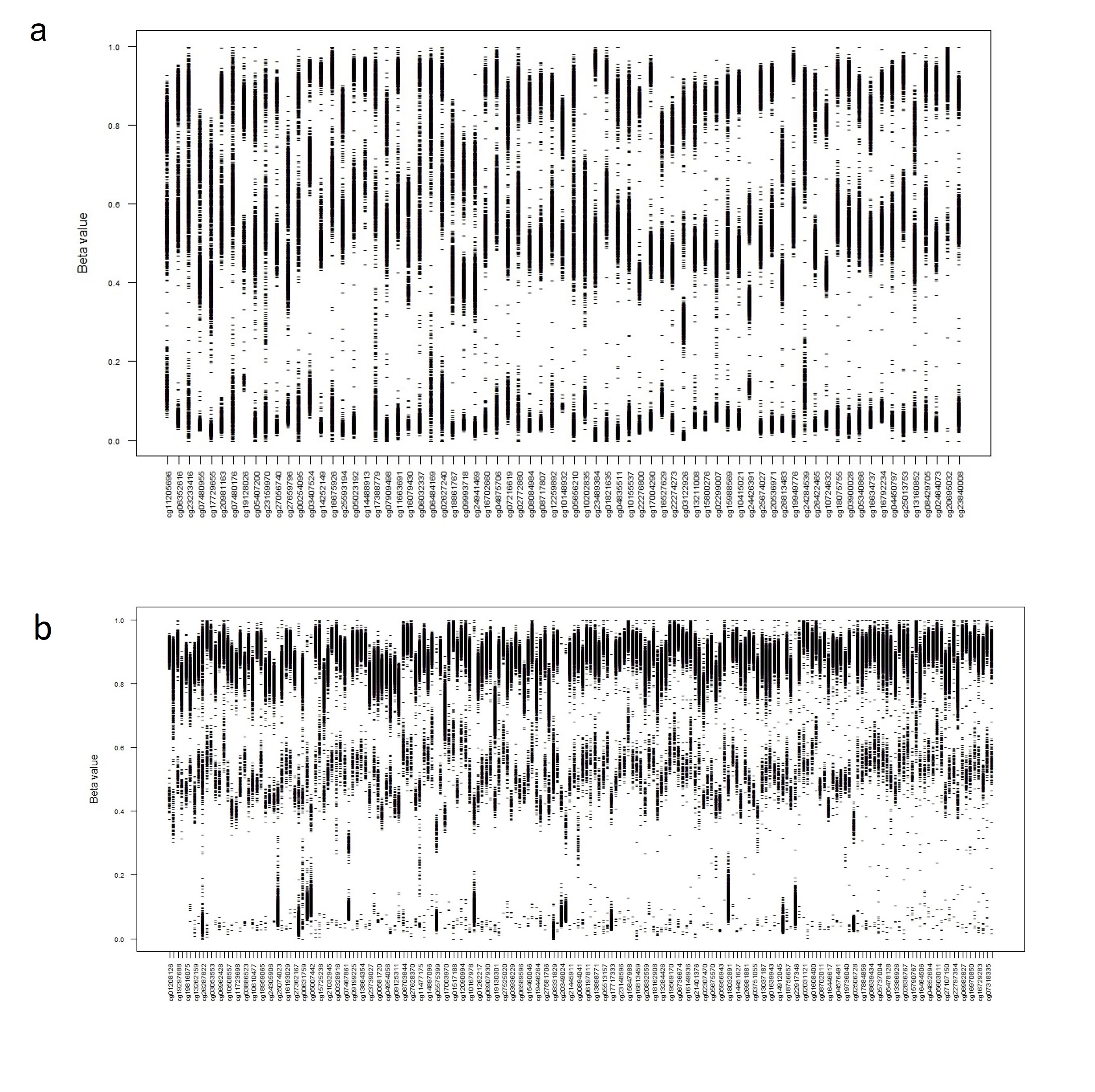

Supplement: Supplementary Figure 1.jpg [file KEPI_A_2281153_SM9087.jpg]

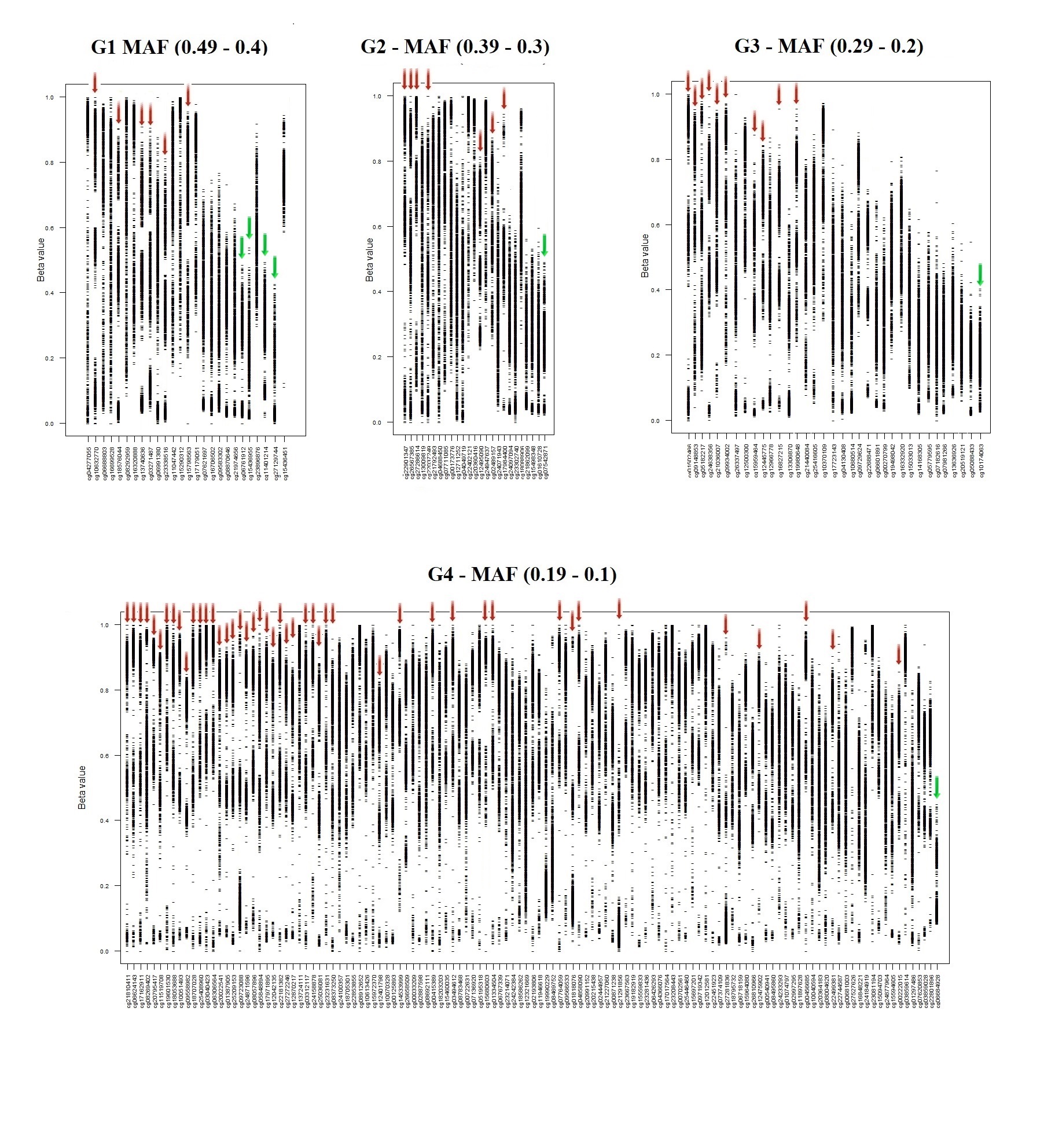

Supplement: Supplementary Figure 3.jpeg [file KEPI_A_2281153_SM9086.jpeg]
