# Supplementary material for: Gaps in current methods to detect polymorphic CpGs from Illumina Infinium human methylation microarrays and exploring their potential impact in multi-EWAS analyses
Source: Epigenetics. 2023 Nov 20;18(1):2281153. doi: 10.1080/15592294.2023.2281153 (PMC10732615; doi:10.1080/15592294.2023.2281153)
Supplement: Supplementary_File_3.docx [file KEPI_A_2281153_SM9091.docx]

| Site | confidence | samples_low | samples_mid | samples_high | Probe_rs | Probe_maf | CpG_rs | CpG_maf | SBE_rs | SBE_maf |
| --- | --- | --- | --- | --- | --- | --- | --- | --- | --- | --- |
| cg08313132 | 0 | 1321 | 2 | 0 | NA | NA | NA | NA | NA | NA |
| cg22403382 | 1 | 3 | 2 | 1318 | NA | NA | NA | NA | NA | NA |
| cg00557440 | 1 | 1 | 4 | 1318 | NA | NA | NA | NA | NA | NA |
| cg02847098 | 0 | 1321 | 2 | 0 | NA | NA | NA | NA | NA | NA |
| cg03044149 | 0 | 1321 | 2 | 0 | NA | NA | NA | NA | NA | NA |
| cg03150322 | 0 | 1321 | 2 | 0 | rs61736926 | 0.177306 | NA | NA | NA | NA |
| cg03152577 | 0 | 1320 | 3 | 0 | rs35601528 | 0.011182 | NA | NA | NA | NA |
| cg03255942 | 0 | 0 | 2 | 1321 | NA | NA | NA | NA | NA | NA |
| cg03331258 | 0 | 1321 | 2 | 0 | NA | NA | NA | NA | NA | NA |
| cg03940437 | 1 | 1 | 5 | 1317 | NA | NA | NA | NA | NA | NA |
| cg04562522 | 0 | 1321 | 2 | 0 | NA | NA | NA | NA | NA | NA |
| cg05638359 | 0 | 1318 | 3 | 2 | NA | NA | NA | NA | NA | NA |
| cg05806577 | 0 | 1321 | 2 | 0 | NA | NA | NA | NA | NA | NA |
| cg05821633 | 0 | 0 | 3 | 1320 | NA | NA | rs112035960 | 0.028754 | NA | NA |
| cg06982544 | 0 | 1320 | 2 | 1 | NA | NA | NA | NA | NA | NA |
| cg07030052 | 0 | 1321 | 2 | 0 | NA | NA | NA | NA | NA | NA |
| cg09858583 | 0 | 1318 | 5 | 0 | NA | NA | NA | NA | rs74155244 | 0.01857 |
| cg10057528 | 0 | 0 | 2 | 1321 | NA | NA | NA | NA | NA | NA |
| cg14993123 | 0 | 1317 | 4 | 2 | NA | NA | NA | NA | NA | NA |
| cg15482792 | 0 | 1321 | 1 | 1 | NA | NA | NA | NA | NA | NA |
| cg16011679 | 0 | 1321 | 2 | 0 | rs71652692 | 0.155751 | NA | NA | NA | NA |
| cg18107019 | 0 | 0 | 48 | 1275 | NA | NA | NA | NA | NA | NA |
| cg18539546 | 0 | 1320 | 1 | 2 | NA | NA | NA | NA | NA | NA |
| cg19755544 | 0 | 1321 | 2 | 0 | NA | NA | NA | NA | NA | NA |
| cg20999912 | 0.01 | 1312 | 2 | 9 | NA | NA | NA | NA | NA | NA |
| cg21859337 | 0 | 1321 | 2 | 0 | NA | NA | NA | NA | NA | NA |
| cg22168480 | 0 | 1318 | 5 | 0 | NA | NA | NA | NA | NA | NA |
| cg25107608 | 0 | 1320 | 3 | 0 | NA | NA | NA | NA | NA | NA |
| cg25138854 | 0 | 1317 | 6 | 0 | rs76087959 | 0.060304 | NA | NA | NA | NA |
| cg25282951 | 1 | 2 | 5 | 1316 | NA | NA | NA | NA | NA | NA |
| cg26521784 | 0 | 1321 | 2 | 0 | NA | NA | NA | NA | NA | NA |
| cg27301328 | 0 | 1321 | 2 | 0 | NA | NA | NA | NA | NA | NA |
| cg04747036 | 0 | 0 | 4 | 1319 | NA | NA | NA | NA | NA | NA |
| cg06478094 | 0 | 1321 | 2 | 0 | rs2303425 | 0.100839 | NA | NA | NA | NA |
| cg06480249 | 0 | 1320 | 3 | 0 | NA | NA | NA | NA | NA | NA |
| cg06493695 | 0 | 0 | 3 | 1320 | NA | NA | NA | NA | NA | NA |
| cg08484665 | 0 | 1321 | 2 | 0 | NA | NA | NA | NA | NA | NA |
| cg09325506 | 0 | 1321 | 2 | 0 | NA | NA | NA | NA | NA | NA |
| cg12908608 | 0 | 1321 | 2 | 0 | NA | NA | NA | NA | NA | NA |
| cg16625916 | 1 | 1 | 4 | 1318 | NA | NA | NA | NA | NA | NA |
| cg16876647 | 0 | 1319 | 4 | 0 | NA | NA | NA | NA | NA | NA |
| cg17377164 | 0 | 1321 | 1 | 1 | NA | NA | NA | NA | NA | NA |
| cg18583879 | 0 | 1320 | 2 | 1 | NA | NA | NA | NA | NA | NA |
| cg19759366 | 0 | 0 | 6 | 1317 | NA | NA | NA | NA | NA | NA |
| cg20366284 | 0 | 1318 | 2 | 3 | NA | NA | NA | NA | NA | NA |
| cg25241559 | 0.96 | 56 | 2 | 1265 | NA | NA | NA | NA | NA | NA |
| cg26297968 | 0 | 1321 | 1 | 1 | NA | NA | NA | NA | NA | NA |
| cg26347887 | 0.01 | 1311 | 1 | 11 | NA | NA | NA | NA | NA | NA |
| cg00213967 | 0 | 1321 | 1 | 1 | NA | NA | NA | NA | NA | NA |
| cg03146073 | 0 | 1321 | 2 | 0 | NA | NA | NA | NA | NA | NA |
| cg12333618 | 1 | 1 | 3 | 1319 | NA | NA | NA | NA | NA | NA |
| cg15223831 | 1 | 2 | 2 | 1319 | rs5013124 | 0.170527 | NA | NA | NA | NA |
| cg18129648 | 0 | 1321 | 1 | 1 | NA | NA | NA | NA | NA | NA |
| cg18336957 | 0 | 0 | 4 | 1319 | NA | NA | rs73142542 | 0.023163 | NA | NA |
| cg20927547 | 0 | 0 | 2 | 1321 | NA | NA | NA | NA | NA | NA |
| cg24291822 | 0 | 1320 | 3 | 0 | NA | NA | NA | NA | NA | NA |
| cg24616795 | 0 | 0 | 3 | 1320 | NA | NA | NA | NA | NA | NA |
| cg01122304 | 0 | 1320 | 3 | 0 | NA | NA | NA | NA | NA | NA |
| cg01726870 | 0 | 0 | 2 | 1321 | NA | NA | rs59557353 | 0.023762 | NA | NA |
| cg03984758 | 1 | 3 | 6 | 1314 | NA | NA | NA | NA | NA | NA |
| cg04214467 | 0 | 1321 | 2 | 0 | NA | NA | NA | NA | NA | NA |
| cg06312136 | 0 | 1321 | 2 | 0 | rs56074082 | 0.035743 | NA | NA | NA | NA |
| cg06634887 | 0 | 1320 | 2 | 1 | NA | NA | NA | NA | NA | NA |
| cg07348587 | 0 | 1321 | 2 | 0 | NA | NA | NA | NA | NA | NA |
| cg16053005 | 0 | 0 | 2 | 1321 | rs79710380 | 0.077676 | NA | NA | NA | NA |
| cg16585580 | 0 | 1321 | 1 | 1 | NA | NA | NA | NA | NA | NA |
| cg17666096 | 0 | 1321 | 2 | 0 | rs358304 | 0.063084 | NA | NA | NA | NA |
| cg19517378 | 0 | 1321 | 2 | 0 | NA | NA | NA | NA | NA | NA |
| cg21343777 | 0.99 | 4 | 14 | 1305 | NA | NA | rs113465254 | 0.072284 | NA | NA |
| cg22531663 | 0 | 1320 | 3 | 0 | NA | NA | NA | NA | NA | NA |
| cg25424031 | 1 | 1 | 2 | 1320 | NA | NA | NA | NA | NA | NA |
| cg26695837 | 0 | 1320 | 3 | 0 | NA | NA | NA | NA | NA | NA |
| cg00632914 | 0.99 | 3 | 8 | 1312 | NA | NA | NA | NA | NA | NA |
| cg05040347 | 1 | 1 | 2 | 1320 | NA | NA | NA | NA | NA | NA |
| cg18505367 | 0 | 1321 | 2 | 0 | NA | NA | NA | NA | NA | NA |
| cg19964638 | 0 | 1321 | 2 | 0 | NA | NA | NA | NA | NA | NA |
| cg22193559 | 0 | 1321 | 2 | 0 | NA | NA | NA | NA | NA | NA |
| cg25415877 | 0 | 1321 | 1 | 1 | NA | NA | NA | NA | NA | NA |
| cg26554323 | 0 | 0 | 6 | 1317 | rs2662471 | 0.278554 | NA | NA | NA | NA |
| cg00847892 | 0 | 1321 | 2 | 0 | NA | NA | NA | NA | NA | NA |
| cg00852768 | 0 | 1321 | 1 | 1 | NA | NA | NA | NA | NA | NA |
| cg01380710 | 0 | 1321 | 2 | 0 | NA | NA | NA | NA | NA | NA |
| cg01392338 | 0 | 1321 | 2 | 0 | NA | NA | NA | NA | NA | NA |
| cg06555155 | 0 | 1321 | 2 | 0 | NA | NA | NA | NA | NA | NA |
| cg06610850 | 0 | 1321 | 1 | 1 | NA | NA | NA | NA | NA | NA |
| cg07113870 | 1 | 2 | 3 | 1318 | NA | NA | NA | NA | NA | NA |
| cg08994658 | 0 | 1320 | 3 | 0 | NA | NA | NA | NA | NA | NA |
| cg10221930 | 0 | 1321 | 2 | 0 | rs17187658 | 0.011669 | NA | NA | NA | NA |
| cg10893014 | 0 | 0 | 3 | 1320 | NA | NA | NA | NA | NA | NA |
| cg12392336 | 0.99 | 5 | 4 | 1314 | rs9398635 | 0.240216 | NA | NA | NA | NA |
| cg13115683 | 0 | 1320 | 2 | 1 | rs2735076 | 0.282947 | NA | NA | NA | NA |
| cg13902446 | 0 | 1321 | 1 | 1 | rs3130455 | 0.123602 | NA | NA | NA | NA |
| cg14851271 | 0 | 0 | 3 | 1320 | NA | NA | NA | NA | NA | NA |
| cg15380588 | 0 | 1321 | 2 | 0 | NA | NA | NA | NA | NA | NA |
| cg16065270 | 0 | 1321 | 2 | 0 | NA | NA | NA | NA | NA | NA |
| cg17639394 | 0 | 1318 | 2 | 3 | rs9260836 | 0.046725 | NA | NA | NA | NA |
| cg19092529 | 1 | 1 | 2 | 1320 | rs2256183 | 0.252796 | NA | NA | NA | NA |
| cg19732154 | 0.01 | 1309 | 9 | 5 | NA | NA | NA | NA | NA | NA |
| cg20486294 | 0 | 1321 | 2 | 0 | NA | NA | NA | NA | NA | NA |
| cg23955494 | 0 | 1321 | 1 | 1 | NA | NA | NA | NA | NA | NA |
| cg02859511 | 0 | 1321 | 2 | 0 | rs10253208 | 0.021366 | NA | NA | NA | NA |
| cg03383886 | 0 | 0 | 5 | 1318 | NA | NA | NA | NA | NA | NA |
| cg04678743 | 0 | 1319 | 3 | 1 | NA | NA | NA | NA | NA | NA |
| cg07459478 | 0 | 0 | 3 | 1320 | NA | NA | NA | NA | NA | NA |
| cg09362578 | 0 | 0 | 3 | 1320 | NA | NA | rs59828898 | 0.001797 | NA | NA |
| cg10698404 | 0 | 1321 | 2 | 0 | NA | NA | NA | NA | NA | NA |
| cg15179113 | 0 | 1320 | 3 | 0 | NA | NA | NA | NA | NA | NA |
| cg15277378 | 0 | 0 | 3 | 1320 | rs12472 | 0.202276 | NA | NA | NA | NA |
| cg17588038 | 0 | 0 | 7 | 1316 | rs58370929 | 0.018371 | NA | NA | NA | NA |
| cg21304619 | 1 | 2 | 1 | 1320 | rs77562869 | 0.018371 | NA | NA | NA | NA |
| cg21787755 | 0 | 0 | 4 | 1319 | NA | NA | rs78687971 | 0.025559 | NA | NA |
| cg25591844 | 0 | 1320 | 3 | 0 | NA | NA | NA | NA | NA | NA |
| cg25618672 | 1 | 1 | 1 | 1321 | NA | NA | NA | NA | NA | NA |
| cg26390609 | 0 | 0 | 2 | 1321 | NA | NA | NA | NA | NA | NA |
| cg02693150 | 1 | 1 | 3 | 1319 | NA | NA | NA | NA | NA | NA |
| cg03471999 | 1 | 1 | 3 | 1319 | NA | NA | rs145293491 | 0.014776 | NA | NA |
| cg05892329 | 0 | 0 | 2 | 1321 | NA | NA | rs76266152 | 0.033147 | NA | NA |
| cg06763467 | 0 | 0 | 2 | 1321 | NA | NA | NA | NA | NA | NA |
| cg07135964 | 0 | 1321 | 2 | 0 | NA | NA | NA | NA | NA | NA |
| cg07276550 | 1 | 1 | 1 | 1321 | rs1016230 | 0.162939 | NA | NA | NA | NA |
| cg10473004 | 0 | 1319 | 4 | 0 | NA | NA | NA | NA | NA | NA |
| cg11372006 | 0 | 1319 | 1 | 3 | NA | NA | NA | NA | NA | NA |
| cg12292213 | 0 | 0 | 3 | 1320 | NA | NA | NA | NA | NA | NA |
| cg13514538 | 1 | 1 | 1 | 1321 | NA | NA | rs117131125 | 0.019424 | NA | NA |
| cg14446015 | 0 | 1320 | 2 | 1 | NA | NA | NA | NA | NA | NA |
| cg16100845 | 1 | 1 | 3 | 1319 | NA | NA | NA | NA | NA | NA |
| cg19727381 | 0 | 0 | 2 | 1321 | NA | NA | NA | NA | NA | NA |
| cg23139473 | 0 | 1319 | 2 | 2 | NA | NA | NA | NA | NA | NA |
| cg23191702 | 1 | 1 | 1 | 1321 | NA | NA | rs78979314 | 0.023562 | NA | NA |
| cg24701320 | 0 | 0 | 2 | 1321 | rs75318724 | 0.028992 | NA | NA | NA | NA |
| cg14242528 | 0 | 1321 | 2 | 0 | NA | NA | NA | NA | NA | NA |
| cg14363146 | 0 | 1318 | 4 | 1 | rs820495 | 0.496406 | NA | NA | NA | NA |
| cg14428669 | 0 | 1320 | 3 | 0 | rs117634485 | 0.127396 | NA | NA | NA | NA |
| cg14615537 | 0 | 0 | 3 | 1320 | NA | NA | NA | NA | NA | NA |
| cg01166071 | 0 | 1319 | 3 | 1 | NA | NA | NA | NA | NA | NA |
| cg03430923 | 0 | 1320 | 3 | 0 | NA | NA | NA | NA | NA | NA |
| cg07832903 | 0 | 1321 | 2 | 0 | NA | NA | NA | NA | NA | NA |
| cg08252499 | 0 | 1320 | 3 | 0 | NA | NA | NA | NA | NA | NA |
| cg08347183 | 0 | 1321 | 1 | 1 | NA | NA | NA | NA | NA | NA |
| cg08709029 | 0 | 1319 | 4 | 0 | rs11250244 | 0.266374 | NA | NA | NA | NA |
| cg11731793 | 1 | 2 | 5 | 1316 | rs2026555 | 0.175519 | NA | NA | NA | NA |
| cg14841842 | 0 | 1321 | 2 | 0 | rs79247418 | 0.039137 | NA | NA | NA | NA |
| cg17093401 | 0 | 1321 | 2 | 0 | NA | NA | NA | NA | NA | NA |
| cg20227259 | 0 | 1321 | 2 | 0 | NA | NA | NA | NA | NA | NA |
| cg23943136 | 0 | 1319 | 4 | 0 | NA | NA | NA | NA | NA | NA |
| cg01320969 | 0 | 1321 | 2 | 0 | NA | NA | NA | NA | NA | NA |
| cg03193454 | 0 | 1319 | 4 | 0 | rs11600264 | 0.010583 | NA | NA | NA | NA |
| cg03919694 | 0 | 1320 | 3 | 0 | NA | NA | NA | NA | NA | NA |
| cg06281265 | 1 | 2 | 1 | 1320 | NA | NA | rs117735462 | 2.00E-05 | NA | NA |
| cg07744270 | 0 | 0 | 3 | 1320 | NA | NA | NA | NA | NA | NA |
| cg09234415 | 1 | 1 | 8 | 1314 | rs117994943 | 0.010879 | NA | NA | NA | NA |
| cg10516766 | 1 | 1 | 5 | 1317 | NA | NA | NA | NA | NA | NA |
| cg11014109 | 0 | 1319 | 3 | 1 | NA | NA | NA | NA | NA | NA |
| cg11139304 | 0 | 1320 | 3 | 0 | rs73489468 | 0.015974 | NA | NA | NA | NA |
| cg13401014 | 0 | 0 | 2 | 1321 | NA | NA | NA | NA | NA | NA |
| cg16000888 | 0 | 1320 | 3 | 0 | NA | NA | NA | NA | NA | NA |
| cg18432864 | 0 | 1321 | 2 | 0 | NA | NA | NA | NA | NA | NA |
| cg19917249 | 0 | 0 | 2 | 1321 | NA | NA | NA | NA | NA | NA |
| cg20051444 | 0 | 1321 | 1 | 1 | NA | NA | NA | NA | NA | NA |
| cg20973347 | 0 | 1320 | 1 | 2 | NA | NA | NA | NA | NA | NA |
| cg03103374 | 0 | 1321 | 2 | 0 | NA | NA | NA | NA | NA | NA |
| cg03392038 | 0 | 1321 | 1 | 1 | NA | NA | NA | NA | NA | NA |
| cg03870172 | 0 | 1321 | 2 | 0 | NA | NA | NA | NA | NA | NA |
| cg08121760 | 0 | 1318 | 4 | 1 | NA | NA | NA | NA | NA | NA |
| cg08320014 | 0 | 1320 | 3 | 0 | NA | NA | NA | NA | NA | NA |
| cg09635784 | 0 | 1321 | 2 | 0 | NA | NA | NA | NA | NA | NA |
| cg13622209 | 0 | 1320 | 2 | 1 | rs12227605 | 0.219649 | NA | NA | NA | NA |
| cg14212870 | 0 | 1321 | 2 | 0 | NA | NA | NA | NA | NA | NA |
| cg14430937 | 0 | 1319 | 4 | 0 | NA | NA | NA | NA | NA | NA |
| cg16091543 | 0 | 1321 | 1 | 1 | NA | NA | NA | NA | NA | NA |
| cg16871994 | 0 | 1320 | 1 | 2 | NA | NA | NA | NA | NA | NA |
| cg19866673 | 0 | 0 | 2 | 1321 | NA | NA | NA | NA | NA | NA |
| cg21626372 | 0 | 1320 | 1 | 2 | rs7308977 | 0.076278 | NA | NA | NA | NA |
| cg22154485 | 1 | 1 | 3 | 1319 | NA | NA | NA | NA | NA | NA |
| cg26137794 | 0 | 1321 | 2 | 0 | rs71458814 | 0.272165 | NA | NA | NA | NA |
| cg27277183 | 1 | 1 | 1 | 1321 | NA | NA | NA | NA | NA | NA |
| cg16398329 | 0 | 1321 | 2 | 0 | NA | NA | NA | NA | NA | NA |
| cg24388789 | 0 | 1321 | 2 | 0 | NA | NA | NA | NA | NA | NA |
| cg03520401 | 0 | 1321 | 2 | 0 | NA | NA | NA | NA | NA | NA |
| cg03925921 | 0 | 1320 | 2 | 1 | NA | NA | rs2295833 | 0.021366 | NA | NA |
| cg05831673 | 0 | 1321 | 2 | 0 | NA | NA | NA | NA | NA | NA |
| cg10100747 | 0 | 1321 | 1 | 1 | NA | NA | NA | NA | NA | NA |
| cg10800989 | 0 | 0 | 2 | 1321 | NA | NA | NA | NA | NA | NA |
| cg12934804 | 0 | 1320 | 3 | 0 | NA | NA | NA | NA | NA | NA |
| cg14178821 | 1 | 3 | 5 | 1315 | NA | NA | NA | NA | NA | NA |
| cg14345980 | 0 | 1321 | 2 | 0 | NA | NA | NA | NA | NA | NA |
| cg15928900 | 0 | 1320 | 3 | 0 | NA | NA | NA | NA | NA | NA |
| cg18729886 | 0 | 1320 | 3 | 0 | NA | NA | NA | NA | NA | NA |
| cg21523812 | 1 | 2 | 2 | 1319 | NA | NA | NA | NA | NA | NA |
| cg23404012 | 0 | 1320 | 3 | 0 | NA | NA | NA | NA | NA | NA |
| cg23570471 | 0 | 1321 | 1 | 1 | NA | NA | NA | NA | NA | NA |
| cg26404748 | 0 | 1321 | 2 | 0 | NA | NA | NA | NA | NA | NA |
| cg08304887 | 0 | 1319 | 3 | 1 | NA | NA | NA | NA | NA | NA |
| cg12368188 | 0 | 1321 | 2 | 0 | NA | NA | NA | NA | NA | NA |
| cg18470710 | 0 | 1321 | 2 | 0 | NA | NA | NA | NA | NA | NA |
| cg20822365 | 0 | 1321 | 1 | 1 | NA | NA | NA | NA | NA | NA |
| cg25186680 | 0 | 1321 | 2 | 0 | rs2589941 | 0.322284 | NA | NA | NA | NA |
| cg00629117 | 0 | 0 | 29 | 1294 | NA | NA | NA | NA | NA | NA |
| cg00747650 | 0.99 | 2 | 12 | 1309 | NA | NA | rs80312210 | 0.091653 | NA | NA |
| cg00932688 | 0 | 1321 | 2 | 0 | NA | NA | NA | NA | NA | NA |
| cg01564943 | 0 | 0 | 2 | 1321 | rs1645351 | 0.34365 | NA | NA | NA | NA |
| cg02446681 | 0 | 1321 | 1 | 1 | NA | NA | NA | NA | NA | NA |
| cg03406609 | 0 | 0 | 4 | 1319 | NA | NA | NA | NA | NA | NA |
| cg04430866 | 0 | 0 | 3 | 1320 | NA | NA | NA | NA | NA | NA |
| cg05408370 | 0 | 1321 | 2 | 0 | rs114653492 | 0.058506 | NA | NA | NA | NA |
| cg05463941 | 0 | 1321 | 2 | 0 | NA | NA | NA | NA | NA | NA |
| cg05531174 | 1 | 1 | 9 | 1313 | NA | NA | rs113135562 | 0.048123 | NA | NA |
| cg06461811 | 0 | 1321 | 1 | 1 | NA | NA | NA | NA | NA | NA |
| cg06722589 | 0 | 1319 | 4 | 0 | NA | NA | NA | NA | NA | NA |
| cg06845639 | 1 | 1 | 6 | 1316 | NA | NA | NA | NA | NA | NA |
| cg06853823 | 0 | 1321 | 2 | 0 | NA | NA | NA | NA | NA | NA |
| cg07208513 | 0 | 1319 | 3 | 1 | NA | NA | NA | NA | NA | NA |
| cg07702637 | 0 | 0 | 2 | 1321 | NA | NA | rs112064138 | 0.01278 | NA | NA |
| cg07870854 | 0 | 1321 | 2 | 0 | NA | NA | NA | NA | NA | NA |
| cg08219183 | 0.99 | 3 | 8 | 1312 | NA | NA | NA | NA | NA | NA |
| cg08320059 | 0 | 1321 | 2 | 0 | NA | NA | NA | NA | NA | NA |
| cg09931891 | 0 | 1321 | 2 | 0 | NA | NA | NA | NA | NA | NA |
| cg26510178 | 0 | 0 | 3 | 1320 | NA | NA | NA | NA | NA | NA |
| cg26605427 | 0 | 1321 | 1 | 1 | rs3794629 | 0.310104 | NA | NA | NA | NA |
| cg03172060 | 0 | 1321 | 1 | 1 | NA | NA | NA | NA | NA | NA |
| cg09571097 | 0 | 1321 | 2 | 0 | NA | NA | NA | NA | NA | NA |
| cg13485703 | 0 | 0 | 2 | 1321 | NA | NA | NA | NA | NA | NA |
| cg13727849 | 0 | 1321 | 2 | 0 | NA | NA | NA | NA | NA | NA |
| cg13817725 | 0 | 0 | 2 | 1321 | NA | NA | NA | NA | NA | NA |
| cg16888106 | 0 | 1321 | 2 | 0 | NA | NA | NA | NA | NA | NA |
| cg17614564 | 0 | 1321 | 2 | 0 | NA | NA | NA | NA | NA | NA |
| cg18960307 | 0 | 1320 | 3 | 0 | rs3169950 | 0.449578 | NA | NA | NA | NA |
| cg19594156 | 0 | 1321 | 1 | 1 | NA | NA | NA | NA | NA | NA |
| cg20820438 | 0 | 1319 | 4 | 0 | NA | NA | NA | NA | NA | NA |
| cg21218476 | 0 | 1321 | 1 | 1 | NA | NA | NA | NA | NA | NA |
| cg22210779 | 0 | 0 | 3 | 1320 | NA | NA | NA | NA | NA | NA |
| cg24220766 | 0 | 1321 | 2 | 0 | rs115849464 | 0.011382 | NA | NA | NA | NA |
| cg24584890 | 0 | 1321 | 2 | 0 | NA | NA | NA | NA | NA | NA |
| cg25532627 | 0 | 0 | 8 | 1315 | NA | NA | NA | NA | NA | NA |
| cg26683316 | 0 | 1318 | 1 | 4 | NA | NA | NA | NA | NA | NA |
| cg26921881 | 0 | 0 | 7 | 1316 | NA | NA | NA | NA | NA | NA |
| cg26952205 | 0 | 1321 | 2 | 0 | NA | NA | NA | NA | NA | NA |
| cg05995555 | 1 | 1 | 6 | 1316 | rs62091049 | 0.045128 | NA | NA | NA | NA |
| cg04523095 | 0 | 1317 | 6 | 0 | NA | NA | NA | NA | NA | NA |
| cg06436655 | 0 | 1321 | 2 | 0 | NA | NA | NA | NA | NA | NA |
| cg06510563 | 0 | 1320 | 3 | 0 | rs113390889 | 0.026557 | NA | NA | NA | NA |
| cg06620210 | 0 | 1321 | 2 | 0 | NA | NA | NA | NA | NA | NA |
| cg10177748 | 0 | 1321 | 2 | 0 | rs75044688 | 0.003594 | NA | NA | NA | NA |
| cg11239220 | 0 | 1319 | 3 | 1 | NA | NA | NA | NA | NA | NA |
| cg11441617 | 0 | 0 | 3 | 1320 | NA | NA | NA | NA | NA | NA |
| cg11587925 | 0 | 1320 | 3 | 0 | NA | NA | NA | NA | NA | NA |
| cg14017942 | 1 | 1 | 3 | 1319 | NA | NA | NA | NA | NA | NA |
| cg14706720 | 0 | 1318 | 5 | 0 | NA | NA | NA | NA | NA | NA |
| cg18596381 | 0 | 1319 | 4 | 0 | NA | NA | NA | NA | NA | NA |
| cg20969822 | 1 | 1 | 1 | 1321 | NA | NA | NA | NA | NA | NA |
| cg24836072 | 0 | 1321 | 2 | 0 | NA | NA | NA | NA | NA | NA |
| cg26540925 | 0 | 0 | 2 | 1321 | NA | NA | NA | NA | NA | NA |
| cg01699577 | 0 | 0 | 3 | 1320 | NA | NA | NA | NA | NA | NA |
| cg14988565 | 0 | 1321 | 1 | 1 | NA | NA | NA | NA | NA | NA |
| cg16397629 | 1 | 3 | 1 | 1319 | NA | NA | NA | NA | NA | NA |
| cg20677240 | 0 | 1320 | 3 | 0 | NA | NA | rs567738841 | 0.022164 | rs549574166 | 0.021965 |
| cg14408416 | 0 | 1319 | 4 | 0 | NA | NA | NA | NA | NA | NA |
| cg18126791 | 0 | 1321 | 2 | 0 | NA | NA | NA | NA | NA | NA |
| cg07600549 | 0 | 0 | 3 | 1320 | NA | NA | NA | NA | NA | NA |
| cg22278400 | 0 | 1321 | 2 | 0 | NA | NA | NA | NA | NA | NA |
| cg22615000 | 0 | 1321 | 2 | 0 | NA | NA | NA | NA | NA | NA |
| cg00023415 | 1 | 2 | 1 | 1320 | NA | NA | NA | NA | NA | NA |
| cg00763907 | 1 | 2 | 6 | 1315 | NA | NA | NA | NA | NA | NA |
| cg01924982 | 1 | 1 | 2 | 1320 | NA | NA | NA | NA | NA | NA |
| cg01988541 | 1 | 4 | 3 | 1316 | NA | NA | NA | NA | NA | NA |
| cg03313909 | 1 | 1 | 5 | 1317 | NA | NA | NA | NA | NA | NA |
| cg07356189 | 0.99 | 5 | 13 | 1305 | NA | NA | NA | NA | NA | NA |
| cg09062995 | 1 | 1 | 1 | 1321 | NA | NA | NA | NA | NA | NA |
| cg10070728 | 1 | 1 | 5 | 1317 | NA | NA | NA | NA | NA | NA |
| cg11075227 | 0 | 1318 | 4 | 1 | NA | NA | NA | NA | NA | NA |
| cg14355748 | 0.99 | 5 | 12 | 1306 | NA | NA | NA | NA | NA | NA |
| cg18877734 | 1 | 2 | 5 | 1316 | NA | NA | NA | NA | NA | NA |
| cg21906379 | 0 | 1320 | 1 | 2 | NA | NA | NA | NA | NA | NA |
| cg22972444 | 1 | 2 | 1 | 1320 | NA | NA | NA | NA | NA | NA |
| cg24448565 | 1 | 1 | 6 | 1316 | NA | NA | NA | NA | NA | NA |
| cg26751510 | 0.98 | 9 | 30 | 1284 | NA | NA | NA | NA | NA | NA |
| cg00279700 | 1 | 2 | 3 | 1318 | NA | NA | NA | NA | NA | NA |
| cg00884805 | 1 | 2 | 6 | 1315 | NA | NA | NA | NA | NA | NA |
| cg01805856 | 0 | 0 | 3 | 1320 | NA | NA | NA | NA | NA | NA |
| cg02582194 | 1 | 1 | 2 | 1320 | NA | NA | NA | NA | NA | NA |
| cg02709178 | 0 | 0 | 4 | 1319 | NA | NA | NA | NA | NA | NA |
| cg02764188 | 0.93 | 25 | 145 | 1153 | NA | NA | rs61489571 | 0.065695 | rs61489571 | 0.065695 |
| cg02913884 | 0.03 | 1258 | 63 | 2 | NA | NA | rs74112299 | 0.021366 | rs74112299 | 0.021366 |
| cg04099562 | 0 | 1317 | 3 | 3 | NA | NA | NA | NA | NA | NA |
| cg04621353 | 0.98 | 2 | 48 | 1273 | NA | NA | rs11800791 | 0.014577 | rs11800791 | 0.014577 |
| cg05145454 | 0 | 1320 | 3 | 0 | NA | NA | NA | NA | NA | NA |
| cg05390563 | 0.01 | 1313 | 6 | 4 | NA | NA | NA | NA | NA | NA |
| cg05460975 | 0.95 | 8 | 118 | 1197 | NA | NA | rs13871 | 0.055112 | rs13871 | 0.055112 |
| cg06706670 | 0 | 0 | 4 | 1319 | NA | NA | NA | NA | NA | NA |
| cg06839383 | 0 | 0 | 28 | 1295 | NA | NA | rs3737711 | 0.017971 | rs3737711 | 0.017971 |
| cg06962428 | 0.95 | 12 | 102 | 1209 | NA | NA | rs59536348 | 0.041134 | rs59536348 | 0.041134 |
| cg07314983 | 0 | 0 | 3 | 1320 | NA | NA | NA | NA | NA | NA |
| cg07362231 | 0 | 0 | 3 | 1320 | NA | NA | NA | NA | NA | NA |
| cg07789281 | 0 | 0 | 2 | 1321 | NA | NA | NA | NA | NA | NA |
| cg07916058 | 1 | 3 | 4 | 1316 | NA | NA | NA | NA | NA | NA |
| cg08167132 | 0 | 0 | 6 | 1317 | NA | NA | NA | NA | NA | NA |
| cg08831369 | 1 | 2 | 2 | 1319 | NA | NA | NA | NA | NA | NA |
| cg09115335 | 0 | 0 | 4 | 1319 | rs59050316 | 0.020567 | NA | NA | NA | NA |
| cg09179987 | 1 | 1 | 3 | 1319 | NA | NA | NA | NA | NA | NA |
| cg09321965 | 1 | 1 | 3 | 1319 | NA | NA | NA | NA | NA | NA |
| cg09509553 | 1 | 1 | 2 | 1320 | NA | NA | NA | NA | NA | NA |
| cg09735598 | 0 | 1313 | 9 | 1 | NA | NA | NA | NA | NA | NA |
| cg10325088 | 1 | 1 | 4 | 1318 | NA | NA | NA | NA | NA | NA |
| cg10519299 | 0 | 0 | 5 | 1318 | NA | NA | NA | NA | NA | NA |
| cg10589840 | 1 | 1 | 4 | 1318 | NA | NA | NA | NA | NA | NA |
| cg10628618 | 1 | 1 | 4 | 1318 | NA | NA | NA | NA | NA | NA |
| cg10886334 | 0.99 | 1 | 12 | 1310 | rs6673211 | 0.056709 | NA | NA | NA | NA |
| cg10970349 | 0.96 | 3 | 104 | 1216 | NA | NA | rs12725787 | 0.051917 | rs12725787 | 0.051917 |
| cg11248857 | 0.95 | 8 | 108 | 1207 | NA | NA | rs7516766 | 0.043131 | rs7516766 | 0.043131 |
| cg11723698 | 0.97 | 2 | 76 | 1245 | NA | NA | rs74049517 | 0.029353 | rs74049517 | 0.029353 |
| cg11853970 | 0.97 | 2 | 76 | 1245 | NA | NA | rs28758798 | 0.026557 | rs28758798 | 0.026557 |
| cg12402831 | 1 | 1 | 7 | 1315 | NA | NA | NA | NA | NA | NA |
| cg12689888 | 1 | 1 | 4 | 1318 | NA | NA | NA | NA | NA | NA |
| cg12704672 | 0 | 0 | 3 | 1320 | NA | NA | NA | NA | NA | NA |
| cg12794131 | 1 | 1 | 2 | 1320 | rs12061713 | 0.09365 | NA | NA | NA | NA |
| cg13363608 | 0.99 | 1 | 26 | 1296 | rs75067260 | 0.013978 | NA | NA | NA | NA |
| cg13681765 | 1 | 2 | 1 | 1320 | NA | NA | rs61443570 | 0.021765 | NA | NA |
| cg14255243 | 0.97 | 4 | 83 | 1236 | NA | NA | rs116400296 | 0.020767 | rs116400296 | 0.020767 |
| cg15064681 | 1 | 1 | 2 | 1320 | rs3827737 | 0.277157 | NA | NA | NA | NA |
| cg15087740 | 1 | 2 | 6 | 1315 | NA | NA | NA | NA | NA | NA |
| cg16175124 | 1 | 2 | 3 | 1318 | NA | NA | NA | NA | NA | NA |
| cg16549063 | 1 | 1 | 4 | 1318 | NA | NA | NA | NA | NA | NA |
| cg16739247 | 0 | 1317 | 3 | 3 | NA | NA | rs61485328 | 0.011182 | NA | NA |
| cg16985301 | 1 | 1 | 4 | 1318 | NA | NA | rs114804232 | 0.041533 | NA | NA |
| cg17170133 | 0.99 | 4 | 6 | 1313 | rs114461050 | 0.013179 | NA | NA | NA | NA |
| cg17218309 | 1 | 4 | 5 | 1314 | NA | NA | NA | NA | NA | NA |
| cg17296053 | 1 | 2 | 5 | 1316 | NA | NA | NA | NA | NA | NA |
| cg17788761 | 1 | 2 | 9 | 1312 | NA | NA | NA | NA | NA | NA |
| cg17938303 | 1 | 2 | 5 | 1316 | NA | NA | NA | NA | NA | NA |
| cg18174222 | 1 | 2 | 3 | 1318 | NA | NA | NA | NA | NA | NA |
| cg19698200 | 0 | 0 | 4 | 1319 | NA | NA | NA | NA | NA | NA |
| cg19930491 | 1 | 3 | 1 | 1319 | NA | NA | NA | NA | NA | NA |
| cg20285609 | 1 | 3 | 4 | 1316 | NA | NA | NA | NA | NA | NA |
| cg20806143 | 1 | 1 | 2 | 1320 | NA | NA | NA | NA | NA | NA |
| cg21568910 | 1 | 1 | 1 | 1321 | NA | NA | NA | NA | NA | NA |
| cg21687300 | 1 | 2 | 3 | 1318 | NA | NA | NA | NA | NA | NA |
| cg22495801 | 0 | 0 | 6 | 1317 | NA | NA | NA | NA | NA | NA |
| cg24674445 | 0 | 1320 | 2 | 1 | NA | NA | NA | NA | NA | NA |
| cg25304816 | 0 | 1321 | 2 | 0 | NA | NA | NA | NA | NA | NA |
| cg25364573 | 1 | 2 | 2 | 1319 | NA | NA | NA | NA | NA | NA |
| cg25977026 | 0 | 0 | 6 | 1317 | NA | NA | NA | NA | NA | NA |
| cg26229155 | 1 | 1 | 2 | 1320 | NA | NA | NA | NA | NA | NA |
| cg26308818 | 1 | 1 | 3 | 1319 | NA | NA | NA | NA | NA | NA |
| cg26513192 | 0 | 1320 | 3 | 0 | NA | NA | NA | NA | NA | NA |
| cg27165687 | 1 | 1 | 2 | 1320 | NA | NA | NA | NA | NA | NA |
| cg27295042 | 0 | 0 | 27 | 1296 | rs114658396 | 0.016573 | NA | NA | NA | NA |
| cg27315388 | 1 | 3 | 6 | 1314 | NA | NA | NA | NA | NA | NA |
| cg27395484 | 0.99 | 5 | 6 | 1312 | rs10916129 | 0.356629 | NA | NA | NA | NA |
| cg27430402 | 1 | 1 | 2 | 1320 | NA | NA | NA | NA | NA | NA |
| cg27506377 | 1 | 1 | 4 | 1318 | rs10218589 | 0.408746 | NA | NA | NA | NA |
| cg27515966 | 0 | 1320 | 3 | 0 | NA | NA | NA | NA | NA | NA |
| cg00396604 | 1 | 1 | 1 | 1321 | NA | NA | rs74452557 | 0.023562 | NA | NA |
| cg00684529 | 0.01 | 1311 | 6 | 6 | NA | NA | NA | NA | NA | NA |
| cg01153080 | 0.98 | 1 | 40 | 1282 | NA | NA | NA | NA | NA | NA |
| cg01177002 | 1 | 1 | 2 | 1320 | NA | NA | NA | NA | NA | NA |
| cg01252760 | 0.99 | 1 | 21 | 1301 | NA | NA | NA | NA | NA | NA |
| cg01381636 | 0 | 1321 | 2 | 0 | NA | NA | NA | NA | NA | NA |
| cg01863674 | 1 | 2 | 5 | 1316 | NA | NA | NA | NA | NA | NA |
| cg03012821 | 0 | 1320 | 2 | 1 | rs4341929 | 0.055711 | NA | NA | NA | NA |
| cg03137700 | 1 | 3 | 5 | 1315 | NA | NA | NA | NA | NA | NA |
| cg03412946 | 0 | 0 | 3 | 1320 | NA | NA | NA | NA | NA | NA |
| cg03482221 | 0 | 0 | 9 | 1314 | NA | NA | NA | NA | NA | NA |
| cg05824768 | 0.99 | 1 | 29 | 1293 | NA | NA | rs114764571 | 8.00E-06 | rs114764571 | 8.00E-06 |
| cg05997278 | 0.99 | 1 | 13 | 1309 | rs72897330 | 0.01897 | NA | NA | NA | NA |
| cg06049107 | 1 | 1 | 3 | 1319 | NA | NA | NA | NA | NA | NA |
| cg06501988 | 1 | 3 | 3 | 1317 | NA | NA | NA | NA | NA | NA |
| cg06703213 | 0.92 | 25 | 153 | 1145 | NA | NA | rs36048308 | 0.02464 | rs36048308 | 0.02464 |
| cg06857837 | 0 | 0 | 3 | 1320 | rs116323950 | 0.011781 | NA | NA | NA | NA |
| cg07856295 | 0 | 1319 | 2 | 2 | NA | NA | NA | NA | NA | NA |
| cg08689708 | 1 | 2 | 8 | 1313 | NA | NA | NA | NA | NA | NA |
| cg09397319 | 1 | 3 | 4 | 1316 | NA | NA | NA | NA | NA | NA |
| cg09500565 | 1 | 1 | 2 | 1320 | NA | NA | NA | NA | NA | NA |
| cg10143030 | 0 | 0 | 5 | 1318 | NA | NA | NA | NA | NA | NA |
| cg10762533 | 0 | 0 | 6 | 1317 | NA | NA | NA | NA | NA | NA |
| cg11574665 | 0.99 | 7 | 3 | 1313 | NA | NA | rs138272815 | 0.057708 | NA | NA |
| cg11641080 | 1 | 2 | 4 | 1317 | NA | NA | NA | NA | NA | NA |
| cg11922514 | 0 | 0 | 4 | 1319 | NA | NA | NA | NA | NA | NA |
| cg12457683 | 1 | 2 | 2 | 1319 | NA | NA | NA | NA | NA | NA |
| cg13685155 | 1 | 1 | 3 | 1319 | NA | NA | NA | NA | NA | NA |
| cg13828674 | 0 | 0 | 3 | 1320 | NA | NA | NA | NA | NA | NA |
| cg13920792 | 0.97 | 4 | 81 | 1238 | NA | NA | rs28900707 | 0.032748 | rs28900707 | 0.032748 |
| cg16512708 | 0.07 | 1151 | 153 | 19 | NA | NA | rs7559215 | 0.038938 | rs7559215 | 0.038938 |
| cg16678169 | 0.09 | 1112 | 188 | 23 | NA | NA | rs2241135 | 0.089657 | rs2241135 | 0.089657 |
| cg16876636 | 1 | 1 | 5 | 1317 | NA | NA | NA | NA | NA | NA |
| cg16970492 | 1 | 3 | 5 | 1315 | NA | NA | NA | NA | NA | NA |
| cg17977362 | 0.93 | 9 | 167 | 1147 | NA | NA | rs59626274 | 0.100856 | rs59626274 | 0.100856 |
| cg18277497 | 1 | 1 | 4 | 1318 | rs117086768 | 0.014976 | NA | NA | NA | NA |
| cg18360755 | 0 | 0 | 2 | 1321 | NA | NA | NA | NA | NA | NA |
| cg18529569 | 1 | 2 | 3 | 1318 | NA | NA | NA | NA | NA | NA |
| cg18898251 | 0 | 0 | 3 | 1320 | NA | NA | rs77141720 | 0.014377 | NA | NA |
| cg19791591 | 0 | 0 | 4 | 1319 | NA | NA | NA | NA | NA | NA |
| cg19826115 | 1 | 2 | 2 | 1319 | NA | NA | NA | NA | NA | NA |
| cg20249877 | 0 | 0 | 2 | 1321 | NA | NA | NA | NA | NA | NA |
| cg21663668 | 1 | 1 | 3 | 1319 | NA | NA | NA | NA | NA | NA |
| cg22122603 | 0.01 | 1310 | 12 | 1 | NA | NA | NA | NA | NA | NA |
| cg23416937 | 1 | 1 | 4 | 1318 | NA | NA | NA | NA | NA | NA |
| cg24048263 | 1 | 1 | 3 | 1319 | NA | NA | rs34016185 | 0.023562 | NA | NA |
| cg24622439 | 1 | 2 | 6 | 1315 | NA | NA | NA | NA | NA | NA |
| cg24782949 | 1 | 2 | 1 | 1320 | NA | NA | NA | NA | NA | NA |
| cg25438788 | 0 | 0 | 2 | 1321 | NA | NA | NA | NA | NA | NA |
| cg25890678 | 1 | 1 | 4 | 1318 | NA | NA | NA | NA | NA | NA |
| cg26471058 | 0.99 | 1 | 21 | 1301 | NA | NA | NA | NA | NA | NA |
| cg27228601 | 1 | 2 | 6 | 1315 | NA | NA | NA | NA | NA | NA |
| cg27418500 | 1 | 1 | 4 | 1318 | NA | NA | NA | NA | NA | NA |
| cg27660720 | 0 | 0 | 8 | 1315 | rs13023468 | 0.204872 | NA | NA | NA | NA |
| cg00115864 | 1 | 1 | 5 | 1317 | NA | NA | NA | NA | NA | NA |
| cg00137750 | 1 | 1 | 3 | 1319 | NA | NA | NA | NA | NA | NA |
| cg00444898 | 1 | 2 | 8 | 1313 | NA | NA | NA | NA | NA | NA |
| cg01173432 | 0 | 0 | 4 | 1319 | NA | NA | NA | NA | NA | NA |
| cg02124645 | 1 | 1 | 4 | 1318 | NA | NA | NA | NA | NA | NA |
| cg02627352 | 1 | 1 | 4 | 1318 | NA | NA | NA | NA | NA | NA |
| cg03154665 | 1 | 3 | 5 | 1315 | NA | NA | NA | NA | NA | NA |
| cg03820837 | 1 | 1 | 7 | 1315 | NA | NA | NA | NA | NA | NA |
| cg04070804 | 0 | 0 | 4 | 1319 | NA | NA | NA | NA | NA | NA |
| cg04135498 | 0 | 1315 | 5 | 3 | NA | NA | NA | NA | NA | NA |
| cg04913653 | 1 | 1 | 4 | 1318 | NA | NA | NA | NA | NA | NA |
| cg05058204 | 0.01 | 1300 | 20 | 3 | NA | NA | NA | NA | NA | NA |
| cg07129714 | 1 | 1 | 5 | 1317 | NA | NA | NA | NA | NA | NA |
| cg07187607 | 0.99 | 2 | 16 | 1305 | NA | NA | NA | NA | NA | NA |
| cg07190778 | 0 | 1318 | 5 | 0 | NA | NA | NA | NA | NA | NA |
| cg08558996 | 1 | 1 | 4 | 1318 | NA | NA | NA | NA | NA | NA |
| cg08819084 | 0.99 | 1 | 12 | 1310 | NA | NA | NA | NA | NA | NA |
| cg09009983 | 1 | 1 | 5 | 1317 | NA | NA | NA | NA | NA | NA |
| cg09601150 | 1 | 1 | 2 | 1320 | NA | NA | NA | NA | NA | NA |
| cg10318621 | 1 | 1 | 5 | 1317 | NA | NA | NA | NA | NA | NA |
| cg10910666 | 1 | 1 | 2 | 1320 | NA | NA | NA | NA | NA | NA |
| cg11035303 | 0.11 | 1060 | 231 | 32 | NA | NA | rs4510359 | 0.1252 | rs4510359 | 0.1252 |
| cg11183415 | 1 | 2 | 3 | 1318 | NA | NA | NA | NA | NA | NA |
| cg11492856 | 1 | 5 | 2 | 1316 | NA | NA | NA | NA | NA | NA |
| cg12513140 | 0 | 0 | 5 | 1318 | NA | NA | NA | NA | NA | NA |
| cg12908968 | 1 | 2 | 3 | 1318 | NA | NA | NA | NA | NA | NA |
| cg13259357 | 1 | 1 | 3 | 1319 | NA | NA | NA | NA | NA | NA |
| cg13518611 | 1 | 2 | 3 | 1318 | NA | NA | NA | NA | NA | NA |
| cg14711243 | 0.03 | 1243 | 78 | 2 | NA | NA | rs62268182 | 0.002596 | rs62268182 | 0.002596 |
| cg15087376 | 1 | 3 | 6 | 1314 | NA | NA | NA | NA | NA | NA |
| cg15239694 | 0 | 0 | 32 | 1291 | NA | NA | NA | NA | NA | NA |
| cg15275017 | 1 | 2 | 5 | 1316 | NA | NA | NA | NA | NA | NA |
| cg15482530 | 1 | 1 | 5 | 1317 | rs7648844 | 0.011182 | NA | NA | NA | NA |
| cg16289175 | 0.99 | 1 | 31 | 1291 | NA | NA | rs2231219 | 0.012158 | rs2231219 | 0.012158 |
| cg16913477 | 1 | 4 | 2 | 1317 | NA | NA | NA | NA | NA | NA |
| cg16969274 | 0 | 0 | 4 | 1319 | NA | NA | NA | NA | NA | NA |
| cg17573586 | 1 | 1 | 4 | 1318 | NA | NA | NA | NA | NA | NA |
| cg18794121 | 1 | 1 | 3 | 1319 | NA | NA | NA | NA | NA | NA |
| cg19087028 | 1 | 2 | 3 | 1318 | NA | NA | NA | NA | NA | NA |
| cg19776833 | 0 | 0 | 3 | 1320 | NA | NA | NA | NA | NA | NA |
| cg20530613 | 1 | 1 | 4 | 1318 | NA | NA | NA | NA | NA | NA |
| cg20586900 | 1 | 3 | 6 | 1314 | NA | NA | NA | NA | NA | NA |
| cg20751057 | 1 | 2 | 7 | 1314 | NA | NA | NA | NA | NA | NA |
| cg21176488 | 1 | 1 | 7 | 1315 | NA | NA | NA | NA | NA | NA |
| cg21386099 | 0 | 0 | 6 | 1317 | NA | NA | NA | NA | NA | NA |
| cg21586613 | 1 | 1 | 6 | 1316 | NA | NA | NA | NA | NA | NA |
| cg21742058 | 1 | 2 | 6 | 1315 | rs115625341 | 0.042133 | NA | NA | NA | NA |
| cg22162799 | 1 | 1 | 4 | 1318 | NA | NA | NA | NA | NA | NA |
| cg22240394 | 0.99 | 1 | 31 | 1291 | rs77722160 | 0.035343 | NA | NA | NA | NA |
| cg23346146 | 1 | 1 | 2 | 1320 | NA | NA | NA | NA | NA | NA |
| cg24026331 | 1 | 1 | 4 | 1318 | NA | NA | NA | NA | NA | NA |
| cg26074940 | 1 | 2 | 8 | 1313 | rs2311298 | 0.177516 | NA | NA | NA | NA |
| cg27288595 | 1 | 1 | 6 | 1316 | NA | NA | NA | NA | NA | NA |
| cg27362167 | 0.97 | 4 | 73 | 1246 | NA | NA | rs113857695 | 0.030551 | rs113857695 | 0.030551 |
| cg00361146 | 0 | 1318 | 4 | 1 | NA | NA | NA | NA | NA | NA |
| cg00973737 | 1 | 2 | 2 | 1319 | NA | NA | NA | NA | NA | NA |
| cg01085399 | 1 | 1 | 4 | 1318 | NA | NA | NA | NA | NA | NA |
| cg01240385 | 1 | 1 | 2 | 1320 | NA | NA | rs79654997 | 0.017971 | NA | NA |
| cg01664014 | 1 | 3 | 5 | 1315 | NA | NA | rs116667533 | 0.013179 | NA | NA |
| cg01924320 | 1 | 1 | 8 | 1314 | NA | NA | NA | NA | NA | NA |
| cg01957786 | 1 | 1 | 6 | 1316 | NA | NA | NA | NA | NA | NA |
| cg02324432 | 0 | 0 | 7 | 1316 | NA | NA | NA | NA | NA | NA |
| cg02442787 | 0 | 0 | 4 | 1319 | NA | NA | NA | NA | NA | NA |
| cg02683371 | 0 | 0 | 4 | 1319 | NA | NA | NA | NA | NA | NA |
| cg02779075 | 0.01 | 1313 | 6 | 4 | NA | NA | NA | NA | NA | NA |
| cg03415001 | 1 | 1 | 1 | 1321 | rs114240612 | 0.021166 | NA | NA | NA | NA |
| cg03640426 | 1 | 2 | 5 | 1316 | NA | NA | NA | NA | NA | NA |
| cg04057161 | 1 | 2 | 7 | 1314 | NA | NA | NA | NA | NA | NA |
| cg04155747 | 1 | 1 | 4 | 1318 | NA | NA | NA | NA | NA | NA |
| cg04428346 | 0 | 0 | 4 | 1319 | NA | NA | NA | NA | NA | NA |
| cg04430637 | 0 | 1319 | 3 | 1 | NA | NA | NA | NA | NA | NA |
| cg04460007 | 1 | 2 | 6 | 1315 | NA | NA | NA | NA | NA | NA |
| cg04570669 | 1 | 3 | 4 | 1316 | NA | NA | NA | NA | NA | NA |
| cg05124242 | 1 | 2 | 6 | 1315 | NA | NA | NA | NA | NA | NA |
| cg05816041 | 1 | 3 | 1 | 1319 | NA | NA | NA | NA | NA | NA |
| cg06065089 | 1 | 4 | 3 | 1316 | NA | NA | NA | NA | NA | NA |
| cg06200982 | 1 | 1 | 7 | 1315 | NA | NA | NA | NA | NA | NA |
| cg06982357 | 0.99 | 1 | 13 | 1309 | NA | NA | NA | NA | NA | NA |
| cg07097005 | 0 | 1317 | 6 | 0 | NA | NA | NA | NA | NA | NA |
| cg07443112 | 1 | 1 | 5 | 1317 | NA | NA | NA | NA | NA | NA |
| cg07686392 | 0.98 | 3 | 60 | 1260 | NA | NA | rs73793111 | 0.021366 | rs73793111 | 0.021366 |
| cg07801506 | 0 | 0 | 4 | 1319 | NA | NA | NA | NA | NA | NA |
| cg07946300 | 1 | 1 | 5 | 1317 | NA | NA | NA | NA | NA | NA |
| cg08373477 | 0 | 0 | 5 | 1318 | NA | NA | NA | NA | NA | NA |
| cg09030852 | 0 | 0 | 4 | 1319 | NA | NA | NA | NA | NA | NA |
| cg09248897 | 1 | 1 | 5 | 1317 | rs72987731 | 0.01258 | NA | NA | NA | NA |
| cg09646593 | 0 | 0 | 4 | 1319 | NA | NA | NA | NA | NA | NA |
| cg09816180 | 1 | 2 | 5 | 1316 | NA | NA | NA | NA | NA | NA |
| cg10497345 | 1 | 1 | 9 | 1313 | NA | NA | NA | NA | NA | NA |
| cg10857203 | 1 | 2 | 4 | 1317 | NA | NA | NA | NA | NA | NA |
| cg11511184 | 1 | 5 | 2 | 1316 | rs12640224 | 0.305312 | NA | NA | NA | NA |
| cg13199720 | 0.99 | 8 | 1 | 1314 | NA | NA | NA | NA | NA | NA |
| cg14871906 | 0 | 1317 | 4 | 2 | NA | NA | NA | NA | NA | NA |
| cg15209369 | 1 | 2 | 4 | 1317 | NA | NA | NA | NA | NA | NA |
| cg15498306 | 0 | 1314 | 9 | 0 | rs17043977 | 0.022754 | NA | NA | NA | NA |
| cg16055817 | 0.99 | 1 | 23 | 1299 | NA | NA | NA | NA | NA | NA |
| cg16737267 | 0 | 0 | 4 | 1319 | NA | NA | NA | NA | NA | NA |
| cg16740092 | 1 | 1 | 2 | 1320 | NA | NA | NA | NA | NA | NA |
| cg16924658 | 1 | 1 | 6 | 1316 | NA | NA | NA | NA | NA | NA |
| cg17858192 | 0.23 | 793 | 454 | 76 | NA | NA | rs4698134 | 0.233826 | rs4698134 | 0.233826 |
| cg20993966 | 1 | 2 | 6 | 1315 | NA | NA | NA | NA | NA | NA |
| cg21059878 | 1 | 5 | 3 | 1315 | NA | NA | NA | NA | NA | NA |
| cg21605061 | 1 | 1 | 3 | 1319 | rs114469589 | 0.010783 | NA | NA | NA | NA |
| cg22260782 | 1 | 1 | 4 | 1318 | NA | NA | NA | NA | NA | NA |
| cg22597340 | 1 | 1 | 2 | 1320 | NA | NA | NA | NA | NA | NA |
| cg23412639 | 1 | 2 | 7 | 1314 | NA | NA | NA | NA | NA | NA |
| cg23756494 | 1 | 2 | 3 | 1318 | NA | NA | NA | NA | NA | NA |
| cg23804764 | 1 | 2 | 5 | 1316 | NA | NA | NA | NA | NA | NA |
| cg23818921 | 1 | 1 | 8 | 1314 | NA | NA | NA | NA | NA | NA |
| cg23903787 | 0.98 | 2 | 47 | 1274 | NA | NA | rs73197148 | 0.015974 | rs73197148 | 0.015974 |
| cg23979876 | 1 | 4 | 4 | 1315 | NA | NA | NA | NA | NA | NA |
| cg24012044 | 0 | 0 | 4 | 1319 | NA | NA | NA | NA | NA | NA |
| cg24076747 | 0 | 0 | 3 | 1320 | NA | NA | NA | NA | NA | NA |
| cg24175289 | 0 | 0 | 4 | 1319 | NA | NA | NA | NA | NA | NA |
| cg24640821 | 1 | 3 | 3 | 1317 | NA | NA | NA | NA | NA | NA |
| cg25222324 | 1 | 1 | 2 | 1320 | NA | NA | NA | NA | NA | NA |
| cg25672720 | 1 | 1 | 9 | 1313 | NA | NA | NA | NA | NA | NA |
| cg26389756 | 1 | 1 | 7 | 1315 | NA | NA | NA | NA | NA | NA |
| cg27176246 | 0 | 0 | 5 | 1318 | NA | NA | NA | NA | NA | NA |
| cg27350042 | 0.99 | 3 | 9 | 1311 | NA | NA | NA | NA | NA | NA |
| cg27362222 | 0 | 1321 | 2 | 0 | NA | NA | NA | NA | NA | NA |
| cg27474095 | 1 | 2 | 2 | 1319 | NA | NA | NA | NA | NA | NA |
| cg27493581 | 1 | 1 | 3 | 1319 | NA | NA | NA | NA | NA | NA |
| cg00526397 | 0 | 0 | 3 | 1320 | NA | NA | NA | NA | NA | NA |
| cg00563031 | 1 | 1 | 5 | 1317 | NA | NA | NA | NA | NA | NA |
| cg01045835 | 1 | 1 | 2 | 1320 | NA | NA | NA | NA | NA | NA |
| cg01107476 | 0 | 0 | 4 | 1319 | NA | NA | NA | NA | NA | NA |
| cg02314394 | 1 | 1 | 9 | 1313 | NA | NA | NA | NA | NA | NA |
| cg02358190 | 0 | 0 | 3 | 1320 | rs73765783 | 0.023163 | NA | NA | NA | NA |
| cg02429945 | 1 | 2 | 3 | 1318 | NA | NA | NA | NA | NA | NA |
| cg02622803 | 0 | 0 | 2 | 1321 | NA | NA | NA | NA | NA | NA |
| cg03703101 | 0 | 0 | 4 | 1319 | rs79884554 | 0.021366 | NA | NA | NA | NA |
| cg04488174 | 1 | 1 | 3 | 1319 | NA | NA | NA | NA | NA | NA |
| cg04641695 | 1 | 2 | 2 | 1319 | rs7711613 | 0.261581 | NA | NA | NA | NA |
| cg05149258 | 0 | 0 | 4 | 1319 | NA | NA | NA | NA | NA | NA |
| cg05666511 | 0 | 0 | 3 | 1320 | NA | NA | NA | NA | NA | NA |
| cg05919044 | 0 | 0 | 2 | 1321 | rs2609085 | 0.445487 | NA | NA | NA | NA |
| cg06449486 | 0.01 | 1308 | 14 | 1 | NA | NA | NA | NA | NA | NA |
| cg06703856 | 1 | 3 | 2 | 1318 | NA | NA | NA | NA | NA | NA |
| cg06943925 | 1 | 2 | 8 | 1313 | NA | NA | NA | NA | NA | NA |
| cg07256206 | 1 | 2 | 2 | 1319 | rs308207 | 0.064812 | NA | NA | NA | NA |
| cg08752947 | 0 | 0 | 4 | 1319 | NA | NA | rs78157131 | 0.01238 | NA | NA |
| cg08853399 | 1 | 1 | 4 | 1318 | NA | NA | NA | NA | NA | NA |
| cg08971867 | 1 | 1 | 5 | 1317 | NA | NA | NA | NA | NA | NA |
| cg09507899 | 1 | 2 | 2 | 1319 | NA | NA | NA | NA | NA | NA |
| cg09854184 | 1 | 4 | 1 | 1318 | NA | NA | NA | NA | NA | NA |
| cg10510558 | 1 | 1 | 4 | 1318 | NA | NA | NA | NA | NA | NA |
| cg11398794 | 1 | 2 | 2 | 1319 | NA | NA | NA | NA | NA | NA |
| cg11541547 | 1 | 1 | 5 | 1317 | NA | NA | NA | NA | NA | NA |
| cg11917750 | 0 | 0 | 3 | 1320 | NA | NA | NA | NA | NA | NA |
| cg12482253 | 0 | 0 | 6 | 1317 | NA | NA | rs111241729 | 0.036541 | NA | NA |
| cg12927715 | 0 | 0 | 8 | 1315 | NA | NA | NA | NA | NA | NA |
| cg14862385 | 0 | 0 | 3 | 1320 | NA | NA | NA | NA | NA | NA |
| cg15380511 | 1 | 4 | 4 | 1315 | NA | NA | NA | NA | NA | NA |
| cg15539318 | 1 | 2 | 3 | 1318 | NA | NA | NA | NA | NA | NA |
| cg15827285 | 1 | 3 | 3 | 1317 | NA | NA | NA | NA | NA | NA |
| cg16235707 | 0.01 | 1300 | 22 | 1 | NA | NA | NA | NA | NA | NA |
| cg16622514 | 0 | 0 | 4 | 1319 | NA | NA | NA | NA | NA | NA |
| cg17436293 | 0 | 0 | 6 | 1317 | NA | NA | NA | NA | NA | NA |
| cg18064842 | 1 | 3 | 6 | 1314 | rs835157 | 0.413339 | NA | NA | NA | NA |
| cg18163543 | 1 | 2 | 3 | 1318 | NA | NA | NA | NA | NA | NA |
| cg18368265 | 1 | 1 | 5 | 1317 | NA | NA | NA | NA | NA | NA |
| cg18460295 | 1 | 3 | 3 | 1317 | NA | NA | rs76798860 | 0.020168 | NA | NA |
| cg18651011 | 1 | 3 | 3 | 1317 | NA | NA | NA | NA | NA | NA |
| cg18841521 | 1 | 1 | 3 | 1319 | NA | NA | NA | NA | NA | NA |
| cg22006288 | 1 | 1 | 4 | 1318 | NA | NA | NA | NA | NA | NA |
| cg22129807 | 0.99 | 2 | 29 | 1292 | NA | NA | NA | NA | NA | NA |
| cg22556768 | 1 | 1 | 2 | 1320 | NA | NA | NA | NA | NA | NA |
| cg22813290 | 1 | 2 | 8 | 1313 | NA | NA | NA | NA | NA | NA |
| cg22816865 | 1 | 1 | 3 | 1319 | NA | NA | NA | NA | NA | NA |
| cg22851875 | 0.39 | 536 | 544 | 243 | NA | NA | rs56167783 | 0.336661 | rs56167783 | 0.336661 |
| cg23343131 | 0.99 | 5 | 11 | 1307 | NA | NA | rs114236244 | 0.061102 | NA | NA |
| cg25353752 | 0 | 0 | 3 | 1320 | NA | NA | NA | NA | NA | NA |
| cg25402706 | 1 | 1 | 3 | 1319 | NA | NA | NA | NA | NA | NA |
| cg25617137 | 0 | 0 | 4 | 1319 | NA | NA | NA | NA | NA | NA |
| cg25961952 | 1 | 2 | 4 | 1317 | NA | NA | NA | NA | NA | NA |
| cg26047920 | 0 | 1320 | 2 | 1 | NA | NA | NA | NA | NA | NA |
| cg26297198 | 1 | 1 | 3 | 1319 | NA | NA | NA | NA | NA | NA |
| cg00026776 | 1 | 1 | 4 | 1318 | rs9268199 | 0.085863 | NA | NA | NA | NA |
| cg00355447 | 1 | 2 | 3 | 1318 | rs116119686 | 0.028554 | NA | NA | NA | NA |
| cg00424166 | 1 | 1 | 4 | 1318 | NA | NA | NA | NA | NA | NA |
| cg01161376 | 0 | 0 | 5 | 1318 | NA | NA | NA | NA | NA | NA |
| cg01354851 | 0 | 0 | 9 | 1314 | rs553948 | 0.441893 | NA | NA | NA | NA |
| cg01985595 | 1 | 3 | 2 | 1318 | NA | NA | NA | NA | NA | NA |
| cg02449202 | 0 | 0 | 5 | 1318 | NA | NA | NA | NA | NA | NA |
| cg02753444 | 0.93 | 7 | 179 | 1137 | NA | NA | rs13197160 | 0.081184 | rs13197160 | 0.081184 |
| cg02777447 | 1 | 1 | 2 | 1320 | NA | NA | NA | NA | NA | NA |
| cg02814641 | 1 | 1 | 7 | 1315 | NA | NA | NA | NA | NA | NA |
| cg02926797 | 0 | 0 | 6 | 1317 | NA | NA | NA | NA | NA | NA |
| cg02933431 | 0 | 0 | 5 | 1318 | NA | NA | NA | NA | NA | NA |
| cg03559880 | 0 | 0 | 5 | 1318 | NA | NA | NA | NA | NA | NA |
| cg03611573 | 1 | 1 | 4 | 1318 | NA | NA | NA | NA | NA | NA |
| cg03735370 | 0 | 1321 | 1 | 1 | rs6928071 | 0.036571 | NA | NA | NA | NA |
| cg03881768 | 0 | 0 | 4 | 1319 | rs2267634 | 0.024161 | NA | NA | NA | NA |
| cg04083533 | 1 | 1 | 3 | 1319 | NA | NA | NA | NA | NA | NA |
| cg04174710 | 1 | 2 | 6 | 1315 | NA | NA | NA | NA | NA | NA |
| cg04561294 | 1 | 3 | 5 | 1315 | NA | NA | NA | NA | NA | NA |
| cg04641403 | 1 | 1 | 2 | 1320 | NA | NA | NA | NA | NA | NA |
| cg04703560 | 1 | 2 | 2 | 1319 | NA | NA | NA | NA | NA | NA |
| cg05338567 | 0 | 0 | 3 | 1320 | NA | NA | NA | NA | NA | NA |
| cg05472743 | 1 | 1 | 1 | 1321 | rs62415285 | 0.025959 | NA | NA | NA | NA |
| cg06559547 | 0.01 | 1314 | 4 | 5 | NA | NA | NA | NA | NA | NA |
| cg06752695 | 0 | 1317 | 3 | 3 | NA | NA | NA | NA | NA | NA |
| cg06815302 | 1 | 3 | 4 | 1316 | NA | NA | NA | NA | NA | NA |
| cg06872047 | 0 | 0 | 6 | 1317 | rs28359973 | 0.105631 | NA | NA | NA | NA |
| cg07124919 | 0.98 | 3 | 47 | 1273 | NA | NA | rs73724751 | 0.017572 | rs73724751 | 0.017572 |
| cg08333778 | 1 | 2 | 6 | 1315 | rs806424 | 0.119609 | NA | NA | NA | NA |
| cg08413657 | 0 | 1320 | 1 | 2 | NA | NA | NA | NA | NA | NA |
| cg08607209 | 1 | 2 | 6 | 1315 | NA | NA | NA | NA | NA | NA |
| cg08747369 | 1 | 2 | 3 | 1318 | rs117096786 | 0.041667 | NA | NA | NA | NA |
| cg08852035 | 1 | 1 | 8 | 1314 | NA | NA | NA | NA | NA | NA |
| cg09824782 | 0 | 1321 | 2 | 0 | NA | NA | NA | NA | NA | NA |
| cg10281924 | 0 | 0 | 2 | 1321 | NA | NA | NA | NA | NA | NA |
| cg10462643 | 1 | 3 | 2 | 1318 | NA | NA | NA | NA | NA | NA |
| cg10725301 | 1 | 1 | 6 | 1316 | rs1555047 | 0.100839 | NA | NA | NA | NA |
| cg11156733 | 1 | 3 | 3 | 1317 | NA | NA | NA | NA | NA | NA |
| cg11282844 | 0 | 1316 | 5 | 2 | NA | NA | NA | NA | NA | NA |
| cg11749508 | 1 | 1 | 3 | 1319 | NA | NA | NA | NA | NA | NA |
| cg11807143 | 1 | 3 | 5 | 1315 | NA | NA | NA | NA | NA | NA |
| cg12186203 | 1 | 1 | 4 | 1318 | NA | NA | NA | NA | NA | NA |
| cg12627901 | 1 | 1 | 5 | 1317 | NA | NA | NA | NA | NA | NA |
| cg12989745 | 1 | 1 | 5 | 1317 | NA | NA | NA | NA | NA | NA |
| cg13567282 | 1 | 1 | 2 | 1320 | NA | NA | NA | NA | NA | NA |
| cg14005827 | 1 | 1 | 2 | 1320 | NA | NA | NA | NA | NA | NA |
| cg14095637 | 0 | 1321 | 1 | 1 | rs2301750 | 0.028954 | NA | NA | NA | NA |
| cg14196824 | 1 | 1 | 1 | 1321 | rs28362679 | 0.018253 | NA | NA | NA | NA |
| cg14422906 | 1 | 4 | 3 | 1316 | rs79128930 | 0.014776 | NA | NA | NA | NA |
| cg15598276 | 0 | 0 | 32 | 1291 | NA | NA | NA | NA | NA | NA |
| cg15604733 | 0.99 | 5 | 7 | 1311 | NA | NA | rs1555969 | 0.337859 | rs1555969 | 0.337859 |
| cg15884905 | 1 | 1 | 4 | 1318 | NA | NA | NA | NA | NA | NA |
| cg16891075 | 1 | 1 | 6 | 1316 | rs6925982 | 0.045727 | NA | NA | NA | NA |
| cg17596249 | 1 | 2 | 4 | 1317 | NA | NA | NA | NA | NA | NA |
| cg17600918 | 1 | 2 | 6 | 1315 | NA | NA | NA | NA | NA | NA |
| cg18214570 | 1 | 2 | 3 | 1318 | NA | NA | NA | NA | NA | NA |
| cg19037598 | 0.99 | 3 | 8 | 1312 | rs3851197 | 0.48123 | NA | NA | NA | NA |
| cg19931206 | 1 | 3 | 5 | 1315 | NA | NA | NA | NA | NA | NA |
| cg20380424 | 0 | 0 | 5 | 1318 | rs9393987 | 0.226837 | NA | NA | NA | NA |
| cg21083089 | 0 | 0 | 2 | 1321 | NA | NA | NA | NA | NA | NA |
| cg21089903 | 0 | 1318 | 5 | 0 | NA | NA | rs41285260 | 8.00E-06 | NA | NA |
| cg21149764 | 0 | 0 | 11 | 1312 | NA | NA | NA | NA | NA | NA |
| cg21700723 | 0 | 0 | 6 | 1317 | NA | NA | NA | NA | NA | NA |
| cg22511413 | 0 | 1318 | 4 | 1 | NA | NA | NA | NA | NA | NA |
| cg22574586 | 1 | 1 | 5 | 1317 | NA | NA | NA | NA | NA | NA |
| cg22944934 | 0 | 0 | 3 | 1320 | NA | NA | NA | NA | NA | NA |
| cg23221791 | 1 | 1 | 3 | 1319 | NA | NA | NA | NA | NA | NA |
| cg23281432 | 1 | 2 | 4 | 1317 | NA | NA | NA | NA | NA | NA |
| cg23357265 | 0 | 1321 | 2 | 0 | rs3132090 | 0.034545 | NA | NA | NA | NA |
| cg23464833 | 0 | 0 | 5 | 1318 | NA | NA | NA | NA | NA | NA |
| cg24117910 | 0.99 | 1 | 14 | 1308 | NA | NA | NA | NA | NA | NA |
| cg24320816 | 0 | 0 | 2 | 1321 | NA | NA | NA | NA | NA | NA |
| cg24656388 | 1 | 2 | 3 | 1318 | rs12197549 | 0.153355 | NA | NA | NA | NA |
| cg24888609 | 0.96 | 8 | 83 | 1232 | NA | NA | rs2797362 | 0.035144 | rs2797362 | 0.035144 |
| cg24950003 | 1 | 3 | 4 | 1316 | NA | NA | NA | NA | NA | NA |
| cg24962268 | 0 | 0 | 7 | 1316 | rs62395799 | 0.170327 | NA | NA | NA | NA |
| cg25758242 | 0 | 0 | 6 | 1317 | NA | NA | NA | NA | NA | NA |
| cg25927551 | 1 | 1 | 2 | 1320 | rs9258215 | 0.217053 | NA | NA | NA | NA |
| cg26272879 | 1 | 1 | 2 | 1320 | NA | NA | NA | NA | NA | NA |
| cg26566189 | 0.11 | 1057 | 244 | 22 | NA | NA | rs3909111 | 0.135982 | rs3909111 | 0.135982 |
| cg26569800 | 1 | 1 | 3 | 1319 | NA | NA | NA | NA | NA | NA |
| cg26889844 | 1 | 2 | 3 | 1318 | NA | NA | NA | NA | NA | NA |
| cg27271975 | 1 | 1 | 4 | 1318 | NA | NA | NA | NA | NA | NA |
| cg27364012 | 1 | 1 | 5 | 1317 | NA | NA | NA | NA | NA | NA |
| cg00653041 | 0 | 0 | 4 | 1319 | rs6947532 | 0.156749 | NA | NA | NA | NA |
| cg00968020 | 1 | 1 | 4 | 1318 | rs369601304 | 0.010184 | NA | NA | NA | NA |
| cg01028379 | 0 | 0 | 2 | 1321 | NA | NA | NA | NA | NA | NA |
| cg02173094 | 1 | 1 | 3 | 1319 | NA | NA | rs117764968 | 0.02143 | NA | NA |
| cg02774963 | 0 | 0 | 4 | 1319 | NA | NA | NA | NA | NA | NA |
| cg02988763 | 1 | 3 | 4 | 1316 | NA | NA | NA | NA | NA | NA |
| cg03033398 | 1 | 1 | 8 | 1314 | NA | NA | rs61746598 | 8.00E-06 | NA | NA |
| cg03530573 | 0 | 0 | 7 | 1316 | NA | NA | NA | NA | NA | NA |
| cg03962846 | 0 | 0 | 4 | 1319 | NA | NA | NA | NA | NA | NA |
| cg04086327 | 1 | 3 | 2 | 1318 | NA | NA | NA | NA | NA | NA |
| cg04725144 | 0.96 | 4 | 90 | 1229 | NA | NA | rs73176461 | 0.028554 | rs73176461 | 0.028554 |
| cg04752818 | 0.93 | 16 | 156 | 1151 | NA | NA | rs77986425 | 0.064297 | rs77986425 | 0.064297 |
| cg05795235 | 1 | 1 | 6 | 1316 | NA | NA | NA | NA | NA | NA |
| cg06690825 | 0 | 0 | 3 | 1320 | NA | NA | NA | NA | NA | NA |
| cg07040517 | 1 | 1 | 5 | 1317 | rs40942 | 0.287939 | NA | NA | NA | NA |
| cg08230910 | 1 | 1 | 11 | 1311 | NA | NA | NA | NA | NA | NA |
| cg08323135 | 0.99 | 5 | 4 | 1314 | NA | NA | NA | NA | NA | NA |
| cg08365802 | 0 | 0 | 3 | 1320 | NA | NA | NA | NA | NA | NA |
| cg08688636 | 0 | 0 | 5 | 1318 | NA | NA | rs58350964 | 0.021566 | NA | NA |
| cg10030633 | 0.92 | 7 | 201 | 1115 | NA | NA | rs1558461 | 0.076078 | rs1558461 | 0.076078 |
| cg10211530 | 0 | 0 | 9 | 1314 | rs184782070 | 0.01258 | NA | NA | NA | NA |
| cg11001670 | 1 | 1 | 8 | 1314 | NA | NA | NA | NA | NA | NA |
| cg11276189 | 0.9 | 12 | 241 | 1070 | NA | NA | rs78139649 | 0.100639 | rs78139649 | 0.100639 |
| cg11493553 | 0 | 0 | 6 | 1317 | NA | NA | NA | NA | NA | NA |
| cg12737520 | 1 | 2 | 2 | 1319 | NA | NA | NA | NA | NA | NA |
| cg13643060 | 1 | 1 | 2 | 1320 | NA | NA | NA | NA | NA | NA |
| cg13661703 | 0 | 0 | 6 | 1317 | rs6968182 | 0.041533 | NA | NA | NA | NA |
| cg13752708 | 0.96 | 3 | 90 | 1230 | NA | NA | rs58954728 | 0.044329 | rs58954728 | 0.044329 |
| cg13865610 | 1 | 2 | 3 | 1318 | NA | NA | NA | NA | NA | NA |
| cg14527108 | 1 | 2 | 6 | 1315 | NA | NA | NA | NA | NA | NA |
| cg14576805 | 1 | 2 | 6 | 1315 | NA | NA | NA | NA | NA | NA |
| cg15592143 | 1 | 1 | 2 | 1320 | NA | NA | NA | NA | NA | NA |
| cg15952586 | 1 | 3 | 1 | 1319 | rs10281407 | 0.093051 | NA | NA | NA | NA |
| cg16686273 | 0.98 | 1 | 38 | 1284 | NA | NA | NA | NA | NA | NA |
| cg16805994 | 0 | 1320 | 2 | 1 | NA | NA | NA | NA | NA | NA |
| cg16969077 | 0 | 0 | 4 | 1319 | NA | NA | NA | NA | NA | NA |
| cg17187143 | 0.99 | 2 | 27 | 1294 | NA | NA | rs115521219 | 0.015375 | rs115521219 | 0.015375 |
| cg17803235 | 0.94 | 15 | 117 | 1191 | NA | NA | rs7800516 | 0.069289 | rs7800516 | 0.069289 |
| cg18124764 | 0 | 0 | 3 | 1320 | NA | NA | NA | NA | NA | NA |
| cg18553202 | 0.99 | 4 | 6 | 1313 | NA | NA | NA | NA | NA | NA |
| cg18577864 | 1 | 2 | 3 | 1318 | NA | NA | NA | NA | NA | NA |
| cg18964251 | 1 | 3 | 3 | 1317 | NA | NA | NA | NA | NA | NA |
| cg20160729 | 1 | 2 | 3 | 1318 | rs150298353 | 0.011382 | NA | NA | NA | NA |
| cg20513448 | 1 | 1 | 4 | 1318 | NA | NA | NA | NA | NA | NA |
| cg20902566 | 0 | 0 | 3 | 1320 | NA | NA | NA | NA | NA | NA |
| cg21618501 | 0 | 0 | 2 | 1321 | NA | NA | NA | NA | NA | NA |
| cg22850258 | 0.98 | 2 | 57 | 1264 | NA | NA | rs113282573 | 0.031749 | rs113282573 | 0.031749 |
| cg24652817 | 1 | 2 | 3 | 1318 | NA | NA | NA | NA | NA | NA |
| cg24946941 | 1 | 1 | 2 | 1320 | NA | NA | NA | NA | NA | NA |
| cg25252443 | 0 | 0 | 4 | 1319 | NA | NA | rs117616815 | 0.019768 | NA | NA |
| cg25453914 | 1 | 2 | 2 | 1319 | NA | NA | NA | NA | NA | NA |
| cg26448609 | 1 | 2 | 6 | 1315 | NA | NA | NA | NA | NA | NA |
| cg26706652 | 0 | 0 | 2 | 1321 | NA | NA | NA | NA | NA | NA |
| cg26815617 | 0 | 0 | 6 | 1317 | NA | NA | NA | NA | NA | NA |
| cg27636594 | 0 | 0 | 5 | 1318 | NA | NA | NA | NA | NA | NA |
| cg00021152 | 1 | 1 | 2 | 1320 | NA | NA | NA | NA | NA | NA |
| cg00032610 | 0 | 0 | 5 | 1318 | NA | NA | NA | NA | NA | NA |
| cg00133629 | 0 | 0 | 5 | 1318 | rs1995947 | 0.050919 | NA | NA | NA | NA |
| cg00252803 | 1 | 1 | 9 | 1313 | NA | NA | NA | NA | NA | NA |
| cg00997969 | 1 | 1 | 3 | 1319 | NA | NA | NA | NA | NA | NA |
| cg01725759 | 0 | 0 | 4 | 1319 | NA | NA | NA | NA | NA | NA |
| cg01898817 | 1 | 1 | 2 | 1320 | NA | NA | rs79620754 | 0.031949 | NA | NA |
| cg02017814 | 1 | 2 | 3 | 1318 | NA | NA | NA | NA | NA | NA |
| cg02151535 | 1 | 1 | 7 | 1315 | NA | NA | NA | NA | NA | NA |
| cg02335844 | 0 | 0 | 3 | 1320 | rs12542312 | 0.321885 | NA | NA | NA | NA |
| cg03599855 | 1 | 1 | 3 | 1319 | NA | NA | NA | NA | NA | NA |
| cg03978067 | 0.93 | 8 | 164 | 1151 | NA | NA | rs58477175 | 0.088459 | rs58477175 | 0.088459 |
| cg04177885 | 0 | 0 | 2 | 1321 | rs17089244 | 0.184027 | NA | NA | NA | NA |
| cg04403442 | 1 | 1 | 3 | 1319 | NA | NA | NA | NA | NA | NA |
| cg05873496 | 1 | 2 | 5 | 1316 | NA | NA | NA | NA | NA | NA |
| cg06521347 | 1 | 2 | 8 | 1313 | NA | NA | NA | NA | NA | NA |
| cg06890813 | 0 | 0 | 7 | 1316 | NA | NA | NA | NA | NA | NA |
| cg08327106 | 0 | 1318 | 5 | 0 | NA | NA | NA | NA | NA | NA |
| cg08383654 | 0.99 | 5 | 5 | 1313 | NA | NA | rs35084958 | 0.032947 | NA | NA |
| cg08542066 | 0.85 | 41 | 302 | 980 | NA | NA | rs7842432 | 0.147364 | rs7842432 | 0.147364 |
| cg08840751 | 1 | 1 | 2 | 1320 | NA | NA | NA | NA | NA | NA |
| cg09704885 | 1 | 1 | 3 | 1319 | NA | NA | NA | NA | NA | NA |
| cg09852619 | 0 | 0 | 3 | 1320 | rs17053452 | 0.238019 | NA | NA | NA | NA |
| cg10606061 | 0 | 0 | 5 | 1318 | NA | NA | NA | NA | NA | NA |
| cg11211624 | 0.98 | 1 | 51 | 1271 | NA | NA | rs116979818 | 0.025959 | rs116979818 | 0.025959 |
| cg12564648 | 1 | 2 | 4 | 1317 | NA | NA | NA | NA | NA | NA |
| cg12981288 | 1 | 2 | 3 | 1318 | NA | NA | NA | NA | NA | NA |
| cg14750367 | 0 | 1318 | 4 | 1 | NA | NA | NA | NA | NA | NA |
| cg14895374 | 0.99 | 1 | 17 | 1305 | NA | NA | NA | NA | NA | NA |
| cg19366147 | 0 | 0 | 3 | 1320 | NA | NA | NA | NA | NA | NA |
| cg20071227 | 1 | 3 | 3 | 1317 | NA | NA | NA | NA | NA | NA |
| cg22991992 | 1 | 1 | 5 | 1317 | NA | NA | NA | NA | NA | NA |
| cg23983141 | 0.98 | 4 | 48 | 1271 | NA | NA | rs77618860 | 0.014577 | rs77618860 | 0.014577 |
| cg25545598 | 0 | 0 | 6 | 1317 | NA | NA | NA | NA | NA | NA |
| cg26719638 | 1 | 1 | 9 | 1313 | NA | NA | NA | NA | NA | NA |
| cg27476810 | 0 | 1319 | 2 | 2 | NA | NA | NA | NA | NA | NA |
| cg09307883 | 0.87 | 34 | 268 | 1021 | NA | NA | rs11549105 | 1.60E-05 | rs11549105 | 1.60E-05 |
| cg13696535 | 1 | 1 | 5 | 1317 | rs113062245 | 0.028554 | NA | NA | NA | NA |
| cg13749266 | 1 | 3 | 2 | 1318 | NA | NA | NA | NA | NA | NA |
| cg13831848 | 1 | 2 | 4 | 1317 | rs116388354 | 0.015775 | NA | NA | NA | NA |
| cg13921686 | 0 | 0 | 4 | 1319 | rs78094405 | 0.001997 | NA | NA | NA | NA |
| cg14398353 | 1 | 1 | 3 | 1319 | NA | NA | NA | NA | NA | NA |
| cg14534337 | 0 | 0 | 5 | 1318 | NA | NA | NA | NA | NA | NA |
| cg14583815 | 0 | 0 | 6 | 1317 | NA | NA | NA | NA | NA | NA |
| cg14651896 | 0 | 0 | 2 | 1321 | NA | NA | NA | NA | NA | NA |
| cg25018881 | 1 | 2 | 4 | 1317 | NA | NA | NA | NA | NA | NA |
| cg00779615 | 0 | 0 | 8 | 1315 | rs72816130 | 0.025559 | NA | NA | NA | NA |
| cg01317050 | 1 | 3 | 3 | 1317 | NA | NA | NA | NA | NA | NA |
| cg02401524 | 0.97 | 1 | 76 | 1246 | NA | NA | rs28971475 | 0.032348 | rs28971475 | 0.032348 |
| cg02875589 | 1 | 1 | 4 | 1318 | rs7091768 | 0.476837 | NA | NA | NA | NA |
| cg02906915 | 1 | 1 | 5 | 1317 | rs74893196 | 0.024561 | NA | NA | NA | NA |
| cg03712837 | 1 | 1 | 6 | 1316 | NA | NA | NA | NA | NA | NA |
| cg04570208 | 0.98 | 1 | 44 | 1278 | NA | NA | rs117109515 | 0.021566 | rs117109515 | 0.021566 |
| cg04650656 | 1 | 2 | 3 | 1318 | NA | NA | NA | NA | NA | NA |
| cg06547034 | 0 | 0 | 4 | 1319 | NA | NA | NA | NA | NA | NA |
| cg07029975 | 0 | 0 | 5 | 1318 | NA | NA | NA | NA | NA | NA |
| cg07180427 | 0 | 1313 | 7 | 3 | NA | NA | NA | NA | NA | NA |
| cg08287211 | 1 | 1 | 5 | 1317 | NA | NA | NA | NA | NA | NA |
| cg10924085 | 0.99 | 1 | 31 | 1291 | NA | NA | NA | NA | NA | NA |
| cg10949880 | 1 | 5 | 3 | 1315 | NA | NA | NA | NA | NA | NA |
| cg11495351 | 1 | 1 | 3 | 1319 | NA | NA | NA | NA | NA | NA |
| cg11881391 | 0.99 | 3 | 10 | 1310 | NA | NA | NA | NA | NA | NA |
| cg12092413 | 1 | 1 | 5 | 1317 | NA | NA | NA | NA | NA | NA |
| cg13654884 | 1 | 5 | 3 | 1315 | NA | NA | NA | NA | NA | NA |
| cg14405439 | 1 | 1 | 1 | 1321 | NA | NA | rs114089626 | 0.018171 | NA | NA |
| cg17026838 | 1 | 2 | 4 | 1317 | NA | NA | NA | NA | NA | NA |
| cg17053251 | 0 | 1318 | 4 | 1 | NA | NA | NA | NA | NA | NA |
| cg17504453 | 0.98 | 1 | 44 | 1278 | NA | NA | rs76227307 | 0.021965 | rs76227307 | 0.021965 |
| cg17614262 | 0 | 1321 | 1 | 1 | rs114189190 | 0.017572 | NA | NA | NA | NA |
| cg18599069 | 0 | 1320 | 3 | 0 | NA | NA | NA | NA | NA | NA |
| cg18876706 | 0.99 | 1 | 37 | 1285 | NA | NA | rs113228253 | 0.015176 | rs113228253 | 0.015176 |
| cg22647566 | 1 | 4 | 2 | 1317 | NA | NA | NA | NA | NA | NA |
| cg23804261 | 0 | 0 | 3 | 1320 | NA | NA | NA | NA | NA | NA |
| cg23958457 | 0 | 1319 | 4 | 0 | NA | NA | NA | NA | NA | NA |
| cg24767634 | 0 | 0 | 3 | 1320 | NA | NA | NA | NA | NA | NA |
| cg25823934 | 0 | 0 | 6 | 1317 | NA | NA | NA | NA | NA | NA |
| cg26507175 | 0 | 0 | 3 | 1320 | NA | NA | rs75877050 | 0.030751 | NA | NA |
| cg26749475 | 0 | 0 | 5 | 1318 | NA | NA | NA | NA | NA | NA |
| cg27405799 | 1 | 1 | 3 | 1319 | NA | NA | NA | NA | NA | NA |
| cg00481637 | 1 | 3 | 3 | 1317 | rs2736533 | 0.345647 | NA | NA | NA | NA |
| cg01453529 | 0 | 0 | 4 | 1319 | NA | NA | NA | NA | NA | NA |
| cg01752322 | 0.99 | 1 | 14 | 1308 | NA | NA | NA | NA | NA | NA |
| cg02297591 | 0 | 0 | 6 | 1317 | NA | NA | NA | NA | NA | NA |
| cg06013329 | 0.99 | 1 | 25 | 1297 | NA | NA | NA | NA | NA | NA |
| cg06539921 | 1 | 3 | 2 | 1318 | NA | NA | NA | NA | NA | NA |
| cg06870124 | 1 | 2 | 6 | 1315 | NA | NA | NA | NA | NA | NA |
| cg07297608 | 0.98 | 2 | 46 | 1275 | NA | NA | rs114061181 | 0.016374 | rs114061181 | 0.016374 |
| cg07347276 | 1 | 6 | 1 | 1316 | NA | NA | NA | NA | NA | NA |
| cg08765357 | 1 | 2 | 4 | 1317 | NA | NA | NA | NA | NA | NA |
| cg09373227 | 0 | 0 | 4 | 1319 | NA | NA | NA | NA | NA | NA |
| cg09500207 | 1 | 1 | 4 | 1318 | NA | NA | rs77048361 | 0.022963 | NA | NA |
| cg10098373 | 0.91 | 16 | 218 | 1089 | NA | NA | rs2924682 | 0.110423 | rs2924682 | 0.110423 |
| cg10983878 | 0 | 0 | 4 | 1319 | NA | NA | NA | NA | NA | NA |
| cg11715147 | 0 | 0 | 3 | 1320 | NA | NA | NA | NA | NA | NA |
| cg13824097 | 0 | 0 | 44 | 1279 | NA | NA | rs116659213 | 0.015375 | rs116659213 | 0.015375 |
| cg15716757 | 0 | 0 | 4 | 1319 | NA | NA | NA | NA | NA | NA |
| cg17296447 | 0 | 0 | 3 | 1320 | NA | NA | NA | NA | NA | NA |
| cg18289710 | 1 | 3 | 6 | 1314 | rs56343845 | 0.028954 | NA | NA | NA | NA |
| cg19557407 | 0 | 0 | 4 | 1319 | NA | NA | NA | NA | NA | NA |
| cg19574514 | 0 | 0 | 11 | 1312 | NA | NA | NA | NA | NA | NA |
| cg19712573 | 1 | 2 | 6 | 1315 | NA | NA | NA | NA | NA | NA |
| cg19778737 | 1 | 2 | 5 | 1316 | rs56336128 | 0.210064 | NA | NA | NA | NA |
| cg19934382 | 0 | 0 | 5 | 1318 | NA | NA | NA | NA | NA | NA |
| cg22272056 | 1 | 2 | 5 | 1316 | NA | NA | NA | NA | NA | NA |
| cg23878202 | 0.98 | 2 | 56 | 1265 | NA | NA | rs116292038 | 0.026158 | rs116292038 | 0.026158 |
| cg24815999 | 1 | 2 | 2 | 1319 | NA | NA | NA | NA | NA | NA |
| cg25037548 | 0 | 0 | 6 | 1317 | NA | NA | NA | NA | NA | NA |
| cg25414537 | 0.98 | 3 | 35 | 1285 | NA | NA | rs71471407 | 0.011637 | rs71471407 | 0.011637 |
| cg26222765 | 0.98 | 2 | 52 | 1269 | NA | NA | rs35643755 | 0.016174 | rs35643755 | 0.016174 |
| cg26985134 | 1 | 1 | 5 | 1317 | NA | NA | NA | NA | NA | NA |
| cg01179055 | 1 | 1 | 7 | 1315 | NA | NA | NA | NA | NA | NA |
| cg01481569 | 1 | 1 | 4 | 1318 | NA | NA | NA | NA | NA | NA |
| cg02340615 | 1 | 1 | 3 | 1319 | NA | NA | NA | NA | NA | NA |
| cg02365078 | 0 | 1321 | 2 | 0 | NA | NA | NA | NA | NA | NA |
| cg02977682 | 0.99 | 1 | 19 | 1303 | NA | NA | NA | NA | NA | NA |
| cg03138863 | 0 | 0 | 3 | 1320 | NA | NA | NA | NA | NA | NA |
| cg03535648 | 1 | 3 | 4 | 1316 | NA | NA | NA | NA | NA | NA |
| cg03804255 | 0 | 0 | 5 | 1318 | NA | NA | NA | NA | NA | NA |
| cg04029483 | 0 | 0 | 6 | 1317 | NA | NA | NA | NA | NA | NA |
| cg04118741 | 1 | 1 | 3 | 1319 | NA | NA | NA | NA | NA | NA |
| cg04319429 | 1 | 4 | 4 | 1315 | NA | NA | NA | NA | NA | NA |
| cg04559293 | 1 | 1 | 4 | 1318 | NA | NA | NA | NA | NA | NA |
| cg05168215 | 1 | 2 | 5 | 1316 | NA | NA | NA | NA | NA | NA |
| cg05179921 | 1 | 1 | 6 | 1316 | NA | NA | NA | NA | NA | NA |
| cg05590294 | 0 | 1317 | 3 | 3 | NA | NA | NA | NA | NA | NA |
| cg05770947 | 1 | 1 | 4 | 1318 | NA | NA | NA | NA | NA | NA |
| cg06352932 | 0 | 0 | 7 | 1316 | NA | NA | NA | NA | NA | NA |
| cg07729842 | 1 | 2 | 3 | 1318 | NA | NA | NA | NA | NA | NA |
| cg08501402 | 0 | 0 | 5 | 1318 | NA | NA | NA | NA | NA | NA |
| cg09039845 | 0.99 | 1 | 21 | 1301 | NA | NA | NA | NA | NA | NA |
| cg10990959 | 1 | 1 | 4 | 1318 | NA | NA | NA | NA | NA | NA |
| cg11701583 | 0.07 | 1152 | 160 | 11 | NA | NA | rs11829843 | 0.060304 | rs11829843 | 0.060304 |
| cg12688217 | 1 | 1 | 2 | 1320 | NA | NA | NA | NA | NA | NA |
| cg13038229 | 1 | 3 | 4 | 1316 | rs7965450 | 0.114816 | NA | NA | NA | NA |
| cg13173541 | 0 | 0 | 2 | 1321 | NA | NA | NA | NA | NA | NA |
| cg13478974 | 1 | 1 | 3 | 1319 | NA | NA | NA | NA | NA | NA |
| cg14579651 | 1 | 3 | 7 | 1313 | NA | NA | NA | NA | NA | NA |
| cg14864782 | 0.98 | 2 | 39 | 1282 | NA | NA | rs74324489 | 0.012979 | rs74324489 | 0.012979 |
| cg15993521 | 1 | 4 | 3 | 1316 | NA | NA | NA | NA | NA | NA |
| cg17849509 | 1 | 1 | 2 | 1320 | NA | NA | NA | NA | NA | NA |
| cg18559471 | 1 | 1 | 5 | 1317 | NA | NA | NA | NA | NA | NA |
| cg18602710 | 0 | 0 | 3 | 1320 | NA | NA | NA | NA | NA | NA |
| cg19121684 | 0 | 0 | 5 | 1318 | NA | NA | NA | NA | NA | NA |
| cg19647197 | 0.99 | 5 | 4 | 1314 | NA | NA | NA | NA | NA | NA |
| cg19880901 | 0.99 | 1 | 19 | 1303 | NA | NA | NA | NA | NA | NA |
| cg21381779 | 1 | 1 | 3 | 1319 | NA | NA | NA | NA | NA | NA |
| cg22190774 | 0 | 1316 | 3 | 4 | NA | NA | NA | NA | NA | NA |
| cg22800453 | 0.99 | 2 | 23 | 1298 | NA | NA | NA | NA | NA | NA |
| cg23245485 | 0.99 | 2 | 18 | 1303 | NA | NA | NA | NA | NA | NA |
| cg23261846 | 1 | 2 | 3 | 1318 | NA | NA | NA | NA | NA | NA |
| cg23680411 | 1 | 2 | 6 | 1315 | NA | NA | NA | NA | NA | NA |
| cg24171152 | 1 | 1 | 1 | 1321 | NA | NA | NA | NA | NA | NA |
| cg24824245 | 1 | 2 | 1 | 1320 | NA | NA | NA | NA | NA | NA |
| cg26124503 | 1 | 1 | 6 | 1316 | NA | NA | NA | NA | NA | NA |
| cg26433955 | 1 | 3 | 3 | 1317 | NA | NA | rs79818720 | 0.028355 | NA | NA |
| cg27157669 | 0 | 0 | 27 | 1296 | NA | NA | rs143070721 | 0.01278 | rs143070721 | 0.01278 |
| cg00655498 | 1 | 3 | 1 | 1319 | NA | NA | NA | NA | NA | NA |
| cg02629791 | 1 | 2 | 5 | 1316 | NA | NA | NA | NA | NA | NA |
| cg02673129 | 1 | 1 | 2 | 1320 | NA | NA | NA | NA | NA | NA |
| cg03026406 | 1 | 3 | 1 | 1319 | NA | NA | NA | NA | NA | NA |
| cg03249710 | 0 | 1321 | 2 | 0 | NA | NA | NA | NA | NA | NA |
| cg05493945 | 1 | 1 | 9 | 1313 | rs9514544 | 0.478834 | NA | NA | NA | NA |
| cg05810731 | 1 | 2 | 7 | 1314 | NA | NA | NA | NA | NA | NA |
| cg07583690 | 1 | 3 | 3 | 1317 | NA | NA | rs80231507 | 0.015974 | NA | NA |
| cg08346664 | 0.02 | 1259 | 63 | 1 | NA | NA | rs79606767 | 0.015775 | rs79606767 | 0.015775 |
| cg09394488 | 1 | 2 | 5 | 1316 | NA | NA | NA | NA | NA | NA |
| cg11008571 | 0.99 | 1 | 15 | 1307 | NA | NA | NA | NA | NA | NA |
| cg11307812 | 1 | 1 | 4 | 1318 | NA | NA | NA | NA | NA | NA |
| cg12895304 | 1 | 1 | 3 | 1319 | NA | NA | NA | NA | NA | NA |
| cg13095768 | 0 | 0 | 5 | 1318 | NA | NA | NA | NA | NA | NA |
| cg14664094 | 1 | 1 | 3 | 1319 | rs9525270 | 0.034345 | NA | NA | NA | NA |
| cg17598724 | 0 | 0 | 6 | 1317 | rs17067518 | 0.018407 | NA | NA | NA | NA |
| cg18445718 | 1 | 2 | 5 | 1316 | NA | NA | NA | NA | NA | NA |
| cg19937572 | 0 | 0 | 5 | 1318 | NA | NA | NA | NA | NA | NA |
| cg22117688 | 1 | 1 | 4 | 1318 | NA | NA | NA | NA | NA | NA |
| cg23052386 | 1 | 2 | 6 | 1315 | NA | NA | NA | NA | NA | NA |
| cg23168520 | 0.88 | 38 | 235 | 1050 | NA | NA | rs9525308 | 0.135383 | rs9525308 | 0.135383 |
| cg24685028 | 1 | 1 | 3 | 1319 | NA | NA | NA | NA | NA | NA |
| cg24776343 | 0 | 0 | 3 | 1320 | rs57216713 | 0.010583 | NA | NA | NA | NA |
| cg25054777 | 1 | 1 | 4 | 1318 | NA | NA | NA | NA | NA | NA |
| cg25492213 | 1 | 2 | 7 | 1314 | NA | NA | NA | NA | NA | NA |
| cg26434653 | 1 | 1 | 2 | 1320 | NA | NA | NA | NA | NA | NA |
| cg26791649 | 1 | 2 | 6 | 1315 | NA | NA | NA | NA | NA | NA |
| cg00457115 | 0.98 | 1 | 41 | 1281 | NA | NA | rs75681836 | 0.021965 | rs75681836 | 0.021965 |
| cg00649500 | 1 | 2 | 5 | 1316 | NA | NA | NA | NA | NA | NA |
| cg02259743 | 0 | 1318 | 4 | 1 | NA | NA | NA | NA | NA | NA |
| cg02898977 | 0.92 | 18 | 187 | 1118 | NA | NA | rs10583 | 0.105536 | rs10583 | 0.105536 |
| cg03591223 | 0 | 1319 | 4 | 0 | rs10144905 | 0.027556 | NA | NA | NA | NA |
| cg05402364 | 0.99 | 4 | 7 | 1312 | NA | NA | NA | NA | NA | NA |
| cg05416141 | 0.99 | 5 | 6 | 1312 | NA | NA | NA | NA | NA | NA |
| cg07140250 | 0.99 | 2 | 34 | 1287 | NA | NA | NA | NA | NA | NA |
| cg07472806 | 0.99 | 6 | 13 | 1304 | NA | NA | NA | NA | NA | NA |
| cg08221288 | 1 | 2 | 3 | 1318 | NA | NA | NA | NA | NA | NA |
| cg09924366 | 0.97 | 4 | 77 | 1242 | NA | NA | rs12432151 | 0.025959 | rs12432151 | 0.025959 |
| cg10309776 | 0 | 0 | 5 | 1318 | NA | NA | NA | NA | NA | NA |
| cg10374040 | 0 | 1319 | 3 | 1 | NA | NA | NA | NA | NA | NA |
| cg13073030 | 1 | 4 | 4 | 1315 | NA | NA | NA | NA | NA | NA |
| cg13153796 | 1 | 3 | 6 | 1314 | rs79645066 | 0.023562 | NA | NA | NA | NA |
| cg13504976 | 1 | 1 | 3 | 1319 | NA | NA | NA | NA | NA | NA |
| cg14290070 | 0 | 0 | 5 | 1318 | NA | NA | NA | NA | NA | NA |
| cg14454338 | 1 | 1 | 3 | 1319 | NA | NA | NA | NA | NA | NA |
| cg16551220 | 1 | 4 | 5 | 1314 | NA | NA | NA | NA | NA | NA |
| cg18229270 | 0 | 0 | 4 | 1319 | NA | NA | NA | NA | NA | NA |
| cg19511994 | 0.99 | 4 | 9 | 1310 | rs2164067 | 0 | NA | NA | NA | NA |
| cg19865727 | 1 | 1 | 3 | 1319 | NA | NA | NA | NA | NA | NA |
| cg19988673 | 0 | 0 | 4 | 1319 | rs143977546 | 0.014976 | NA | NA | NA | NA |
| cg20963002 | 0.97 | 2 | 73 | 1248 | NA | NA | rs76984454 | 0.031549 | rs76984454 | 0.031549 |
| cg22783747 | 1 | 2 | 4 | 1317 | NA | NA | NA | NA | NA | NA |
| cg26339162 | 1 | 2 | 3 | 1318 | NA | NA | NA | NA | NA | NA |
| cg27034225 | 0 | 0 | 5 | 1318 | NA | NA | NA | NA | NA | NA |
| cg27062397 | 0 | 0 | 5 | 1318 | NA | NA | NA | NA | NA | NA |
| cg27293090 | 0 | 0 | 3 | 1320 | NA | NA | NA | NA | NA | NA |
| cg00333583 | 1 | 1 | 5 | 1317 | NA | NA | NA | NA | NA | NA |
| cg01281649 | 1 | 1 | 2 | 1320 | NA | NA | NA | NA | NA | NA |
| cg04489066 | 1 | 1 | 1 | 1321 | rs1704396 | 0.455975 | NA | NA | NA | NA |
| cg05907398 | 0.99 | 1 | 13 | 1309 | NA | NA | NA | NA | NA | NA |
| cg08475996 | 1 | 2 | 6 | 1315 | NA | NA | NA | NA | NA | NA |
| cg12220058 | 0.99 | 13 | 2 | 1308 | NA | NA | NA | NA | NA | NA |
| cg12354961 | 1 | 3 | 3 | 1317 | NA | NA | NA | NA | NA | NA |
| cg13957124 | 1 | 3 | 3 | 1317 | NA | NA | NA | NA | NA | NA |
| cg15609631 | 0 | 0 | 4 | 1319 | NA | NA | NA | NA | NA | NA |
| cg16068495 | 1 | 1 | 4 | 1318 | NA | NA | NA | NA | NA | NA |
| cg16268380 | 0.01 | 1307 | 15 | 1 | NA | NA | NA | NA | NA | NA |
| cg16416584 | 0.95 | 8 | 105 | 1210 | NA | NA | rs2005180 | 0.060647 | rs2005180 | 0.060647 |
| cg16590643 | 0.97 | 1 | 70 | 1252 | NA | NA | rs61753871 | 0.044846 | rs61753871 | 0.044846 |
| cg16767040 | 1 | 1 | 3 | 1319 | NA | NA | NA | NA | NA | NA |
| cg19575909 | 1 | 1 | 5 | 1317 | NA | NA | NA | NA | NA | NA |
| cg20033079 | 1 | 1 | 1 | 1321 | NA | NA | NA | NA | NA | NA |
| cg20555018 | 1 | 3 | 1 | 1319 | NA | NA | NA | NA | NA | NA |
| cg20608709 | 1 | 3 | 3 | 1317 | NA | NA | NA | NA | NA | NA |
| cg21541477 | 0 | 0 | 5 | 1318 | NA | NA | NA | NA | NA | NA |
| cg21658515 | 0 | 0 | 7 | 1316 | NA | NA | rs62023449 | 0.067692 | NA | NA |
| cg22413635 | 0 | 0 | 5 | 1318 | NA | NA | NA | NA | NA | NA |
| cg22584911 | 1 | 1 | 5 | 1317 | NA | NA | NA | NA | NA | NA |
| cg24029551 | 1 | 1 | 3 | 1319 | NA | NA | NA | NA | NA | NA |
| cg24784129 | 1 | 2 | 4 | 1317 | NA | NA | NA | NA | NA | NA |
| cg24787593 | 0.98 | 25 | 3 | 1295 | NA | NA | rs72622090 | 0.084465 | NA | NA |
| cg25211412 | 1 | 4 | 4 | 1315 | NA | NA | NA | NA | NA | NA |
| cg26782261 | 1 | 1 | 10 | 1312 | NA | NA | NA | NA | NA | NA |
| cg26819633 | 1 | 3 | 2 | 1318 | NA | NA | NA | NA | NA | NA |
| cg27201332 | 0 | 0 | 6 | 1317 | NA | NA | NA | NA | NA | NA |
| cg00117311 | 0.98 | 1 | 48 | 1274 | NA | NA | rs78210031 | 0.014776 | rs78210031 | 0.014776 |
| cg00240860 | 1 | 1 | 5 | 1317 | NA | NA | NA | NA | NA | NA |
| cg01147079 | 0 | 0 | 4 | 1319 | rs77230647 | 0.030751 | NA | NA | NA | NA |
| cg02371802 | 0.98 | 1 | 43 | 1279 | rs34923865 | 0.012026 | NA | NA | NA | NA |
| cg02451516 | 0 | 0 | 3 | 1320 | NA | NA | NA | NA | NA | NA |
| cg02459859 | 0.99 | 1 | 30 | 1292 | NA | NA | rs35987447 | 0.017509 | rs35987447 | 0.017509 |
| cg02594607 | 0.99 | 3 | 8 | 1312 | NA | NA | NA | NA | NA | NA |
| cg02719221 | 1 | 1 | 11 | 1311 | NA | NA | NA | NA | NA | NA |
| cg03884018 | 1 | 2 | 2 | 1319 | NA | NA | NA | NA | NA | NA |
| cg04612030 | 0.93 | 10 | 172 | 1141 | NA | NA | rs35583599 | 0.080898 | rs35583599 | 0.080898 |
| cg04908106 | 1 | 1 | 6 | 1316 | NA | NA | NA | NA | NA | NA |
| cg05404009 | 1 | 1 | 3 | 1319 | NA | NA | rs57967030 | 0.018371 | NA | NA |
| cg06103394 | 0.08 | 1131 | 165 | 27 | rs4786364 | 0.380192 | NA | NA | NA | NA |
| cg06528228 | 1 | 1 | 5 | 1317 | NA | NA | NA | NA | NA | NA |
| cg07192961 | 0.99 | 1 | 35 | 1287 | NA | NA | rs145837399 | 0.014577 | rs145837399 | 0.014577 |
| cg07722333 | 1 | 1 | 4 | 1318 | NA | NA | NA | NA | NA | NA |
| cg07794169 | 0 | 0 | 3 | 1320 | NA | NA | NA | NA | NA | NA |
| cg07984980 | 0.99 | 2 | 15 | 1306 | NA | NA | NA | NA | NA | NA |
| cg08075631 | 1 | 1 | 2 | 1320 | NA | NA | NA | NA | NA | NA |
| cg08215675 | 1 | 1 | 4 | 1318 | NA | NA | NA | NA | NA | NA |
| cg26661402 | 1 | 5 | 2 | 1316 | NA | NA | rs2231708 | 2.60E-05 | NA | NA |
| cg26992524 | 0.99 | 1 | 35 | 1287 | NA | NA | NA | NA | NA | NA |
| cg27368709 | 0 | 0 | 9 | 1314 | rs74022021 | 0.031749 | NA | NA | NA | NA |
| cg02434736 | 0 | 0 | 27 | 1296 | NA | NA | NA | NA | NA | NA |
| cg02505689 | 0 | 1321 | 1 | 1 | NA | NA | NA | NA | NA | NA |
| cg02864638 | 1 | 2 | 5 | 1316 | NA | NA | NA | NA | NA | NA |
| cg02988795 | 1 | 2 | 6 | 1315 | NA | NA | NA | NA | NA | NA |
| cg03936229 | 0.97 | 4 | 71 | 1248 | NA | NA | rs79034241 | 0.024561 | rs79034241 | 0.024561 |
| cg05271336 | 1 | 4 | 3 | 1316 | NA | NA | NA | NA | NA | NA |
| cg05865769 | 1 | 1 | 6 | 1316 | NA | NA | NA | NA | NA | NA |
| cg06213598 | 1 | 3 | 6 | 1314 | NA | NA | NA | NA | NA | NA |
| cg06614423 | 0 | 1317 | 6 | 0 | NA | NA | NA | NA | NA | NA |
| cg06668724 | 0 | 0 | 2 | 1321 | NA | NA | NA | NA | NA | NA |
| cg07973125 | 0.9 | 14 | 238 | 1071 | NA | NA | rs72837315 | 0.104233 | rs72837315 | 0.104233 |
| cg08683615 | 0 | 0 | 2 | 1321 | NA | NA | NA | NA | NA | NA |
| cg09509528 | 0 | 1321 | 2 | 0 | NA | NA | NA | NA | NA | NA |
| cg10760240 | 0.94 | 9 | 146 | 1168 | NA | NA | rs11653434 | 0.08487 | rs11653434 | 0.08487 |
| cg12423398 | 0 | 1321 | 2 | 0 | rs2251564 | 0.106829 | NA | NA | NA | NA |
| cg13155712 | 0 | 0 | 2 | 1321 | NA | NA | rs73366242 | 0.01258 | NA | NA |
| cg13359054 | 0 | 1320 | 3 | 0 | NA | NA | NA | NA | NA | NA |
| cg13628937 | 1 | 2 | 4 | 1317 | NA | NA | NA | NA | NA | NA |
| cg13723217 | 0.05 | 1205 | 111 | 7 | NA | NA | rs60618945 | 0.040535 | rs60618945 | 0.040535 |
| cg14009013 | 1 | 1 | 3 | 1319 | NA | NA | NA | NA | NA | NA |
| cg14399851 | 0 | 1321 | 2 | 0 | NA | NA | NA | NA | NA | NA |
| cg15836138 | 1 | 1 | 1 | 1321 | NA | NA | rs74672530 | 0.026358 | NA | NA |
| cg17514485 | 0.99 | 1 | 18 | 1304 | NA | NA | rs56153053 | 0.036541 | NA | NA |
| cg18057692 | 0.98 | 1 | 53 | 1269 | NA | NA | rs116402533 | 0.015176 | rs116402533 | 0.015176 |
| cg18605237 | 1 | 3 | 3 | 1317 | NA | NA | rs114109966 | 0.028954 | NA | NA |
| cg19058765 | 1 | 1 | 9 | 1313 | NA | NA | NA | NA | NA | NA |
| cg22833065 | 0.94 | 1 | 157 | 1165 | NA | NA | NA | NA | NA | NA |
| cg23655623 | 1 | 1 | 2 | 1320 | NA | NA | NA | NA | NA | NA |
| cg26675906 | 1 | 1 | 6 | 1316 | NA | NA | NA | NA | NA | NA |
| cg27224372 | 0.54 | 1 | 1227 | 95 | NA | NA | rs115127871 | 0.023562 | rs115127871 | 0.023562 |
| cg02950608 | 1 | 1 | 2 | 1320 | NA | NA | NA | NA | NA | NA |
| cg03295626 | 1 | 3 | 4 | 1316 | rs4890862 | 0.047923 | NA | NA | NA | NA |
| cg09836531 | 0 | 1321 | 2 | 0 | NA | NA | NA | NA | NA | NA |
| cg10391823 | 0.99 | 1 | 19 | 1303 | rs112489150 | 0.011582 | NA | NA | NA | NA |
| cg15630599 | 1 | 1 | 4 | 1318 | NA | NA | NA | NA | NA | NA |
| cg16128296 | 0 | 0 | 6 | 1317 | rs2298722 | 0.403355 | NA | NA | NA | NA |
| cg18387085 | 0 | 1319 | 4 | 0 | NA | NA | NA | NA | NA | NA |
| cg22227168 | 1 | 1 | 3 | 1319 | NA | NA | NA | NA | NA | NA |
| cg22568695 | 1 | 4 | 4 | 1315 | NA | NA | NA | NA | NA | NA |
| cg23290664 | 0.98 | 1 | 63 | 1259 | NA | NA | rs34878160 | 0.029818 | rs34878160 | 0.029818 |
| cg23785882 | 0 | 0 | 2 | 1321 | NA | NA | NA | NA | NA | NA |
| cg01232138 | 0.99 | 1 | 31 | 1291 | NA | NA | NA | NA | NA | NA |
| cg01294521 | 1 | 1 | 10 | 1312 | rs71337061 | 0.033147 | NA | NA | NA | NA |
| cg03123424 | 1 | 1 | 5 | 1317 | NA | NA | rs3745238 | 8.00E-06 | NA | NA |
| cg03704806 | 1 | 1 | 10 | 1312 | NA | NA | NA | NA | NA | NA |
| cg08692328 | 0 | 0 | 10 | 1313 | NA | NA | NA | NA | NA | NA |
| cg09303582 | 0 | 0 | 4 | 1319 | NA | NA | NA | NA | NA | NA |
| cg13083111 | 0 | 1321 | 1 | 1 | NA | NA | NA | NA | NA | NA |
| cg13747174 | 1 | 4 | 3 | 1316 | NA | NA | NA | NA | NA | NA |
| cg18414741 | 0 | 1316 | 7 | 0 | NA | NA | rs56243325 | 0.036342 | NA | NA |
| cg18588930 | 0 | 0 | 6 | 1317 | NA | NA | NA | NA | NA | NA |
| cg21414902 | 0.94 | 17 | 119 | 1187 | NA | NA | rs187406577 | 0.000399 | rs187406577 | 0.000399 |
| cg22330055 | 0.99 | 11 | 2 | 1310 | NA | NA | rs76698783 | 0.052117 | NA | NA |
| cg22621913 | 0.99 | 1 | 20 | 1302 | NA | NA | rs140442783 | 0.01258 | rs140442783 | 0.01258 |
| cg23393214 | 1 | 1 | 3 | 1319 | rs8112112 | 0.186302 | NA | NA | NA | NA |
| cg25371549 | 1 | 2 | 5 | 1316 | NA | NA | NA | NA | NA | NA |
| cg25693289 | 0 | 1321 | 2 | 0 | NA | NA | NA | NA | NA | NA |
| cg26777040 | 0.98 | 2 | 60 | 1261 | NA | NA | NA | NA | NA | NA |
| cg27384074 | 1 | 1 | 3 | 1319 | NA | NA | NA | NA | NA | NA |
| cg00414709 | 1 | 2 | 6 | 1315 | NA | NA | NA | NA | NA | NA |
| cg03223734 | 1 | 1 | 7 | 1315 | NA | NA | NA | NA | NA | NA |
| cg03734225 | 0.97 | 2 | 65 | 1256 | NA | NA | NA | NA | NA | NA |
| cg04772817 | 0 | 0 | 3 | 1320 | rs76442957 | 0.025959 | NA | NA | NA | NA |
| cg08231038 | 0 | 0 | 6 | 1317 | NA | NA | NA | NA | NA | NA |
| cg10748765 | 0 | 1321 | 1 | 1 | NA | NA | NA | NA | NA | NA |
| cg19201723 | 1 | 1 | 6 | 1316 | NA | NA | NA | NA | NA | NA |
| cg21492942 | 0 | 1320 | 3 | 0 | NA | NA | NA | NA | NA | NA |
| cg22060087 | 1 | 3 | 4 | 1316 | NA | NA | NA | NA | NA | NA |
| cg22618509 | 1 | 1 | 5 | 1317 | rs79940214 | 0.013339 | NA | NA | NA | NA |
| cg24615945 | 1 | 2 | 5 | 1316 | NA | NA | NA | NA | NA | NA |
| cg26032363 | 0 | 0 | 3 | 1320 | NA | NA | NA | NA | NA | NA |
| cg26082838 | 0 | 1318 | 5 | 0 | NA | NA | NA | NA | NA | NA |
| cg26843176 | 0.99 | 5 | 5 | 1313 | NA | NA | NA | NA | NA | NA |
| cg07612468 | 1 | 2 | 4 | 1317 | NA | NA | NA | NA | NA | NA |
| cg12054554 | 0 | 1318 | 5 | 0 | rs13051949 | 0.300719 | NA | NA | NA | NA |
| cg16140273 | 0.99 | 2 | 10 | 1311 | NA | NA | NA | NA | NA | NA |
| cg21274607 | 0 | 0 | 7 | 1316 | NA | NA | NA | NA | NA | NA |
| cg00488512 | 0 | 1317 | 5 | 1 | NA | NA | NA | NA | NA | NA |
| cg06432843 | 0.98 | 3 | 41 | 1279 | NA | NA | rs114068801 | 0.011582 | rs114068801 | 0.011582 |
| cg14412792 | 1 | 1 | 7 | 1315 | NA | NA | rs117000644 | 0.072883 | NA | NA |
| cg18132256 | 0 | 0 | 3 | 1320 | NA | NA | NA | NA | NA | NA |
| cg19800640 | 0.91 | 23 | 187 | 1113 | NA | NA | rs6520149 | 0.089657 | rs6520149 | 0.089657 |
| cg20124409 | 0 | 0 | 12 | 1311 | NA | NA | NA | NA | NA | NA |
| cg22089929 | 1 | 1 | 5 | 1317 | NA | NA | rs149932317 | 0.01218 | NA | NA |
