# Supplementary material for: Gaps in current methods to detect polymorphic CpGs from Illumina Infinium human methylation microarrays and exploring their potential impact in multi-EWAS analyses
Source: Epigenetics. 2023 Nov 20;18(1):2281153. doi: 10.1080/15592294.2023.2281153 (PMC10732615; doi:10.1080/15592294.2023.2281153)
Supplement: Supplementary_File_2.docx [file KEPI_A_2281153_SM9089.docx]

| Sites | Groups | Group1 | Group2 | Group3 | Group4 | Group5 | Group6 | Group7 | Group8 | Group9 | Group10 | Group11 |
| --- | --- | --- | --- | --- | --- | --- | --- | --- | --- | --- | --- | --- |
| cg04448376 | 2 | 1253 | 70 | 0 | 0 | 0 | 0 | 0 | 0 | 0 | 0 | 0 |
| cg00011200 | 3 | 615 | 2 | 706 | 0 | 0 | 0 | 0 | 0 | 0 | 0 | 0 |
| cg00011891 | 2 | 611 | 712 | 0 | 0 | 0 | 0 | 0 | 0 | 0 | 0 | 0 |
| cg00016522 | 2 | 619 | 704 | 0 | 0 | 0 | 0 | 0 | 0 | 0 | 0 | 0 |
| cg00018261 | 2 | 616 | 707 | 0 | 0 | 0 | 0 | 0 | 0 | 0 | 0 | 0 |
| cg00026186 | 4 | 618 | 1 | 1 | 703 | 0 | 0 | 0 | 0 | 0 | 0 | 0 |
| cg00112256 | 2 | 608 | 715 | 0 | 0 | 0 | 0 | 0 | 0 | 0 | 0 | 0 |
| cg00114913 | 3 | 616 | 706 | 1 | 0 | 0 | 0 | 0 | 0 | 0 | 0 | 0 |
| cg00116709 | 6 | 609 | 3 | 2 | 707 | 1 | 1 | 0 | 0 | 0 | 0 | 0 |
| cg00139317 | 2 | 617 | 706 | 0 | 0 | 0 | 0 | 0 | 0 | 0 | 0 | 0 |
| cg00140085 | 3 | 617 | 705 | 1 | 0 | 0 | 0 | 0 | 0 | 0 | 0 | 0 |
| cg00192980 | 2 | 615 | 708 | 0 | 0 | 0 | 0 | 0 | 0 | 0 | 0 | 0 |
| cg00265812 | 3 | 614 | 1 | 708 | 0 | 0 | 0 | 0 | 0 | 0 | 0 | 0 |
| cg00380985 | 2 | 619 | 704 | 0 | 0 | 0 | 0 | 0 | 0 | 0 | 0 | 0 |
| cg00399450 | 4 | 609 | 8 | 705 | 1 | 0 | 0 | 0 | 0 | 0 | 0 | 0 |
| cg00466309 | 3 | 614 | 2 | 707 | 0 | 0 | 0 | 0 | 0 | 0 | 0 | 0 |
| cg00473354 | 2 | 618 | 705 | 0 | 0 | 0 | 0 | 0 | 0 | 0 | 0 | 0 |
| cg00478198 | 3 | 617 | 1 | 705 | 0 | 0 | 0 | 0 | 0 | 0 | 0 | 0 |
| cg00575851 | 4 | 611 | 5 | 1 | 706 | 0 | 0 | 0 | 0 | 0 | 0 | 0 |
| cg00583618 | 5 | 608 | 6 | 1 | 707 | 1 | 0 | 0 | 0 | 0 | 0 | 0 |
| cg00645049 | 2 | 617 | 706 | 0 | 0 | 0 | 0 | 0 | 0 | 0 | 0 | 0 |
| cg00666173 | 3 | 608 | 11 | 704 | 0 | 0 | 0 | 0 | 0 | 0 | 0 | 0 |
| cg00680673 | 3 | 610 | 8 | 705 | 0 | 0 | 0 | 0 | 0 | 0 | 0 | 0 |
| cg00685925 | 2 | 615 | 708 | 0 | 0 | 0 | 0 | 0 | 0 | 0 | 0 | 0 |
| cg00729461 | 3 | 616 | 2 | 705 | 0 | 0 | 0 | 0 | 0 | 0 | 0 | 0 |
| cg00739467 | 2 | 610 | 713 | 0 | 0 | 0 | 0 | 0 | 0 | 0 | 0 | 0 |
| cg00769799 | 3 | 614 | 708 | 1 | 0 | 0 | 0 | 0 | 0 | 0 | 0 | 0 |
| cg00810519 | 5 | 608 | 7 | 2 | 2 | 704 | 0 | 0 | 0 | 0 | 0 | 0 |
| cg00813156 | 3 | 615 | 707 | 1 | 0 | 0 | 0 | 0 | 0 | 0 | 0 | 0 |
| cg00832270 | 3 | 609 | 5 | 709 | 0 | 0 | 0 | 0 | 0 | 0 | 0 | 0 |
| cg00845806 | 3 | 604 | 686 | 33 | 0 | 0 | 0 | 0 | 0 | 0 | 0 | 0 |
| cg00904184 | 3 | 617 | 705 | 1 | 0 | 0 | 0 | 0 | 0 | 0 | 0 | 0 |
| cg00988678 | 3 | 616 | 2 | 705 | 0 | 0 | 0 | 0 | 0 | 0 | 0 | 0 |
| cg00993140 | 3 | 615 | 707 | 1 | 0 | 0 | 0 | 0 | 0 | 0 | 0 | 0 |
| cg00996177 | 3 | 615 | 1 | 707 | 0 | 0 | 0 | 0 | 0 | 0 | 0 | 0 |
| cg01000789 | 3 | 608 | 8 | 707 | 0 | 0 | 0 | 0 | 0 | 0 | 0 | 0 |
| cg01056373 | 2 | 615 | 708 | 0 | 0 | 0 | 0 | 0 | 0 | 0 | 0 | 0 |
| cg01062269 | 2 | 617 | 706 | 0 | 0 | 0 | 0 | 0 | 0 | 0 | 0 | 0 |
| cg01120894 | 3 | 614 | 2 | 707 | 0 | 0 | 0 | 0 | 0 | 0 | 0 | 0 |
| cg01191902 | 2 | 615 | 708 | 0 | 0 | 0 | 0 | 0 | 0 | 0 | 0 | 0 |
| cg01215118 | 4 | 613 | 1 | 708 | 1 | 0 | 0 | 0 | 0 | 0 | 0 | 0 |
| cg01303569 | 2 | 616 | 707 | 0 | 0 | 0 | 0 | 0 | 0 | 0 | 0 | 0 |
| cg01318188 | 3 | 609 | 10 | 704 | 0 | 0 | 0 | 0 | 0 | 0 | 0 | 0 |
| cg01364818 | 3 | 619 | 702 | 2 | 0 | 0 | 0 | 0 | 0 | 0 | 0 | 0 |
| cg01366378 | 2 | 615 | 708 | 0 | 0 | 0 | 0 | 0 | 0 | 0 | 0 | 0 |
| cg01370077 | 5 | 610 | 6 | 1 | 1 | 705 | 0 | 0 | 0 | 0 | 0 | 0 |
| cg01374431 | 2 | 616 | 707 | 0 | 0 | 0 | 0 | 0 | 0 | 0 | 0 | 0 |
| cg01405303 | 4 | 613 | 3 | 706 | 1 | 0 | 0 | 0 | 0 | 0 | 0 | 0 |
| cg01483252 | 2 | 614 | 709 | 0 | 0 | 0 | 0 | 0 | 0 | 0 | 0 | 0 |
| cg01600123 | 3 | 614 | 708 | 1 | 0 | 0 | 0 | 0 | 0 | 0 | 0 | 0 |
| cg01668271 | 3 | 613 | 4 | 706 | 0 | 0 | 0 | 0 | 0 | 0 | 0 | 0 |
| cg01671070 | 3 | 611 | 3 | 709 | 0 | 0 | 0 | 0 | 0 | 0 | 0 | 0 |
| cg01742836 | 6 | 605 | 2 | 1 | 7 | 1 | 707 | 0 | 0 | 0 | 0 | 0 |
| cg01798375 | 2 | 614 | 709 | 0 | 0 | 0 | 0 | 0 | 0 | 0 | 0 | 0 |
| cg01804836 | 2 | 616 | 707 | 0 | 0 | 0 | 0 | 0 | 0 | 0 | 0 | 0 |
| cg01807728 | 2 | 616 | 707 | 0 | 0 | 0 | 0 | 0 | 0 | 0 | 0 | 0 |
| cg01813294 | 2 | 615 | 708 | 0 | 0 | 0 | 0 | 0 | 0 | 0 | 0 | 0 |
| cg01825872 | 4 | 615 | 1 | 706 | 1 | 0 | 0 | 0 | 0 | 0 | 0 | 0 |
| cg01869765 | 4 | 612 | 5 | 1 | 705 | 0 | 0 | 0 | 0 | 0 | 0 | 0 |
| cg01873087 | 4 | 617 | 1 | 704 | 1 | 0 | 0 | 0 | 0 | 0 | 0 | 0 |
| cg01991530 | 2 | 616 | 707 | 0 | 0 | 0 | 0 | 0 | 0 | 0 | 0 | 0 |
| cg02004156 | 2 | 614 | 709 | 0 | 0 | 0 | 0 | 0 | 0 | 0 | 0 | 0 |
| cg02049865 | 3 | 614 | 708 | 1 | 0 | 0 | 0 | 0 | 0 | 0 | 0 | 0 |
| cg02156782 | 2 | 614 | 709 | 0 | 0 | 0 | 0 | 0 | 0 | 0 | 0 | 0 |
| cg02161919 | 2 | 619 | 704 | 0 | 0 | 0 | 0 | 0 | 0 | 0 | 0 | 0 |
| cg02195366 | 3 | 606 | 9 | 708 | 0 | 0 | 0 | 0 | 0 | 0 | 0 | 0 |
| cg02201836 | 2 | 617 | 706 | 0 | 0 | 0 | 0 | 0 | 0 | 0 | 0 | 0 |
| cg02274705 | 2 | 618 | 705 | 0 | 0 | 0 | 0 | 0 | 0 | 0 | 0 | 0 |
| cg02276461 | 2 | 618 | 705 | 0 | 0 | 0 | 0 | 0 | 0 | 0 | 0 | 0 |
| cg02283535 | 2 | 617 | 706 | 0 | 0 | 0 | 0 | 0 | 0 | 0 | 0 | 0 |
| cg02288667 | 2 | 614 | 709 | 0 | 0 | 0 | 0 | 0 | 0 | 0 | 0 | 0 |
| cg02291897 | 2 | 617 | 706 | 0 | 0 | 0 | 0 | 0 | 0 | 0 | 0 | 0 |
| cg02295504 | 2 | 617 | 706 | 0 | 0 | 0 | 0 | 0 | 0 | 0 | 0 | 0 |
| cg02330362 | 3 | 616 | 705 | 2 | 0 | 0 | 0 | 0 | 0 | 0 | 0 | 0 |
| cg02333283 | 2 | 610 | 713 | 0 | 0 | 0 | 0 | 0 | 0 | 0 | 0 | 0 |
| cg02337670 | 2 | 615 | 708 | 0 | 0 | 0 | 0 | 0 | 0 | 0 | 0 | 0 |
| cg02360578 | 2 | 616 | 707 | 0 | 0 | 0 | 0 | 0 | 0 | 0 | 0 | 0 |
| cg02386310 | 3 | 618 | 704 | 1 | 0 | 0 | 0 | 0 | 0 | 0 | 0 | 0 |
| cg02480419 | 2 | 616 | 707 | 0 | 0 | 0 | 0 | 0 | 0 | 0 | 0 | 0 |
| cg02496423 | 4 | 611 | 7 | 703 | 2 | 0 | 0 | 0 | 0 | 0 | 0 | 0 |
| cg02510708 | 3 | 616 | 1 | 706 | 0 | 0 | 0 | 0 | 0 | 0 | 0 | 0 |
| cg02533866 | 2 | 617 | 706 | 0 | 0 | 0 | 0 | 0 | 0 | 0 | 0 | 0 |
| cg02539591 | 2 | 615 | 708 | 0 | 0 | 0 | 0 | 0 | 0 | 0 | 0 | 0 |
| cg02615131 | 3 | 610 | 7 | 706 | 0 | 0 | 0 | 0 | 0 | 0 | 0 | 0 |
| cg02621658 | 3 | 617 | 1 | 705 | 0 | 0 | 0 | 0 | 0 | 0 | 0 | 0 |
| cg02634083 | 4 | 610 | 2 | 709 | 2 | 0 | 0 | 0 | 0 | 0 | 0 | 0 |
| cg02770249 | 3 | 614 | 1 | 708 | 0 | 0 | 0 | 0 | 0 | 0 | 0 | 0 |
| cg02772106 | 2 | 616 | 707 | 0 | 0 | 0 | 0 | 0 | 0 | 0 | 0 | 0 |
| cg02775175 | 3 | 611 | 5 | 707 | 0 | 0 | 0 | 0 | 0 | 0 | 0 | 0 |
| cg02869694 | 2 | 616 | 707 | 0 | 0 | 0 | 0 | 0 | 0 | 0 | 0 | 0 |
| cg02921434 | 4 | 610 | 4 | 1 | 708 | 0 | 0 | 0 | 0 | 0 | 0 | 0 |
| cg02937293 | 2 | 619 | 704 | 0 | 0 | 0 | 0 | 0 | 0 | 0 | 0 | 0 |
| cg02971902 | 3 | 617 | 705 | 1 | 0 | 0 | 0 | 0 | 0 | 0 | 0 | 0 |
| cg02994943 | 3 | 615 | 1 | 707 | 0 | 0 | 0 | 0 | 0 | 0 | 0 | 0 |
| cg03031357 | 3 | 615 | 707 | 1 | 0 | 0 | 0 | 0 | 0 | 0 | 0 | 0 |
| cg03219705 | 4 | 612 | 709 | 1 | 1 | 0 | 0 | 0 | 0 | 0 | 0 | 0 |
| cg03272642 | 2 | 608 | 715 | 0 | 0 | 0 | 0 | 0 | 0 | 0 | 0 | 0 |
| cg03323437 | 3 | 616 | 1 | 706 | 0 | 0 | 0 | 0 | 0 | 0 | 0 | 0 |
| cg03334316 | 2 | 613 | 710 | 0 | 0 | 0 | 0 | 0 | 0 | 0 | 0 | 0 |
| cg03335173 | 2 | 619 | 704 | 0 | 0 | 0 | 0 | 0 | 0 | 0 | 0 | 0 |
| cg03364121 | 2 | 616 | 707 | 0 | 0 | 0 | 0 | 0 | 0 | 0 | 0 | 0 |
| cg03391801 | 4 | 612 | 4 | 706 | 1 | 0 | 0 | 0 | 0 | 0 | 0 | 0 |
| cg03505772 | 2 | 614 | 709 | 0 | 0 | 0 | 0 | 0 | 0 | 0 | 0 | 0 |
| cg03513471 | 2 | 614 | 709 | 0 | 0 | 0 | 0 | 0 | 0 | 0 | 0 | 0 |
| cg03590418 | 2 | 618 | 705 | 0 | 0 | 0 | 0 | 0 | 0 | 0 | 0 | 0 |
| cg03605032 | 2 | 616 | 707 | 0 | 0 | 0 | 0 | 0 | 0 | 0 | 0 | 0 |
| cg03670113 | 3 | 610 | 7 | 706 | 0 | 0 | 0 | 0 | 0 | 0 | 0 | 0 |
| cg03691313 | 2 | 609 | 714 | 0 | 0 | 0 | 0 | 0 | 0 | 0 | 0 | 0 |
| cg03705894 | 2 | 617 | 706 | 0 | 0 | 0 | 0 | 0 | 0 | 0 | 0 | 0 |
| cg03730082 | 2 | 618 | 705 | 0 | 0 | 0 | 0 | 0 | 0 | 0 | 0 | 0 |
| cg03748372 | 2 | 614 | 709 | 0 | 0 | 0 | 0 | 0 | 0 | 0 | 0 | 0 |
| cg03751162 | 2 | 615 | 708 | 0 | 0 | 0 | 0 | 0 | 0 | 0 | 0 | 0 |
| cg03759948 | 3 | 614 | 708 | 1 | 0 | 0 | 0 | 0 | 0 | 0 | 0 | 0 |
| cg03769817 | 4 | 1 | 618 | 703 | 1 | 0 | 0 | 0 | 0 | 0 | 0 | 0 |
| cg03831206 | 2 | 614 | 709 | 0 | 0 | 0 | 0 | 0 | 0 | 0 | 0 | 0 |
| cg03900860 | 2 | 616 | 707 | 0 | 0 | 0 | 0 | 0 | 0 | 0 | 0 | 0 |
| cg03905487 | 3 | 617 | 2 | 704 | 0 | 0 | 0 | 0 | 0 | 0 | 0 | 0 |
| cg03906572 | 2 | 609 | 714 | 0 | 0 | 0 | 0 | 0 | 0 | 0 | 0 | 0 |
| cg03944921 | 2 | 614 | 709 | 0 | 0 | 0 | 0 | 0 | 0 | 0 | 0 | 0 |
| cg03989304 | 3 | 614 | 707 | 2 | 0 | 0 | 0 | 0 | 0 | 0 | 0 | 0 |
| cg04026379 | 2 | 617 | 706 | 0 | 0 | 0 | 0 | 0 | 0 | 0 | 0 | 0 |
| cg04027004 | 2 | 615 | 708 | 0 | 0 | 0 | 0 | 0 | 0 | 0 | 0 | 0 |
| cg04027312 | 2 | 610 | 713 | 0 | 0 | 0 | 0 | 0 | 0 | 0 | 0 | 0 |
| cg04029664 | 2 | 614 | 709 | 0 | 0 | 0 | 0 | 0 | 0 | 0 | 0 | 0 |
| cg04061482 | 3 | 618 | 704 | 1 | 0 | 0 | 0 | 0 | 0 | 0 | 0 | 0 |
| cg04075675 | 2 | 617 | 706 | 0 | 0 | 0 | 0 | 0 | 0 | 0 | 0 | 0 |
| cg04149024 | 2 | 618 | 705 | 0 | 0 | 0 | 0 | 0 | 0 | 0 | 0 | 0 |
| cg04225046 | 2 | 614 | 709 | 0 | 0 | 0 | 0 | 0 | 0 | 0 | 0 | 0 |
| cg04288012 | 2 | 616 | 707 | 0 | 0 | 0 | 0 | 0 | 0 | 0 | 0 | 0 |
| cg04292836 | 2 | 617 | 706 | 0 | 0 | 0 | 0 | 0 | 0 | 0 | 0 | 0 |
| cg04302178 | 2 | 616 | 707 | 0 | 0 | 0 | 0 | 0 | 0 | 0 | 0 | 0 |
| cg04317640 | 4 | 611 | 4 | 1 | 707 | 0 | 0 | 0 | 0 | 0 | 0 | 0 |
| cg04327763 | 2 | 618 | 705 | 0 | 0 | 0 | 0 | 0 | 0 | 0 | 0 | 0 |
| cg04345928 | 3 | 614 | 3 | 706 | 0 | 0 | 0 | 0 | 0 | 0 | 0 | 0 |
| cg04371001 | 2 | 616 | 707 | 0 | 0 | 0 | 0 | 0 | 0 | 0 | 0 | 0 |
| cg04376185 | 5 | 617 | 3 | 701 | 1 | 1 | 0 | 0 | 0 | 0 | 0 | 0 |
| cg04388383 | 2 | 616 | 707 | 0 | 0 | 0 | 0 | 0 | 0 | 0 | 0 | 0 |
| cg04419618 | 3 | 616 | 2 | 705 | 0 | 0 | 0 | 0 | 0 | 0 | 0 | 0 |
| cg04524851 | 3 | 607 | 4 | 712 | 0 | 0 | 0 | 0 | 0 | 0 | 0 | 0 |
| cg04552106 | 2 | 615 | 708 | 0 | 0 | 0 | 0 | 0 | 0 | 0 | 0 | 0 |
| cg04595393 | 2 | 617 | 706 | 0 | 0 | 0 | 0 | 0 | 0 | 0 | 0 | 0 |
| cg04626171 | 4 | 614 | 707 | 1 | 1 | 0 | 0 | 0 | 0 | 0 | 0 | 0 |
| cg04650971 | 2 | 616 | 707 | 0 | 0 | 0 | 0 | 0 | 0 | 0 | 0 | 0 |
| cg04665139 | 3 | 614 | 708 | 1 | 0 | 0 | 0 | 0 | 0 | 0 | 0 | 0 |
| cg04675919 | 2 | 616 | 707 | 0 | 0 | 0 | 0 | 0 | 0 | 0 | 0 | 0 |
| cg04691867 | 2 | 617 | 706 | 0 | 0 | 0 | 0 | 0 | 0 | 0 | 0 | 0 |
| cg04712194 | 2 | 614 | 709 | 0 | 0 | 0 | 0 | 0 | 0 | 0 | 0 | 0 |
| cg04836978 | 2 | 617 | 706 | 0 | 0 | 0 | 0 | 0 | 0 | 0 | 0 | 0 |
| cg04855977 | 3 | 614 | 708 | 1 | 0 | 0 | 0 | 0.00E+00 | 0 | 0 | 0 | 0 |
| cg05045028 | 2 | 616 | 707 | 0 | 0 | 0 | 0 | 0 | 0 | 0 | 0 | 0 |
| cg05049545 | 4 | 613 | 4 | 704 | 2 | 0 | 0 | 0 | 0 | 0 | 0 | 0 |
| cg05059648 | 2 | 613 | 710 | 0 | 0 | 0 | 0 | 0 | 0 | 0 | 0 | 0 |
| cg05088151 | 2 | 612 | 711 | 0 | 0 | 0 | 0 | 0 | 0 | 0 | 0 | 0 |
| cg05091873 | 2 | 616 | 707 | 0 | 0 | 0 | 0 | 0 | 0 | 0 | 0 | 0 |
| cg05100261 | 2 | 618 | 705 | 0 | 0 | 0 | 0 | 0 | 0 | 0 | 0 | 0 |
| cg05109619 | 3 | 610 | 7 | 706 | 0 | 0 | 0 | 0 | 0 | 0 | 0 | 0 |
| cg05200254 | 3 | 615 | 706 | 2 | 0 | 0 | 0 | 0 | 0 | 0 | 0 | 0 |
| cg05260852 | 4 | 614 | 3 | 705 | 1 | 0 | 0 | 0 | 0 | 0 | 0 | 0 |
| cg05374090 | 2 | 610 | 713 | 0 | 0 | 0 | 0 | 0 | 0 | 0 | 0 | 0 |
| cg05397816 | 3 | 609 | 7 | 707 | 0 | 0 | 0 | 0 | 0 | 0 | 0 | 0 |
| cg05438092 | 3 | 608 | 7 | 708 | 0 | 0 | 0 | 0 | 0 | 0 | 0 | 0 |
| cg05452887 | 2 | 615 | 708 | 0 | 0 | 0 | 0 | 0 | 0 | 0 | 0 | 0 |
| cg05461361 | 2 | 617 | 706 | 0 | 0 | 0 | 0 | 0 | 0 | 0 | 0 | 0 |
| cg05476089 | 3 | 615 | 1 | 707 | 0 | 0 | 0 | 0 | 0 | 0 | 0 | 0 |
| cg05526804 | 3 | 615 | 707 | 1 | 0 | 0 | 0 | 0 | 0 | 0 | 0 | 0 |
| cg05534333 | 3 | 608 | 4 | 711 | 0 | 0 | 0 | 0 | 0 | 0 | 0 | 0 |
| cg05785344 | 3 | 613 | 709 | 1 | 0 | 0 | 0 | 0 | 0 | 0 | 0 | 0 |
| cg05788681 | 3 | 616 | 706 | 1 | 0 | 0 | 0 | 0 | 0 | 0 | 0 | 0 |
| cg05803370 | 2 | 617 | 706 | 0 | 0 | 0 | 0 | 0 | 0 | 0 | 0 | 0 |
| cg05806018 | 3 | 618 | 704 | 1 | 0 | 0 | 0 | 0 | 0 | 0 | 0 | 0 |
| cg05835545 | 3 | 615 | 1 | 707 | 0 | 0 | 0 | 0 | 0 | 0 | 0 | 0 |
| cg05893072 | 2 | 622 | 701 | 0 | 0 | 0 | 0 | 0 | 0 | 0 | 0 | 0 |
| cg05899999 | 2 | 615 | 708 | 0 | 0 | 0 | 0 | 0 | 0 | 0 | 0 | 0 |
| cg05907136 | 2 | 616 | 707 | 0 | 0 | 0 | 0 | 0 | 0 | 0 | 0 | 0 |
| cg05908188 | 2 | 618 | 705 | 0 | 0 | 0 | 0 | 0 | 0 | 0 | 0 | 0 |
| cg05935584 | 3 | 604 | 12 | 707 | 0 | 0 | 0 | 0 | 0 | 0 | 0 | 0 |
| cg05996419 | 2 | 608 | 715 | 0 | 0 | 0 | 0 | 0 | 0 | 0 | 0 | 0 |
| cg06027835 | 2 | 615 | 708 | 0 | 0 | 0 | 0 | 0 | 0 | 0 | 0 | 0 |
| cg06041068 | 2 | 616 | 707 | 0 | 0 | 0 | 0 | 0 | 0 | 0 | 0 | 0 |
| cg06042004 | 3 | 613 | 3 | 707 | 0 | 0 | 0 | 0 | 0 | 0 | 0 | 0 |
| cg06051391 | 3 | 610 | 711 | 2 | 0 | 0 | 0 | 0 | 0 | 0 | 0 | 0 |
| cg06055478 | 3 | 616 | 2 | 705 | 0 | 0 | 0 | 0 | 0 | 0 | 0 | 0 |
| cg06072560 | 3 | 618 | 704 | 1 | 0 | 0 | 0 | 0 | 0 | 0 | 0 | 0 |
| cg06079963 | 2 | 617 | 706 | 0 | 0 | 0 | 0 | 0 | 0 | 0 | 0 | 0 |
| cg06097615 | 2 | 616 | 707 | 0 | 0 | 0 | 0 | 0 | 0 | 0 | 0 | 0 |
| cg06098232 | 2 | 616 | 707 | 0 | 0 | 0 | 0 | 0 | 0 | 0 | 0 | 0 |
| cg06136002 | 3 | 614 | 2 | 707 | 0 | 0 | 0 | 0 | 0 | 0 | 0 | 0 |
| cg06139288 | 3 | 617 | 705 | 1 | 0 | 0 | 0 | 0 | 0 | 0 | 0 | 0 |
| cg06144999 | 3 | 615 | 707 | 1 | 0 | 0 | 0 | 0 | 0 | 0 | 0 | 0 |
| cg06164717 | 2 | 614 | 709 | 0 | 0 | 0 | 0 | 0 | 0 | 0 | 0 | 0 |
| cg06252876 | 4 | 616 | 704 | 1 | 2 | 0 | 0 | 0 | 0 | 0 | 0 | 0 |
| cg06266461 | 2 | 617 | 706 | 0 | 0 | 0 | 0 | 0 | 0 | 0 | 0 | 0 |
| cg06295352 | 2 | 616 | 707 | 0 | 0 | 0 | 0 | 0 | 0 | 0 | 0 | 0 |
| cg06297686 | 3 | 615 | 1 | 707 | 0 | 0 | 0 | 0 | 0 | 0 | 0 | 0 |
| cg06408025 | 2 | 615 | 708 | 0 | 0 | 0 | 0 | 0 | 0 | 0 | 0 | 0 |
| cg06506080 | 3 | 615 | 707 | 1 | 0 | 0 | 0 | 0 | 0 | 0 | 0 | 0 |
| cg06510592 | 3 | 613 | 3 | 707 | 0 | 0 | 0 | 0 | 0 | 0 | 0 | 0 |
| cg06535161 | 2 | 614 | 709 | 0 | 0 | 0 | 0 | 0 | 0 | 0 | 0 | 0 |
| cg06538336 | 2 | 615 | 708 | 0 | 0 | 0 | 0 | 0 | 0 | 0 | 0 | 0 |
| cg06558166 | 3 | 614 | 1 | 708 | 0 | 0 | 0 | 0 | 0 | 0 | 0 | 0 |
| cg06615444 | 2 | 610 | 713 | 0 | 0 | 0 | 0 | 0 | 0 | 0 | 0 | 0 |
| cg06616857 | 3 | 611 | 4 | 708 | 0 | 0 | 0 | 0 | 0 | 0 | 0 | 0 |
| cg06624970 | 2 | 617 | 706 | 0 | 0 | 0 | 0 | 0 | 0 | 0 | 0 | 0 |
| cg06673178 | 2 | 616 | 707 | 0 | 0 | 0 | 0 | 0 | 0 | 0 | 0 | 0 |
| cg06721573 | 2 | 616 | 707 | 0 | 0 | 0 | 0 | 0 | 0 | 0 | 0 | 0 |
| cg06779802 | 2 | 617 | 706 | 0 | 0 | 0 | 0 | 0 | 0 | 0 | 0 | 0 |
| cg06783548 | 3 | 614 | 1 | 708 | 0 | 0 | 0 | 0 | 0 | 0 | 0 | 0 |
| cg06805513 | 4 | 614 | 3 | 705 | 1 | 0 | 0 | 0 | 0 | 0 | 0 | 0 |
| cg06834235 | 3 | 620 | 701 | 2 | 0 | 0 | 0 | 0 | 0 | 0 | 0 | 0 |
| cg06915321 | 2 | 615 | 708 | 0 | 0 | 0 | 0 | 0 | 0 | 0 | 0 | 0 |
| cg06945800 | 2 | 615 | 708 | 0 | 0 | 0 | 0 | 0 | 0 | 0 | 0 | 0 |
| cg07012573 | 3 | 614 | 1 | 708 | 0 | 0 | 0 | 0 | 0 | 0 | 0 | 0 |
| cg07038409 | 2 | 616 | 707 | 0 | 0 | 0 | 0 | 0 | 0 | 0 | 0 | 0 |
| cg07056506 | 2 | 615 | 708 | 0 | 0 | 0 | 0 | 0 | 0 | 0 | 0 | 0 |
| cg07089242 | 3 | 616 | 705 | 2 | 0 | 0 | 0 | 0 | 0 | 0 | 0 | 0 |
| cg07136872 | 2 | 617 | 706 | 0 | 0 | 0 | 0 | 0 | 0 | 0 | 0 | 0 |
| cg07187289 | 3 | 606 | 9 | 708 | 0 | 0 | 0 | 0 | 0 | 0 | 0 | 0 |
| cg07215528 | 3 | 614 | 1 | 708 | 0 | 0 | 0 | 0 | 0 | 0 | 0 | 0 |
| cg07312966 | 3 | 616 | 705 | 2 | 0 | 0 | 0 | 0 | 0 | 0 | 0 | 0 |
| cg07363416 | 3 | 615 | 705 | 3 | 0 | 0 | 0 | 0 | 0 | 0 | 0 | 0 |
| cg07393670 | 2 | 616 | 707 | 0 | 0 | 0 | 0 | 0 | 0 | 0 | 0 | 0 |
| cg07419801 | 4 | 611 | 4 | 1 | 707 | 0 | 0 | 0 | 0 | 0 | 0 | 0 |
| cg07446674 | 3 | 609 | 3 | 711 | 0 | 0 | 0 | 0 | 0 | 0 | 0 | 0 |
| cg07507776 | 4 | 615 | 3 | 704 | 1 | 0 | 0 | 0 | 0 | 0 | 0 | 0 |
| cg07516457 | 2 | 615 | 708 | 0 | 0 | 0 | 0 | 0 | 0 | 0 | 0 | 0 |
| cg07648454 | 2 | 618 | 705 | 0 | 0 | 0 | 0 | 0 | 0 | 0 | 0 | 0 |
| cg07674075 | 4 | 614 | 3 | 705 | 1 | 0 | 0 | 0 | 0 | 0 | 0 | 0 |
| cg07674503 | 4 | 612 | 5 | 705 | 1 | 0 | 0 | 0 | 0 | 0 | 0 | 0 |
| cg07682072 | 3 | 615 | 1 | 707 | 0 | 0 | 0 | 0 | 0 | 0 | 0 | 0 |
| cg07750402 | 3 | 614 | 1 | 708 | 0 | 0 | 0 | 0 | 0 | 0 | 0 | 0 |
| cg07758529 | 5 | 612 | 2 | 707 | 1 | 1 | 0 | 0 | 0 | 0 | 0 | 0 |
| cg07781082 | 2 | 615 | 708 | 0 | 0 | 0 | 0 | 0 | 0 | 0 | 0 | 0 |
| cg07806343 | 3 | 616 | 706 | 1 | 0 | 0 | 0 | 0 | 0 | 0 | 0 | 0 |
| cg07809462 | 2 | 616 | 707 | 0 | 0 | 0 | 0 | 0 | 0 | 0 | 0 | 0 |
| cg07810091 | 2 | 615 | 708 | 0 | 0 | 0 | 0 | 0 | 0 | 0 | 0 | 0 |
| cg07822777 | 4 | 615 | 2 | 705 | 1 | 0 | 0 | 0 | 0 | 0 | 0 | 0 |
| cg07861180 | 3 | 608 | 8 | 707 | 0 | 0 | 0 | 0 | 0 | 0 | 0 | 0 |
| cg07865580 | 2 | 617 | 706 | 0 | 0 | 0 | 0 | 0 | 0 | 0 | 0 | 0 |
| cg07867687 | 2 | 616 | 707 | 0 | 0 | 0 | 0 | 0 | 0 | 0 | 0 | 0 |
| cg07874334 | 2 | 617 | 706 | 0 | 0 | 0 | 0 | 0 | 0 | 0 | 0 | 0 |
| cg07896193 | 2 | 617 | 706 | 0 | 0 | 0 | 0 | 0 | 0 | 0 | 0 | 0 |
| cg07902213 | 2 | 615 | 708 | 0 | 0 | 0 | 0 | 0 | 0 | 0 | 0 | 0 |
| cg07910525 | 2 | 616 | 707 | 0 | 0 | 0 | 0 | 0 | 0 | 0 | 0 | 0 |
| cg07912337 | 4 | 616 | 1 | 705 | 1 | 0 | 0 | 0 | 0 | 0 | 0 | 0 |
| cg07951153 | 4 | 612 | 2 | 708 | 1 | 0 | 0 | 0 | 0 | 0 | 0 | 0 |
| cg07954607 | 2 | 616 | 707 | 0 | 0 | 0 | 0 | 0 | 0 | 0 | 0 | 0 |
| cg07990395 | 3 | 613 | 1 | 709 | 0 | 0 | 0 | 0 | 0 | 0 | 0 | 0 |
| cg08059778 | 3 | 613 | 2 | 708 | 0 | 0 | 0 | 0 | 0 | 0 | 0 | 0 |
| cg08108619 | 2 | 615 | 708 | 0 | 0 | 0 | 0 | 0 | 0 | 0 | 0 | 0 |
| cg08199506 | 4 | 615 | 1 | 706 | 1 | 0 | 0 | 0 | 0 | 0 | 0 | 0 |
| cg08282969 | 2 | 617 | 706 | 0 | 0 | 0 | 0 | 0 | 0 | 0 | 0 | 0 |
| cg08298085 | 2 | 619 | 704 | 0 | 0 | 0 | 0 | 0 | 0 | 0 | 0 | 0 |
| cg08358587 | 2 | 616 | 707 | 0 | 0 | 0 | 0 | 0 | 0 | 0 | 0 | 0 |
| cg08395966 | 3 | 613 | 3 | 707 | 0 | 0 | 0 | 0 | 0 | 0 | 0 | 0 |
| cg08405463 | 2 | 615 | 708 | 0 | 0 | 0 | 0 | 0 | 0 | 0 | 0 | 0 |
| cg08408091 | 2 | 615 | 708 | 0 | 0 | 0 | 0 | 0 | 0 | 0 | 0 | 0 |
| cg08417382 | 2 | 613 | 710 | 0 | 0 | 0 | 0 | 0 | 0 | 0 | 0 | 0 |
| cg08446187 | 2 | 619 | 704 | 0 | 0 | 0 | 0 | 0 | 0 | 0 | 0 | 0 |
| cg08479532 | 2 | 618 | 705 | 0 | 0 | 0 | 0 | 0 | 0 | 0 | 0 | 0 |
| cg08648877 | 2 | 616 | 707 | 0 | 0 | 0 | 0 | 0 | 0 | 0 | 0 | 0 |
| cg08798116 | 2 | 618 | 705 | 0 | 0 | 0 | 0 | 0 | 0 | 0 | 0 | 0 |
| cg08848711 | 3 | 612 | 4 | 707 | 0 | 0 | 0 | 0 | 0 | 0 | 0 | 0 |
| cg08950703 | 3 | 610 | 8 | 705 | 0 | 0 | 0 | 0 | 0 | 0 | 0 | 0 |
| cg08952424 | 2 | 617 | 706 | 0 | 0 | 0 | 0 | 0 | 0 | 0 | 0 | 0 |
| cg08955276 | 2 | 615 | 708 | 0 | 0 | 0 | 0 | 0 | 0 | 0 | 0 | 0 |
| cg08964402 | 2 | 616 | 707 | 0 | 0 | 0 | 0 | 0 | 0 | 0 | 0 | 0 |
| cg08966380 | 3 | 617 | 704 | 2 | 0 | 0 | 0 | 0 | 0 | 0 | 0 | 0 |
| cg08969352 | 3 | 609 | 7 | 707 | 0 | 0 | 0 | 0 | 0 | 0 | 0 | 0 |
| cg08980509 | 2 | 616 | 707 | 0 | 0 | 0 | 0 | 0 | 0 | 0 | 0 | 0 |
| cg09016243 | 2 | 618 | 705 | 0 | 0 | 0 | 0 | 0 | 0 | 0 | 0 | 0 |
| cg09018810 | 3 | 609 | 7 | 707 | 0 | 0 | 0 | 0 | 0 | 0 | 0 | 0 |
| cg09056691 | 2 | 617 | 706 | 0 | 0 | 0 | 0 | 0 | 0 | 0 | 0 | 0 |
| cg09060772 | 2 | 616 | 707 | 0 | 0 | 0 | 0 | 0 | 0 | 0 | 0 | 0 |
| cg09068301 | 2 | 617 | 706 | 0 | 0 | 0 | 0 | 0 | 0 | 0 | 0 | 0 |
| cg09072865 | 4 | 614 | 2 | 706 | 1 | 0 | 0 | 0 | 0 | 0 | 0 | 0 |
| cg09084933 | 2 | 613 | 710 | 0 | 0 | 0 | 0 | 0 | 0 | 0 | 0 | 0 |
| cg09091181 | 2 | 616 | 707 | 0 | 0 | 0 | 0 | 0 | 0 | 0 | 0 | 0 |
| cg09134165 | 2 | 615 | 708 | 0 | 0 | 0 | 0 | 0 | 0 | 0 | 0 | 0 |
| cg09207137 | 4 | 612 | 4 | 706 | 1 | 0 | 0 | 0 | 0 | 0 | 0 | 0 |
| cg09210933 | 2 | 617 | 706 | 0 | 0 | 0 | 0 | 0 | 0 | 0 | 0 | 0 |
| cg09229960 | 3 | 616 | 706 | 1 | 0 | 0 | 0 | 0 | 0 | 0 | 0 | 0 |
| cg09285672 | 2 | 615 | 708 | 0 | 0 | 0 | 0 | 0 | 0 | 0 | 0 | 0 |
| cg09307104 | 3 | 613 | 4 | 706 | 0 | 0 | 0 | 0 | 0 | 0 | 0 | 0 |
| cg09309899 | 3 | 616 | 705 | 2 | 0 | 0 | 0 | 0 | 0 | 0 | 0 | 0 |
| cg09310980 | 3 | 611 | 7 | 705 | 0 | 0 | 0 | 0 | 0 | 0 | 0 | 0 |
| cg09368074 | 2 | 616 | 707 | 0 | 0 | 0 | 0 | 0 | 0 | 0 | 0 | 0 |
| cg09411587 | 2 | 615 | 708 | 0 | 0 | 0 | 0 | 0 | 0 | 0 | 0 | 0 |
| cg09418475 | 3 | 615 | 707 | 1 | 0 | 0 | 0 | 0 | 0 | 0 | 0 | 0 |
| cg09434493 | 2 | 616 | 707 | 0 | 0 | 0 | 0 | 0 | 0 | 0 | 0 | 0 |
| cg09514431 | 2 | 616 | 707 | 0 | 0 | 0 | 0 | 0 | 0 | 0 | 0 | 0 |
| cg09521623 | 3 | 1 | 709 | 613 | 0 | 0 | 0 | 0 | 0 | 0 | 0 | 0 |
| cg09523186 | 2 | 615 | 708 | 0 | 0 | 0 | 0 | 0 | 0 | 0 | 0 | 0 |
| cg09610589 | 3 | 619 | 703 | 1 | 0 | 0 | 0 | 0 | 0 | 0 | 0 | 0 |
| cg09624684 | 2 | 613 | 710 | 0 | 0 | 0 | 0 | 0 | 0 | 0 | 0 | 0 |
| cg09720515 | 4 | 616 | 704 | 2 | 1 | 0 | 0 | 0 | 0 | 0 | 0 | 0 |
| cg09738386 | 3 | 615 | 706 | 2 | 0 | 0 | 0 | 0 | 0 | 0 | 0 | 0 |
| cg09773839 | 2 | 616 | 707 | 0 | 0 | 0 | 0 | 0 | 0 | 0 | 0 | 0 |
| cg09799350 | 5 | 616 | 704 | 1 | 1 | 1 | 0 | 0 | 0 | 0 | 0 | 0 |
| cg09834142 | 2 | 616 | 707 | 0 | 0 | 0 | 0 | 0 | 0 | 0 | 0 | 0 |
| cg09850561 | 4 | 609 | 5 | 708 | 1 | 0 | 0 | 0 | 0 | 0 | 0 | 0 |
| cg09965404 | 2 | 619 | 704 | 0 | 0 | 0 | 0 | 0 | 0 | 0 | 0 | 0 |
| cg09978401 | 2 | 617 | 706 | 0 | 0 | 0 | 0 | 0 | 0 | 0 | 0 | 0 |
| cg09996779 | 2 | 618 | 705 | 0 | 0 | 0 | 0 | 0 | 0 | 0 | 0 | 0 |
| cg10047502 | 2 | 614 | 709 | 0 | 0 | 0 | 0 | 0 | 0 | 0 | 0 | 0 |
| cg10055097 | 4 | 613 | 2 | 707 | 1 | 0 | 0 | 0 | 0 | 0 | 0 | 0 |
| cg10078687 | 5 | 613 | 706 | 2 | 1 | 1 | 0 | 0 | 0 | 0 | 0 | 0 |
| cg10088372 | 2 | 617 | 706 | 0 | 0 | 0 | 0 | 0 | 0 | 0 | 0 | 0 |
| cg10201390 | 2 | 619 | 704 | 0 | 0 | 0 | 0 | 0 | 0 | 0 | 0 | 0 |
| cg10274815 | 2 | 615 | 708 | 0 | 0 | 0 | 0 | 0 | 0 | 0 | 0 | 0 |
| cg10286673 | 3 | 616 | 706 | 1 | 0 | 0 | 0 | 0 | 0 | 0 | 0 | 0 |
| cg10305945 | 2 | 616 | 707 | 0 | 0 | 0 | 0 | 0 | 0 | 0 | 0 | 0 |
| cg10315562 | 3 | 611 | 7 | 705 | 0 | 0 | 0 | 0 | 0 | 0 | 0 | 0 |
| cg10347293 | 3 | 620 | 702 | 1 | 0 | 0 | 0 | 0 | 0 | 0 | 0 | 0 |
| cg10347326 | 3 | 614 | 5 | 704 | 0 | 0 | 0 | 0 | 0 | 0 | 0 | 0 |
| cg10645578 | 2 | 616 | 707 | 0 | 0 | 0 | 0 | 0 | 0 | 0 | 0 | 0 |
| cg10717149 | 5 | 606 | 7 | 1 | 1 | 708 | 0 | 0 | 0 | 0 | 0 | 0 |
| cg10721440 | 3 | 611 | 710 | 2 | 0 | 0 | 0 | 0 | 0 | 0 | 0 | 0 |
| cg10848980 | 2 | 618 | 705 | 0 | 0 | 0 | 0 | 0 | 0 | 0 | 0 | 0 |
| cg10860619 | 2 | 610 | 713 | 0 | 0 | 0 | 0 | 0 | 0 | 0 | 0 | 0 |
| cg10869581 | 2 | 620 | 703 | 0 | 0 | 0 | 0 | 0 | 0 | 0 | 0 | 0 |
| cg10912974 | 4 | 613 | 2 | 707 | 1 | 0 | 0 | 0 | 0 | 0 | 0 | 0 |
| cg10914789 | 2 | 616 | 707 | 0 | 0 | 0 | 0 | 0 | 0 | 0 | 0 | 0 |
| cg10956264 | 2 | 617 | 706 | 0 | 0 | 0 | 0 | 0 | 0 | 0 | 0 | 0 |
| cg10987536 | 2 | 615 | 708 | 0 | 0 | 0 | 0 | 0 | 0 | 0 | 0 | 0 |
| cg10991108 | 3 | 610 | 6 | 707 | 0 | 0 | 0 | 0 | 0 | 0 | 0 | 0 |
| cg10991514 | 2 | 618 | 705 | 0 | 0 | 0 | 0 | 0 | 0 | 0 | 0 | 0 |
| cg11049774 | 2 | 611 | 712 | 0 | 0 | 0 | 0 | 0 | 0 | 0 | 0 | 0 |
| cg11111131 | 3 | 619 | 3 | 701 | 0 | 0 | 0 | 0 | 0 | 0 | 0 | 0 |
| cg11111271 | 2 | 621 | 702 | 0 | 0 | 0 | 0 | 0 | 0 | 0 | 0 | 0 |
| cg11152253 | 4 | 609 | 7 | 706 | 1 | 0 | 0 | 0 | 0 | 0 | 0 | 0 |
| cg11154719 | 2 | 617 | 706 | 0 | 0 | 0 | 0 | 0 | 0 | 0 | 0 | 0 |
| cg11184697 | 4 | 611 | 6 | 1 | 705 | 0 | 0 | 0 | 0 | 0 | 0 | 0 |
| cg11233153 | 3 | 616 | 706 | 1 | 0 | 0 | 0 | 0 | 0 | 0 | 0 | 0 |
| cg11308037 | 3 | 608 | 8 | 707 | 0 | 0 | 0 | 0 | 0 | 0 | 0 | 0 |
| cg11329209 | 2 | 617 | 706 | 0 | 0 | 0 | 0 | 0 | 0 | 0 | 0 | 0 |
| cg11337025 | 2 | 618 | 705 | 0 | 0 | 0 | 0 | 0 | 0 | 0 | 0 | 0 |
| cg11409998 | 2 | 617 | 706 | 0 | 0 | 0 | 0 | 0 | 0 | 0 | 0 | 0 |
| cg11458217 | 3 | 616 | 706 | 1 | 0 | 0 | 0 | 0 | 0 | 0 | 0 | 0 |
| cg11506821 | 3 | 621 | 701 | 1 | 0 | 0 | 0 | 0 | 0 | 0 | 0 | 0 |
| cg11520843 | 2 | 616 | 707 | 0 | 0 | 0 | 0 | 0 | 0 | 0 | 0 | 0 |
| cg11594566 | 4 | 616 | 2 | 1 | 704 | 0 | 0 | 0 | 0 | 0 | 0 | 0 |
| cg11637006 | 2 | 615 | 708 | 0 | 0 | 0 | 0 | 0 | 0 | 0 | 0 | 0 |
| cg11653314 | 2 | 616 | 707 | 0 | 0 | 0 | 0 | 0 | 0 | 0 | 0 | 0 |
| cg11653864 | 3 | 615 | 1 | 707 | 0 | 0 | 0 | 0 | 0 | 0 | 0 | 0 |
| cg11663393 | 2 | 610 | 713 | 0 | 0 | 0 | 0 | 0 | 0 | 0 | 0 | 0 |
| cg11673471 | 4 | 611 | 3 | 3 | 706 | 0 | 0 | 0 | 0 | 0 | 0 | 0 |
| cg11704790 | 2 | 611 | 712 | 0 | 0 | 0 | 0 | 0 | 0 | 0 | 0 | 0 |
| cg11720358 | 3 | 614 | 2 | 707 | 0 | 0 | 0 | 0 | 0 | 0 | 0 | 0 |
| cg11851349 | 2 | 616 | 707 | 0 | 0 | 0 | 0 | 0 | 0 | 0 | 0 | 0 |
| cg11858468 | 3 | 617 | 1 | 705 | 0 | 0 | 0 | 0 | 0 | 0 | 0 | 0 |
| cg11902811 | 3 | 616 | 1 | 706 | 0 | 0 | 0 | 0 | 0 | 0 | 0 | 0 |
| cg12026625 | 3 | 617 | 1 | 705 | 0 | 0 | 0 | 0 | 0 | 0 | 0 | 0 |
| cg12058262 | 2 | 616 | 707 | 0 | 0 | 0 | 0 | 0 | 0 | 0 | 0 | 0 |
| cg12064531 | 3 | 614 | 5 | 704 | 0 | 0 | 0 | 0 | 0 | 0 | 0 | 0 |
| cg12075609 | 4 | 616 | 1 | 705 | 1 | 0 | 0 | 0 | 0 | 0 | 0 | 0 |
| cg12165338 | 2 | 618 | 705 | 0 | 0 | 0 | 0 | 0 | 0 | 0 | 0 | 0 |
| cg12166502 | 4 | 609 | 4 | 4 | 706 | 0 | 0 | 0 | 0 | 0 | 0 | 0 |
| cg12277627 | 3 | 619 | 1 | 703 | 0 | 0 | 0 | 0 | 0 | 0 | 0 | 0 |
| cg12284142 | 3 | 614 | 4 | 705 | 0 | 0 | 0 | 0 | 0 | 0 | 0 | 0 |
| cg12298823 | 3 | 610 | 5 | 708 | 0 | 0 | 0 | 0 | 0 | 0 | 0 | 0 |
| cg12375308 | 2 | 616 | 707 | 0 | 0 | 0 | 0 | 0 | 0 | 0 | 0 | 0 |
| cg12413138 | 3 | 609 | 8 | 706 | 0 | 0 | 0 | 0 | 0 | 0 | 0 | 0 |
| cg12419462 | 2 | 616 | 707 | 0 | 0 | 0 | 0 | 0.00E+00 | 0 | 0.00E+00 | 0 | 0 |
| cg12447182 | 2 | 618 | 705 | 0 | 0 | 0 | 0 | 0 | 0 | 0 | 0 | 0 |
| cg12452512 | 2 | 620 | 703 | 0 | 0 | 0 | 0 | 0 | 0 | 0 | 0 | 0 |
| cg12472218 | 5 | 618 | 2 | 701 | 1 | 1 | 0 | 0 | 0 | 0 | 0 | 0 |
| cg12521790 | 3 | 606 | 9 | 708 | 0 | 0 | 0 | 0 | 0 | 0 | 0 | 0 |
| cg12584551 | 3 | 616 | 705 | 2 | 0 | 0 | 0 | 0 | 0 | 0 | 0 | 0 |
| cg12598428 | 3 | 612 | 2 | 709 | 0 | 0 | 0 | 0 | 0 | 0 | 0 | 0 |
| cg12614178 | 2 | 613 | 710 | 0 | 0 | 0 | 0 | 0 | 0 | 0 | 0 | 0 |
| cg12614702 | 3 | 611 | 1 | 711 | 0 | 0 | 0 | 0 | 0 | 0 | 0 | 0 |
| cg12704708 | 3 | 614 | 708 | 1 | 0 | 0 | 0 | 0 | 0 | 0 | 0 | 0 |
| cg12709057 | 4 | 614 | 1 | 1 | 707 | 0 | 0 | 0 | 0 | 0 | 0 | 0 |
| cg12747864 | 2 | 617 | 706 | 0 | 0 | 0 | 0 | 0 | 0 | 0 | 0 | 0 |
| cg12814550 | 3 | 615 | 706 | 2 | 0 | 0 | 0 | 0 | 0 | 0 | 0 | 0 |
| cg12920408 | 2 | 618 | 705 | 0 | 0 | 0 | 0 | 0 | 0 | 0 | 0 | 0 |
| cg12935118 | 4 | 613 | 1 | 1 | 708 | 0 | 0 | 0 | 0 | 0 | 0 | 0 |
| cg12944030 | 3 | 616 | 706 | 1 | 0 | 0 | 0 | 0 | 0 | 0 | 0 | 0 |
| cg12980185 | 2 | 619 | 704 | 0 | 0 | 0 | 0 | 0 | 0 | 0 | 0 | 0 |
| cg13014545 | 3 | 609 | 8 | 706 | 0 | 0 | 0 | 0 | 0 | 0 | 0 | 0 |
| cg13014982 | 2 | 616 | 707 | 0 | 0 | 0 | 0 | 0 | 0 | 0 | 0 | 0 |
| cg13023833 | 2 | 608 | 715 | 0 | 0 | 0 | 0 | 0 | 0 | 0 | 0 | 0 |
| cg13024624 | 2 | 614 | 709 | 0 | 0 | 0 | 0 | 0 | 0 | 0 | 0 | 0 |
| cg13115118 | 3 | 616 | 1 | 706 | 0 | 0 | 0 | 0 | 0 | 0 | 0 | 0 |
| cg13115455 | 3 | 615 | 1 | 707 | 0 | 0 | 0 | 0 | 0 | 0 | 0 | 0 |
| cg13120260 | 2 | 616 | 707 | 0 | 0 | 0 | 0 | 0 | 0 | 0 | 0 | 0 |
| cg13130271 | 3 | 609 | 7 | 707 | 0 | 0 | 0 | 0 | 0 | 0 | 0 | 0 |
| cg13178935 | 3 | 614 | 1 | 708 | 0 | 0 | 0 | 0 | 0 | 0 | 0 | 0 |
| cg13182820 | 4 | 611 | 5 | 706 | 1 | 0 | 0 | 0 | 0 | 0 | 0 | 0 |
| cg13203541 | 4 | 608 | 5 | 3 | 707 | 0 | 0 | 0 | 0 | 0 | 0 | 0 |
| cg13232664 | 2 | 616 | 707 | 0 | 0 | 0 | 0 | 0 | 0 | 0 | 0 | 0 |
| cg13234839 | 2 | 619 | 704 | 0 | 0 | 0 | 0 | 0 | 0 | 0 | 0 | 0 |
| cg13240932 | 3 | 615 | 1 | 707 | 0 | 0 | 0 | 0 | 0 | 0 | 0 | 0 |
| cg13241003 | 2 | 616 | 707 | 0 | 0 | 0 | 0 | 0 | 0 | 0 | 0 | 0 |
| cg13304035 | 3 | 616 | 705 | 2 | 0 | 0 | 0 | 0 | 0 | 0 | 0 | 0 |
| cg13307142 | 3 | 616 | 706 | 1 | 0 | 0 | 0 | 0 | 0 | 0 | 0 | 0 |
| cg13369210 | 2 | 617 | 706 | 0 | 0 | 0 | 0 | 0 | 0 | 0 | 0 | 0 |
| cg13574945 | 4 | 609 | 4 | 3 | 707 | 0 | 0 | 0 | 0 | 0 | 0 | 0 |
| cg13663706 | 2 | 616 | 707 | 0 | 0 | 0 | 0 | 0 | 0 | 0 | 0 | 0 |
| cg13664654 | 3 | 615 | 707 | 1 | 0 | 0 | 0 | 0 | 0 | 0 | 0 | 0 |
| cg13680016 | 2 | 615 | 708 | 0 | 0 | 0 | 0 | 0 | 0 | 0 | 0 | 0 |
| cg13682241 | 2 | 617 | 706 | 0 | 0 | 0 | 0 | 0 | 0 | 0 | 0 | 0 |
| cg13766601 | 2 | 616 | 707 | 0 | 0 | 0 | 0 | 0 | 0 | 0 | 0 | 0 |
| cg13771263 | 3 | 613 | 709 | 1 | 0 | 0 | 0 | 0 | 0 | 0 | 0 | 0 |
| cg13797960 | 3 | 611 | 6 | 706 | 0 | 0 | 0 | 0 | 0 | 0 | 0 | 0 |
| cg13915481 | 3 | 615 | 707 | 1 | 0 | 0 | 0 | 0 | 0 | 0 | 0 | 0 |
| cg13918312 | 3 | 615 | 707 | 1 | 0 | 0 | 0 | 0 | 0 | 0 | 0 | 0 |
| cg13941987 | 6 | 604 | 8 | 1 | 1 | 708 | 1 | 0 | 0 | 0 | 0 | 0 |
| cg14007036 | 2 | 614 | 709 | 0 | 0 | 0 | 0 | 0 | 0 | 0 | 0 | 0 |
| cg14061423 | 2 | 613 | 710 | 0 | 0 | 0 | 0 | 0 | 0 | 0 | 0 | 0 |
| cg14127907 | 3 | 615 | 707 | 1 | 0 | 0 | 0 | 0 | 0 | 0 | 0 | 0 |
| cg14191108 | 3 | 613 | 709 | 1 | 0 | 0 | 0 | 0 | 0 | 0 | 0 | 0 |
| cg14214586 | 3 | 615 | 707 | 1 | 0 | 0 | 0 | 0 | 0 | 0 | 0 | 0 |
| cg14247154 | 2 | 615 | 708 | 0 | 0 | 0 | 0 | 0 | 0 | 0 | 0 | 0 |
| cg14295915 | 3 | 610 | 10 | 703 | 0 | 0 | 0 | 0 | 0 | 0 | 0 | 0 |
| cg14308265 | 2 | 615 | 708 | 0 | 0 | 0 | 0 | 0 | 0 | 0 | 0 | 0 |
| cg14350469 | 3 | 605 | 12 | 706 | 0 | 0 | 0 | 0 | 0 | 0 | 0 | 0 |
| cg14388993 | 2 | 617 | 706 | 0 | 0 | 0 | 0 | 0 | 0 | 0 | 0 | 0 |
| cg14513804 | 2 | 620 | 703 | 0 | 0 | 0 | 0 | 0 | 0 | 0 | 0 | 0 |
| cg14720319 | 2 | 616 | 707 | 0 | 0 | 0 | 0 | 0 | 0 | 0 | 0 | 0 |
| cg14812623 | 2 | 619 | 704 | 0 | 0 | 0 | 0 | 0 | 0 | 0 | 0 | 0 |
| cg14927076 | 4 | 613 | 1 | 707 | 2 | 0 | 0 | 0 | 0 | 0 | 0 | 0 |
| cg14949922 | 2 | 615 | 708 | 0 | 0 | 0 | 0 | 0 | 0 | 0 | 0 | 0 |
| cg14970569 | 3 | 615 | 1 | 707 | 0 | 0 | 0 | 0 | 0 | 0 | 0 | 0 |
| cg14972002 | 3 | 618 | 1 | 704 | 0 | 0 | 0 | 0 | 0 | 0 | 0 | 0 |
| cg14990368 | 3 | 615 | 707 | 1 | 0 | 0 | 0 | 0 | 0 | 0 | 0 | 0 |
| cg15043283 | 2 | 614 | 709 | 0 | 0 | 0 | 0 | 0 | 0 | 0 | 0 | 0 |
| cg15165114 | 4 | 608 | 2 | 6 | 707 | 0 | 0 | 0 | 0 | 0 | 0 | 0 |
| cg15165694 | 2 | 619 | 704 | 0 | 0 | 0 | 0 | 0 | 0 | 0 | 0 | 0 |
| cg15257930 | 2 | 605 | 718 | 0 | 0 | 0 | 0 | 0 | 0 | 0 | 0 | 0 |
| cg15317927 | 2 | 615 | 708 | 0 | 0 | 0 | 0 | 0 | 0 | 0 | 0 | 0 |
| cg15391239 | 2 | 612 | 711 | 0 | 0 | 0 | 0 | 0 | 0 | 0 | 0 | 0 |
| cg15392343 | 2 | 617 | 706 | 0 | 0 | 0 | 0 | 0 | 0 | 0 | 0 | 0 |
| cg15418221 | 4 | 608 | 7 | 1 | 707 | 0 | 0 | 0 | 0 | 0 | 0 | 0 |
| cg15450782 | 2 | 613 | 710 | 0 | 0 | 0 | 0 | 0 | 0 | 0 | 0 | 0 |
| cg15452747 | 2 | 617 | 706 | 0 | 0 | 0 | 0 | 0 | 0 | 0 | 0 | 0 |
| cg15511490 | 3 | 618 | 704 | 1 | 0 | 0 | 0 | 0 | 0 | 0 | 0 | 0 |
| cg15511516 | 6 | 609 | 4 | 2 | 704 | 1 | 3 | 0 | 0 | 0 | 0 | 0 |
| cg15521289 | 2 | 615 | 708 | 0 | 0 | 0 | 0 | 0 | 0 | 0 | 0 | 0 |
| cg15565409 | 3 | 617 | 2 | 704 | 0 | 0 | 0 | 0 | 0 | 0 | 0 | 0 |
| cg15579650 | 3 | 614 | 6 | 703 | 0 | 0 | 0 | 0 | 0 | 0 | 0 | 0 |
| cg15582794 | 3 | 615 | 707 | 1 | 0 | 0 | 0 | 0 | 0 | 0 | 0 | 0 |
| cg15685776 | 2 | 619 | 704 | 0 | 0 | 0 | 0 | 0 | 0 | 0 | 0 | 0 |
| cg15755924 | 2 | 615 | 708 | 0 | 0 | 0 | 0 | 0 | 0 | 0 | 0 | 0 |
| cg15757320 | 2 | 617 | 706 | 0 | 0 | 0 | 0 | 0 | 0 | 0 | 0 | 0 |
| cg15769969 | 3 | 615 | 707 | 1 | 0 | 0 | 0 | 0 | 0 | 0 | 0 | 0 |
| cg15771339 | 2 | 615 | 708 | 0 | 0 | 0 | 0 | 0 | 0 | 0 | 0 | 0 |
| cg15841434 | 2 | 619 | 704 | 0 | 0 | 0 | 0 | 0 | 0 | 0 | 0 | 0 |
| cg15858894 | 2 | 615 | 708 | 0 | 0 | 0 | 0 | 0 | 0 | 0 | 0 | 0 |
| cg15891447 | 3 | 617 | 704 | 2 | 0 | 0 | 0 | 0 | 0 | 0 | 0 | 0 |
| cg15906052 | 2 | 616 | 707 | 0 | 0 | 0 | 0 | 0 | 0 | 0 | 0 | 0 |
| cg15922956 | 4 | 616 | 705 | 1 | 1 | 0 | 0 | 0 | 0 | 0 | 0 | 0 |
| cg16059374 | 3 | 615 | 706 | 2 | 0 | 0 | 0 | 0 | 0 | 0 | 0 | 0 |
| cg16097380 | 2 | 616 | 707 | 0 | 0 | 0 | 0 | 0 | 0 | 0 | 0 | 0 |
| cg16181678 | 2 | 616 | 707 | 0 | 0 | 0 | 0 | 0 | 0 | 0 | 0 | 0 |
| cg16211147 | 2 | 620 | 703 | 0 | 0 | 0 | 0 | 0 | 0 | 0 | 0 | 0 |
| cg16221895 | 4 | 608 | 5 | 2 | 708 | 0 | 0 | 0 | 0 | 0 | 0 | 0 |
| cg16245086 | 3 | 613 | 709 | 1 | 0 | 0 | 0 | 0 | 0 | 0 | 0 | 0 |
| cg16269097 | 2 | 614 | 709 | 0 | 0 | 0 | 0 | 0 | 0 | 0 | 0 | 0 |
| cg16272791 | 2 | 616 | 707 | 0 | 0 | 0 | 0 | 0 | 0 | 0 | 0 | 0 |
| cg16315447 | 4 | 614 | 706 | 1 | 2 | 0 | 0 | 0 | 0 | 0 | 0 | 0 |
| cg16318983 | 2 | 615 | 708 | 0 | 0 | 0 | 0 | 0 | 0 | 0 | 0 | 0 |
| cg16328033 | 2 | 612 | 711 | 0 | 0 | 0 | 0 | 0 | 0 | 0 | 0 | 0 |
| cg16412513 | 2 | 616 | 707 | 0 | 0 | 0 | 0 | 0 | 0 | 0 | 0 | 0 |
| cg16431713 | 2 | 617 | 706 | 0 | 0 | 0 | 0 | 0 | 0 | 0 | 0 | 0 |
| cg16488754 | 4 | 613 | 2 | 1 | 707 | 0 | 0 | 0 | 0 | 0 | 0 | 0 |
| cg16501779 | 4 | 617 | 703 | 2 | 1 | 0 | 0 | 0 | 0 | 0 | 0 | 0 |
| cg16510200 | 3 | 615 | 1 | 707 | 0 | 0 | 0 | 0 | 0 | 0 | 0 | 0 |
| cg16515238 | 3 | 614 | 1 | 708 | 0 | 0 | 0 | 0 | 0 | 0 | 0 | 0 |
| cg16590821 | 2 | 614 | 709 | 0 | 0 | 0 | 0 | 0 | 0 | 0 | 0 | 0 |
| cg16617830 | 2 | 616 | 707 | 0 | 0 | 0 | 0 | 0 | 0 | 0 | 0 | 0 |
| cg16626088 | 2 | 615 | 708 | 0 | 0 | 0 | 0 | 0 | 0 | 0 | 0 | 0 |
| cg16638301 | 3 | 614 | 2 | 707 | 0 | 0 | 0 | 0 | 0 | 0 | 0 | 0 |
| cg16641060 | 3 | 614 | 3 | 706 | 0 | 0 | 0 | 0 | 0 | 0 | 0 | 0 |
| cg16680922 | 3 | 611 | 6 | 706 | 0 | 0 | 0 | 0 | 0 | 0 | 0 | 0 |
| cg16716035 | 2 | 616 | 707 | 0 | 0 | 0 | 0 | 0 | 0 | 0 | 0 | 0 |
| cg16734817 | 4 | 616 | 2 | 704 | 1 | 0 | 0 | 0 | 0 | 0 | 0 | 0 |
| cg16778620 | 3 | 614 | 708 | 1 | 0 | 0 | 0 | 0 | 0 | 0 | 0 | 0 |
| cg16870351 | 3 | 616 | 706 | 1 | 0 | 0 | 0 | 0 | 0 | 0 | 0 | 0 |
| cg16940942 | 2 | 617 | 706 | 0 | 0 | 0 | 0 | 0 | 0 | 0 | 0 | 0 |
| cg16976875 | 3 | 614 | 4 | 705 | 0 | 0 | 0 | 0 | 0 | 0 | 0 | 0 |
| cg16984885 | 2 | 616 | 707 | 0 | 0 | 0 | 0 | 0 | 0 | 0 | 0 | 0 |
| cg16991511 | 2 | 614 | 709 | 0 | 0 | 0 | 0 | 0 | 0 | 0 | 0 | 0 |
| cg16998810 | 2 | 618 | 705 | 0 | 0 | 0 | 0 | 0 | 0 | 0 | 0 | 0 |
| cg17036062 | 2 | 616 | 707 | 0 | 0 | 0 | 0 | 0 | 0 | 0 | 0 | 0 |
| cg17058724 | 2 | 615 | 708 | 0 | 0 | 0 | 0 | 0 | 0 | 0 | 0 | 0 |
| cg17149359 | 2 | 618 | 705 | 0 | 0 | 0 | 0 | 0 | 0 | 0 | 0 | 0 |
| cg17195879 | 2 | 619 | 704 | 0 | 0 | 0 | 0 | 0 | 0 | 0 | 0 | 0 |
| cg17246352 | 2 | 618 | 705 | 0 | 0 | 0 | 0 | 0 | 0 | 0 | 0 | 0 |
| cg17292622 | 3 | 614 | 1 | 708 | 0 | 0 | 0 | 0 | 0 | 0 | 0 | 0 |
| cg17363084 | 2 | 618 | 705 | 0 | 0 | 0 | 0 | 0 | 0 | 0 | 0 | 0 |
| cg17396400 | 3 | 610 | 7 | 706 | 0 | 0 | 0 | 0 | 0 | 0 | 0 | 0 |
| cg17399684 | 2 | 613 | 710 | 0 | 0 | 0 | 0 | 0 | 0 | 0 | 0 | 0 |
| cg17479100 | 2 | 616 | 707 | 0 | 0 | 0 | 0 | 0 | 0 | 0 | 0 | 0 |
| cg17547524 | 3 | 619 | 703 | 1 | 0 | 0 | 0 | 0 | 0 | 0 | 0 | 0 |
| cg17592148 | 3 | 614 | 707 | 2 | 0 | 0 | 0 | 0 | 0 | 0 | 0 | 0 |
| cg17607496 | 2 | 620 | 703 | 0 | 0 | 0 | 0 | 0 | 0 | 0 | 0 | 0 |
| cg17624691 | 2 | 615 | 708 | 0 | 0 | 0 | 0 | 0 | 0 | 0 | 0 | 0 |
| cg17639056 | 3 | 612 | 2 | 709 | 0 | 0 | 0 | 0 | 0 | 0 | 0 | 0 |
| cg17736560 | 2 | 616 | 707 | 0 | 0 | 0 | 0 | 0 | 0 | 0 | 0 | 0 |
| cg17776579 | 2 | 619 | 704 | 0 | 0 | 0 | 0 | 0 | 0 | 0 | 0 | 0 |
| cg17788031 | 2 | 619 | 704 | 0 | 0 | 0 | 0 | 0 | 0 | 0 | 0 | 0 |
| cg17800714 | 2 | 616 | 707 | 0 | 0 | 0 | 0 | 0 | 0 | 0 | 0 | 0 |
| cg17811760 | 2 | 616 | 707 | 0 | 0 | 0 | 0 | 0 | 0 | 0 | 0 | 0 |
| cg17824401 | 3 | 615 | 707 | 1 | 0 | 0 | 0 | 0 | 0 | 0 | 0 | 0 |
| cg17831869 | 2 | 617 | 706 | 0 | 0 | 0 | 0 | 0 | 0 | 0 | 0 | 0 |
| cg17849117 | 2 | 617 | 706 | 0 | 0 | 0 | 0 | 0 | 0 | 0 | 0 | 0 |
| cg17877174 | 2 | 618 | 705 | 0 | 0 | 0 | 0 | 0 | 0 | 0 | 0 | 0 |
| cg17917970 | 2 | 613 | 710 | 0 | 0 | 0 | 0 | 0 | 0 | 0 | 0 | 0 |
| cg18031773 | 2 | 618 | 705 | 0 | 0 | 0 | 0 | 0 | 0 | 0 | 0 | 0 |
| cg18102950 | 3 | 611 | 5 | 707 | 0 | 0 | 0 | 0 | 0 | 0 | 0 | 0 |
| cg18112782 | 2 | 616 | 707 | 0 | 0 | 0 | 0 | 0 | 0 | 0 | 0 | 0 |
| cg18124907 | 4 | 616 | 1 | 705 | 1 | 0 | 0 | 0 | 0 | 0 | 0 | 0 |
| cg18140045 | 3 | 614 | 3 | 706 | 0 | 0 | 0 | 0 | 0 | 0 | 0 | 0 |
| cg18288715 | 2 | 611 | 712 | 0 | 0 | 0 | 0 | 0 | 0 | 0 | 0 | 0 |
| cg18383984 | 3 | 618 | 703 | 2 | 0 | 0 | 0 | 0 | 0 | 0 | 0 | 0 |
| cg18395382 | 3 | 615 | 707 | 1 | 0 | 0 | 0 | 0 | 0 | 0 | 0 | 0 |
| cg18397310 | 2 | 612 | 711 | 0 | 0 | 0 | 0 | 0 | 0 | 0 | 0 | 0 |
| cg18406472 | 3 | 616 | 704 | 3 | 0 | 0 | 0 | 0 | 0 | 0 | 0 | 0 |
| cg18426869 | 2 | 617 | 706 | 0 | 0 | 0 | 0 | 0 | 0 | 0 | 0 | 0 |
| cg18632412 | 2 | 614 | 709 | 0 | 0 | 0 | 0 | 0 | 0 | 0 | 0 | 0 |
| cg18636716 | 3 | 618 | 2 | 703 | 0 | 0 | 0 | 0 | 0 | 0 | 0 | 0 |
| cg18689730 | 2 | 616 | 707 | 0 | 0 | 0 | 0 | 0 | 0 | 0 | 0 | 0 |
| cg18717600 | 2 | 621 | 702 | 0 | 0 | 0 | 0 | 0 | 0 | 0 | 0 | 0 |
| cg18742441 | 2 | 618 | 705 | 0 | 0 | 0 | 0 | 0 | 0 | 0 | 0 | 0 |
| cg18769303 | 2 | 620 | 703 | 0 | 0 | 0 | 0 | 0 | 0 | 0 | 0 | 0 |
| cg18816329 | 2 | 615 | 708 | 0 | 0 | 0 | 0 | 0 | 0 | 0 | 0 | 0 |
| cg18818432 | 3 | 618 | 704 | 1 | 0 | 0 | 0 | 0 | 0 | 0 | 0 | 0 |
| cg18828303 | 4 | 617 | 1 | 703 | 2 | 0 | 0 | 0 | 0 | 0 | 0 | 0 |
| cg18904224 | 2 | 616 | 707 | 0 | 0 | 0 | 0 | 0 | 0 | 0 | 0 | 0 |
| cg18932686 | 2 | 615 | 708 | 0 | 0 | 0 | 0 | 0 | 0 | 0 | 0 | 0 |
| cg18983709 | 2 | 614 | 709 | 0 | 0 | 0 | 0 | 0 | 0 | 0 | 0 | 0 |
| cg18989810 | 2 | 615 | 708 | 0 | 0 | 0 | 0 | 0 | 0 | 0 | 0 | 0 |
| cg18998000 | 3 | 611 | 5 | 707 | 0 | 0 | 0 | 0 | 0 | 0 | 0 | 0 |
| cg19005062 | 3 | 608 | 7 | 708 | 0 | 0 | 0 | 0 | 0 | 0 | 0 | 0 |
| cg19056391 | 4 | 615 | 1 | 1 | 706 | 0 | 0 | 0 | 0 | 0 | 0 | 0 |
| cg19060387 | 4 | 614 | 1 | 707 | 1 | 0 | 0 | 0 | 0 | 0 | 0 | 0 |
| cg19068385 | 2 | 619 | 704 | 0 | 0 | 0 | 0 | 0 | 0 | 0 | 0 | 0 |
| cg19162841 | 2 | 619 | 704 | 0 | 0 | 0 | 0 | 0 | 0 | 0 | 0 | 0 |
| cg19238394 | 3 | 612 | 7 | 704 | 0 | 0 | 0 | 0 | 0 | 0 | 0 | 0 |
| cg19265708 | 2 | 617 | 706 | 0 | 0 | 0 | 0 | 0 | 0 | 0 | 0 | 0 |
| cg19318186 | 2 | 616 | 707 | 0 | 0 | 0 | 0 | 0 | 0 | 0 | 0 | 0 |
| cg19355911 | 2 | 609 | 714 | 0 | 0 | 0 | 0 | 0 | 0 | 0 | 0 | 0 |
| cg19398192 | 2 | 614 | 709 | 0 | 0 | 0 | 0 | 0 | 0 | 0 | 0 | 0 |
| cg19415116 | 2 | 616 | 707 | 0 | 0 | 0 | 0 | 0 | 0 | 0 | 0 | 0 |
| cg19415339 | 4 | 615 | 2 | 703 | 3 | 0 | 0 | 0 | 0 | 0 | 0 | 0 |
| cg19428430 | 4 | 616 | 2 | 704 | 1 | 0 | 0 | 0 | 0 | 0 | 0 | 0 |
| cg19441529 | 2 | 615 | 708 | 0 | 0 | 0 | 0 | 0 | 0 | 0 | 0 | 0 |
| cg19493242 | 2 | 614 | 709 | 0 | 0 | 0 | 0 | 0 | 0 | 0 | 0 | 0 |
| cg19505129 | 2 | 611 | 712 | 0 | 0 | 0 | 0 | 0 | 0 | 0 | 0 | 0 |
| cg19525496 | 2 | 612 | 711 | 0 | 0 | 0 | 0 | 0 | 0 | 0 | 0 | 0 |
| cg19528502 | 2 | 615 | 708 | 0 | 0 | 0 | 0 | 0 | 0 | 0 | 0 | 0 |
| cg19572135 | 3 | 615 | 707 | 1 | 0 | 0 | 0 | 0 | 0 | 0 | 0 | 0 |
| cg19579913 | 2 | 616 | 707 | 0 | 0 | 0 | 0 | 0 | 0 | 0 | 0 | 0 |
| cg19587616 | 2 | 612 | 711 | 0 | 0 | 0 | 0 | 0 | 0 | 0 | 0 | 0 |
| cg19635884 | 3 | 615 | 1 | 707 | 0 | 0 | 0 | 0 | 0 | 0 | 0 | 0 |
| cg19638040 | 2 | 615 | 708 | 0 | 0 | 0 | 0 | 0 | 0 | 0 | 0 | 0 |
| cg19718903 | 3 | 612 | 6 | 705 | 0 | 0 | 0 | 0 | 0 | 0 | 0 | 0 |
| cg19736647 | 3 | 615 | 707 | 1 | 0 | 0 | 0 | 0 | 0 | 0 | 0 | 0 |
| cg19780807 | 4 | 613 | 708 | 1 | 1 | 0 | 0 | 0 | 0 | 0 | 0 | 0 |
| cg19800913 | 2 | 613 | 710 | 0 | 0 | 0 | 0 | 0 | 0 | 0 | 0 | 0 |
| cg19834421 | 2 | 619 | 704 | 0 | 0 | 0 | 0 | 0 | 0 | 0 | 0 | 0 |
| cg19859323 | 2 | 612 | 711 | 0 | 0 | 0 | 0 | 0 | 0 | 0 | 0 | 0 |
| cg19937618 | 2 | 615 | 708 | 0 | 0 | 0 | 0 | 0 | 0 | 0 | 0 | 0 |
| cg19939909 | 3 | 608 | 7 | 708 | 0 | 0 | 0 | 0 | 0 | 0 | 0 | 0 |
| cg19944582 | 2 | 615 | 708 | 0 | 0 | 0 | 0 | 0 | 0 | 0 | 0 | 0 |
| cg19992190 | 3 | 614 | 2 | 707 | 0 | 0 | 0 | 0 | 0 | 0 | 0 | 0 |
| cg19998137 | 3 | 613 | 3 | 707 | 0 | 0 | 0 | 0 | 0 | 0 | 0 | 0 |
| cg20015269 | 2 | 617 | 706 | 0 | 0 | 0 | 0 | 0 | 0 | 0 | 0 | 0 |
| cg20074774 | 2 | 617 | 706 | 0 | 0 | 0 | 0 | 0 | 0 | 0 | 0 | 0 |
| cg20077602 | 2 | 618 | 705 | 0 | 0 | 0 | 0 | 0 | 0 | 0 | 0 | 0 |
| cg20113724 | 2 | 610 | 713 | 0 | 0 | 0 | 0 | 0 | 0 | 0 | 0 | 0 |
| cg20121427 | 3 | 614 | 708 | 1 | 0 | 0 | 0 | 0 | 0 | 0 | 0 | 0 |
| cg20207108 | 2 | 616 | 707 | 0 | 0 | 0 | 0 | 0 | 0 | 0 | 0 | 0 |
| cg20208613 | 3 | 612 | 3 | 708 | 0 | 0 | 0 | 0 | 0 | 0 | 0 | 0 |
| cg20348344 | 2 | 711 | 612 | 0 | 0 | 0 | 0 | 0 | 0 | 0 | 0 | 0 |
| cg20359784 | 2 | 616 | 707 | 0 | 0 | 0 | 0 | 0 | 0 | 0 | 0 | 0 |
| cg20420363 | 2 | 616 | 707 | 0 | 0 | 0 | 0 | 0 | 0 | 0 | 0 | 0 |
| cg20421037 | 4 | 611 | 4 | 1 | 707 | 0 | 0 | 0 | 0 | 0 | 0 | 0 |
| cg20439892 | 3 | 610 | 8 | 705 | 0 | 0 | 0 | 0 | 0 | 0 | 0 | 0 |
| cg20442640 | 2 | 618 | 705 | 0 | 0 | 0 | 0 | 0 | 0 | 0 | 0 | 0 |
| cg20445038 | 2 | 619 | 704 | 0 | 0 | 0 | 0 | 0 | 0 | 0 | 0 | 0 |
| cg20455959 | 3 | 611 | 6 | 706 | 0 | 0 | 0 | 0 | 0 | 0 | 0 | 0 |
| cg20484507 | 2 | 616 | 707 | 0 | 0 | 0 | 0 | 0 | 0 | 0 | 0 | 0 |
| cg20498086 | 4 | 609 | 4 | 2 | 708 | 0 | 0 | 0 | 0 | 0 | 0 | 0 |
| cg20517444 | 2 | 614 | 709 | 0 | 0 | 0 | 0 | 0 | 0 | 0 | 0 | 0 |
| cg20749341 | 4 | 607 | 9 | 1 | 706 | 0 | 0 | 0 | 0 | 0 | 0 | 0 |
| cg20766178 | 4 | 606 | 3 | 7 | 707 | 0 | 0 | 0 | 0 | 0 | 0 | 0 |
| cg20767561 | 3 | 617 | 1 | 705 | 0 | 0 | 0 | 0 | 0 | 0 | 0 | 0 |
| cg20781383 | 3 | 615 | 3 | 705 | 0 | 0 | 0 | 0 | 0 | 0 | 0 | 0 |
| cg20858239 | 3 | 618 | 704 | 1 | 0 | 0 | 0 | 0 | 0 | 0 | 0 | 0 |
| cg20859738 | 2 | 615 | 708 | 0 | 0 | 0 | 0 | 0 | 0 | 0 | 0 | 0 |
| cg20948364 | 3 | 616 | 706 | 1 | 0 | 0 | 0 | 0 | 0 | 0 | 0 | 0 |
| cg21016188 | 2 | 616 | 707 | 0 | 0 | 0 | 0 | 0 | 0 | 0 | 0 | 0 |
| cg21137943 | 2 | 618 | 705 | 0 | 0 | 0 | 0 | 0 | 0 | 0 | 0 | 0 |
| cg21159768 | 3 | 614 | 708 | 1 | 0 | 0 | 0 | 0 | 0 | 0 | 0 | 0 |
| cg21179956 | 2 | 615 | 708 | 0 | 0 | 0 | 0 | 0 | 0 | 0 | 0 | 0 |
| cg21205654 | 2 | 616 | 707 | 0 | 0 | 0 | 0 | 0 | 0 | 0 | 0 | 0 |
| cg21284493 | 3 | 616 | 1 | 706 | 0 | 0 | 0 | 0 | 0 | 0 | 0 | 0 |
| cg21290550 | 3 | 614 | 1 | 708 | 0 | 0 | 0 | 0 | 0 | 0 | 0 | 0 |
| cg21290775 | 3 | 614 | 708 | 1 | 0 | 0 | 0 | 0 | 0 | 0 | 0 | 0 |
| cg21304892 | 3 | 615 | 2 | 706 | 0 | 0 | 0 | 0 | 0 | 0 | 0 | 0 |
| cg21327581 | 4 | 614 | 2 | 706 | 1 | 0 | 0 | 0 | 0 | 0 | 0 | 0 |
| cg21331369 | 3 | 616 | 706 | 1 | 0 | 0 | 0 | 0 | 0 | 0 | 0 | 0 |
| cg21473514 | 3 | 616 | 705 | 2 | 0 | 0 | 0 | 0 | 0 | 0 | 0 | 0 |
| cg21544051 | 2 | 615 | 708 | 0 | 0 | 0 | 0 | 0 | 0 | 0 | 0 | 0 |
| cg21545785 | 3 | 616 | 706 | 1 | 0 | 0 | 0 | 0 | 0 | 0 | 0 | 0 |
| cg21557668 | 3 | 618 | 2 | 703 | 0 | 0 | 0 | 0 | 0 | 0 | 0 | 0 |
| cg21634944 | 2 | 637 | 686 | 0 | 0 | 0 | 0 | 0 | 0 | 0 | 0 | 0 |
| cg21635596 | 2 | 618 | 705 | 0 | 0 | 0 | 0 | 0 | 0 | 0 | 0 | 0 |
| cg21864829 | 2 | 616 | 707 | 0 | 0 | 0 | 0 | 0 | 0 | 0 | 0 | 0 |
| cg21887683 | 2 | 615 | 708 | 0 | 0 | 0 | 0 | 0 | 0 | 0 | 0 | 0 |
| cg21890239 | 3 | 618 | 704 | 1 | 0 | 0 | 0 | 0 | 0 | 0 | 0 | 0 |
| cg21911363 | 2 | 614 | 709 | 0 | 0 | 0 | 0 | 0 | 0 | 0 | 0 | 0 |
| cg21953876 | 3 | 609 | 9 | 705 | 0 | 0 | 0 | 0 | 0 | 0 | 0 | 0 |
| cg21966453 | 3 | 614 | 3 | 706 | 0 | 0 | 0 | 0 | 0 | 0 | 0 | 0 |
| cg21978299 | 3 | 619 | 703 | 1 | 0 | 0 | 0 | 0 | 0 | 0 | 0 | 0 |
| cg21983484 | 4 | 613 | 5 | 2 | 703 | 0 | 0 | 0 | 0 | 0 | 0 | 0 |
| cg22044840 | 2 | 617 | 706 | 0 | 0 | 0 | 0 | 0 | 0 | 0 | 0 | 0 |
| cg22053855 | 2 | 617 | 706 | 0 | 0 | 0 | 0 | 0 | 0 | 0 | 0 | 0 |
| cg22069989 | 2 | 616 | 707 | 0 | 0 | 0 | 0 | 0 | 0 | 0 | 0 | 0 |
| cg22164912 | 3 | 611 | 5 | 707 | 0 | 0 | 0 | 0 | 0 | 0 | 0 | 0 |
| cg22221554 | 4 | 616 | 2 | 1 | 704 | 0 | 0 | 0 | 0 | 0 | 0 | 0 |
| cg22223600 | 2 | 615 | 708 | 0 | 0 | 0 | 0 | 0 | 0 | 0 | 0 | 0 |
| cg22275278 | 3 | 614 | 706 | 3 | 0 | 0 | 0 | 0 | 0 | 0 | 0 | 0 |
| cg22366618 | 3 | 612 | 710 | 1 | 0 | 0 | 0 | 0 | 0 | 0 | 0 | 0 |
| cg22412459 | 3 | 606 | 8 | 709 | 0 | 0 | 0 | 0 | 0 | 0 | 0 | 0 |
| cg22417589 | 3 | 617 | 705 | 1 | 0 | 0 | 0 | 0 | 0 | 0 | 0 | 0 |
| cg22449896 | 2 | 619 | 704 | 0 | 0 | 0 | 0 | 0 | 0 | 0 | 0 | 0 |
| cg22452543 | 5 | 610 | 3 | 2 | 706 | 2 | 0 | 0 | 0 | 0 | 0 | 0 |
| cg22561883 | 3 | 616 | 1 | 706 | 0 | 0 | 0 | 0 | 0 | 0 | 0 | 0 |
| cg22575892 | 3 | 618 | 704 | 1 | 0 | 0 | 0 | 0 | 0 | 0 | 0 | 0 |
| cg22662205 | 2 | 617 | 706 | 0 | 0 | 0 | 0 | 0 | 0 | 0 | 0 | 0 |
| cg22662834 | 4 | 608 | 6 | 2 | 707 | 0 | 0 | 0 | 0 | 0 | 0 | 0 |
| cg22674664 | 3 | 613 | 709 | 1 | 0 | 0 | 0 | 0 | 0 | 0 | 0 | 0 |
| cg22682304 | 3 | 616 | 706 | 1 | 0 | 0 | 0 | 0 | 0 | 0 | 0 | 0 |
| cg22682567 | 2 | 622 | 701 | 0 | 0 | 0 | 0 | 0 | 0 | 0 | 0 | 0 |
| cg22776211 | 2 | 616 | 707 | 0 | 0 | 0 | 0 | 0 | 0 | 0 | 0 | 0 |
| cg22807592 | 2 | 609 | 714 | 0 | 0 | 0 | 0 | 0 | 0 | 0 | 0 | 0 |
| cg22817647 | 2 | 616 | 707 | 0 | 0 | 0 | 0 | 0 | 0 | 0 | 0 | 0 |
| cg22823767 | 4 | 615 | 1 | 705 | 2 | 0 | 0 | 0 | 0 | 0 | 0 | 0 |
| cg22824415 | 2 | 618 | 705 | 0 | 0 | 0 | 0 | 0 | 0 | 0 | 0 | 0 |
| cg22917680 | 4 | 615 | 706 | 1 | 1 | 0 | 0 | 0 | 0 | 0 | 0 | 0 |
| cg22918218 | 2 | 616 | 707 | 0 | 0 | 0 | 0 | 0.00E+00 | 0 | 0 | 0 | 0 |
| cg22944282 | 2 | 618 | 705 | 0 | 0 | 0 | 0 | 0 | 0 | 0 | 0 | 0 |
| cg22951728 | 2 | 615 | 708 | 0 | 0 | 0 | 0 | 0 | 0 | 0 | 0 | 0 |
| cg22969661 | 2 | 617 | 706 | 0 | 0 | 0 | 0 | 0 | 0 | 0 | 0 | 0 |
| cg23064627 | 3 | 618 | 704 | 1 | 0 | 0 | 0 | 0 | 0 | 0 | 0 | 0 |
| cg23121114 | 2 | 616 | 707 | 0 | 0 | 0 | 0 | 0 | 0 | 0 | 0 | 0 |
| cg23124867 | 2 | 619 | 704 | 0 | 0 | 0 | 0 | 0 | 0 | 0 | 0 | 0 |
| cg23143524 | 3 | 609 | 7 | 707 | 0 | 0 | 0 | 0 | 0 | 0 | 0 | 0 |
| cg23154024 | 3 | 615 | 706 | 2 | 0 | 0 | 0 | 0 | 0 | 0 | 0 | 0 |
| cg23184276 | 2 | 617 | 706 | 0 | 0 | 0 | 0 | 0 | 0 | 0 | 0 | 0 |
| cg23214992 | 2 | 616 | 707 | 0 | 0 | 0 | 0 | 0 | 0 | 0 | 0 | 0 |
| cg23251359 | 3 | 616 | 1 | 706 | 0 | 0 | 0 | 0 | 0 | 0 | 0 | 0 |
| cg23299576 | 3 | 615 | 707 | 1 | 0 | 0 | 0 | 0 | 0 | 0 | 0 | 0 |
| cg23323276 | 2 | 615 | 708 | 0 | 0 | 0 | 0 | 0 | 0 | 0 | 0 | 0 |
| cg23323745 | 3 | 615 | 707 | 1 | 0 | 0 | 0 | 0 | 0 | 0 | 0 | 0 |
| cg23356769 | 3 | 618 | 704 | 1 | 0 | 0 | 0 | 0 | 0 | 0 | 0 | 0 |
| cg23408987 | 2 | 616 | 707 | 0 | 0 | 0 | 0 | 0 | 0 | 0 | 0 | 0 |
| cg23429746 | 2 | 616 | 707 | 0 | 0 | 0 | 0 | 0 | 0 | 0 | 0 | 0 |
| cg23443158 | 3 | 616 | 706 | 1 | 0 | 0 | 0 | 0 | 0 | 0 | 0 | 0 |
| cg23455407 | 3 | 617 | 1 | 705 | 0 | 0 | 0 | 0 | 0 | 0 | 0 | 0 |
| cg23475462 | 3 | 613 | 709 | 1 | 0 | 0 | 0 | 0 | 0 | 0 | 0 | 0 |
| cg23475921 | 2 | 615 | 708 | 0 | 0 | 0 | 0 | 0 | 0 | 0 | 0 | 0 |
| cg23484208 | 2 | 616 | 707 | 0 | 0 | 0 | 0 | 0 | 0 | 0 | 0 | 0 |
| cg23493872 | 3 | 612 | 8 | 703 | 0 | 0 | 0 | 0 | 0 | 0 | 0 | 0 |
| cg23503517 | 3 | 606 | 12 | 705 | 0 | 0 | 0 | 0 | 0 | 0 | 0 | 0 |
| cg23524524 | 3 | 615 | 707 | 1 | 0 | 0 | 0 | 0 | 0 | 0 | 0 | 0 |
| cg23543766 | 2 | 616 | 707 | 0 | 0 | 0 | 0 | 0 | 0 | 0 | 0 | 0 |
| cg23627980 | 3 | 618 | 704 | 1 | 0 | 0 | 0 | 0 | 0 | 0 | 0 | 0 |
| cg23685102 | 2 | 617 | 706 | 0 | 0 | 0 | 0 | 0 | 0 | 0 | 0 | 0 |
| cg23696472 | 4 | 607 | 9 | 3 | 704 | 0 | 0 | 0 | 0 | 0 | 0 | 0 |
| cg23712855 | 4 | 613 | 2 | 1 | 707 | 0 | 0 | 0.00E+00 | 0 | 0 | 0 | 0 |
| cg23724412 | 2 | 617 | 706 | 0 | 0 | 0 | 0 | 0 | 0 | 0 | 0 | 0 |
| cg23834765 | 2 | 618 | 705 | 0 | 0 | 0 | 0 | 0 | 0 | 0 | 0 | 0 |
| cg23852001 | 3 | 616 | 706 | 1 | 0 | 0 | 0 | 0 | 0 | 0 | 0 | 0 |
| cg23857909 | 2 | 616 | 707 | 0 | 0 | 0 | 0 | 0 | 0 | 0 | 0 | 0 |
| cg23896353 | 3 | 612 | 6 | 705 | 0 | 0 | 0 | 0 | 0 | 0 | 0 | 0 |
| cg23919845 | 2 | 620 | 703 | 0 | 0 | 0 | 0 | 0 | 0 | 0 | 0 | 0 |
| cg23936476 | 2 | 618 | 705 | 0 | 0 | 0 | 0 | 0 | 0 | 0 | 0 | 0 |
| cg23941728 | 2 | 611 | 712 | 0 | 0 | 0 | 0 | 0 | 0 | 0 | 0 | 0 |
| cg23951868 | 3 | 611 | 8 | 704 | 0 | 0 | 0 | 0 | 0 | 0 | 0 | 0 |
| cg23954206 | 2 | 616 | 707 | 0 | 0 | 0 | 0 | 0 | 0 | 0 | 0 | 0 |
| cg23990273 | 2 | 618 | 705 | 0 | 0 | 0 | 0 | 0 | 0 | 0 | 0 | 0 |
| cg24034992 | 3 | 615 | 706 | 2 | 0 | 0 | 0 | 0 | 0 | 0 | 0 | 0 |
| cg24035229 | 3 | 615 | 2 | 706 | 0 | 0 | 0 | 0 | 0 | 0 | 0 | 0 |
| cg24184541 | 2 | 615 | 708 | 0 | 0 | 0 | 0 | 0 | 0 | 0 | 0 | 0 |
| cg24186901 | 3 | 608 | 7 | 708 | 0 | 0 | 0 | 0 | 0 | 0 | 0 | 0 |
| cg24202706 | 4 | 614 | 3 | 705 | 1 | 0 | 0 | 0 | 0 | 0 | 0 | 0 |
| cg24264679 | 2 | 616 | 707 | 0 | 0 | 0 | 0 | 0 | 0 | 0 | 0 | 0 |
| cg24328927 | 2 | 617 | 706 | 0 | 0 | 0 | 0 | 0 | 0 | 0 | 0 | 0 |
| cg24341236 | 2 | 619 | 704 | 0 | 0 | 0 | 0 | 0 | 0 | 0 | 0 | 0 |
| cg24347720 | 3 | 617 | 705 | 1 | 0 | 0 | 0 | 0 | 0 | 0 | 0 | 0 |
| cg24512517 | 3 | 615 | 1 | 707 | 0 | 0 | 0 | 0 | 0 | 0 | 0 | 0 |
| cg24546622 | 3 | 619 | 702 | 2 | 0 | 0 | 0 | 0 | 0 | 0 | 0 | 0 |
| cg24559073 | 2 | 615 | 708 | 0 | 0 | 0 | 0 | 0 | 0 | 0 | 0 | 0 |
| cg24597825 | 2 | 619 | 704 | 0 | 0 | 0 | 0 | 0 | 0 | 0 | 0 | 0 |
| cg24627956 | 4 | 616 | 1 | 705 | 1 | 0 | 0 | 0 | 0 | 0 | 0 | 0 |
| cg24701794 | 2 | 616 | 707 | 0 | 0 | 0 | 0 | 0 | 0 | 0 | 0 | 0 |
| cg24721916 | 2 | 618 | 705 | 0 | 0 | 0 | 0 | 0 | 0 | 0 | 0 | 0 |
| cg24740922 | 2 | 608 | 715 | 0 | 0 | 0 | 0 | 0 | 0 | 0 | 0 | 0 |
| cg24741068 | 4 | 616 | 1 | 704 | 2 | 0 | 0 | 0 | 0 | 0 | 0 | 0 |
| cg24748621 | 2 | 608 | 715 | 0 | 0 | 0 | 0 | 0 | 0 | 0 | 0 | 0 |
| cg24783624 | 2 | 616 | 707 | 0 | 0 | 0 | 0 | 0 | 0 | 0 | 0 | 0 |
| cg24790801 | 3 | 615 | 1 | 707 | 0 | 0 | 0 | 0 | 0 | 0 | 0 | 0 |
| cg24818939 | 2 | 619 | 704 | 0 | 0 | 0 | 0 | 0 | 0 | 0 | 0 | 0 |
| cg24826812 | 2 | 619 | 704 | 0 | 0 | 0 | 0 | 0 | 0 | 0 | 0 | 0 |
| cg24832428 | 2 | 611 | 712 | 0 | 0 | 0 | 0 | 0 | 0 | 0 | 0 | 0 |
| cg24844608 | 3 | 618 | 704 | 1 | 0 | 0 | 0 | 0 | 0 | 0 | 0 | 0 |
| cg24863802 | 2 | 615 | 708 | 0 | 0 | 0 | 0 | 0 | 0 | 0 | 0 | 0 |
| cg24880787 | 2 | 616 | 707 | 0 | 0 | 0 | 0 | 0 | 0 | 0 | 0 | 0 |
| cg24925526 | 3 | 614 | 2 | 707 | 0 | 0 | 0 | 0 | 0 | 0 | 0 | 0 |
| cg25132467 | 3 | 609 | 2 | 712 | 0 | 0 | 0 | 0 | 0 | 0 | 0 | 0 |
| cg25156485 | 2 | 616 | 707 | 0 | 0 | 0 | 0 | 0 | 0 | 0 | 0 | 0 |
| cg25202270 | 4 | 615 | 1 | 705 | 2 | 0 | 0 | 0 | 0 | 0 | 0 | 0 |
| cg25205946 | 3 | 618 | 704 | 1 | 0 | 0 | 0 | 0 | 0 | 0 | 0 | 0 |
| cg25206026 | 3 | 616 | 1 | 706 | 0 | 0 | 0 | 0 | 0 | 0 | 0 | 0 |
| cg25225807 | 3 | 610 | 6 | 707 | 0 | 0 | 0 | 0 | 0 | 0 | 0 | 0 |
| cg25236484 | 3 | 617 | 705 | 1 | 0 | 0 | 0 | 0 | 0 | 0 | 0 | 0 |
| cg25258879 | 2 | 616 | 707 | 0 | 0 | 0 | 0 | 0 | 0 | 0 | 0 | 0 |
| cg25321762 | 2 | 619 | 704 | 0 | 0 | 0 | 0 | 0 | 0 | 0 | 0 | 0 |
| cg25415674 | 2 | 620 | 703 | 0 | 0 | 0 | 0 | 0 | 0 | 0 | 0 | 0 |
| cg25427918 | 4 | 611 | 3 | 4 | 705 | 0 | 0 | 0 | 0 | 0 | 0 | 0 |
| cg25499687 | 2 | 615 | 708 | 0 | 0 | 0 | 0 | 0 | 0 | 0 | 0 | 0 |
| cg25528646 | 3 | 616 | 706 | 1 | 0 | 0 | 0 | 0 | 0 | 0 | 0 | 0 |
| cg25571060 | 3 | 616 | 706 | 1 | 0 | 0 | 0 | 0 | 0 | 0 | 0 | 0 |
| cg25656978 | 4 | 607 | 7 | 2 | 707 | 0 | 0 | 0 | 0 | 0 | 0 | 0 |
| cg25698940 | 2 | 615 | 708 | 0 | 0 | 0 | 0 | 0 | 0 | 0 | 0 | 0 |
| cg25777540 | 2 | 615 | 708 | 0 | 0 | 0 | 0 | 0 | 0 | 0 | 0 | 0 |
| cg25777856 | 5 | 615 | 1 | 702 | 4 | 1 | 0 | 0 | 0 | 0 | 0 | 0 |
| cg25832410 | 3 | 609 | 713 | 1 | 0 | 0 | 0 | 0 | 0 | 0 | 0 | 0 |
| cg25858008 | 2 | 619 | 704 | 0 | 0 | 0 | 0 | 0 | 0 | 0 | 0 | 0 |
| cg25888700 | 4 | 615 | 1 | 706 | 1 | 0 | 0 | 0 | 0 | 0 | 0 | 0 |
| cg25897349 | 4 | 607 | 9 | 706 | 1 | 0 | 0 | 0 | 0 | 0 | 0 | 0 |
| cg25940844 | 3 | 615 | 1 | 707 | 0 | 0 | 0 | 0 | 0 | 0 | 0 | 0 |
| cg26023405 | 4 | 608 | 1 | 8 | 706 | 0 | 0 | 0 | 0 | 0 | 0 | 0 |
| cg26059639 | 3 | 616 | 2 | 705 | 0 | 0 | 0 | 0 | 0 | 0 | 0 | 0 |
| cg26146690 | 3 | 616 | 706 | 1 | 0 | 0 | 0 | 0 | 0 | 0 | 0 | 0 |
| cg26159385 | 3 | 615 | 707 | 1 | 0 | 0 | 0 | 0 | 0 | 0 | 0 | 0 |
| cg26234774 | 3 | 607 | 715 | 1 | 0 | 0 | 0 | 0 | 0 | 0 | 0 | 0 |
| cg26308359 | 2 | 617 | 706 | 0 | 0 | 0 | 0 | 0 | 0 | 0 | 0 | 0 |
| cg26333397 | 2 | 618 | 705 | 0 | 0 | 0 | 0 | 0 | 0 | 0 | 0 | 0 |
| cg26342575 | 3 | 618 | 703 | 2 | 0 | 0 | 0 | 0 | 0 | 0 | 0 | 0 |
| cg26380710 | 2 | 612 | 711 | 0 | 0 | 0 | 0 | 0 | 0 | 0 | 0 | 0 |
| cg26381742 | 3 | 616 | 1 | 706 | 0 | 0 | 0 | 0 | 0 | 0 | 0 | 0 |
| cg26382696 | 4 | 611 | 1 | 697 | 14 | 0 | 0 | 0 | 0 | 0 | 0 | 0 |
| cg26410404 | 2 | 615 | 708 | 0 | 0 | 0 | 0 | 0 | 0 | 0 | 0 | 0 |
| cg26418790 | 3 | 615 | 2 | 706 | 0 | 0 | 0 | 0 | 0 | 0 | 0 | 0 |
| cg26421947 | 3 | 616 | 706 | 1 | 0 | 0 | 0 | 0 | 0 | 0 | 0 | 0 |
| cg26428339 | 3 | 613 | 1 | 709 | 0 | 0 | 0 | 0 | 0 | 0 | 0 | 0 |
| cg26457165 | 2 | 615 | 708 | 0 | 0 | 0 | 0 | 0 | 0 | 0 | 0 | 0 |
| cg26505478 | 4 | 616 | 2 | 704 | 1 | 0 | 0 | 0 | 0 | 0 | 0 | 0 |
| cg26584339 | 4 | 604 | 7 | 8 | 704 | 0 | 0 | 0 | 0 | 0 | 0 | 0 |
| cg26628435 | 2 | 616 | 707 | 0 | 0 | 0 | 0 | 0 | 0 | 0 | 0 | 0 |
| cg26674826 | 3 | 613 | 707 | 3 | 0 | 0 | 0 | 0 | 0 | 0 | 0 | 0 |
| cg26682103 | 3 | 608 | 2 | 713 | 0 | 0 | 0 | 0 | 0 | 0 | 0 | 0 |
| cg26744454 | 2 | 618 | 705 | 0 | 0 | 0 | 0 | 0 | 0 | 0 | 0 | 0 |
| cg26746069 | 4 | 618 | 1 | 1 | 703 | 0 | 0 | 0.00E+00 | 0 | 0.00E+00 | 0 | 0 |
| cg26756979 | 2 | 616 | 707 | 0 | 0 | 0 | 0 | 0 | 0 | 0 | 0 | 0 |
| cg26764180 | 3 | 614 | 2 | 707 | 0 | 0 | 0 | 0 | 0 | 0 | 0 | 0 |
| cg26777746 | 3 | 617 | 705 | 1 | 0 | 0 | 0 | 0 | 0 | 0 | 0 | 0 |
| cg26777760 | 2 | 618 | 705 | 0 | 0 | 0 | 0 | 0 | 0 | 0 | 0 | 0 |
| cg26799772 | 2 | 615 | 708 | 0 | 0 | 0 | 0 | 0 | 0 | 0 | 0 | 0 |
| cg26896756 | 4 | 614 | 1 | 707 | 1 | 0 | 0 | 0 | 0 | 0 | 0 | 0 |
| cg26927606 | 4 | 618 | 703 | 1 | 1 | 0 | 0 | 0 | 0 | 0 | 0 | 0 |
| cg26951364 | 3 | 616 | 706 | 1 | 0 | 0 | 0 | 0 | 0 | 0 | 0 | 0 |
| cg27047283 | 2 | 611 | 712 | 0 | 0 | 0 | 0 | 0 | 0 | 0 | 0 | 0 |
| cg27059259 | 2 | 615 | 708 | 0 | 0 | 0 | 0 | 0 | 0 | 0 | 0 | 0 |
| cg27062326 | 3 | 617 | 705 | 1 | 0 | 0 | 0 | 0 | 0 | 0 | 0 | 0 |
| cg27069132 | 3 | 611 | 5 | 707 | 0 | 0 | 0 | 0 | 0 | 0 | 0 | 0 |
| cg27083627 | 2 | 609 | 714 | 0 | 0 | 0 | 0 | 0 | 0 | 0 | 0 | 0 |
| cg27123903 | 6 | 606 | 3 | 4 | 1 | 2 | 707 | 0 | 0 | 0 | 0 | 0 |
| cg27124847 | 4 | 611 | 1 | 709 | 2 | 0 | 0 | 0 | 0 | 0 | 0 | 0 |
| cg27207144 | 4 | 611 | 5 | 2 | 705 | 0 | 0 | 0 | 0 | 0 | 0 | 0 |
| cg27268405 | 2 | 616 | 707 | 0 | 0 | 0 | 0 | 0 | 0 | 0 | 0 | 0 |
| cg27296089 | 3 | 616 | 706 | 1 | 0 | 0 | 0 | 0 | 0 | 0 | 0 | 0 |
| cg27327591 | 2 | 616 | 707 | 0 | 0 | 0 | 0 | 0 | 0 | 0 | 0 | 0 |
| cg27470278 | 2 | 617 | 706 | 0 | 0 | 0 | 0 | 0 | 0 | 0 | 0 | 0 |
| cg27474516 | 2 | 613 | 710 | 0 | 0 | 0 | 0 | 0 | 0 | 0 | 0 | 0 |
| cg27496708 | 2 | 616 | 707 | 0 | 0 | 0 | 0 | 0 | 0 | 0 | 0 | 0 |
| cg27501007 | 3 | 616 | 706 | 1 | 0 | 0 | 0 | 0 | 0 | 0 | 0 | 0 |
| cg27501723 | 2 | 617 | 706 | 0 | 0 | 0 | 0 | 0 | 0 | 0 | 0 | 0 |
| cg27515272 | 3 | 614 | 705 | 4 | 0 | 0 | 0 | 0 | 0 | 0 | 0 | 0 |
| cg27609596 | 2 | 615 | 708 | 0 | 0 | 0 | 0 | 0 | 0 | 0 | 0 | 0 |
| cg27616996 | 3 | 615 | 1 | 707 | 0 | 0 | 0 | 0 | 0 | 0 | 0 | 0 |
| cg27665489 | 2 | 614 | 709 | 0 | 0 | 0 | 0 | 0 | 0 | 0 | 0 | 0 |
| cg00999163 | 3 | 802 | 4 | 517 | 0 | 0 | 0 | 0 | 0 | 0 | 0 | 0 |
| cg02155655 | 3 | 1 | 77 | 1245 | 0 | 0 | 0 | 0 | 0 | 0 | 0 | 0 |
| cg02368820 | 3 | 650 | 1 | 672 | 0 | 0 | 0 | 0 | 0 | 0 | 0 | 0 |
| cg04154653 | 2 | 103 | 1220 | 0 | 0 | 0 | 0 | 0 | 0 | 0 | 0 | 0 |
| cg05392448 | 4 | 89 | 1 | 4 | 1229 | 0 | 0 | 0 | 0 | 0 | 0 | 0 |
| cg08055132 | 4 | 97 | 1 | 2 | 1223 | 0 | 0 | 0 | 0 | 0 | 0 | 0 |
| cg08146708 | 3 | 241 | 1 | 1081 | 0 | 0 | 0 | 0 | 0 | 0 | 0 | 0 |
| cg11418303 | 2 | 117 | 1206 | 0 | 0 | 0 | 0 | 0 | 0 | 0 | 0 | 0 |
| cg12417704 | 2 | 90 | 1233 | 0 | 0 | 0 | 0 | 0 | 0 | 0 | 0 | 0 |
| cg12770003 | 4 | 6 | 90 | 1 | 1226 | 0 | 0 | 0 | 0 | 0 | 0 | 0 |
| cg12814070 | 3 | 1222 | 100 | 1 | 0 | 0 | 0 | 0 | 0 | 0 | 0 | 0 |
| cg13064658 | 3 | 1120 | 3 | 200 | 0 | 0 | 0 | 0 | 0 | 0 | 0 | 0 |
| cg13491296 | 4 | 1253 | 68 | 1 | 1 | 0 | 0 | 0 | 0 | 0 | 0 | 0 |
| cg13926174 | 2 | 134 | 1189 | 0 | 0 | 0 | 0 | 0 | 0 | 0 | 0 | 0 |
| cg15209419 | 3 | 11 | 86 | 1226 | 0 | 0 | 0 | 0 | 0 | 0 | 0 | 0 |
| cg16162930 | 2 | 235 | 1088 | 0 | 0 | 0 | 0 | 0 | 0 | 0 | 0 | 0 |
| cg16682227 | 4 | 32 | 1253 | 2 | 36 | 0 | 0 | 0 | 0 | 0 | 0 | 0 |
| cg19629430 | 3 | 117 | 1 | 1205 | 0 | 0 | 0 | 0 | 0 | 0 | 0 | 0 |
| cg19712277 | 3 | 246 | 3 | 1074 | 0 | 0 | 0 | 0 | 0 | 0 | 0 | 0 |
| cg20697066 | 3 | 4 | 89 | 1230 | 0 | 0 | 0 | 0 | 0 | 0 | 0 | 0 |
| cg20828284 | 5 | 17 | 2 | 183 | 1 | 1120 | 0 | 0 | 0 | 0 | 0 | 0 |
| cg21272897 | 3 | 727 | 1 | 595 | 0 | 0 | 0 | 0 | 0 | 0 | 0 | 0 |
| cg22337407 | 3 | 128 | 430 | 765 | 0 | 0 | 0 | 0 | 0 | 0 | 0 | 0 |
| cg26027669 | 4 | 1243 | 1 | 1 | 78 | 0 | 0 | 0 | 0 | 0 | 0 | 0 |
| cg05385718 | 4 | 67 | 458 | 1 | 797 | 0 | 0 | 0 | 0 | 0 | 0 | 0 |
| cg05570109 | 3 | 145 | 559 | 619 | 0 | 0 | 0 | 0 | 0 | 0 | 0 | 0 |
| cg06193597 | 4 | 415 | 2 | 2 | 904 | 0 | 0 | 0 | 0 | 0 | 0 | 0 |
| cg06545389 | 2 | 1173 | 150 | 0 | 0 | 0 | 0 | 0 | 0 | 0 | 0 | 0 |
| cg06634576 | 4 | 196 | 1124 | 1 | 2 | 0 | 0 | 0 | 0 | 0 | 0 | 0 |
| cg12814117 | 4 | 271 | 1 | 1 | 1050 | 0 | 0 | 0 | 0 | 0 | 0 | 0 |
| cg14111685 | 2 | 167 | 1156 | 0 | 0 | 0 | 0 | 0 | 0 | 0 | 0 | 0 |
| cg14181874 | 4 | 3 | 199 | 10 | 1111 | 0 | 0 | 0 | 0 | 0 | 0 | 0 |
| cg15998127 | 4 | 3 | 149 | 3 | 1168 | 0 | 0 | 0 | 0 | 0 | 0 | 0 |
| cg19961153 | 4 | 199 | 1 | 1 | 1122 | 0 | 0 | 0 | 0 | 0 | 0 | 0 |
| cg22972806 | 3 | 152 | 2 | 1169 | 0 | 0 | 0 | 0 | 0 | 0 | 0 | 0 |
| cg23491743 | 4 | 39 | 57 | 1 | 1226 | 0 | 0 | 0 | 0 | 0 | 0 | 0 |
| cg24007926 | 5 | 510 | 4 | 1 | 2 | 806 | 0 | 0 | 0 | 0 | 0 | 0 |
| cg26075039 | 4 | 4 | 1 | 82 | 1236 | 0 | 0 | 0 | 0 | 0 | 0 | 0 |
| cg21114725 | 2 | 153 | 1170 | 0 | 0 | 0 | 0 | 0 | 0 | 0 | 0 | 0 |
| cg22986662 | 3 | 206 | 1 | 1116 | 0 | 0 | 0 | 0 | 0 | 0 | 0 | 0 |
| cg06794034 | 3 | 5 | 92 | 1226 | 0 | 0 | 0 | 0 | 0 | 0 | 0 | 0 |
| cg08823359 | 3 | 1039 | 283 | 1 | 0 | 0 | 0 | 0 | 0 | 0 | 0 | 0 |
| cg11294620 | 3 | 2 | 107 | 1214 | 0 | 0 | 0 | 0 | 0 | 0 | 0 | 0 |
| cg14115431 | 2 | 166 | 1157 | 0 | 0 | 0 | 0 | 0 | 0 | 0 | 0 | 0 |
| cg19151808 | 4 | 42 | 1237 | 2 | 42 | 0 | 0 | 0 | 0 | 0 | 0 | 0 |
| cg20702875 | 4 | 35 | 1 | 276 | 1011 | 0 | 0 | 0 | 0 | 0 | 0 | 0 |
| cg01063413 | 5 | 13 | 134 | 1 | 1 | 1174 | 0 | 0 | 0 | 0 | 0 | 0 |
| cg03871140 | 4 | 840 | 1 | 412 | 70 | 0 | 0 | 0 | 0 | 0 | 0 | 0 |
| cg05656210 | 4 | 311 | 1 | 1 | 1010 | 0 | 0 | 0 | 0 | 0 | 0 | 0 |
| cg10202835 | 4 | 252 | 2 | 1068 | 1 | 0 | 0 | 0 | 0 | 0 | 0 | 0 |
| cg11282828 | 3 | 96 | 1 | 1226 | 0 | 0 | 0 | 0 | 0 | 0 | 0 | 0 |
| cg11524184 | 4 | 2 | 3 | 68 | 1250 | 0 | 0 | 0 | 0 | 0 | 0 | 0 |
| cg14061270 | 3 | 264 | 1 | 1058 | 0 | 0 | 0 | 0 | 0 | 0 | 0 | 0 |
| cg14708411 | 3 | 1 | 248 | 1074 | 0 | 0 | 0 | 0 | 0 | 0 | 0 | 0 |
| cg18666982 | 3 | 6 | 82 | 1235 | 0 | 0 | 0 | 0 | 0 | 0 | 0 | 0 |
| cg18816122 | 3 | 356 | 1 | 966 | 0 | 0 | 0 | 0 | 0 | 0 | 0 | 0 |
| cg25366315 | 4 | 73 | 3 | 5 | 1242 | 0 | 0 | 0 | 0 | 0 | 0 | 0 |
| cg00023507 | 2 | 209 | 1114 | 0 | 0 | 0 | 0 | 0 | 0 | 0 | 0 | 0 |
| cg02246922 | 3 | 170 | 1 | 1152 | 0 | 0 | 0 | 0 | 0 | 0 | 0 | 0 |
| cg09703840 | 4 | 92 | 2 | 428 | 801 | 0 | 0 | 0 | 0 | 0 | 0 | 0 |
| cg10167891 | 3 | 88 | 3 | 1232 | 0 | 0 | 0 | 0 | 0 | 0 | 0 | 0 |
| cg10983013 | 2 | 148 | 1175 | 0 | 0 | 0 | 0 | 0 | 0 | 0 | 0 | 0 |
| cg11437465 | 5 | 219 | 1 | 1 | 1 | 1101 | 0 | 0 | 0 | 0 | 0 | 0 |
| cg14645244 | 2 | 393 | 930 | 0 | 0 | 0 | 0 | 0 | 0 | 0 | 0 | 0 |
| cg15991478 | 2 | 100 | 1223 | 0 | 0 | 0 | 0 | 0 | 0 | 0 | 0 | 0 |
| cg17929630 | 4 | 49 | 1 | 396 | 877 | 0 | 0 | 0 | 0 | 0 | 0 | 0 |
| cg18110333 | 3 | 241 | 1081 | 1 | 0 | 0 | 0 | 0 | 0 | 0 | 0 | 0 |
| cg19475903 | 4 | 23 | 277 | 1 | 1022 | 0 | 0 | 0 | 0 | 0 | 0 | 0 |
| cg20332088 | 3 | 16 | 205 | 1102 | 0 | 0 | 0 | 0 | 0 | 0 | 0 | 0 |
| cg21526238 | 3 | 69 | 1 | 1253 | 0 | 0 | 0 | 0 | 0 | 0 | 0 | 0 |
| cg27414868 | 3 | 122 | 7 | 1194 | 0 | 0 | 0 | 0 | 0 | 0 | 0 | 0 |
| cg06950937 | 3 | 237 | 1 | 1085 | 0 | 0 | 0 | 0 | 0 | 0 | 0 | 0 |
| cg07315018 | 3 | 71 | 1251 | 1 | 0 | 0 | 0 | 0 | 0 | 0 | 0 | 0 |
| cg09727210 | 4 | 74 | 1 | 424 | 824 | 0 | 0 | 0 | 0 | 0 | 0 | 0 |
| cg11769349 | 2 | 185 | 1138 | 0 | 0 | 0 | 0 | 0 | 0 | 0 | 0 | 0 |
| cg13160627 | 3 | 136 | 1 | 1186 | 0 | 0 | 0 | 0 | 0 | 0 | 0 | 0 |
| cg13703663 | 3 | 17 | 245 | 1061 | 0 | 0 | 0 | 0 | 0 | 0 | 0 | 0 |
| cg15464485 | 2 | 120 | 1203 | 0 | 0 | 0 | 0 | 0 | 0 | 0 | 0 | 0 |
| cg15574437 | 3 | 155 | 4 | 1164 | 0 | 0 | 0 | 0 | 0 | 0 | 0 | 0 |
| cg15935227 | 2 | 129 | 1194 | 0 | 0 | 0 | 0 | 0 | 0 | 0 | 0 | 0 |
| cg16178271 | 5 | 1 | 142 | 1 | 1 | 1178 | 0 | 0 | 0 | 0 | 0 | 0 |
| cg16542356 | 4 | 138 | 2 | 5 | 1178 | 0 | 0 | 0 | 0 | 0 | 0 | 0 |
| cg17714861 | 5 | 19 | 2 | 1 | 109 | 1192 | 0 | 0 | 0 | 0 | 0 | 0 |
| cg18404925 | 5 | 1254 | 1 | 1 | 65 | 2 | 0 | 0 | 0 | 0 | 0 | 0 |
| cg20550012 | 3 | 329 | 1 | 993 | 0 | 0 | 0 | 0 | 0 | 0 | 0 | 0 |
| cg21015022 | 2 | 513 | 810 | 0 | 0 | 0 | 0 | 0 | 0 | 0 | 0 | 0 |
| cg21885112 | 2 | 123 | 1200 | 0 | 0 | 0 | 0 | 0 | 0 | 0 | 0 | 0 |
| cg22109827 | 2 | 259 | 1064 | 0 | 0 | 0 | 0 | 0 | 0 | 0 | 0 | 0 |
| cg24185656 | 4 | 20 | 276 | 1 | 1026 | 0 | 0 | 0 | 0 | 0 | 0 | 0 |
| cg26075639 | 2 | 1256 | 67 | 0 | 0 | 0 | 0 | 0 | 0 | 0 | 0 | 0 |
| cg27468880 | 2 | 204 | 1119 | 0 | 0 | 0 | 0 | 0 | 0 | 0 | 0 | 0 |
| cg01652021 | 4 | 66 | 1 | 1 | 1255 | 0 | 0 | 0 | 0 | 0 | 0 | 0 |
| cg02118671 | 4 | 132 | 1 | 1189 | 1 | 0 | 0 | 0 | 0 | 0 | 0 | 0 |
| cg04124606 | 2 | 1249 | 74 | 0 | 0 | 0 | 0 | 0 | 0 | 0 | 0 | 0 |
| cg05891136 | 2 | 77 | 1246 | 0 | 0 | 0 | 0 | 0 | 0 | 0 | 0 | 0 |
| cg06002687 | 4 | 359 | 2 | 605 | 357 | 0 | 0 | 0 | 0 | 0 | 0 | 0 |
| cg11124135 | 3 | 101 | 1 | 1221 | 0 | 0 | 0 | 0 | 0 | 0 | 0 | 0 |
| cg11205696 | 2 | 253 | 1070 | 0 | 0 | 0 | 0 | 0 | 0 | 0 | 0 | 0 |
| cg15251140 | 2 | 604 | 719 | 0 | 0 | 0 | 0 | 0 | 0 | 0 | 0 | 0 |
| cg16120147 | 4 | 170 | 1 | 647 | 505 | 0 | 0 | 0 | 0 | 0 | 0 | 0 |
| cg17373649 | 8 | 1 | 2 | 117 | 1 | 1 | 1 | 1 | 1199 | 0 | 0 | 0 |
| cg17527589 | 2 | 139 | 1184 | 0 | 0 | 0 | 0 | 0 | 0 | 0 | 0 | 0 |
| cg18339359 | 2 | 285 | 1038 | 0 | 0 | 0 | 0 | 0 | 0 | 0 | 0 | 0 |
| cg19787013 | 3 | 208 | 2 | 1113 | 0 | 0 | 0 | 0 | 0 | 0 | 0 | 0 |
| cg24634471 | 2 | 349 | 974 | 0 | 0 | 0 | 0 | 0 | 0 | 0 | 0 | 0 |
| cg25614253 | 2 | 755 | 568 | 0 | 0 | 0 | 0 | 0 | 0 | 0 | 0 | 0 |
| cg26400491 | 4 | 2 | 1 | 77 | 1243 | 0 | 0 | 0 | 0 | 0 | 0 | 0 |
| cg13568515 | 4 | 1 | 1 | 71 | 1250 | 0 | 0 | 0 | 0 | 0 | 0 | 0 |
| cg13672736 | 3 | 69 | 2 | 1252 | 0 | 0 | 0 | 0 | 0 | 0 | 0 | 0 |
| cg13963044 | 2 | 68 | 1255 | 0 | 0 | 0 | 0 | 0 | 0 | 0 | 0 | 0 |
| cg14368972 | 5 | 10 | 1 | 162 | 4 | 1146 | 0 | 0 | 0 | 0 | 0 | 0 |
| cg21211688 | 5 | 378 | 2 | 2 | 1 | 940 | 0 | 0 | 0 | 0 | 0 | 0 |
| cg00117869 | 2 | 90 | 1233 | 0 | 0 | 0 | 0 | 0 | 0 | 0 | 0 | 0 |
| cg02309026 | 2 | 162 | 1161 | 0 | 0 | 0 | 0 | 0 | 0 | 0 | 0 | 0 |
| cg02583163 | 3 | 76 | 2 | 1245 | 0 | 0 | 0 | 0 | 0 | 0 | 0 | 0 |
| cg07629625 | 2 | 295 | 1028 | 0 | 0 | 0 | 0 | 0 | 0 | 0 | 0 | 0 |
| cg07684215 | 2 | 312 | 1011 | 0 | 0 | 0 | 0 | 0 | 0 | 0 | 0 | 0 |
| cg12129080 | 2 | 621 | 702 | 0 | 0 | 0 | 0 | 0 | 0 | 0 | 0 | 0 |
| cg12196389 | 2 | 133 | 1190 | 0 | 0 | 0 | 0 | 0 | 0 | 0 | 0 | 0 |
| cg12682323 | 2 | 225 | 1098 | 0 | 0 | 0 | 0 | 0 | 0 | 0 | 0 | 0 |
| cg14464852 | 3 | 3 | 151 | 1169 | 0 | 0 | 0 | 0 | 0 | 0 | 0 | 0 |
| cg19126377 | 2 | 1240 | 83 | 0 | 0 | 0 | 0 | 0 | 0 | 0 | 0 | 0 |
| cg19754622 | 2 | 1197 | 126 | 0 | 0 | 0 | 0 | 0 | 0 | 0 | 0 | 0 |
| cg24284539 | 5 | 345 | 1 | 1 | 1 | 975 | 0 | 0 | 0 | 0 | 0 | 0 |
| cg24320034 | 2 | 154 | 1169 | 0 | 0 | 0 | 0 | 0 | 0 | 0 | 0 | 0 |
| cg10528424 | 7 | 206 | 1 | 2 | 2 | 558 | 1 | 553 | 0 | 0 | 0 | 0 |
| cg11008123 | 3 | 95 | 505 | 723 | 0 | 0 | 0 | 0 | 0 | 0 | 0 | 0 |
| cg13262159 | 3 | 8 | 117 | 1198 | 0 | 0 | 0 | 0 | 0 | 0 | 0 | 0 |
| cg16788050 | 2 | 180 | 1143 | 0 | 0 | 0 | 0 | 0 | 0 | 0 | 0 | 0 |
| cg26753908 | 3 | 1 | 229 | 1093 | 0 | 0 | 0 | 0 | 0 | 0 | 0 | 0 |
| cg05929129 | 2 | 337 | 986 | 0 | 0 | 0 | 0 | 0 | 0 | 0 | 0 | 0 |
| cg10240950 | 2 | 100 | 1223 | 0 | 0 | 0 | 0 | 0 | 0 | 0 | 0 | 0 |
| cg10617763 | 3 | 72 | 1 | 1250 | 0 | 0 | 0 | 0 | 0 | 0 | 0 | 0 |
| cg10966873 | 4 | 5 | 1 | 76 | 1241 | 0 | 0 | 0 | 0 | 0 | 0 | 0 |
| cg15360451 | 6 | 123 | 1 | 1 | 2 | 454 | 742 | 0 | 0 | 0 | 0 | 0 |
| cg18232235 | 2 | 557 | 766 | 0 | 0 | 0 | 0 | 0 | 0 | 0 | 0 | 0 |
| cg19191272 | 3 | 17 | 237 | 1069 | 0 | 0 | 0 | 0 | 0 | 0 | 0 | 0 |
| cg24784730 | 4 | 1216 | 94 | 1 | 12 | 0 | 0 | 0 | 0 | 0 | 0 | 0 |
| cg00587941 | 3 | 1 | 134 | 1188 | 0 | 0 | 0 | 0 | 0 | 0 | 0 | 0 |
| cg03040740 | 4 | 180 | 3 | 1 | 1139 | 0 | 0 | 0 | 0 | 0 | 0 | 0 |
| cg06398883 | 2 | 87 | 1236 | 0 | 0 | 0 | 0 | 0 | 0 | 0 | 0 | 0 |
| cg07223206 | 3 | 81 | 2 | 1240 | 0 | 0 | 0 | 0 | 0 | 0 | 0 | 0 |
| cg12532878 | 3 | 79 | 3 | 1241 | 0 | 0 | 0 | 0 | 0 | 0 | 0 | 0 |
| cg25871816 | 3 | 1 | 70 | 1252 | 0 | 0 | 0 | 0 | 0 | 0 | 0 | 0 |
| cg04955116 | 4 | 155 | 1 | 1166 | 1 | 0 | 0 | 0 | 0 | 0 | 0 | 0 |
| cg05859760 | 3 | 114 | 1 | 1208 | 0 | 0 | 0 | 0 | 0 | 0 | 0 | 0 |
| cg13749548 | 2 | 560 | 763 | 0 | 0 | 0 | 0 | 0 | 0 | 0 | 0 | 0 |
| cg16802892 | 2 | 644 | 679 | 0 | 0 | 0 | 0 | 0 | 0 | 0 | 0 | 0 |
| cg23489384 | 4 | 344 | 630 | 1 | 348 | 0 | 0 | 0 | 0 | 0 | 0 | 0 |
| cg24976563 | 2 | 182 | 1141 | 0 | 0 | 0 | 0 | 0 | 0 | 0 | 0 | 0 |
| cg26031613 | 2 | 1223 | 100 | 0 | 0 | 0 | 0 | 0 | 0 | 0 | 0 | 0 |
| cg26258120 | 4 | 161 | 1 | 1 | 1160 | 0 | 0 | 0 | 0 | 0 | 0 | 0 |
| cg03449867 | 4 | 316 | 3 | 1 | 1003 | 0 | 0 | 0 | 0 | 0 | 0 | 0 |
| cg06753055 | 3 | 75 | 1 | 1247 | 0 | 0 | 0 | 0 | 0 | 0 | 0 | 0 |
| cg08296868 | 5 | 5 | 3 | 1 | 96 | 1218 | 0 | 0 | 0 | 0 | 0 | 0 |
| cg09803413 | 3 | 8 | 78 | 1237 | 0 | 0 | 0 | 0 | 0 | 0 | 0 | 0 |
| cg09827761 | 3 | 1089 | 223 | 11 | 0 | 0 | 0 | 0 | 0 | 0 | 0 | 0 |
| cg16398051 | 2 | 239 | 1084 | 0 | 0 | 0 | 0 | 0 | 0 | 0 | 0 | 0 |
| cg16775095 | 3 | 137 | 1 | 1185 | 0 | 0 | 0 | 0 | 0 | 0 | 0 | 0 |
| cg19169023 | 3 | 3 | 378 | 942 | 0 | 0 | 0 | 0 | 0 | 0 | 0 | 0 |
| cg21829038 | 3 | 1 | 219 | 1103 | 0 | 0 | 0 | 0 | 0 | 0 | 0 | 0 |
| cg00324979 | 3 | 1 | 253 | 1069 | 0 | 0 | 0 | 0 | 0 | 0 | 0 | 0 |
| cg01454815 | 4 | 1250 | 1 | 71 | 1 | 0 | 0 | 0 | 0 | 0 | 0 | 0 |
| cg01741056 | 6 | 62 | 4 | 1 | 1 | 1 | 1254 | 0 | 0 | 0 | 0 | 0 |
| cg02043600 | 3 | 1256 | 1 | 66 | 0 | 0 | 0 | 0 | 0 | 0 | 0 | 0 |
| cg05477582 | 3 | 163 | 496 | 664 | 0 | 0 | 0 | 0 | 0 | 0 | 0 | 0 |
| cg06048169 | 2 | 77 | 1246 | 0 | 0 | 0 | 0 | 0 | 0 | 0 | 0 | 0 |
| cg06352616 | 2 | 294 | 1029 | 0 | 0 | 0 | 0 | 0 | 0 | 0 | 0 | 0 |
| cg08335008 | 4 | 9 | 1 | 133 | 1180 | 0 | 0 | 0 | 0 | 0 | 0 | 0 |
| cg08928871 | 3 | 2 | 100 | 1221 | 0 | 0 | 0 | 0 | 0 | 0 | 0 | 0 |
| cg10516012 | 2 | 76 | 1247 | 0 | 0 | 0 | 0 | 0 | 0 | 0 | 0 | 0 |
| cg02067491 | 3 | 9 | 78 | 1236 | 0 | 0 | 0 | 0 | 0 | 0 | 0 | 0 |
| cg02159489 | 4 | 271 | 3 | 2 | 1047 | 0 | 0 | 0 | 0 | 0 | 0 | 0 |
| cg02459042 | 4 | 69 | 1 | 1 | 1252 | 0 | 0 | 0 | 0 | 0 | 0 | 0 |
| cg02823329 | 3 | 96 | 445 | 782 | 0 | 0 | 0 | 0 | 0 | 0 | 0 | 0 |
| cg03407524 | 3 | 332 | 1 | 990 | 0 | 0 | 0 | 0 | 0 | 0 | 0 | 0 |
| cg05248234 | 2 | 719 | 604 | 0 | 0 | 0 | 0 | 0 | 0 | 0 | 0 | 0 |
| cg05291429 | 4 | 179 | 1 | 1 | 1142 | 0 | 0 | 0 | 0 | 0 | 0 | 0 |
| cg07712165 | 3 | 251 | 5 | 1067 | 0 | 0 | 0 | 0 | 0 | 0 | 0 | 0 |
| cg08900396 | 3 | 145 | 3 | 1175 | 0 | 0 | 0 | 0 | 0 | 0 | 0 | 0 |
| cg09951588 | 4 | 33 | 1 | 154 | 1135 | 0 | 0 | 0 | 0 | 0 | 0 | 0 |
| cg11606607 | 4 | 95 | 3 | 1 | 1224 | 0 | 0 | 0 | 0 | 0 | 0 | 0 |
| cg13164195 | 4 | 3 | 2 | 92 | 1226 | 0 | 0 | 0 | 0 | 0 | 0 | 0 |
| cg15250633 | 3 | 85 | 3 | 1235 | 0 | 0 | 0 | 0 | 0 | 0 | 0 | 0 |
| cg15880245 | 3 | 130 | 3 | 1190 | 0 | 0 | 0 | 0 | 0 | 0 | 0 | 0 |
| cg16464924 | 2 | 519 | 804 | 0 | 0 | 0 | 0 | 0 | 0 | 0 | 0 | 0 |
| cg17628491 | 3 | 100 | 489 | 734 | 0 | 0 | 0 | 0 | 0 | 0 | 0 | 0 |
| cg17737776 | 4 | 4 | 3 | 111 | 1205 | 0 | 0 | 0 | 0 | 0 | 0 | 0 |
| cg17906851 | 2 | 169 | 1154 | 0 | 0 | 0 | 0 | 0 | 0 | 0 | 0 | 0 |
| cg22968622 | 4 | 1077 | 1 | 1 | 244 | 0 | 0 | 0 | 0 | 0 | 0 | 0 |
| cg23676314 | 3 | 26 | 278 | 1019 | 0 | 0 | 0 | 0 | 0 | 0 | 0 | 0 |
| cg24439623 | 2 | 80 | 1243 | 0 | 0 | 0 | 0 | 0 | 0 | 0 | 0 | 0 |
| cg24636332 | 2 | 102 | 1221 | 0 | 0 | 0 | 0 | 0 | 0 | 0 | 0 | 0 |
| cg26480039 | 4 | 1 | 1 | 97 | 1224 | 0 | 0 | 0 | 0 | 0 | 0 | 0 |
| cg21848624 | 4 | 134 | 3 | 2 | 1184 | 0 | 0 | 0 | 0 | 0 | 0 | 0 |
| cg00843105 | 2 | 1173 | 150 | 0 | 0 | 0 | 0 | 0 | 0 | 0 | 0 | 0 |
| cg01270299 | 2 | 251 | 1072 | 0 | 0 | 0 | 0 | 0.00E+00 | 0 | 0 | 0 | 0 |
| cg01647917 | 6 | 1 | 203 | 2 | 1 | 2 | 1114 | 0 | 0 | 0 | 0 | 0 |
| cg04748098 | 2 | 115 | 1208 | 0 | 0 | 0 | 0 | 0 | 0 | 0 | 0 | 0 |
| cg07515196 | 4 | 1245 | 2 | 75 | 1 | 0 | 0 | 0 | 0 | 0 | 0 | 0 |
| cg11738485 | 4 | 767 | 1 | 457 | 98 | 0 | 0 | 0 | 0 | 0 | 0 | 0 |
| cg17313483 | 3 | 1248 | 1 | 74 | 0 | 0 | 0 | 0 | 0 | 0 | 0 | 0 |
| cg18105999 | 2 | 70 | 1253 | 0 | 0 | 0 | 0 | 0 | 0 | 0 | 0 | 0 |
| cg18634314 | 4 | 1206 | 113 | 1 | 3 | 0 | 0 | 0 | 0 | 0 | 0 | 0 |
| cg20242379 | 4 | 71 | 10 | 3 | 1239 | 0 | 0 | 0 | 0 | 0 | 0 | 0 |
| cg20242889 | 3 | 891 | 1 | 431 | 0 | 0 | 0 | 0 | 0 | 0 | 0 | 0 |
| cg22531183 | 3 | 1180 | 132 | 11 | 0 | 0 | 0 | 0 | 0 | 0 | 0 | 0 |
| cg24181266 | 4 | 1248 | 73 | 1 | 1 | 0 | 0 | 0 | 0 | 0 | 0 | 0 |
| cg24413826 | 3 | 19 | 215 | 1089 | 0 | 0 | 0 | 0 | 0 | 0 | 0 | 0 |
| cg11495604 | 4 | 1 | 2 | 177 | 1143 | 0 | 0 | 0 | 0 | 0 | 0 | 0 |
| cg11979743 | 4 | 1253 | 1 | 2 | 67 | 0 | 0 | 0 | 0 | 0 | 0 | 0 |
| cg16310958 | 3 | 409 | 1 | 913 | 0 | 0 | 0 | 0 | 0 | 0 | 0 | 0 |
| cg22588144 | 3 | 1100 | 1 | 222 | 0 | 0 | 0 | 0 | 0 | 0 | 0 | 0 |
| cg23574719 | 3 | 156 | 4 | 1163 | 0 | 0 | 0 | 0 | 0 | 0 | 0 | 0 |
| cg04574724 | 4 | 16 | 101 | 2 | 1204 | 0 | 0 | 0 | 0 | 0 | 0 | 0 |
| cg19245335 | 2 | 215 | 1108 | 0 | 0 | 0 | 0 | 0 | 0 | 0 | 0 | 0 |
| cg21139150 | 2 | 1100 | 223 | 0 | 0 | 0 | 0 | 0 | 0 | 0 | 0 | 0 |
| cg00801716 | 3 | 2 | 90 | 1231 | 0 | 0 | 0 | 0 | 0 | 0 | 0 | 0 |
| cg07918799 | 4 | 2 | 3 | 63 | 1255 | 0 | 0 | 0 | 0 | 0 | 0 | 0 |
| cg14112997 | 3 | 722 | 2 | 599 | 0 | 0 | 0 | 0 | 0 | 0 | 0 | 0 |
| cg22487450 | 3 | 23 | 159 | 1141 | 0 | 0 | 0 | 0 | 0 | 0 | 0 | 0 |
| cg01086462 | 4 | 706 | 5 | 611 | 1 | 0 | 0 | 0 | 0 | 0 | 0 | 0 |
| cg03278611 | 2 | 719 | 604 | 0 | 0 | 0 | 0 | 0 | 0 | 0 | 0 | 0 |
| cg03905640 | 2 | 685 | 638 | 0 | 0 | 0 | 0 | 0 | 0 | 0 | 0 | 0 |
| cg05940236 | 2 | 1250 | 73 | 0 | 0 | 0 | 0 | 0 | 0 | 0 | 0 | 0 |
| cg09197443 | 2 | 124 | 1199 | 0 | 0 | 0 | 0 | 0 | 0 | 0 | 0 | 0 |
| cg10422744 | 3 | 708 | 613 | 2 | 0 | 0 | 0 | 0 | 0 | 0 | 0 | 0 |
| cg11898347 | 2 | 1180 | 143 | 0 | 0 | 0 | 0 | 0 | 0 | 0 | 0 | 0 |
| cg00046099 | 3 | 613 | 3 | 707 | 0 | 0 | 0 | 0 | 0 | 0 | 0 | 0 |
| cg00113623 | 2 | 611 | 712 | 0 | 0 | 0 | 0 | 0 | 0 | 0 | 0 | 0 |
| cg00115511 | 3 | 614 | 708 | 1 | 0 | 0 | 0 | 0 | 0 | 0 | 0 | 0 |
| cg00139547 | 4 | 615 | 1 | 705 | 2 | 0 | 0 | 0 | 0 | 0 | 0 | 0 |
| cg00151234 | 2 | 615 | 708 | 0 | 0 | 0 | 0 | 0 | 0 | 0 | 0 | 0 |
| cg00227584 | 2 | 615 | 708 | 0 | 0 | 0 | 0 | 0 | 0 | 0 | 0 | 0 |
| cg00253811 | 2 | 616 | 707 | 0 | 0 | 0 | 0 | 0 | 0 | 0 | 0 | 0 |
| cg00337921 | 3 | 613 | 8 | 702 | 0 | 0 | 0 | 0 | 0 | 0 | 0 | 0 |
| cg00347850 | 2 | 615 | 708 | 0 | 0 | 0 | 0 | 0 | 0 | 0 | 0 | 0 |
| cg00368230 | 2 | 616 | 707 | 0 | 0 | 0 | 0 | 0 | 0 | 0 | 0 | 0 |
| cg00433220 | 3 | 615 | 2 | 706 | 0 | 0 | 0 | 0 | 0 | 0 | 0 | 0 |
| cg00581583 | 2 | 620 | 703 | 0 | 0 | 0 | 0 | 0 | 0 | 0 | 0 | 0 |
| cg00603890 | 3 | 614 | 3 | 706 | 0 | 0 | 0 | 0 | 0 | 0 | 0 | 0 |
| cg00618396 | 3 | 620 | 702 | 1 | 0 | 0 | 0 | 0 | 0 | 0 | 0 | 0 |
| cg00632374 | 2 | 617 | 706 | 0 | 0 | 0 | 0 | 0.00E+00 | 0 | 0 | 0 | 0 |
| cg00634642 | 2 | 618 | 705 | 0 | 0 | 0 | 0 | 0 | 0 | 0 | 0 | 0 |
| cg00667781 | 2 | 619 | 704 | 0 | 0 | 0 | 0 | 0 | 0 | 0 | 0 | 0 |
| cg00790086 | 2 | 618 | 705 | 0 | 0 | 0 | 0 | 0 | 0 | 0 | 0 | 0 |
| cg00823357 | 3 | 611 | 710 | 2 | 0 | 0 | 0 | 0 | 0 | 0 | 0 | 0 |
| cg00917018 | 2 | 615 | 708 | 0 | 0 | 0 | 0 | 0 | 0 | 0 | 0 | 0 |
| cg00937263 | 2 | 618 | 705 | 0 | 0 | 0 | 0 | 0 | 0 | 0 | 0 | 0 |
| cg00940232 | 2 | 616 | 707 | 0 | 0 | 0 | 0 | 0 | 0 | 0 | 0 | 0 |
| cg00963467 | 2 | 616 | 707 | 0 | 0 | 0 | 0 | 0 | 0 | 0 | 0 | 0 |
| cg01003197 | 2 | 617 | 706 | 0 | 0 | 0 | 0 | 0 | 0 | 0 | 0 | 0 |
| cg01019600 | 2 | 615 | 708 | 0 | 0 | 0 | 0 | 0 | 0 | 0 | 0 | 0 |
| cg01039990 | 2 | 616 | 707 | 0 | 0 | 0 | 0 | 0 | 0 | 0 | 0 | 0 |
| cg01166930 | 3 | 616 | 706 | 1 | 0 | 0 | 0 | 0 | 0 | 0 | 0 | 0 |
| cg01225298 | 2 | 616 | 707 | 0 | 0 | 0 | 0 | 0 | 0 | 0 | 0 | 0 |
| cg01241836 | 3 | 618 | 704 | 1 | 0 | 0 | 0 | 0 | 0 | 0 | 0 | 0 |
| cg01286388 | 3 | 616 | 706 | 1 | 0 | 0 | 0 | 0 | 0 | 0 | 0 | 0 |
| cg01345087 | 3 | 615 | 707 | 1 | 0 | 0 | 0 | 0 | 0 | 0 | 0 | 0 |
| cg01384686 | 2 | 607 | 716 | 0 | 0 | 0 | 0 | 0 | 0 | 0 | 0 | 0 |
| cg01398912 | 3 | 616 | 1 | 706 | 0 | 0 | 0 | 0 | 0 | 0 | 0 | 0 |
| cg01408383 | 2 | 619 | 704 | 0 | 0 | 0 | 0 | 0 | 0 | 0 | 0 | 0 |
| cg01445307 | 2 | 618 | 705 | 0 | 0 | 0 | 0 | 0 | 0 | 0 | 0 | 0 |
| cg01472026 | 2 | 614 | 709 | 0 | 0 | 0 | 0 | 0 | 0 | 0 | 0 | 0 |
| cg01530521 | 3 | 613 | 3 | 707 | 0 | 0 | 0 | 0 | 0 | 0 | 0 | 0 |
| cg01556010 | 2 | 619 | 704 | 0 | 0 | 0 | 0 | 0 | 0 | 0 | 0 | 0 |
| cg01579322 | 4 | 608 | 10 | 704 | 1 | 0 | 0 | 0 | 0 | 0 | 0 | 0 |
| cg01683788 | 2 | 616 | 707 | 0 | 0 | 0 | 0 | 0 | 0 | 0 | 0 | 0 |
| cg01716666 | 2 | 618 | 705 | 0 | 0 | 0 | 0 | 0 | 0 | 0 | 0 | 0 |
| cg01733439 | 2 | 618 | 705 | 0 | 0 | 0 | 0 | 0 | 0 | 0 | 0 | 0 |
| cg01740135 | 5 | 398 | 1 | 1 | 2 | 921 | 0 | 0 | 0 | 0 | 0 | 0 |
| cg01756638 | 2 | 618 | 705 | 0 | 0 | 0 | 0 | 0 | 0 | 0 | 0 | 0 |
| cg01771673 | 2 | 616 | 707 | 0 | 0 | 0 | 0 | 0 | 0 | 0 | 0 | 0 |
| cg01780361 | 3 | 616 | 2 | 705 | 0 | 0 | 0 | 0 | 0 | 0 | 0 | 0 |
| cg01855997 | 2 | 618 | 705 | 0 | 0 | 0 | 0 | 0 | 0 | 0 | 0 | 0 |
| cg01868753 | 2 | 613 | 710 | 0 | 0 | 0 | 0 | 0 | 0 | 0 | 0 | 0 |
| cg01910452 | 3 | 616 | 705 | 2 | 0 | 0 | 0 | 0 | 0 | 0 | 0 | 0 |
| cg02025065 | 2 | 619 | 704 | 0 | 0 | 0 | 0 | 0 | 0 | 0 | 0 | 0 |
| cg02148711 | 2 | 616 | 707 | 0 | 0 | 0 | 0 | 0 | 0 | 0 | 0 | 0 |
| cg02161824 | 3 | 616 | 706 | 1 | 0 | 0 | 0 | 0 | 0 | 0 | 0 | 0 |
| cg02165720 | 2 | 616 | 707 | 0 | 0 | 0 | 0 | 0 | 0 | 0 | 0 | 0 |
| cg02264182 | 2 | 610 | 713 | 0 | 0 | 0 | 0 | 0 | 0 | 0 | 0 | 0 |
| cg02264284 | 3 | 622 | 700 | 1 | 0 | 0 | 0 | 0 | 0 | 0 | 0 | 0 |
| cg02301364 | 3 | 617 | 705 | 1 | 0 | 0 | 0 | 0 | 0 | 0 | 0 | 0 |
| cg02456261 | 2 | 614 | 709 | 0 | 0 | 0 | 0 | 0 | 0 | 0 | 0 | 0 |
| cg02634916 | 3 | 616 | 2 | 705 | 0 | 0 | 0 | 0 | 0 | 0 | 0 | 0 |
| cg02649608 | 3 | 618 | 703 | 2 | 0 | 0 | 0 | 0 | 0 | 0 | 0 | 0 |
| cg02805922 | 3 | 617 | 705 | 1 | 0 | 0 | 0 | 0 | 0 | 0 | 0 | 0 |
| cg02856792 | 2 | 615 | 708 | 0 | 0 | 0 | 0 | 0 | 0 | 0 | 0 | 0 |
| cg02871887 | 4 | 614 | 2 | 3 | 704 | 0 | 0 | 0 | 0 | 0 | 0 | 0 |
| cg02882301 | 2 | 615 | 708 | 0 | 0 | 0 | 0 | 0 | 0 | 0 | 0 | 0 |
| cg02896496 | 3 | 616 | 1 | 706 | 0 | 0 | 0 | 0 | 0 | 0 | 0 | 0 |
| cg02902015 | 2 | 617 | 706 | 0 | 0 | 0 | 0 | 0 | 0 | 0 | 0 | 0 |
| cg03019024 | 2 | 609 | 714 | 0 | 0 | 0 | 0 | 0 | 0 | 0 | 0 | 0 |
| cg03040210 | 4 | 616 | 1 | 705 | 1 | 0 | 0 | 0 | 0 | 0 | 0 | 0 |
| cg03050491 | 2 | 616 | 707 | 0 | 0 | 0 | 0 | 0 | 0 | 0 | 0 | 0 |
| cg03057808 | 2 | 616 | 707 | 0 | 0 | 0 | 0 | 0 | 0 | 0 | 0 | 0 |
| cg03181214 | 2 | 616 | 707 | 0 | 0 | 0 | 0 | 0 | 0 | 0 | 0 | 0 |
| cg03273606 | 2 | 614 | 709 | 0 | 0 | 0 | 0 | 0 | 0 | 0 | 0 | 0 |
| cg03317455 | 2 | 616 | 707 | 0 | 0 | 0 | 0 | 0 | 0 | 0 | 0 | 0 |
| cg03372815 | 2 | 616 | 707 | 0 | 0 | 0 | 0 | 0 | 0 | 0 | 0 | 0 |
| cg03424637 | 2 | 619 | 704 | 0 | 0 | 0 | 0 | 0 | 0 | 0 | 0 | 0 |
| cg03487706 | 3 | 613 | 3 | 707 | 0 | 0 | 0 | 0 | 0 | 0 | 0 | 0 |
| cg03537243 | 2 | 617 | 706 | 0 | 0 | 0 | 0 | 0 | 0 | 0 | 0 | 0 |
| cg03576039 | 2 | 616 | 707 | 0 | 0 | 0 | 0 | 0 | 0 | 0 | 0 | 0 |
| cg03610137 | 3 | 616 | 706 | 1 | 0 | 0 | 0 | 0 | 0 | 0 | 0 | 0 |
| cg03672021 | 3 | 615 | 2 | 706 | 0 | 0 | 0 | 0 | 0 | 0 | 0 | 0 |
| cg03732411 | 2 | 616 | 707 | 0 | 0 | 0 | 0 | 0 | 0 | 0 | 0 | 0 |
| cg03773146 | 3 | 603 | 11 | 709 | 0 | 0 | 0 | 0 | 0 | 0 | 0 | 0 |
| cg03834574 | 2 | 618 | 705 | 0 | 0 | 0 | 0 | 0 | 0 | 0 | 0 | 0 |
| cg03878985 | 3 | 617 | 705 | 1 | 0 | 0 | 0 | 0 | 0 | 0 | 0 | 0 |
| cg03885028 | 6 | 179 | 2 | 2 | 240 | 3 | 897 | 0 | 0 | 0 | 0 | 0 |
| cg03946442 | 2 | 618 | 705 | 0 | 0 | 0 | 0 | 0 | 0 | 0 | 0 | 0 |
| cg04070122 | 3 | 618 | 704 | 1 | 0 | 0 | 0 | 0 | 0 | 0 | 0 | 0 |
| cg04158739 | 2 | 615 | 708 | 0 | 0 | 0 | 0 | 0 | 0 | 0 | 0 | 0 |
| cg04238548 | 4 | 616 | 705 | 1 | 1 | 0 | 0 | 0 | 0 | 0 | 0 | 0 |
| cg04493740 | 3 | 616 | 706 | 1 | 0 | 0 | 0 | 0 | 0 | 0 | 0 | 0 |
| cg04514385 | 4 | 1 | 1 | 69 | 1252 | 0 | 0 | 0 | 0 | 0 | 0 | 0 |
| cg04575501 | 2 | 616 | 707 | 0 | 0 | 0 | 0 | 0 | 0 | 0 | 0 | 0 |
| cg04586456 | 2 | 616 | 707 | 0 | 0 | 0 | 0 | 0 | 0 | 0 | 0 | 0 |
| cg04596655 | 2 | 615 | 708 | 0 | 0 | 0 | 0 | 0 | 0 | 0 | 0 | 0 |
| cg04681171 | 3 | 615 | 3 | 705 | 0 | 0 | 0 | 0 | 0 | 0 | 0 | 0 |
| cg04703500 | 3 | 616 | 705 | 2 | 0 | 0 | 0 | 0 | 0 | 0 | 0 | 0 |
| cg04769268 | 2 | 612 | 711 | 0 | 0 | 0 | 0 | 0 | 0 | 0 | 0 | 0 |
| cg04828704 | 2 | 615 | 708 | 0 | 0 | 0 | 0 | 0 | 0 | 0 | 0 | 0 |
| cg04907664 | 2 | 615 | 708 | 0 | 0 | 0 | 0 | 0 | 0 | 0 | 0 | 0 |
| cg04944936 | 2 | 618 | 705 | 0 | 0 | 0 | 0 | 0 | 0 | 0 | 0 | 0 |
| cg05034175 | 3 | 615 | 1 | 707 | 0 | 0 | 0 | 0 | 0 | 0 | 0 | 0 |
| cg05045738 | 3 | 618 | 1 | 704 | 0 | 0 | 0 | 0 | 0 | 0 | 0 | 0 |
| cg05073171 | 3 | 619 | 703 | 1 | 0 | 0 | 0 | 0 | 0 | 0 | 0 | 0 |
| cg05100268 | 2 | 613 | 710 | 0 | 0 | 0 | 0 | 0 | 0 | 0 | 0 | 0 |
| cg05116966 | 3 | 614 | 2 | 707 | 0 | 0 | 0 | 0 | 0 | 0 | 0 | 0 |
| cg05130312 | 3 | 1 | 708 | 614 | 0 | 0 | 0 | 0 | 0 | 0 | 0 | 0 |
| cg05204037 | 2 | 616 | 707 | 0 | 0 | 0 | 0 | 0 | 0 | 0 | 0 | 0 |
| cg05257947 | 2 | 607 | 716 | 0 | 0 | 0 | 0 | 0 | 0 | 0 | 0 | 0 |
| cg05275343 | 2 | 615 | 708 | 0 | 0 | 0 | 0 | 0 | 0 | 0 | 0 | 0 |
| cg05418443 | 2 | 616 | 707 | 0 | 0 | 0 | 0 | 0 | 0 | 0 | 0 | 0 |
| cg05419227 | 2 | 619 | 704 | 0 | 0 | 0 | 0 | 0 | 0 | 0 | 0 | 0 |
| cg05427163 | 2 | 620 | 703 | 0 | 0 | 0 | 0 | 0 | 0 | 0 | 0 | 0 |
| cg05554396 | 3 | 618 | 703 | 2 | 0 | 0 | 0 | 0 | 0 | 0 | 0 | 0 |
| cg05673346 | 2 | 618 | 705 | 0 | 0 | 0 | 0 | 0 | 0 | 0 | 0 | 0 |
| cg05688478 | 2 | 617 | 706 | 0 | 0 | 0 | 0 | 0 | 0 | 0 | 0 | 0 |
| cg05707815 | 2 | 616 | 707 | 0 | 0 | 0 | 0 | 0 | 0 | 0 | 0 | 0 |
| cg05837905 | 2 | 618 | 705 | 0 | 0 | 0 | 0 | 0 | 0 | 0 | 0 | 0 |
| cg05872215 | 2 | 618 | 705 | 0 | 0 | 0 | 0 | 0 | 0 | 0 | 0 | 0 |
| cg05872808 | 2 | 620 | 703 | 0 | 0 | 0 | 0 | 0 | 0 | 0 | 0 | 0 |
| cg05886698 | 3 | 30 | 87 | 1206 | 0 | 0 | 0 | 0 | 0 | 0 | 0 | 0 |
| cg05889642 | 2 | 616 | 707 | 0 | 0 | 0 | 0 | 0 | 0 | 0 | 0 | 0 |
| cg05911704 | 2 | 612 | 711 | 0 | 0 | 0 | 0 | 0 | 0 | 0 | 0 | 0 |
| cg05961595 | 2 | 619 | 704 | 0 | 0 | 0 | 0 | 0 | 0 | 0 | 0 | 0 |
| cg06055266 | 2 | 616 | 707 | 0 | 0 | 0 | 0 | 0 | 0 | 0 | 0 | 0 |
| cg06149454 | 2 | 619 | 704 | 0 | 0 | 0 | 0 | 0 | 0 | 0 | 0 | 0 |
| cg06341336 | 3 | 615 | 2 | 706 | 0 | 0 | 0 | 0 | 0 | 0 | 0 | 0 |
| cg06363801 | 2 | 615 | 708 | 0 | 0 | 0 | 0 | 0 | 0 | 0 | 0 | 0 |
| cg06438901 | 3 | 616 | 2 | 705 | 0 | 0 | 0 | 0 | 0 | 0 | 0 | 0 |
| cg06475150 | 2 | 616 | 707 | 0 | 0 | 0 | 0 | 0 | 0 | 0 | 0 | 0 |
| cg06481089 | 2 | 616 | 707 | 0 | 0 | 0 | 0 | 0 | 0 | 0 | 0 | 0 |
| cg06482328 | 3 | 615 | 707 | 1 | 0 | 0 | 0 | 0 | 0 | 0 | 0 | 0 |
| cg06513359 | 2 | 618 | 705 | 0 | 0 | 0 | 0 | 0 | 0 | 0 | 0 | 0 |
| cg06549249 | 2 | 616 | 707 | 0 | 0 | 0 | 0 | 0 | 0 | 0 | 0 | 0 |
| cg06558952 | 4 | 615 | 1 | 705 | 2 | 0 | 0 | 0 | 0 | 0 | 0 | 0 |
| cg06617418 | 3 | 21 | 78 | 1224 | 0 | 0 | 0 | 0 | 0 | 0 | 0 | 0 |
| cg06620254 | 3 | 622 | 700 | 1 | 0 | 0 | 0 | 0 | 0 | 0 | 0 | 0 |
| cg06645047 | 4 | 617 | 704 | 1 | 1 | 0 | 0 | 0 | 0 | 0 | 0 | 0 |
| cg06772067 | 3 | 618 | 703 | 2 | 0 | 0 | 0 | 0 | 0 | 0 | 0 | 0 |
| cg06822229 | 3 | 616 | 2 | 705 | 0 | 0 | 0 | 0 | 0 | 0 | 0 | 0 |
| cg06908232 | 3 | 615 | 707 | 1 | 0 | 0 | 0 | 0 | 0 | 0 | 0 | 0 |
| cg06942445 | 2 | 616 | 707 | 0 | 0 | 0 | 0 | 0 | 0 | 0 | 0 | 0 |
| cg06971254 | 3 | 613 | 3 | 707 | 0 | 0 | 0 | 0 | 0 | 0 | 0 | 0 |
| cg07150680 | 3 | 616 | 3 | 704 | 0 | 0 | 0 | 0 | 0 | 0 | 0 | 0 |
| cg07256221 | 2 | 617 | 706 | 0 | 0 | 0 | 0 | 0 | 0 | 0 | 0 | 0 |
| cg07264097 | 2 | 618 | 705 | 0 | 0 | 0 | 0 | 0 | 0 | 0 | 0 | 0 |
| cg07342016 | 2 | 617 | 706 | 0 | 0 | 0 | 0 | 0 | 0 | 0 | 0 | 0 |
| cg07507339 | 2 | 616 | 707 | 0 | 0 | 0 | 0 | 0 | 0 | 0 | 0 | 0 |
| cg07629996 | 2 | 616 | 707 | 0 | 0 | 0 | 0 | 0 | 0 | 0 | 0 | 0 |
| cg07649160 | 2 | 616 | 707 | 0 | 0 | 0 | 0 | 0 | 0 | 0 | 0 | 0 |
| cg07799491 | 3 | 618 | 704 | 1 | 0 | 0 | 0 | 0 | 0 | 0 | 0 | 0 |
| cg07863318 | 2 | 616 | 707 | 0 | 0 | 0 | 0 | 0 | 0 | 0 | 0 | 0 |
| cg07946630 | 3 | 613 | 4 | 706 | 0 | 0 | 0 | 0 | 0 | 0 | 0 | 0 |
| cg08065501 | 3 | 619 | 1 | 703 | 0 | 0 | 0 | 0 | 0 | 0 | 0 | 0 |
| cg08118032 | 2 | 619 | 704 | 0 | 0 | 0 | 0 | 0 | 0 | 0 | 0 | 0 |
| cg08221357 | 3 | 614 | 708 | 1 | 0 | 0 | 0 | 0 | 0 | 0 | 0 | 0 |
| cg08239458 | 3 | 614 | 2 | 707 | 0 | 0 | 0 | 0 | 0 | 0 | 0 | 0 |
| cg08275242 | 2 | 615 | 708 | 0 | 0 | 0 | 0 | 0 | 0 | 0 | 0 | 0 |
| cg08327960 | 2 | 614 | 709 | 0 | 0 | 0 | 0 | 0 | 0 | 0 | 0 | 0 |
| cg08338641 | 3 | 616 | 1 | 706 | 0 | 0 | 0 | 0 | 0 | 0 | 0 | 0 |
| cg08395108 | 2 | 616 | 707 | 0 | 0 | 0 | 0 | 0 | 0 | 0 | 0 | 0 |
| cg08461617 | 3 | 620 | 701 | 2 | 0 | 0 | 0 | 0 | 0 | 0 | 0 | 0 |
| cg08560117 | 3 | 616 | 1 | 706 | 0 | 0 | 0 | 0 | 0 | 0 | 0 | 0 |
| cg08656747 | 2 | 616 | 707 | 0 | 0 | 0 | 0 | 0 | 0 | 0 | 0 | 0 |
| cg08663085 | 3 | 617 | 704 | 2 | 0 | 0 | 0 | 0 | 0 | 0 | 0 | 0 |
| cg08673814 | 2 | 615 | 708 | 0 | 0 | 0 | 0 | 0 | 0 | 0 | 0 | 0 |
| cg08695223 | 2 | 608 | 715 | 0 | 0 | 0 | 0 | 0 | 0 | 0 | 0 | 0 |
| cg08782677 | 2 | 616 | 707 | 0 | 0 | 0 | 0 | 0 | 0 | 0 | 0 | 0 |
| cg08785133 | 2 | 618 | 705 | 0 | 0 | 0 | 0 | 0 | 0 | 0 | 0 | 0 |
| cg08855111 | 3 | 606 | 10 | 707 | 0 | 0 | 0 | 0 | 0 | 0 | 0 | 0 |
| cg08955859 | 2 | 618 | 705 | 0 | 0 | 0 | 0 | 0 | 0 | 0 | 0 | 0 |
| cg08960045 | 3 | 92 | 1 | 1230 | 0 | 0 | 0 | 0 | 0 | 0 | 0 | 0 |
| cg08983668 | 3 | 624 | 697 | 2 | 0 | 0 | 0 | 0 | 0 | 0 | 0 | 0 |
| cg08991015 | 2 | 621 | 702 | 0 | 0 | 0 | 0 | 0 | 0 | 0 | 0 | 0 |
| cg09100792 | 2 | 621 | 702 | 0 | 0 | 0 | 0 | 0 | 0 | 0 | 0 | 0 |
| cg09186478 | 3 | 614 | 708 | 1 | 0 | 0 | 0 | 0 | 0 | 0 | 0 | 0 |
| cg09202373 | 2 | 610 | 713 | 0 | 0 | 0 | 0 | 0 | 0 | 0 | 0 | 0 |
| cg09347151 | 2 | 615 | 708 | 0 | 0 | 0 | 0 | 0 | 0 | 0 | 0 | 0 |
| cg09443457 | 2 | 617 | 706 | 0 | 0 | 0 | 0 | 0 | 0 | 0 | 0 | 0 |
| cg09598844 | 3 | 617 | 705 | 1 | 0 | 0 | 0 | 0 | 0 | 0 | 0 | 0 |
| cg09720420 | 2 | 616 | 707 | 0 | 0 | 0 | 0 | 0 | 0 | 0 | 0 | 0 |
| cg09725439 | 3 | 614 | 4 | 705 | 0 | 0 | 0 | 0 | 0 | 0 | 0 | 0 |
| cg09735782 | 3 | 614 | 5 | 704 | 0 | 0 | 0 | 0 | 0 | 0 | 0 | 0 |
| cg09909750 | 2 | 616 | 707 | 0 | 0 | 0 | 0 | 0 | 0 | 0 | 0 | 0 |
| cg09966745 | 2 | 613 | 710 | 0 | 0 | 0 | 0 | 0 | 0 | 0 | 0 | 0 |
| cg10016783 | 3 | 615 | 706 | 2 | 0 | 0 | 0 | 0 | 0 | 0 | 0 | 0 |
| cg10030436 | 2 | 619 | 704 | 0 | 0 | 0 | 0 | 0 | 0 | 0 | 0 | 0 |
| cg10101479 | 2 | 618 | 705 | 0 | 0 | 0 | 0 | 0 | 0 | 0 | 0 | 0 |
| cg10174816 | 2 | 616 | 707 | 0 | 0 | 0 | 0 | 0 | 0 | 0 | 0 | 0 |
| cg10277365 | 2 | 618 | 705 | 0 | 0 | 0 | 0 | 0 | 0 | 0 | 0 | 0 |
| cg10310824 | 2 | 619 | 704 | 0 | 0 | 0 | 0 | 0 | 0 | 0 | 0 | 0 |
| cg10401803 | 2 | 617 | 706 | 0 | 0 | 0 | 0 | 0 | 0 | 0 | 0 | 0 |
| cg10403109 | 2 | 617 | 706 | 0 | 0 | 0 | 0 | 0 | 0 | 0 | 0 | 0 |
| cg10426500 | 2 | 612 | 711 | 0 | 0 | 0 | 0 | 0 | 0 | 0 | 0 | 0 |
| cg10560144 | 3 | 615 | 707 | 1 | 0 | 0 | 0 | 0 | 0 | 0 | 0 | 0 |
| cg10581449 | 2 | 618 | 705 | 0 | 0 | 0 | 0 | 0 | 0 | 0 | 0 | 0 |
| cg10649042 | 3 | 618 | 704 | 1 | 0 | 0 | 0 | 0 | 0 | 0 | 0 | 0 |
| cg10906138 | 2 | 617 | 706 | 0 | 0 | 0 | 0 | 0 | 0 | 0 | 0 | 0 |
| cg10981178 | 2 | 615 | 708 | 0 | 0 | 0 | 0 | 0 | 0 | 0 | 0 | 0 |
| cg11038686 | 3 | 620 | 702 | 1 | 0 | 0 | 0 | 0 | 0 | 0 | 0 | 0 |
| cg11165479 | 3 | 615 | 707 | 1 | 0 | 0 | 0 | 0 | 0 | 0 | 0 | 0 |
| cg11253356 | 2 | 620 | 703 | 0 | 0 | 0 | 0 | 0 | 0 | 0 | 0 | 0 |
| cg11264711 | 3 | 617 | 705 | 1 | 0 | 0 | 0 | 0 | 0 | 0 | 0 | 0 |
| cg11320810 | 2 | 615 | 708 | 0 | 0 | 0 | 0 | 0 | 0 | 0 | 0 | 0 |
| cg11369993 | 3 | 617 | 705 | 1 | 0 | 0 | 0 | 0 | 0 | 0 | 0 | 0 |
| cg11609126 | 2 | 616 | 707 | 0 | 0 | 0 | 0 | 0 | 0 | 0 | 0 | 0 |
| cg11661234 | 2 | 618 | 705 | 0 | 0 | 0 | 0 | 0 | 0 | 0 | 0 | 0 |
| cg11717280 | 3 | 5 | 704 | 614 | 0 | 0 | 0 | 0 | 0 | 0 | 0 | 0 |
| cg11727174 | 2 | 617 | 706 | 0 | 0 | 0 | 0 | 0 | 0 | 0 | 0 | 0 |
| cg11870452 | 2 | 608 | 715 | 0 | 0 | 0 | 0 | 0 | 0 | 0 | 0 | 0 |
| cg12074916 | 2 | 616 | 707 | 0 | 0 | 0 | 0 | 0 | 0 | 0 | 0 | 0 |
| cg12158214 | 4 | 612 | 2 | 708 | 1 | 0 | 0 | 0 | 0 | 0 | 0 | 0 |
| cg12171481 | 3 | 617 | 705 | 1 | 0 | 0 | 0 | 0 | 0 | 0 | 0 | 0 |
| cg12217778 | 2 | 617 | 706 | 0 | 0 | 0 | 0 | 0 | 0 | 0 | 0 | 0 |
| cg12291192 | 2 | 619 | 704 | 0 | 0 | 0 | 0 | 0 | 0 | 0 | 0 | 0 |
| cg12308243 | 2 | 616 | 707 | 0 | 0 | 0 | 0 | 0 | 0 | 0 | 0 | 0 |
| cg12403148 | 2 | 619 | 704 | 0 | 0 | 0 | 0 | 0 | 0 | 0 | 0 | 0 |
| cg12443477 | 3 | 614 | 707 | 2 | 0 | 0 | 0 | 0 | 0 | 0 | 0 | 0 |
| cg12454975 | 2 | 617 | 706 | 0 | 0 | 0 | 0 | 0 | 0 | 0 | 0 | 0 |
| cg12464216 | 2 | 621 | 702 | 0 | 0 | 0 | 0 | 0 | 0 | 0 | 0 | 0 |
| cg12517167 | 3 | 616 | 706 | 1 | 0 | 0 | 0 | 0 | 0 | 0 | 0 | 0 |
| cg12543338 | 2 | 621 | 702 | 0 | 0 | 0 | 0 | 0 | 0 | 0 | 0 | 0 |
| cg12576145 | 3 | 614 | 3 | 706 | 0 | 0 | 0 | 0 | 0 | 0 | 0 | 0 |
| cg12620265 | 2 | 614 | 709 | 0 | 0 | 0 | 0 | 0 | 0 | 0 | 0 | 0 |
| cg12687215 | 3 | 616 | 706 | 1 | 0 | 0 | 0 | 0 | 0 | 0 | 0 | 0 |
| cg12755246 | 3 | 617 | 705 | 1 | 0 | 0 | 0 | 0 | 0 | 0 | 0 | 0 |
| cg12857702 | 2 | 617 | 706 | 0 | 0 | 0 | 0 | 0 | 0 | 0 | 0 | 0 |
| cg12942133 | 2 | 617 | 706 | 0 | 0 | 0 | 0 | 0 | 0 | 0 | 0 | 0 |
| cg12981362 | 2 | 616 | 707 | 0 | 0 | 0 | 0 | 0 | 0 | 0 | 0 | 0 |
| cg13016553 | 2 | 616 | 707 | 0 | 0 | 0 | 0 | 0 | 0 | 0 | 0 | 0 |
| cg13077484 | 2 | 615 | 708 | 0 | 0 | 0 | 0 | 0 | 0 | 0 | 0 | 0 |
| cg13156574 | 2 | 617 | 706 | 0 | 0 | 0 | 0 | 0 | 0 | 0 | 0 | 0 |
| cg13177658 | 5 | 611 | 708 | 1 | 1 | 2 | 0 | 0 | 0 | 0 | 0 | 0 |
| cg13183496 | 2 | 616 | 707 | 0 | 0 | 0 | 0 | 0 | 0 | 0 | 0 | 0 |
| cg13243544 | 4 | 617 | 1 | 704 | 1 | 0 | 0 | 0 | 0 | 0 | 0 | 0 |
| cg13286902 | 3 | 619 | 703 | 1 | 0 | 0 | 0 | 0 | 0 | 0 | 0 | 0 |
| cg13318368 | 2 | 615 | 708 | 0 | 0 | 0 | 0 | 0 | 0 | 0 | 0 | 0 |
| cg13516940 | 3 | 615 | 2 | 706 | 0 | 0 | 0 | 0 | 0 | 0 | 0 | 0 |
| cg13645300 | 2 | 620 | 703 | 0 | 0 | 0 | 0 | 0 | 0 | 0 | 0 | 0 |
| cg13655660 | 2 | 615 | 708 | 0 | 0 | 0 | 0 | 0 | 0 | 0 | 0 | 0 |
| cg13658777 | 2 | 619 | 704 | 0 | 0 | 0 | 0 | 0 | 0 | 0 | 0 | 0 |
| cg13726724 | 3 | 618 | 704 | 1 | 0 | 0 | 0 | 0 | 0 | 0 | 0 | 0 |
| cg13741548 | 2 | 622 | 701 | 0 | 0 | 0 | 0 | 0 | 0 | 0 | 0 | 0 |
| cg13768026 | 2 | 613 | 710 | 0 | 0 | 0 | 0 | 0 | 0 | 0 | 0 | 0 |
| cg13818628 | 3 | 617 | 705 | 1 | 0 | 0 | 0 | 0 | 0 | 0 | 0 | 0 |
| cg13828067 | 2 | 619 | 704 | 0 | 0 | 0 | 0 | 0 | 0 | 0 | 0 | 0 |
| cg14006678 | 2 | 620 | 703 | 0 | 0 | 0 | 0 | 0 | 0 | 0 | 0 | 0 |
| cg14098973 | 3 | 616 | 4 | 703 | 0 | 0 | 0 | 0 | 0 | 0 | 0 | 0 |
| cg14258356 | 3 | 616 | 704 | 3 | 0 | 0 | 0 | 0 | 0 | 0 | 0 | 0 |
| cg14260918 | 2 | 620 | 703 | 0 | 0 | 0 | 0 | 0 | 0 | 0 | 0 | 0 |
| cg14314132 | 3 | 613 | 709 | 1 | 0 | 0 | 0 | 0 | 0 | 0 | 0 | 0 |
| cg14314451 | 2 | 616 | 707 | 0 | 0 | 0 | 0 | 0 | 0 | 0 | 0 | 0 |
| cg14369777 | 3 | 612 | 1 | 710 | 0 | 0 | 0 | 0 | 0 | 0 | 0 | 0 |
| cg14460470 | 3 | 613 | 3 | 707 | 0 | 0 | 0 | 0 | 0 | 0 | 0 | 0 |
| cg14479037 | 3 | 618 | 1 | 704 | 0 | 0 | 0 | 0 | 0 | 0 | 0 | 0 |
| cg14520448 | 4 | 615 | 706 | 1 | 1 | 0 | 0 | 0 | 0 | 0 | 0 | 0 |
| cg14520892 | 2 | 618 | 705 | 0 | 0 | 0 | 0 | 0 | 0 | 0 | 0 | 0 |
| cg14533874 | 3 | 615 | 4 | 704 | 0 | 0 | 0 | 0 | 0 | 0 | 0 | 0 |
| cg14596086 | 2 | 615 | 708 | 0 | 0 | 0 | 0 | 0 | 0 | 0 | 0 | 0 |
| cg14642832 | 3 | 615 | 2 | 706 | 0 | 0 | 0 | 0 | 0 | 0 | 0 | 0 |
| cg14743649 | 2 | 609 | 714 | 0 | 0 | 0 | 0 | 0 | 0 | 0 | 0 | 0 |
| cg14772068 | 2 | 617 | 706 | 0 | 0 | 0 | 0 | 0 | 0 | 0 | 0 | 0 |
| cg14834143 | 3 | 612 | 1 | 710 | 0 | 0 | 0 | 0 | 0 | 0 | 0 | 0 |
| cg14959801 | 3 | 610 | 712 | 1 | 0 | 0 | 0 | 0 | 0 | 0 | 0 | 0 |
| cg15008991 | 2 | 618 | 705 | 0 | 0 | 0 | 0 | 0 | 0 | 0 | 0 | 0 |
| cg15050093 | 2 | 615 | 708 | 0 | 0 | 0 | 0 | 0 | 0 | 0 | 0 | 0 |
| cg15067665 | 4 | 592 | 706 | 1 | 24 | 0 | 0 | 0 | 0 | 0 | 0 | 0 |
| cg15132216 | 2 | 617 | 706 | 0 | 0 | 0 | 0 | 0 | 0 | 0 | 0 | 0 |
| cg15224006 | 2 | 617 | 706 | 0 | 0 | 0 | 0 | 0 | 0 | 0 | 0 | 0 |
| cg15231886 | 2 | 617 | 706 | 0 | 0 | 0 | 0 | 0 | 0 | 0 | 0 | 0 |
| cg15315638 | 2 | 616 | 707 | 0 | 0 | 0 | 0 | 0 | 0 | 0 | 0 | 0 |
| cg15465809 | 2 | 618 | 705 | 0 | 0 | 0 | 0 | 0 | 0 | 0 | 0 | 0 |
| cg15536552 | 2 | 616 | 707 | 0 | 0 | 0 | 0 | 0 | 0 | 0 | 0 | 0 |
| cg15667675 | 2 | 620 | 703 | 0 | 0 | 0 | 0 | 0 | 0 | 0 | 0 | 0 |
| cg15691129 | 2 | 617 | 706 | 0 | 0 | 0 | 0 | 0 | 0 | 0 | 0 | 0 |
| cg15715844 | 2 | 617 | 706 | 0 | 0 | 0 | 0 | 0 | 0 | 0 | 0 | 0 |
| cg15788661 | 4 | 616 | 705 | 1 | 1 | 0 | 0 | 0 | 0 | 0 | 0 | 0 |
| cg15798883 | 2 | 616 | 707 | 0 | 0 | 0 | 0 | 0 | 0 | 0 | 0 | 0 |
| cg15802548 | 4 | 616 | 705 | 1 | 1 | 0 | 0 | 0 | 0 | 0 | 0 | 0 |
| cg15953767 | 2 | 617 | 706 | 0 | 0 | 0 | 0 | 0 | 0 | 0 | 0 | 0 |
| cg15977272 | 2 | 615 | 708 | 0 | 0 | 0 | 0 | 0 | 0 | 0 | 0 | 0 |
| cg16091746 | 2 | 618 | 705 | 0 | 0 | 0 | 0 | 0 | 0 | 0 | 0 | 0 |
| cg16145139 | 2 | 616 | 707 | 0 | 0 | 0 | 0 | 0 | 0 | 0 | 0 | 0 |
| cg16190093 | 2 | 617 | 706 | 0 | 0 | 0 | 0 | 0 | 0 | 0 | 0 | 0 |
| cg16314146 | 2 | 616 | 707 | 0 | 0 | 0 | 0 | 0 | 0 | 0 | 0 | 0 |
| cg16343842 | 4 | 615 | 1 | 1 | 706 | 0 | 0 | 0 | 0 | 0 | 0 | 0 |
| cg16357225 | 3 | 618 | 1 | 704 | 0 | 0 | 0 | 0 | 0 | 0 | 0 | 0 |
| cg16405946 | 2 | 612 | 711 | 0 | 0 | 0 | 0 | 0 | 0 | 0 | 0 | 0 |
| cg16531277 | 2 | 620 | 703 | 0 | 0 | 0 | 0 | 0 | 0 | 0 | 0 | 0 |
| cg16567137 | 2 | 615 | 708 | 0 | 0 | 0 | 0 | 0 | 0 | 0 | 0 | 0 |
| cg16617753 | 2 | 616 | 707 | 0 | 0 | 0 | 0 | 0 | 0 | 0 | 0 | 0 |
| cg16639627 | 2 | 617 | 706 | 0 | 0 | 0 | 0 | 0 | 0 | 0 | 0 | 0 |
| cg16653057 | 2 | 608 | 715 | 0 | 0 | 0 | 0 | 0 | 0 | 0 | 0 | 0 |
| cg16723510 | 2 | 616 | 707 | 0 | 0 | 0 | 0 | 0 | 0 | 0 | 0 | 0 |
| cg16726201 | 2 | 616 | 707 | 0 | 0 | 0 | 0 | 0 | 0 | 0 | 0 | 0 |
| cg16832275 | 3 | 616 | 2 | 705 | 0 | 0 | 0 | 0 | 0 | 0 | 0 | 0 |
| cg16970748 | 2 | 618 | 705 | 0 | 0 | 0 | 0 | 0 | 0 | 0 | 0 | 0 |
| cg17008556 | 2 | 620 | 703 | 0 | 0 | 0 | 0 | 0 | 0 | 0 | 0 | 0 |
| cg17122979 | 2 | 620 | 703 | 0 | 0 | 0 | 0 | 0 | 0 | 0 | 0 | 0 |
| cg17197278 | 2 | 618 | 705 | 0 | 0 | 0 | 0 | 0 | 0 | 0 | 0 | 0 |
| cg17227030 | 2 | 617 | 706 | 0 | 0 | 0 | 0 | 0 | 0 | 0 | 0 | 0 |
| cg17238522 | 3 | 615 | 2 | 706 | 0 | 0 | 0 | 0 | 0 | 0 | 0 | 0 |
| cg17294479 | 2 | 622 | 701 | 0 | 0 | 0 | 0 | 0 | 0 | 0 | 0 | 0 |
| cg17345373 | 2 | 620 | 703 | 0 | 0 | 0 | 0 | 0 | 0 | 0 | 0 | 0 |
| cg17355765 | 2 | 617 | 706 | 0 | 0 | 0 | 0 | 0 | 0 | 0 | 0 | 0 |
| cg17503853 | 3 | 618 | 704 | 1 | 0 | 0 | 0 | 0 | 0 | 0 | 0 | 0 |
| cg17508549 | 3 | 612 | 5 | 706 | 0 | 0 | 0 | 0 | 0 | 0 | 0 | 0 |
| cg17552650 | 3 | 616 | 2 | 705 | 0 | 0 | 0 | 0 | 0 | 0 | 0 | 0 |
| cg17670641 | 2 | 616 | 707 | 0 | 0 | 0 | 0 | 0 | 0 | 0 | 0 | 0 |
| cg17758583 | 3 | 618 | 702 | 3 | 0 | 0 | 0 | 0 | 0 | 0 | 0 | 0 |
| cg17835180 | 3 | 614 | 706 | 3 | 0 | 0 | 0 | 0 | 0 | 0 | 0 | 0 |
| cg17853057 | 2 | 614 | 709 | 0 | 0 | 0 | 0 | 0 | 0 | 0 | 0 | 0 |
| cg17934320 | 3 | 615 | 707 | 1 | 0 | 0 | 0 | 0 | 0 | 0 | 0 | 0 |
| cg17970282 | 2 | 571 | 752 | 0 | 0 | 0 | 0 | 0 | 0 | 0 | 0 | 0 |
| cg17995340 | 3 | 614 | 1 | 708 | 0 | 0 | 0 | 0 | 0 | 0 | 0 | 0 |
| cg18030003 | 3 | 616 | 704 | 3 | 0 | 0 | 0 | 0 | 0 | 0 | 0 | 0 |
| cg18091964 | 2 | 615 | 708 | 0 | 0 | 0 | 0 | 0 | 0 | 0 | 0 | 0 |
| cg18093711 | 2 | 616 | 707 | 0 | 0 | 0 | 0 | 0 | 0 | 0 | 0 | 0 |
| cg18156601 | 2 | 617 | 706 | 0 | 0 | 0 | 0 | 0 | 0 | 0 | 0 | 0 |
| cg18159654 | 3 | 616 | 706 | 1 | 0 | 0 | 0 | 0 | 0 | 0 | 0 | 0 |
| cg18307604 | 3 | 614 | 706 | 3 | 0 | 0 | 0 | 0 | 0 | 0 | 0 | 0 |
| cg18399551 | 2 | 618 | 705 | 0 | 0 | 0 | 0 | 0 | 0 | 0 | 0 | 0 |
| cg18414950 | 2 | 619 | 704 | 0 | 0 | 0 | 0 | 0 | 0 | 0 | 0 | 0 |
| cg18780401 | 2 | 616 | 707 | 0 | 0 | 0 | 0 | 0 | 0 | 0 | 0 | 0 |
| cg18785414 | 2 | 617 | 706 | 0 | 0 | 0 | 0 | 0 | 0 | 0 | 0 | 0 |
| cg19039586 | 2 | 621 | 702 | 0 | 0 | 0 | 0 | 0 | 0 | 0 | 0 | 0 |
| cg19062189 | 3 | 616 | 706 | 1 | 0 | 0 | 0 | 0 | 0 | 0 | 0 | 0 |
| cg19063343 | 2 | 619 | 704 | 0 | 0 | 0 | 0 | 0 | 0 | 0 | 0 | 0 |
| cg19212576 | 2 | 617 | 706 | 0 | 0 | 0 | 0 | 0 | 0 | 0 | 0 | 0 |
| cg19235109 | 3 | 608 | 8 | 707 | 0 | 0 | 0 | 0 | 0 | 0 | 0 | 0 |
| cg19257537 | 3 | 614 | 708 | 1 | 0 | 0 | 0 | 0 | 0 | 0 | 0 | 0 |
| cg19280671 | 4 | 15 | 1 | 66 | 1241 | 0 | 0 | 0 | 0 | 0 | 0 | 0 |
| cg19410841 | 2 | 618 | 705 | 0 | 0 | 0 | 0 | 0 | 0 | 0 | 0 | 0 |
| cg19523014 | 3 | 615 | 707 | 1 | 0 | 0 | 0 | 0 | 0 | 0 | 0 | 0 |
| cg19548593 | 3 | 619 | 703 | 1 | 0 | 0 | 0 | 0 | 0 | 0 | 0 | 0 |
| cg19586382 | 2 | 617 | 706 | 0 | 0 | 0 | 0 | 0 | 0 | 0 | 0 | 0 |
| cg19616372 | 3 | 612 | 4 | 707 | 0 | 0 | 0 | 0 | 0 | 0 | 0 | 0 |
| cg19658849 | 2 | 616 | 707 | 0 | 0 | 0 | 0 | 0 | 0 | 0 | 0 | 0 |
| cg19679687 | 2 | 617 | 706 | 0 | 0 | 0 | 0 | 0 | 0 | 0 | 0 | 0 |
| cg19716713 | 2 | 607 | 716 | 0 | 0 | 0 | 0 | 0 | 0 | 0 | 0 | 0 |
| cg19732480 | 3 | 617 | 704 | 2 | 0 | 0 | 0 | 0 | 0 | 0 | 0 | 0 |
| cg19797013 | 2 | 612 | 711 | 0 | 0 | 0 | 0 | 0 | 0 | 0 | 0 | 0 |
| cg19840763 | 3 | 616 | 706 | 1 | 0 | 0 | 0 | 0 | 0 | 0 | 0 | 0 |
| cg19854896 | 3 | 617 | 1 | 705 | 0 | 0 | 0 | 0 | 0 | 0 | 0 | 0 |
| cg19928247 | 2 | 610 | 713 | 0 | 0 | 0 | 0 | 0 | 0 | 0 | 0 | 0 |
| cg19930056 | 2 | 616 | 707 | 0 | 0 | 0 | 0 | 0 | 0 | 0 | 0 | 0 |
| cg19933311 | 3 | 288 | 2 | 1033 | 0 | 0 | 0 | 0 | 0 | 0 | 0 | 0 |
| cg20056133 | 2 | 617 | 706 | 0 | 0 | 0 | 0 | 0 | 0 | 0 | 0 | 0 |
| cg20291091 | 4 | 617 | 1 | 703 | 2 | 0 | 0 | 0 | 0 | 0 | 0 | 0 |
| cg20340631 | 2 | 620 | 703 | 0 | 0 | 0 | 0 | 0 | 0 | 0 | 0 | 0 |
| cg20416767 | 3 | 621 | 701 | 1 | 0 | 0 | 0 | 0 | 0 | 0 | 0 | 0 |
| cg20504202 | 3 | 612 | 5 | 706 | 0 | 0 | 0 | 0 | 0 | 0 | 0 | 0 |
| cg20514061 | 2 | 618 | 705 | 0 | 0 | 0 | 0 | 0 | 0 | 0 | 0 | 0 |
| cg20641280 | 3 | 615 | 1 | 707 | 0 | 0 | 0 | 0 | 0 | 0 | 0 | 0 |
| cg20662859 | 2 | 616 | 707 | 0 | 0 | 0 | 0 | 0 | 0 | 0 | 0 | 0 |
| cg20790618 | 2 | 617 | 706 | 0 | 0 | 0 | 0 | 0 | 0 | 0 | 0 | 0 |
| cg20816612 | 3 | 615 | 706 | 2 | 0 | 0 | 0 | 0 | 0 | 0 | 0 | 0 |
| cg20971536 | 3 | 617 | 1 | 705 | 0 | 0 | 0 | 0 | 0 | 0 | 0 | 0 |
| cg21028156 | 4 | 903 | 1 | 1 | 418 | 0 | 0 | 0 | 0 | 0 | 0 | 0 |
| cg21046413 | 2 | 618 | 705 | 0 | 0 | 0 | 0 | 0 | 0 | 0 | 0 | 0 |
| cg21080294 | 2 | 612 | 711 | 0 | 0 | 0 | 0 | 0 | 0 | 0 | 0 | 0 |
| cg21127593 | 2 | 611 | 712 | 0 | 0 | 0 | 0 | 0 | 0 | 0 | 0 | 0 |
| cg21142420 | 2 | 620 | 703 | 0 | 0 | 0 | 0 | 0 | 0 | 0 | 0 | 0 |
| cg21201934 | 3 | 617 | 2 | 704 | 0 | 0 | 0 | 0 | 0 | 0 | 0 | 0 |
| cg21360798 | 2 | 616 | 707 | 0 | 0 | 0 | 0 | 0 | 0 | 0 | 0 | 0 |
| cg21365235 | 2 | 617 | 706 | 0 | 0 | 0 | 0 | 0 | 0 | 0 | 0 | 0 |
| cg21397157 | 2 | 618 | 705 | 0 | 0 | 0 | 0 | 0 | 0 | 0 | 0 | 0 |
| cg21491240 | 3 | 616 | 703 | 4 | 0 | 0 | 0 | 0 | 0 | 0 | 0 | 0 |
| cg21537736 | 2 | 618 | 705 | 0 | 0 | 0 | 0 | 0 | 0 | 0 | 0 | 0 |
| cg21606887 | 2 | 618 | 705 | 0 | 0 | 0 | 0 | 0 | 0 | 0 | 0 | 0 |
| cg21797452 | 2 | 711 | 612 | 0 | 0 | 0 | 0 | 0 | 0 | 0 | 0 | 0 |
| cg21910941 | 3 | 616 | 706 | 1 | 0 | 0 | 0 | 0 | 0 | 0 | 0 | 0 |
| cg21988739 | 2 | 619 | 704 | 0 | 0 | 0 | 0 | 0 | 0 | 0 | 0 | 0 |
| cg22012543 | 3 | 616 | 705 | 2 | 0 | 0 | 0 | 0 | 0 | 0 | 0 | 0 |
| cg22134140 | 2 | 612 | 711 | 0 | 0 | 0 | 0 | 0 | 0 | 0 | 0 | 0 |
| cg22151131 | 3 | 616 | 705 | 2 | 0 | 0 | 0 | 0 | 0 | 0 | 0 | 0 |
| cg22252245 | 2 | 616 | 707 | 0 | 0 | 0 | 0 | 0 | 0 | 0 | 0 | 0 |
| cg22404117 | 3 | 616 | 706 | 1 | 0 | 0 | 0 | 0 | 0 | 0 | 0 | 0 |
| cg22471695 | 2 | 617 | 706 | 0 | 0 | 0 | 0 | 0 | 0 | 0 | 0 | 0 |
| cg22484503 | 2 | 617 | 706 | 0 | 0 | 0 | 0 | 0 | 0 | 0 | 0 | 0 |
| cg22530983 | 3 | 1 | 92 | 1230 | 0 | 0 | 0 | 0 | 0 | 0 | 0 | 0 |
| cg22569627 | 2 | 618 | 705 | 0 | 0 | 0 | 0 | 0 | 0 | 0 | 0 | 0 |
| cg22604777 | 3 | 616 | 1 | 706 | 0 | 0 | 0 | 0 | 0 | 0 | 0 | 0 |
| cg22618269 | 3 | 613 | 2 | 708 | 0 | 0 | 0 | 0 | 0 | 0 | 0 | 0 |
| cg22645859 | 2 | 620 | 703 | 0 | 0 | 0 | 0 | 0 | 0 | 0 | 0 | 0 |
| cg22646149 | 3 | 616 | 706 | 1 | 0 | 0 | 0 | 0 | 0 | 0 | 0 | 0 |
| cg22655232 | 3 | 1 | 711 | 611 | 0 | 0 | 0 | 0 | 0 | 0 | 0 | 0 |
| cg22660483 | 2 | 616 | 707 | 0 | 0 | 0 | 0 | 0 | 0 | 0 | 0 | 0 |
| cg22713892 | 4 | 616 | 3 | 700 | 4 | 0 | 0 | 0 | 0 | 0 | 0 | 0 |
| cg22827938 | 2 | 615 | 708 | 0 | 0 | 0 | 0 | 0 | 0 | 0 | 0 | 0 |
| cg22876425 | 2 | 604 | 719 | 0 | 0 | 0 | 0 | 0 | 0 | 0 | 0 | 0 |
| cg22952406 | 2 | 620 | 703 | 0 | 0 | 0 | 0 | 0 | 0 | 0 | 0 | 0 |
| cg23112505 | 2 | 616 | 707 | 0 | 0 | 0 | 0 | 0 | 0 | 0 | 0 | 0 |
| cg23119380 | 2 | 616 | 707 | 0 | 0 | 0 | 0 | 0 | 0 | 0 | 0 | 0 |
| cg23157067 | 2 | 615 | 708 | 0 | 0 | 0 | 0 | 0 | 0 | 0 | 0 | 0 |
| cg23277098 | 2 | 616 | 707 | 0 | 0 | 0 | 0 | 0 | 0 | 0 | 0 | 0 |
| cg23280339 | 2 | 617 | 706 | 0 | 0 | 0 | 0 | 0 | 0 | 0 | 0 | 0 |
| cg23374711 | 3 | 619 | 703 | 1 | 0 | 0 | 0 | 0 | 0 | 0 | 0 | 0 |
| cg23390865 | 2 | 617 | 706 | 0 | 0 | 0 | 0 | 0 | 0 | 0 | 0 | 0 |
| cg23412653 | 2 | 616 | 707 | 0 | 0 | 0 | 0 | 0 | 0 | 0 | 0 | 0 |
| cg23486773 | 3 | 617 | 705 | 1 | 0 | 0 | 0 | 0 | 0 | 0 | 0 | 0 |
| cg23545272 | 2 | 620 | 703 | 0 | 0 | 0 | 0 | 0 | 0 | 0 | 0 | 0 |
| cg23554546 | 4 | 615 | 2 | 1 | 705 | 0 | 0 | 0 | 0 | 0 | 0 | 0 |
| cg23675214 | 3 | 616 | 706 | 1 | 0 | 0 | 0 | 0 | 0 | 0 | 0 | 0 |
| cg23694557 | 3 | 615 | 707 | 1 | 0 | 0 | 0 | 0 | 0 | 0 | 0 | 0 |
| cg23698956 | 2 | 616 | 707 | 0 | 0 | 0 | 0 | 0 | 0 | 0 | 0 | 0 |
| cg23718917 | 2 | 616 | 707 | 0 | 0 | 0 | 0 | 0 | 0 | 0 | 0 | 0 |
| cg23832225 | 2 | 616 | 707 | 0 | 0 | 0 | 0 | 0 | 0 | 0 | 0 | 0 |
| cg23875045 | 2 | 615 | 708 | 0 | 0 | 0 | 0 | 0 | 0 | 0 | 0 | 0 |
| cg24052239 | 3 | 616 | 706 | 1 | 0 | 0 | 0 | 0 | 0 | 0 | 0 | 0 |
| cg24139739 | 2 | 615 | 708 | 0 | 0 | 0 | 0 | 0 | 0 | 0 | 0 | 0 |
| cg24139895 | 2 | 608 | 715 | 0 | 0 | 0 | 0 | 0 | 0 | 0 | 0 | 0 |
| cg24194941 | 3 | 620 | 702 | 1 | 0 | 0 | 0 | 0 | 0 | 0 | 0 | 0 |
| cg24340926 | 2 | 617 | 706 | 0 | 0 | 0 | 0 | 0 | 0 | 0 | 0 | 0 |
| cg24378152 | 2 | 616 | 707 | 0 | 0 | 0 | 0 | 0 | 0 | 0 | 0 | 0 |
| cg24389217 | 2 | 616 | 707 | 0 | 0 | 0 | 0 | 0 | 0 | 0 | 0 | 0 |
| cg24479484 | 2 | 609 | 714 | 0 | 0 | 0 | 0 | 0 | 0 | 0 | 0 | 0 |
| cg24779040 | 3 | 616 | 706 | 1 | 0 | 0 | 0 | 0 | 0 | 0 | 0 | 0 |
| cg24829975 | 2 | 615 | 708 | 0 | 0 | 0 | 0 | 0 | 0 | 0 | 0 | 0 |
| cg24831179 | 2 | 616 | 707 | 0 | 0 | 0 | 0 | 0 | 0 | 0 | 0 | 0 |
| cg24888621 | 2 | 617 | 706 | 0 | 0 | 0 | 0 | 0 | 0 | 0 | 0 | 0 |
| cg24923226 | 2 | 613 | 710 | 0 | 0 | 0 | 0 | 0 | 0 | 0 | 0 | 0 |
| cg24964466 | 2 | 616 | 707 | 0 | 0 | 0 | 0 | 0 | 0 | 0 | 0 | 0 |
| cg25053301 | 2 | 615 | 708 | 0 | 0 | 0 | 0 | 0 | 0 | 0 | 0 | 0 |
| cg25078813 | 3 | 617 | 705 | 1 | 0 | 0 | 0 | 0 | 0 | 0 | 0 | 0 |
| cg25129414 | 2 | 616 | 707 | 0 | 0 | 0 | 0 | 0 | 0 | 0 | 0 | 0 |
| cg25179313 | 3 | 616 | 706 | 1 | 0 | 0 | 0 | 0 | 0 | 0 | 0 | 0 |
| cg25220235 | 2 | 618 | 705 | 0 | 0 | 0 | 0 | 0 | 0 | 0 | 0 | 0 |
| cg25317260 | 3 | 618 | 1 | 704 | 0 | 0 | 0 | 0 | 0 | 0 | 0 | 0 |
| cg25496792 | 2 | 616 | 707 | 0 | 0 | 0 | 0 | 0 | 0 | 0 | 0 | 0 |
| cg25514427 | 2 | 621 | 702 | 0 | 0 | 0 | 0 | 0 | 0 | 0 | 0 | 0 |
| cg25547772 | 2 | 618 | 705 | 0 | 0 | 0 | 0 | 0 | 0 | 0 | 0 | 0 |
| cg25576048 | 2 | 614 | 709 | 0 | 0 | 0 | 0 | 0 | 0 | 0 | 0 | 0 |
| cg25591670 | 2 | 616 | 707 | 0 | 0 | 0 | 0 | 0 | 0 | 0 | 0 | 0 |
| cg25645693 | 2 | 618 | 705 | 0 | 0 | 0 | 0 | 0 | 0 | 0 | 0 | 0 |
| cg25645840 | 2 | 616 | 707 | 0 | 0 | 0 | 0 | 0 | 0 | 0 | 0 | 0 |
| cg25692732 | 2 | 613 | 710 | 0 | 0 | 0 | 0 | 0 | 0 | 0 | 0 | 0 |
| cg25712015 | 3 | 617 | 705 | 1 | 0 | 0 | 0 | 0 | 0 | 0 | 0 | 0 |
| cg25813820 | 2 | 611 | 712 | 0 | 0 | 0 | 0 | 0 | 0 | 0 | 0 | 0 |
| cg25852925 | 2 | 615 | 708 | 0 | 0 | 0 | 0 | 0 | 0 | 0 | 0 | 0 |
| cg25874079 | 2 | 616 | 707 | 0 | 0 | 0 | 0 | 0 | 0 | 0 | 0 | 0 |
| cg25943986 | 2 | 618 | 705 | 0 | 0 | 0 | 0 | 0 | 0 | 0 | 0 | 0 |
| cg26055054 | 2 | 616 | 707 | 0 | 0 | 0 | 0 | 0 | 0 | 0 | 0 | 0 |
| cg26129200 | 4 | 613 | 3 | 705 | 2 | 0 | 0 | 0 | 0 | 0 | 0 | 0 |
| cg26135767 | 2 | 617 | 706 | 0 | 0 | 0 | 0 | 0 | 0 | 0 | 0 | 0 |
| cg26155828 | 3 | 617 | 705 | 1 | 0 | 0 | 0 | 0 | 0 | 0 | 0 | 0 |
| cg26176649 | 2 | 616 | 707 | 0 | 0 | 0 | 0 | 0 | 0 | 0 | 0 | 0 |
| cg26287822 | 3 | 344 | 247 | 732 | 0 | 0 | 0 | 0 | 0 | 0 | 0 | 0 |
| cg26301245 | 2 | 620 | 703 | 0 | 0 | 0 | 0 | 0 | 0 | 0 | 0 | 0 |
| cg26359388 | 4 | 3 | 704 | 2 | 614 | 0 | 0 | 0 | 0 | 0 | 0 | 0 |
| cg26688923 | 3 | 613 | 709 | 1 | 0 | 0 | 0 | 0 | 0 | 0 | 0 | 0 |
| cg26792541 | 2 | 615 | 708 | 0 | 0 | 0 | 0 | 0 | 0 | 0 | 0 | 0 |
| cg26801383 | 3 | 615 | 706 | 2 | 0 | 0 | 0 | 0 | 0 | 0 | 0 | 0 |
| cg26807533 | 6 | 614 | 1 | 695 | 7 | 1 | 5 | 0 | 0 | 0 | 0 | 0 |
| cg26810336 | 3 | 615 | 707 | 1 | 0 | 0 | 0 | 0 | 0 | 0 | 0 | 0 |
| cg26814883 | 2 | 612 | 711 | 0 | 0 | 0 | 0 | 0 | 0 | 0 | 0 | 0 |
| cg26907889 | 2 | 615 | 708 | 0 | 0 | 0 | 0 | 0 | 0 | 0 | 0 | 0 |
| cg27187671 | 3 | 618 | 704 | 1 | 0 | 0 | 0 | 0 | 0 | 0 | 0 | 0 |
| cg27198071 | 2 | 616 | 707 | 0 | 0 | 0 | 0 | 0 | 0 | 0 | 0 | 0 |
| cg27198824 | 2 | 618 | 705 | 0 | 0 | 0 | 0 | 0 | 0 | 0 | 0 | 0 |
| cg27375748 | 2 | 617 | 706 | 0 | 0 | 0 | 0 | 0 | 0 | 0 | 0 | 0 |
| cg27485646 | 3 | 615 | 1 | 707 | 0 | 0 | 0 | 0 | 0 | 0 | 0 | 0 |
| cg27505047 | 2 | 615 | 708 | 0 | 0 | 0 | 0 | 0 | 0 | 0 | 0 | 0 |
| cg27519679 | 3 | 616 | 706 | 1 | 0 | 0 | 0 | 0 | 0 | 0 | 0 | 0 |
| cg27615590 | 2 | 616 | 707 | 0 | 0 | 0 | 0 | 0 | 0 | 0 | 0 | 0 |
| cg00017157 | 5 | 439 | 1 | 1 | 629 | 253 | 0 | 0 | 0 | 0 | 0 | 0 |
| cg00045070 | 4 | 488 | 1 | 568 | 266 | 0 | 0 | 0 | 0 | 0 | 0 | 0 |
| cg00069771 | 3 | 57 | 446 | 820 | 0 | 0 | 0 | 0 | 0 | 0 | 0 | 0 |
| cg00084271 | 6 | 87 | 1 | 2 | 418 | 6 | 809 | 0 | 0 | 0 | 0 | 0 |
| cg00123214 | 4 | 166 | 3 | 565 | 589 | 0 | 0 | 0 | 0 | 0 | 0 | 0 |
| cg00124902 | 3 | 93 | 1227 | 3 | 0 | 0 | 0 | 0 | 0 | 0 | 0 | 0 |
| cg00167913 | 3 | 126 | 1 | 1196 | 0 | 0 | 0 | 0 | 0 | 0 | 0 | 0 |
| cg00256632 | 4 | 5 | 1 | 71 | 1246 | 0 | 0 | 0 | 0 | 0 | 0 | 0 |
| cg00345083 | 4 | 326 | 1 | 654 | 342 | 0 | 0 | 0 | 0 | 0 | 0 | 0 |
| cg00669623 | 4 | 1155 | 151 | 10 | 7 | 0 | 0 | 0 | 0 | 0 | 0 | 0 |
| cg00794381 | 4 | 4 | 2 | 102 | 1215 | 0 | 0 | 0 | 0 | 0 | 0 | 0 |
| cg00894041 | 4 | 5 | 82 | 1 | 1235 | 0 | 0 | 0 | 0 | 0 | 0 | 0 |
| cg01081438 | 5 | 569 | 2 | 557 | 194 | 1 | 0 | 0 | 0 | 0 | 0 | 0 |
| cg01195628 | 4 | 1 | 73 | 3 | 1246 | 0 | 0 | 0 | 0 | 0 | 0 | 0 |
| cg01296877 | 4 | 171 | 2 | 517 | 633 | 0 | 0 | 0 | 0 | 0 | 0 | 0 |
| cg01463139 | 2 | 381 | 942 | 0 | 0 | 0 | 0 | 0 | 0 | 0 | 0 | 0 |
| cg01778345 | 4 | 160 | 1 | 3 | 1159 | 0 | 0 | 0 | 0 | 0 | 0 | 0 |
| cg01796338 | 4 | 46 | 216 | 2 | 1059 | 0 | 0 | 0 | 0 | 0 | 0 | 0 |
| cg01802772 | 2 | 532 | 791 | 0 | 0 | 0 | 0 | 0 | 0 | 0 | 0 | 0 |
| cg01821635 | 4 | 276 | 1 | 1 | 1045 | 0 | 0 | 0 | 0 | 0 | 0 | 0 |
| cg01836910 | 3 | 10 | 121 | 1192 | 0 | 0 | 0 | 0 | 0 | 0 | 0 | 0 |
| cg01876809 | 3 | 256 | 651 | 416 | 0 | 0 | 0 | 0 | 0 | 0 | 0 | 0 |
| cg02097890 | 3 | 3 | 82 | 1238 | 0 | 0 | 0 | 0 | 0 | 0 | 0 | 0 |
| cg02293501 | 4 | 19 | 1 | 132 | 1171 | 0 | 0 | 0 | 0 | 0 | 0 | 0 |
| cg02694395 | 4 | 15 | 137 | 1170 | 1 | 0 | 0 | 0 | 0 | 0 | 0 | 0 |
| cg02764188 | 3 | 25 | 145 | 1153 | 0 | 0 | 0 | 0 | 0 | 0 | 0 | 0 |
| cg02890259 | 5 | 539 | 1 | 559 | 1 | 223 | 0 | 0 | 0 | 0 | 0 | 0 |
| cg03041055 | 4 | 4 | 1 | 64 | 1254 | 0 | 0 | 0 | 0 | 0 | 0 | 0 |
| cg03053553 | 3 | 67 | 1 | 1255 | 0 | 0 | 0 | 0 | 0 | 0 | 0 | 0 |
| cg03129555 | 4 | 20 | 225 | 2 | 1076 | 0 | 0 | 0 | 0 | 0 | 0 | 0 |
| cg03221390 | 3 | 222 | 568 | 533 | 0 | 0 | 0 | 0 | 0 | 0 | 0 | 0 |
| cg03224005 | 5 | 29 | 304 | 1 | 1 | 988 | 0 | 0 | 0 | 0 | 0 | 0 |
| cg03263197 | 2 | 142 | 1181 | 0 | 0 | 0 | 0 | 0 | 0 | 0 | 0 | 0 |
| cg03373960 | 2 | 96 | 1227 | 0 | 0 | 0 | 0 | 0 | 0 | 0 | 0 | 0 |
| cg03396604 | 4 | 151 | 1 | 2 | 1169 | 0 | 0 | 0 | 0 | 0 | 0 | 0 |
| cg03420970 | 4 | 1 | 73 | 1 | 1248 | 0 | 0 | 0 | 0 | 0 | 0 | 0 |
| cg03428888 | 5 | 3 | 2 | 2 | 71 | 1245 | 0 | 0 | 0 | 0 | 0 | 0 |
| cg03548008 | 3 | 61 | 7 | 1255 | 0 | 0 | 0 | 0 | 0 | 0 | 0 | 0 |
| cg03881934 | 5 | 1 | 1 | 1 | 67 | 1253 | 0 | 0 | 0 | 0 | 0 | 0 |
| cg03964373 | 5 | 68 | 1 | 1 | 383 | 870 | 0 | 0 | 0 | 0 | 0 | 0 |
| cg04083966 | 4 | 24 | 233 | 1 | 1065 | 0 | 0 | 0 | 0 | 0 | 0 | 0 |
| cg04246708 | 5 | 222 | 1 | 534 | 1 | 565 | 0 | 0 | 0 | 0 | 0 | 0 |
| cg04323713 | 3 | 3 | 92 | 1228 | 0 | 0 | 0 | 0 | 0 | 0 | 0 | 0 |
| cg04411337 | 5 | 7 | 105 | 1 | 1 | 1209 | 0 | 0 | 0 | 0 | 0 | 0 |
| cg04610742 | 3 | 85 | 1237 | 1 | 0 | 0 | 0 | 0 | 0 | 0 | 0 | 0 |
| cg04798314 | 3 | 258 | 1 | 1064 | 0 | 0 | 0 | 0 | 0 | 0 | 0 | 0 |
| cg04835511 | 4 | 321 | 3 | 1 | 998 | 0 | 0 | 0 | 0 | 0 | 0 | 0 |
| cg04882216 | 2 | 195 | 1128 | 0 | 0 | 0 | 0 | 0 | 0 | 0 | 0 | 0 |
| cg04888234 | 3 | 157 | 542 | 624 | 0 | 0 | 0 | 0 | 0 | 0 | 0 | 0 |
| cg04922029 | 2 | 1008 | 315 | 0 | 0 | 0 | 0 | 0 | 0 | 0 | 0 | 0 |
| cg05460975 | 4 | 8 | 1 | 117 | 1197 | 0 | 0 | 0 | 0 | 0 | 0 | 0 |
| cg05704942 | 5 | 449 | 4 | 3 | 634 | 233 | 0 | 0 | 0 | 0 | 0 | 0 |
| cg05712748 | 4 | 70 | 384 | 1 | 868 | 0 | 0 | 0 | 0 | 0 | 0 | 0 |
| cg05794927 | 3 | 30 | 224 | 1069 | 0 | 0 | 0 | 0 | 0 | 0 | 0 | 0 |
| cg05795077 | 3 | 3 | 68 | 1252 | 0 | 0 | 0 | 0 | 0 | 0 | 0 | 0 |
| cg06129455 | 4 | 139 | 1 | 3 | 1180 | 0 | 0 | 0 | 0 | 0 | 0 | 0 |
| cg06197811 | 4 | 1 | 1 | 106 | 1215 | 0 | 0 | 0 | 0 | 0 | 0 | 0 |
| cg06205333 | 2 | 629 | 694 | 0 | 0 | 0 | 0 | 0 | 0 | 0 | 0 | 0 |
| cg06437931 | 2 | 89 | 1234 | 0 | 0 | 0 | 0 | 0 | 0 | 0 | 0 | 0 |
| cg06621919 | 4 | 128 | 4 | 2 | 1189 | 0 | 0 | 0 | 0 | 0 | 0 | 0 |
| cg06804873 | 3 | 23 | 271 | 1029 | 0 | 0 | 0 | 0 | 0 | 0 | 0 | 0 |
| cg06961873 | 5 | 268 | 3 | 600 | 4 | 448 | 0 | 0 | 0 | 0 | 0 | 0 |
| cg06962428 | 3 | 12 | 102 | 1209 | 0 | 0 | 0 | 0 | 0 | 0 | 0 | 0 |
| cg06981887 | 3 | 4 | 65 | 1254 | 0 | 0 | 0 | 0 | 0 | 0 | 0 | 0 |
| cg06999043 | 2 | 126 | 1197 | 0 | 0 | 0 | 0 | 0 | 0 | 0 | 0 | 0 |
| cg07134368 | 3 | 223 | 2 | 1098 | 0 | 0 | 0 | 0 | 0 | 0 | 0 | 0 |
| cg07156839 | 2 | 231 | 1092 | 0 | 0 | 0 | 0 | 0 | 0 | 0 | 0 | 0 |
| cg07180971 | 5 | 36 | 162 | 1 | 1 | 1123 | 0 | 0 | 0 | 0 | 0 | 0 |
| cg07318158 | 3 | 5 | 73 | 1245 | 0 | 0 | 0 | 0 | 0 | 0 | 0 | 0 |
| cg07371337 | 4 | 7 | 1 | 116 | 1199 | 0 | 0 | 0 | 0 | 0 | 0 | 0 |
| cg07392838 | 3 | 4 | 69 | 1250 | 0 | 0 | 0 | 0 | 0 | 0 | 0 | 0 |
| cg07501029 | 4 | 214 | 2 | 1 | 1106 | 0 | 0 | 0 | 0 | 0 | 0 | 0 |
| cg07584620 | 5 | 11 | 1 | 223 | 1 | 1087 | 0 | 0 | 0 | 0 | 0 | 0 |
| cg07592775 | 3 | 125 | 3 | 1195 | 0 | 0 | 0 | 0 | 0 | 0 | 0 | 0 |
| cg07703391 | 4 | 137 | 1 | 519 | 666 | 0 | 0 | 0 | 0 | 0 | 0 | 0 |
| cg07796016 | 4 | 77 | 1 | 1 | 1244 | 0 | 0 | 0 | 0 | 0 | 0 | 0 |
| cg08121686 | 2 | 82 | 1241 | 0 | 0 | 0 | 0 | 0 | 0 | 0 | 0 | 0 |
| cg08250118 | 6 | 177 | 1 | 539 | 1 | 2 | 603 | 0 | 0 | 0 | 0 | 0 |
| cg08266644 | 2 | 213 | 1110 | 0 | 0 | 0 | 0 | 0 | 0 | 0 | 0 | 0 |
| cg08332163 | 4 | 76 | 1 | 1 | 1245 | 0 | 0 | 0 | 0 | 0 | 0 | 0 |
| cg08477332 | 4 | 545 | 1 | 484 | 293 | 0 | 0 | 0 | 0 | 0 | 0 | 0 |
| cg08639434 | 5 | 1 | 1 | 78 | 1 | 1242 | 0 | 0 | 0 | 0 | 0 | 0 |
| cg08937639 | 4 | 2 | 1 | 77 | 1243 | 0 | 0 | 0 | 0 | 0 | 0 | 0 |
| cg08942192 | 2 | 98 | 1225 | 0 | 0 | 0 | 0 | 0 | 0 | 0 | 0 | 0 |
| cg08950364 | 5 | 74 | 1 | 1 | 1245 | 2 | 0 | 0 | 0 | 0 | 0 | 0 |
| cg09134876 | 3 | 98 | 1 | 1224 | 0 | 0 | 0 | 0 | 0 | 0 | 0 | 0 |
| cg09518270 | 2 | 142 | 1181 | 0 | 0 | 0 | 0 | 0 | 0 | 0 | 0 | 0 |
| cg09762182 | 4 | 131 | 1 | 455 | 736 | 0 | 0 | 0 | 0 | 0 | 0 | 0 |
| cg09799733 | 3 | 3 | 73 | 1247 | 0 | 0 | 0 | 0 | 0 | 0 | 0 | 0 |
| cg09900152 | 4 | 4 | 73 | 1 | 1245 | 0 | 0 | 0 | 0 | 0 | 0 | 0 |
| cg10030147 | 3 | 2 | 92 | 1229 | 0 | 0 | 0 | 0 | 0 | 0 | 0 | 0 |
| cg10091792 | 4 | 126 | 1 | 542 | 654 | 0 | 0 | 0 | 0 | 0 | 0 | 0 |
| cg10117077 | 2 | 544 | 779 | 0 | 0 | 0 | 0 | 0 | 0 | 0 | 0 | 0 |
| cg10130257 | 3 | 58 | 234 | 1031 | 0 | 0 | 0 | 0 | 0 | 0 | 0 | 0 |
| cg10144473 | 4 | 10 | 166 | 1 | 1146 | 0 | 0 | 0 | 0 | 0 | 0 | 0 |
| cg10155537 | 4 | 315 | 1 | 2 | 1005 | 0 | 0 | 0 | 0 | 0 | 0 | 0 |
| cg10316617 | 5 | 35 | 225 | 1 | 2 | 1060 | 0 | 0 | 0 | 0 | 0 | 0 |
| cg10506445 | 3 | 178 | 3 | 1142 | 0 | 0 | 0 | 0 | 0 | 0 | 0 | 0 |
| cg10508557 | 3 | 3 | 75 | 1245 | 0 | 0 | 0 | 0 | 0 | 0 | 0 | 0 |
| cg10536901 | 3 | 1 | 79 | 1243 | 0 | 0 | 0 | 0 | 0 | 0 | 0 | 0 |
| cg10625579 | 4 | 9 | 105 | 1 | 1208 | 0 | 0 | 0 | 0 | 0 | 0 | 0 |
| cg10644916 | 3 | 10 | 170 | 1143 | 0 | 0 | 0 | 0 | 0 | 0 | 0 | 0 |
| cg10701801 | 4 | 888 | 1 | 383 | 51 | 0 | 0 | 0 | 0 | 0 | 0 | 0 |
| cg10759817 | 5 | 66 | 1 | 307 | 1 | 948 | 0 | 0 | 0 | 0 | 0 | 0 |
| cg10818676 | 3 | 200 | 559 | 564 | 0 | 0 | 0 | 0 | 0 | 0 | 0 | 0 |
| cg10970349 | 3 | 3 | 102 | 1218 | 0 | 0 | 0 | 0 | 0 | 0 | 0 | 0 |
| cg11188103 | 4 | 102 | 3 | 433 | 785 | 0 | 0 | 0 | 0 | 0 | 0 | 0 |
| cg11248857 | 3 | 8 | 107 | 1208 | 0 | 0 | 0 | 0 | 0 | 0 | 0 | 0 |
| cg11251367 | 3 | 720 | 1 | 602 | 0 | 0 | 0 | 0 | 0 | 0 | 0 | 0 |
| cg11404532 | 3 | 4 | 158 | 1161 | 0 | 0 | 0 | 0 | 0 | 0 | 0 | 0 |
| cg11680857 | 2 | 169 | 1154 | 0 | 0 | 0 | 0 | 0 | 0 | 0 | 0 | 0 |
| cg11702662 | 4 | 10 | 71 | 1 | 1241 | 0 | 0 | 0 | 0 | 0 | 0 | 0 |
| cg11723698 | 3 | 2 | 68 | 1253 | 0 | 0 | 0 | 0 | 0 | 0 | 0 | 0 |
| cg11733135 | 5 | 40 | 2 | 319 | 1 | 961 | 0 | 0 | 0 | 0 | 0 | 0 |
| cg11802806 | 5 | 1 | 12 | 144 | 1 | 1165 | 0 | 0 | 0 | 0 | 0 | 0 |
| cg11853970 | 3 | 2 | 75 | 1246 | 0 | 0 | 0 | 0 | 0 | 0 | 0 | 0 |
| cg12213037 | 2 | 655 | 668 | 0 | 0 | 0 | 0 | 0 | 0 | 0 | 0 | 0 |
| cg12386614 | 5 | 60 | 409 | 1 | 1 | 852 | 0 | 0 | 0 | 0 | 0 | 0 |
| cg12388523 | 3 | 134 | 2 | 1187 | 0 | 0 | 0 | 0 | 0 | 0 | 0 | 0 |
| cg12406683 | 2 | 94 | 1229 | 0 | 0 | 0 | 0 | 0 | 0 | 0 | 0 | 0 |
| cg12466610 | 6 | 496 | 2 | 1 | 1 | 555 | 268 | 0 | 0 | 0 | 0 | 0 |
| cg12761965 | 3 | 1255 | 67 | 1 | 0 | 0 | 0 | 0 | 0 | 0 | 0 | 0 |
| cg13078798 | 3 | 546 | 586 | 191 | 0 | 0 | 0 | 0 | 0 | 0 | 0 | 0 |
| cg13167158 | 4 | 22 | 2 | 283 | 1016 | 0 | 0 | 0 | 0 | 0 | 0 | 0 |
| cg13205059 | 4 | 5 | 121 | 2 | 1195 | 0 | 0 | 0 | 0 | 0 | 0 | 0 |
| cg13232075 | 3 | 402 | 532 | 389 | 0 | 0 | 0 | 0 | 0 | 0 | 0 | 0 |
| cg13239126 | 5 | 75 | 1 | 394 | 1 | 852 | 0 | 0 | 0 | 0 | 0 | 0 |
| cg13370086 | 5 | 13 | 3 | 178 | 1 | 1128 | 0 | 0 | 0 | 0 | 0 | 0 |
| cg13387643 | 4 | 187 | 1 | 516 | 619 | 0 | 0 | 0 | 0 | 0 | 0 | 0 |
| cg13462557 | 6 | 481 | 1 | 1 | 1 | 576 | 263 | 0 | 0 | 0 | 0 | 0 |
| cg13482134 | 2 | 350 | 973 | 0 | 0 | 0 | 0 | 0 | 0 | 0 | 0 | 0 |
| cg13496167 | 3 | 2 | 74 | 1247 | 0 | 0 | 0 | 0 | 0 | 0 | 0 | 0 |
| cg13688186 | 4 | 75 | 1 | 2 | 1245 | 0 | 0 | 0 | 0 | 0 | 0 | 0 |
| cg13898771 | 4 | 6 | 1 | 113 | 1203 | 0 | 0 | 0 | 0 | 0 | 0 | 0 |
| cg13928473 | 4 | 390 | 1 | 931 | 1 | 0 | 0 | 0 | 0 | 0 | 0 | 0 |
| cg13950674 | 3 | 131 | 1190 | 2 | 0 | 0 | 0 | 0 | 0 | 0 | 0 | 0 |
| cg13962846 | 4 | 124 | 3 | 4 | 1192 | 0 | 0 | 0 | 0 | 0 | 0 | 0 |
| cg13976154 | 3 | 5 | 101 | 1217 | 0 | 0 | 0 | 0 | 0 | 0 | 0 | 0 |
| cg14078368 | 2 | 98 | 1225 | 0 | 0 | 0 | 0 | 0 | 0 | 0 | 0 | 0 |
| cg14252149 | 3 | 454 | 612 | 257 | 0 | 0 | 0 | 0 | 0 | 0 | 0 | 0 |
| cg14255243 | 3 | 4 | 83 | 1236 | 0 | 0 | 0 | 0 | 0 | 0 | 0 | 0 |
| cg14378564 | 3 | 1195 | 124 | 4 | 0 | 0 | 0 | 0 | 0 | 0 | 0 | 0 |
| cg14825858 | 4 | 65 | 1 | 2 | 1255 | 0 | 0 | 0 | 0 | 0 | 0 | 0 |
| cg14859874 | 2 | 430 | 893 | 0 | 0 | 0 | 0 | 0 | 0 | 0 | 0 | 0 |
| cg15075357 | 3 | 279 | 548 | 496 | 0 | 0 | 0 | 0 | 0 | 0 | 0 | 0 |
| cg15100209 | 3 | 5 | 69 | 1249 | 0 | 0 | 0 | 0 | 0 | 0 | 0 | 0 |
| cg15198148 | 4 | 138 | 1 | 1 | 1183 | 0 | 0 | 0 | 0 | 0 | 0 | 0 |
| cg15585213 | 3 | 2 | 88 | 1233 | 0 | 0 | 0 | 0 | 0 | 0 | 0 | 0 |
| cg15600437 | 5 | 172 | 506 | 1 | 5 | 639 | 0 | 0 | 0 | 0 | 0 | 0 |
| cg15903449 | 4 | 11 | 2 | 114 | 1196 | 0 | 0 | 0 | 0 | 0 | 0 | 0 |
| cg16046605 | 3 | 122 | 3 | 1198 | 0 | 0 | 0 | 0 | 0 | 0 | 0 | 0 |
| cg16060930 | 4 | 116 | 1 | 478 | 728 | 0 | 0 | 0 | 0 | 0 | 0 | 0 |
| cg16112880 | 5 | 669 | 2 | 513 | 1 | 138 | 0 | 0 | 0 | 0 | 0 | 0 |
| cg16270643 | 5 | 21 | 1 | 212 | 1 | 1088 | 0 | 0 | 0 | 0 | 0 | 0 |
| cg16352443 | 3 | 8 | 95 | 1220 | 0 | 0 | 0 | 0 | 0 | 0 | 0 | 0 |
| cg16393182 | 4 | 6 | 1 | 80 | 1236 | 0 | 0 | 0 | 0 | 0 | 0 | 0 |
| cg16490124 | 4 | 789 | 1 | 443 | 90 | 0 | 0 | 0 | 0 | 0 | 0 | 0 |
| cg16652920 | 5 | 93 | 1 | 1 | 394 | 834 | 0 | 0 | 0 | 0 | 0 | 0 |
| cg16675581 | 5 | 33 | 1 | 1 | 356 | 932 | 0 | 0 | 0 | 0 | 0 | 0 |
| cg16675926 | 3 | 308 | 2 | 1013 | 0 | 0 | 0 | 0 | 0 | 0 | 0 | 0 |
| cg17025908 | 3 | 131 | 2 | 1190 | 0 | 0 | 0 | 0 | 0 | 0 | 0 | 0 |
| cg17096979 | 5 | 2 | 1 | 1 | 174 | 1145 | 0 | 0 | 0 | 0 | 0 | 0 |
| cg17107246 | 6 | 92 | 2 | 1 | 3 | 4 | 1221 | 0 | 0 | 0 | 0 | 0 |
| cg17143270 | 3 | 3 | 70 | 1250 | 0 | 0 | 0 | 0 | 0 | 0 | 0 | 0 |
| cg17171259 | 3 | 717 | 604 | 2 | 0 | 0 | 0 | 0 | 0 | 0 | 0 | 0 |
| cg17279365 | 4 | 161 | 530 | 627 | 5 | 0 | 0 | 0 | 0 | 0 | 0 | 0 |
| cg17744878 | 3 | 169 | 3 | 1151 | 0 | 0 | 0 | 0 | 0 | 0 | 0 | 0 |
| cg17820060 | 5 | 19 | 5 | 1 | 241 | 1057 | 0 | 0 | 0 | 0 | 0 | 0 |
| cg17821453 | 3 | 28 | 296 | 999 | 0 | 0 | 0 | 0 | 0 | 0 | 0 | 0 |
| cg18235051 | 5 | 8 | 1 | 207 | 2 | 1105 | 0 | 0 | 0 | 0 | 0 | 0 |
| cg18285337 | 3 | 283 | 620 | 420 | 0 | 0 | 0 | 0 | 0 | 0 | 0 | 0 |
| cg18346634 | 2 | 392 | 931 | 0 | 0 | 0 | 0 | 0 | 0 | 0 | 0 | 0 |
| cg18756931 | 5 | 58 | 1 | 368 | 1 | 895 | 0 | 0 | 0 | 0 | 0 | 0 |
| cg19373347 | 2 | 280 | 1043 | 0 | 0 | 0 | 0 | 0 | 0 | 0 | 0 | 0 |
| cg19405842 | 5 | 345 | 5 | 1 | 640 | 332 | 0 | 0 | 0 | 0 | 0 | 0 |
| cg19743622 | 2 | 139 | 1184 | 0 | 0 | 0 | 0 | 0 | 0 | 0 | 0 | 0 |
| cg19750824 | 3 | 182 | 2 | 1139 | 0 | 0 | 0 | 0 | 0 | 0 | 0 | 0 |
| cg20269544 | 3 | 7 | 99 | 1217 | 0 | 0 | 0 | 0 | 0 | 0 | 0 | 0 |
| cg20299670 | 2 | 135 | 1188 | 0 | 0 | 0 | 0 | 0 | 0 | 0 | 0 | 0 |
| cg20485607 | 2 | 95 | 1228 | 0 | 0 | 0 | 0 | 0 | 0 | 0 | 0 | 0 |
| cg20568108 | 7 | 4 | 1 | 1 | 1 | 92 | 1 | 1223 | 0 | 0 | 0 | 0 |
| cg20696674 | 2 | 233 | 1090 | 0 | 0 | 0 | 0 | 0 | 0 | 0 | 0 | 0 |
| cg20775316 | 3 | 65 | 1256 | 2 | 0 | 0 | 0 | 0 | 0 | 0 | 0 | 0 |
| cg20778915 | 3 | 99 | 6 | 1218 | 0 | 0 | 0 | 0 | 0 | 0 | 0 | 0 |
| cg20794208 | 3 | 82 | 2 | 1239 | 0 | 0 | 0 | 0 | 0 | 0 | 0 | 0 |
| cg20801751 | 3 | 2 | 67 | 1254 | 0 | 0 | 0 | 0 | 0 | 0 | 0 | 0 |
| cg20823859 | 4 | 407 | 1 | 565 | 350 | 0 | 0 | 0 | 0 | 0 | 0 | 0 |
| cg20960322 | 4 | 109 | 2 | 525 | 687 | 0 | 0 | 0 | 0 | 0 | 0 | 0 |
| cg20987072 | 4 | 42 | 1 | 321 | 959 | 0 | 0 | 0 | 0 | 0 | 0 | 0 |
| cg21202276 | 5 | 97 | 3 | 3 | 1 | 1219 | 0 | 0 | 0 | 0 | 0 | 0 |
| cg21245975 | 4 | 1212 | 108 | 1 | 2 | 0 | 0 | 0 | 0 | 0 | 0 | 0 |
| cg21294301 | 4 | 112 | 1 | 402 | 808 | 0 | 0 | 0 | 0 | 0 | 0 | 0 |
| cg21388339 | 2 | 657 | 666 | 0 | 0 | 0 | 0 | 0 | 0 | 0 | 0 | 0 |
| cg21397839 | 3 | 4 | 109 | 1210 | 0 | 0 | 0 | 0 | 0 | 0 | 0 | 0 |
| cg21456300 | 5 | 2 | 84 | 2 | 1 | 1234 | 0 | 0 | 0 | 0 | 0 | 0 |
| cg21783012 | 3 | 191 | 4 | 1128 | 0 | 0 | 0 | 0 | 0 | 0 | 0 | 0 |
| cg21805734 | 4 | 17 | 1 | 137 | 1168 | 0 | 0 | 0 | 0 | 0 | 0 | 0 |
| cg21826272 | 4 | 21 | 1 | 128 | 1173 | 0 | 0 | 0 | 0 | 0 | 0 | 0 |
| cg21857822 | 4 | 95 | 4 | 1 | 1223 | 0 | 0 | 0 | 0 | 0 | 0 | 0 |
| cg21860360 | 4 | 29 | 234 | 2 | 1058 | 0 | 0 | 0 | 0 | 0 | 0 | 0 |
| cg22017303 | 3 | 95 | 2 | 1226 | 0 | 0 | 0 | 0 | 0 | 0 | 0 | 0 |
| cg22167148 | 5 | 4 | 1 | 113 | 1 | 1204 | 0 | 0 | 0 | 0 | 0 | 0 |
| cg22237495 | 2 | 103 | 1220 | 0 | 0 | 0 | 0 | 0 | 0 | 0 | 0 | 0 |
| cg22337626 | 5 | 309 | 2 | 653 | 1 | 358 | 0 | 0 | 0 | 0 | 0 | 0 |
| cg22402398 | 3 | 96 | 231 | 996 | 0 | 0 | 0 | 0 | 0 | 0 | 0 | 0 |
| cg22481673 | 5 | 127 | 1 | 1 | 436 | 758 | 0 | 0 | 0 | 0 | 0 | 0 |
| cg22505202 | 4 | 109 | 2 | 560 | 652 | 0 | 0 | 0 | 0 | 0 | 0 | 0 |
| cg22626897 | 4 | 134 | 7 | 1 | 1181 | 0 | 0 | 0 | 0 | 0 | 0 | 0 |
| cg22717235 | 3 | 10 | 195 | 1118 | 0 | 0 | 0 | 0 | 0 | 0 | 0 | 0 |
| cg22802014 | 5 | 54 | 406 | 1 | 1 | 861 | 0 | 0 | 0 | 0 | 0 | 0 |
| cg23021268 | 3 | 8 | 130 | 1185 | 0 | 0 | 0 | 0 | 0 | 0 | 0 | 0 |
| cg23050079 | 4 | 5 | 101 | 1 | 1216 | 0 | 0 | 0 | 0 | 0 | 0 | 0 |
| cg23112672 | 2 | 1251 | 72 | 0 | 0 | 0 | 0 | 0 | 0 | 0 | 0 | 0 |
| cg23209941 | 4 | 123 | 1 | 1 | 1198 | 0 | 0 | 0 | 0 | 0 | 0 | 0 |
| cg23213876 | 4 | 36 | 209 | 1075 | 3 | 0 | 0 | 0 | 0 | 0 | 0 | 0 |
| cg23564471 | 4 | 78 | 1 | 385 | 859 | 0 | 0 | 0 | 0 | 0 | 0 | 0 |
| cg23586423 | 4 | 8 | 122 | 1 | 1192 | 0 | 0 | 0 | 0 | 0 | 0 | 0 |
| cg23681001 | 5 | 110 | 1 | 453 | 2 | 757 | 0 | 0 | 0 | 0 | 0 | 0 |
| cg23812489 | 2 | 140 | 1183 | 0 | 0 | 0 | 0 | 0 | 0 | 0 | 0 | 0 |
| cg23887839 | 2 | 510 | 813 | 0 | 0 | 0 | 0 | 0 | 0 | 0 | 0 | 0 |
| cg23919742 | 5 | 137 | 1 | 2 | 542 | 641 | 0 | 0 | 0 | 0 | 0 | 0 |
| cg24051749 | 3 | 155 | 548 | 620 | 0 | 0 | 0 | 0 | 0 | 0 | 0 | 0 |
| cg24088508 | 2 | 646 | 677 | 0 | 0 | 0 | 0 | 0 | 0 | 0 | 0 | 0 |
| cg24117504 | 7 | 41 | 1 | 2 | 1 | 246 | 1 | 1031 | 0 | 0 | 0 | 0 |
| cg24345856 | 4 | 65 | 1 | 385 | 872 | 0 | 0 | 0 | 0 | 0 | 0 | 0 |
| cg24529615 | 5 | 162 | 3 | 363 | 2 | 793 | 0 | 0 | 0 | 0 | 0 | 0 |
| cg24534774 | 3 | 274 | 634 | 415 | 0 | 0 | 0 | 0 | 0 | 0 | 0 | 0 |
| cg24586205 | 4 | 545 | 2 | 774 | 2 | 0 | 0 | 0 | 0 | 0 | 0 | 0 |
| cg24699914 | 3 | 128 | 1 | 1194 | 0 | 0 | 0 | 0 | 0 | 0 | 0 | 0 |
| cg24846009 | 4 | 117 | 1 | 481 | 724 | 0 | 0 | 0 | 0 | 0 | 0 | 0 |
| cg24925741 | 5 | 553 | 582 | 1 | 186 | 1 | 0 | 0 | 0 | 0 | 0 | 0 |
| cg25150572 | 4 | 233 | 5 | 1 | 1084 | 0 | 0 | 0 | 0 | 0 | 0 | 0 |
| cg25280720 | 3 | 3 | 76 | 1244 | 0 | 0 | 0 | 0 | 0 | 0 | 0 | 0 |
| cg25282454 | 3 | 43 | 311 | 969 | 0 | 0 | 0 | 0 | 0 | 0 | 0 | 0 |
| cg25307521 | 3 | 15 | 199 | 1109 | 0 | 0 | 0 | 0 | 0 | 0 | 0 | 0 |
| cg25465065 | 3 | 176 | 454 | 693 | 0 | 0 | 0 | 0 | 0 | 0 | 0 | 0 |
| cg25593194 | 3 | 337 | 3 | 983 | 0 | 0 | 0 | 0 | 0 | 0 | 0 | 0 |
| cg25771854 | 3 | 79 | 1 | 1243 | 0 | 0 | 0 | 0 | 0 | 0 | 0 | 0 |
| cg26035071 | 7 | 173 | 2 | 1 | 1 | 532 | 1 | 613 | 0 | 0 | 0 | 0 |
| cg26300135 | 4 | 3 | 63 | 2 | 1255 | 0 | 0 | 0 | 0 | 0 | 0 | 0 |
| cg26303777 | 4 | 117 | 464 | 1 | 741 | 0 | 0 | 0 | 0 | 0 | 0 | 0 |
| cg26346563 | 2 | 106 | 1217 | 0 | 0 | 0 | 0 | 0 | 0 | 0 | 0 | 0 |
| cg26348696 | 4 | 18 | 229 | 3 | 1073 | 0 | 0 | 0 | 0 | 0 | 0 | 0 |
| cg26400546 | 3 | 74 | 2 | 1247 | 0 | 0 | 0 | 0 | 0 | 0 | 0 | 0 |
| cg26422465 | 5 | 424 | 1 | 589 | 2 | 307 | 0 | 0 | 0 | 0 | 0 | 0 |
| cg26570389 | 3 | 4 | 129 | 1190 | 0 | 0 | 0 | 0 | 0 | 0 | 0 | 0 |
| cg26659797 | 4 | 2 | 1 | 138 | 1182 | 0 | 0 | 0 | 0 | 0 | 0 | 0 |
| cg26785303 | 3 | 17 | 193 | 1113 | 0 | 0 | 0 | 0 | 0 | 0 | 0 | 0 |
| cg26824678 | 4 | 78 | 532 | 2 | 711 | 0 | 0 | 0 | 0 | 0 | 0 | 0 |
| cg26883836 | 5 | 3 | 1 | 1 | 113 | 1205 | 0 | 0 | 0 | 0 | 0 | 0 |
| cg26908356 | 4 | 173 | 1 | 2 | 1147 | 0 | 0 | 0 | 0 | 0 | 0 | 0 |
| cg26924218 | 4 | 2 | 2 | 76 | 1243 | 0 | 0 | 0 | 0 | 0 | 0 | 0 |
| cg27183036 | 3 | 4 | 103 | 1216 | 0 | 0 | 0 | 0 | 0 | 0 | 0 | 0 |
| cg27399895 | 2 | 122 | 1201 | 0 | 0 | 0 | 0 | 0 | 0 | 0 | 0 | 0 |
| cg27433479 | 5 | 204 | 1 | 2 | 1 | 1115 | 0 | 0 | 0 | 0 | 0 | 0 |
| cg27452255 | 3 | 68 | 1 | 1254 | 0 | 0 | 0 | 0 | 0 | 0 | 0 | 0 |
| cg27518860 | 3 | 1 | 70 | 1252 | 0 | 0 | 0 | 0 | 0 | 0 | 0 | 0 |
| cg27567580 | 3 | 198 | 6 | 1119 | 0 | 0 | 0 | 0 | 0 | 0 | 0 | 0 |
| cg00004073 | 4 | 159 | 2 | 1161 | 1 | 0 | 0 | 0 | 0 | 0 | 0 | 0 |
| cg00009523 | 5 | 200 | 1 | 565 | 1 | 556 | 0 | 0 | 0 | 0 | 0 | 0 |
| cg00040530 | 5 | 66 | 1 | 1 | 1 | 1254 | 0 | 0 | 0 | 0 | 0 | 0 |
| cg00051154 | 5 | 873 | 401 | 1 | 1 | 47 | 0 | 0 | 0 | 0 | 0 | 0 |
| cg00257789 | 5 | 579 | 1 | 1 | 591 | 151 | 0 | 0 | 0 | 0 | 0 | 0 |
| cg00424152 | 3 | 72 | 414 | 837 | 0 | 0 | 0 | 0 | 0 | 0 | 0 | 0 |
| cg00474373 | 3 | 232 | 2 | 1089 | 0 | 0 | 0 | 0 | 0 | 0 | 0 | 0 |
| cg00570635 | 3 | 728 | 594 | 1 | 0 | 0 | 0 | 0 | 0 | 0 | 0 | 0 |
| cg00684178 | 3 | 57 | 10 | 1256 | 0 | 0 | 0 | 0 | 0 | 0 | 0 | 0 |
| cg00686453 | 4 | 106 | 2 | 2 | 1213 | 0 | 0 | 0 | 0 | 0 | 0 | 0 |
| cg00745855 | 4 | 7 | 102 | 1 | 1213 | 0 | 0 | 0 | 0 | 0 | 0 | 0 |
| cg00881894 | 4 | 72 | 4 | 473 | 774 | 0 | 0 | 0 | 0 | 0 | 0 | 0 |
| cg00980980 | 3 | 69 | 362 | 892 | 0 | 0 | 0 | 0 | 0 | 0 | 0 | 0 |
| cg01013522 | 4 | 134 | 3 | 1184 | 2 | 0 | 0 | 0 | 0 | 0 | 0 | 0 |
| cg01111179 | 6 | 22 | 1 | 1 | 306 | 987 | 6 | 0 | 0 | 0 | 0 | 0 |
| cg01188578 | 4 | 449 | 1 | 1 | 872 | 0 | 0 | 0 | 0 | 0 | 0 | 0 |
| cg01397495 | 4 | 31 | 302 | 987 | 3 | 0 | 0 | 0 | 0 | 0 | 0 | 0 |
| cg01592350 | 5 | 19 | 14 | 114 | 1 | 1175 | 0 | 0 | 0 | 0 | 0 | 0 |
| cg01594260 | 3 | 451 | 502 | 370 | 0 | 0 | 0 | 0 | 0 | 0 | 0 | 0 |
| cg01695532 | 3 | 11 | 173 | 1139 | 0 | 0 | 0 | 0 | 0 | 0 | 0 | 0 |
| cg01783736 | 2 | 208 | 1115 | 0 | 0 | 0 | 0 | 0 | 0 | 0 | 0 | 0 |
| cg01957222 | 5 | 48 | 2 | 427 | 2 | 844 | 0 | 0 | 0 | 0 | 0 | 0 |
| cg01957712 | 4 | 1 | 66 | 1 | 1255 | 0 | 0 | 0 | 0 | 0 | 0 | 0 |
| cg02139050 | 6 | 3 | 1 | 1 | 92 | 2 | 1224 | 0 | 0 | 0 | 0 | 0 |
| cg02448395 | 4 | 6 | 143 | 1 | 1173 | 0 | 0 | 0 | 0 | 0 | 0 | 0 |
| cg02458875 | 4 | 10 | 2 | 245 | 1066 | 0 | 0 | 0 | 0 | 0 | 0 | 0 |
| cg02502145 | 4 | 839 | 1 | 373 | 110 | 0 | 0 | 0 | 0 | 0 | 0 | 0 |
| cg02527811 | 4 | 83 | 325 | 1 | 914 | 0 | 0 | 0 | 0 | 0 | 0 | 0 |
| cg02529760 | 3 | 75 | 1 | 1247 | 0 | 0 | 0 | 0 | 0 | 0 | 0 | 0 |
| cg02741327 | 4 | 155 | 1 | 1 | 1166 | 0 | 0 | 0 | 0 | 0 | 0 | 0 |
| cg02745784 | 3 | 65 | 2 | 1256 | 0 | 0 | 0 | 0 | 0 | 0 | 0 | 0 |
| cg02819655 | 4 | 290 | 2 | 527 | 504 | 0 | 0 | 0 | 0 | 0 | 0 | 0 |
| cg02823625 | 3 | 20 | 249 | 1054 | 0 | 0 | 0 | 0 | 0 | 0 | 0 | 0 |
| cg02943411 | 5 | 5 | 2 | 87 | 1 | 1228 | 0 | 0 | 0 | 0 | 0 | 0 |
| cg03024489 | 3 | 8 | 88 | 1227 | 0 | 0 | 0 | 0 | 0 | 0 | 0 | 0 |
| cg03028786 | 4 | 36 | 1 | 338 | 948 | 0 | 0 | 0 | 0 | 0 | 0 | 0 |
| cg03075631 | 4 | 118 | 238 | 2 | 965 | 0 | 0 | 0 | 0 | 0 | 0 | 0 |
| cg03167407 | 2 | 181 | 1142 | 0 | 0 | 0 | 0 | 0 | 0 | 0 | 0 | 0 |
| cg03206401 | 2 | 236 | 1087 | 0 | 0 | 0 | 0 | 0 | 0 | 0 | 0 | 0 |
| cg03428619 | 2 | 70 | 1253 | 0 | 0 | 0 | 0 | 0 | 0 | 0 | 0 | 0 |
| cg03549208 | 4 | 157 | 4 | 2 | 1160 | 0 | 0 | 0 | 0 | 0 | 0 | 0 |
| cg03565013 | 5 | 85 | 1 | 1 | 2 | 1234 | 0 | 0 | 0 | 0 | 0 | 0 |
| cg03886523 | 3 | 7 | 73 | 1243 | 0 | 0 | 0 | 0 | 0 | 0 | 0 | 0 |
| cg03987818 | 5 | 116 | 1 | 1 | 1 | 1204 | 0 | 0 | 0 | 0 | 0 | 0 |
| cg04003990 | 3 | 45 | 350 | 928 | 0 | 0 | 0 | 0 | 0 | 0 | 0 | 0 |
| cg04033559 | 3 | 152 | 1 | 1170 | 0 | 0 | 0 | 0 | 0 | 0 | 0 | 0 |
| cg04066495 | 2 | 309 | 1014 | 0 | 0 | 0 | 0 | 0 | 0 | 0 | 0 | 0 |
| cg04131969 | 3 | 293 | 628 | 402 | 0 | 0 | 0 | 0 | 0 | 0 | 0 | 0 |
| cg04316537 | 3 | 111 | 3 | 1209 | 0 | 0 | 0 | 0 | 0 | 0 | 0 | 0 |
| cg04459585 | 5 | 2 | 19 | 1 | 178 | 1123 | 0 | 0 | 0 | 0 | 0 | 0 |
| cg04506342 | 2 | 228 | 1095 | 0 | 0 | 0 | 0 | 0 | 0 | 0 | 0 | 0 |
| cg04508606 | 3 | 99 | 1 | 1223 | 0 | 0 | 0 | 0 | 0 | 0 | 0 | 0 |
| cg04541421 | 3 | 93 | 2 | 1228 | 0 | 0 | 0 | 0 | 0 | 0 | 0 | 0 |
| cg04951549 | 3 | 1239 | 83 | 1 | 0 | 0 | 0 | 0 | 0 | 0 | 0 | 0 |
| cg05023192 | 3 | 313 | 1 | 1009 | 0 | 0 | 0 | 0 | 0 | 0 | 0 | 0 |
| cg05092371 | 4 | 32 | 1 | 368 | 922 | 0 | 0 | 0 | 0 | 0 | 0 | 0 |
| cg05513157 | 4 | 8 | 117 | 1 | 1197 | 0 | 0 | 0 | 0 | 0 | 0 | 0 |
| cg05708441 | 2 | 226 | 1097 | 0 | 0 | 0 | 0 | 0 | 0 | 0 | 0 | 0 |
| cg05737004 | 5 | 12 | 1 | 119 | 1 | 1190 | 0 | 0 | 0 | 0 | 0 | 0 |
| cg05755715 | 4 | 22 | 2 | 175 | 1124 | 0 | 0 | 0 | 0 | 0 | 0 | 0 |
| cg05971102 | 4 | 309 | 2 | 611 | 401 | 0 | 0 | 0 | 0 | 0 | 0 | 0 |
| cg05995465 | 2 | 375 | 948 | 0 | 0 | 0 | 0 | 0 | 0 | 0 | 0 | 0 |
| cg06125229 | 2 | 78 | 1245 | 0 | 0 | 0 | 0 | 0 | 0 | 0 | 0 | 0 |
| cg06225639 | 4 | 1 | 381 | 4 | 937 | 0 | 0 | 0 | 0 | 0 | 0 | 0 |
| cg06264882 | 4 | 179 | 1 | 549 | 594 | 0 | 0 | 0 | 0 | 0 | 0 | 0 |
| cg06430688 | 3 | 38 | 373 | 912 | 0 | 0 | 0 | 0 | 0 | 0 | 0 | 0 |
| cg06589199 | 3 | 12 | 117 | 1194 | 0 | 0 | 0 | 0 | 0 | 0 | 0 | 0 |
| cg06703213 | 3 | 25 | 153 | 1145 | 0 | 0 | 0 | 0 | 0 | 0 | 0 | 0 |
| cg06737250 | 5 | 343 | 1 | 1 | 609 | 369 | 0 | 0 | 0 | 0 | 0 | 0 |
| cg06982827 | 6 | 2 | 2 | 1 | 61 | 1 | 1256 | 0 | 0 | 0 | 0 | 0 |
| cg07025003 | 5 | 11 | 1 | 1 | 92 | 1218 | 0 | 0 | 0 | 0 | 0 | 0 |
| cg07227024 | 2 | 411 | 912 | 0 | 0 | 0 | 0 | 0 | 0 | 0 | 0 | 0 |
| cg07287078 | 3 | 36 | 219 | 1068 | 0 | 0 | 0 | 0 | 0 | 0 | 0 | 0 |
| cg07319199 | 5 | 191 | 2 | 478 | 1 | 651 | 0 | 0 | 0 | 0 | 0 | 0 |
| cg07646083 | 4 | 41 | 235 | 2 | 1045 | 0 | 0 | 0 | 0 | 0 | 0 | 0 |
| cg08210468 | 2 | 410 | 913 | 0 | 0 | 0 | 0 | 0 | 0 | 0 | 0 | 0 |
| cg08318587 | 4 | 1170 | 1 | 136 | 16 | 0 | 0 | 0 | 0 | 0 | 0 | 0 |
| cg08520690 | 4 | 5 | 1 | 120 | 1197 | 0 | 0 | 0 | 0 | 0 | 0 | 0 |
| cg08537127 | 2 | 229 | 1094 | 0 | 0 | 0 | 0 | 0 | 0 | 0 | 0 | 0 |
| cg08551408 | 4 | 80 | 2 | 1 | 1240 | 0 | 0 | 0 | 0 | 0 | 0 | 0 |
| cg08963013 | 5 | 198 | 1 | 1 | 522 | 601 | 0 | 0 | 0 | 0 | 0 | 0 |
| cg09167828 | 4 | 142 | 265 | 5 | 911 | 0 | 0 | 0 | 0 | 0 | 0 | 0 |
| cg09289202 | 4 | 481 | 1 | 575 | 266 | 0 | 0 | 0 | 0 | 0 | 0 | 0 |
| cg09714315 | 5 | 126 | 1 | 1 | 1193 | 2 | 0 | 0 | 0 | 0 | 0 | 0 |
| cg09921423 | 3 | 27 | 155 | 1141 | 0 | 0 | 0 | 0 | 0 | 0 | 0 | 0 |
| cg10224537 | 6 | 54 | 1 | 1 | 1 | 303 | 963 | 0 | 0 | 0 | 0 | 0 |
| cg10263003 | 3 | 123 | 2 | 1198 | 0 | 0 | 0 | 0 | 0 | 0 | 0 | 0 |
| cg10280035 | 3 | 74 | 1 | 1248 | 0 | 0 | 0 | 0 | 0 | 0 | 0 | 0 |
| cg10359347 | 4 | 14 | 160 | 1 | 1148 | 0 | 0 | 0 | 0 | 0 | 0 | 0 |
| cg10610477 | 3 | 8 | 140 | 1175 | 0 | 0 | 0 | 0 | 0 | 0 | 0 | 0 |
| cg10724632 | 5 | 352 | 536 | 1 | 433 | 1 | 0 | 0 | 0 | 0 | 0 | 0 |
| cg10750264 | 4 | 67 | 1 | 1 | 1254 | 0 | 0 | 0 | 0 | 0 | 0 | 0 |
| cg10879348 | 2 | 340 | 983 | 0 | 0 | 0 | 0 | 0 | 0 | 0 | 0 | 0 |
| cg11015768 | 3 | 3 | 92 | 1228 | 0 | 0 | 0 | 0 | 0 | 0 | 0 | 0 |
| cg11016420 | 6 | 43 | 1 | 1 | 1 | 385 | 892 | 0 | 0 | 0 | 0 | 0 |
| cg11229715 | 4 | 770 | 1 | 445 | 107 | 0 | 0 | 0 | 0 | 0 | 0 | 0 |
| cg11716267 | 3 | 488 | 1 | 834 | 0 | 0 | 0 | 0 | 0 | 0 | 0 | 0 |
| cg12045080 | 3 | 5 | 65 | 1253 | 0 | 0 | 0 | 0 | 0 | 0 | 0 | 0 |
| cg12074150 | 2 | 829 | 494 | 0 | 0 | 0 | 0 | 0 | 0 | 0 | 0 | 0 |
| cg12342501 | 4 | 123 | 1 | 592 | 607 | 0 | 0 | 0 | 0 | 0 | 0 | 0 |
| cg12349837 | 4 | 1 | 1 | 73 | 1248 | 0 | 0 | 0 | 0 | 0 | 0 | 0 |
| cg12390014 | 5 | 3 | 1 | 1 | 86 | 1232 | 0 | 0 | 0 | 0 | 0 | 0 |
| cg12624040 | 5 | 138 | 3 | 1 | 1 | 1180 | 0 | 0 | 0 | 0 | 0 | 0 |
| cg13167631 | 2 | 71 | 1252 | 0 | 0 | 0 | 0 | 0 | 0 | 0 | 0 | 0 |
| cg13215060 | 4 | 67 | 2 | 465 | 789 | 0 | 0 | 0 | 0 | 0 | 0 | 0 |
| cg13219409 | 3 | 95 | 7 | 1221 | 0 | 0 | 0 | 0 | 0 | 0 | 0 | 0 |
| cg13226272 | 4 | 896 | 4 | 378 | 45 | 0 | 0 | 0 | 0 | 0 | 0 | 0 |
| cg13649600 | 4 | 16 | 1 | 126 | 1180 | 0 | 0 | 0 | 0 | 0 | 0 | 0 |
| cg13821051 | 4 | 180 | 1 | 544 | 598 | 0 | 0 | 0 | 0 | 0 | 0 | 0 |
| cg13920792 | 3 | 4 | 81 | 1238 | 0 | 0 | 0 | 0 | 0 | 0 | 0 | 0 |
| cg14183136 | 2 | 85 | 1238 | 0 | 0 | 0 | 0 | 0 | 0 | 0 | 0 | 0 |
| cg14252237 | 5 | 5 | 116 | 2 | 2 | 1198 | 0 | 0 | 0 | 0 | 0 | 0 |
| cg14271023 | 3 | 179 | 429 | 715 | 0 | 0 | 0 | 0 | 0 | 0 | 0 | 0 |
| cg14506194 | 3 | 140 | 1181 | 2 | 0 | 0 | 0 | 0 | 0 | 0 | 0 | 0 |
| cg14548901 | 4 | 215 | 4 | 2 | 1102 | 0 | 0 | 0 | 0 | 0 | 0 | 0 |
| cg14691886 | 3 | 8 | 69 | 1246 | 0 | 0 | 0 | 0 | 0 | 0 | 0 | 0 |
| cg14795227 | 3 | 149 | 1 | 1173 | 0 | 0 | 0 | 0 | 0 | 0 | 0 | 0 |
| cg14883135 | 3 | 23 | 282 | 1018 | 0 | 0 | 0 | 0 | 0 | 0 | 0 | 0 |
| cg14994124 | 6 | 3 | 1 | 1 | 1 | 85 | 1232 | 0 | 0 | 0 | 0 | 0 |
| cg15150396 | 5 | 1 | 213 | 3 | 1 | 1105 | 0 | 0 | 0 | 0 | 0 | 0 |
| cg15177604 | 4 | 918 | 361 | 1 | 43 | 0 | 0 | 0 | 0 | 0 | 0 | 0 |
| cg15518264 | 4 | 81 | 6 | 1 | 1235 | 0 | 0 | 0 | 0 | 0 | 0 | 0 |
| cg15770150 | 5 | 3 | 1 | 3 | 68 | 1248 | 0 | 0 | 0 | 0 | 0 | 0 |
| cg15837943 | 2 | 324 | 999 | 0 | 0 | 0 | 0 | 0 | 0 | 0 | 0 | 0 |
| cg15840554 | 5 | 72 | 1 | 1 | 165 | 1084 | 0 | 0 | 0 | 0 | 0 | 0 |
| cg15958422 | 2 | 113 | 1210 | 0 | 0 | 0 | 0 | 0 | 0 | 0 | 0 | 0 |
| cg16331745 | 2 | 129 | 1194 | 0 | 0 | 0 | 0 | 0 | 0 | 0 | 0 | 0 |
| cg16403360 | 3 | 219 | 1103 | 1 | 0 | 0 | 0 | 0 | 0 | 0 | 0 | 0 |
| cg16512708 | 3 | 1151 | 153 | 19 | 0 | 0 | 0 | 0 | 0 | 0 | 0 | 0 |
| cg16519574 | 5 | 48 | 1 | 283 | 1 | 990 | 0 | 0 | 0 | 0 | 0 | 0 |
| cg16678169 | 3 | 1112 | 188 | 23 | 0 | 0 | 0 | 0 | 0 | 0 | 0 | 0 |
| cg16908938 | 4 | 133 | 1 | 1188 | 1 | 0 | 0 | 0 | 0 | 0 | 0 | 0 |
| cg16995742 | 2 | 465 | 858 | 0 | 0 | 0 | 0 | 0 | 0 | 0 | 0 | 0 |
| cg17056069 | 4 | 87 | 1 | 382 | 853 | 0 | 0 | 0 | 0 | 0 | 0 | 0 |
| cg17149911 | 5 | 530 | 1 | 1 | 539 | 252 | 0 | 0 | 0 | 0 | 0 | 0 |
| cg17174466 | 4 | 228 | 5 | 2 | 1088 | 0 | 0 | 0 | 0 | 0 | 0 | 0 |
| cg17181941 | 5 | 58 | 1 | 1 | 391 | 872 | 0 | 0 | 0 | 0 | 0 | 0 |
| cg17240976 | 5 | 41 | 332 | 1 | 948 | 1 | 0 | 0 | 0 | 0 | 0 | 0 |
| cg17248267 | 4 | 47 | 227 | 1 | 1048 | 0 | 0 | 0 | 0 | 0 | 0 | 0 |
| cg17283620 | 5 | 605 | 571 | 1 | 145 | 1 | 0 | 0 | 0 | 0 | 0 | 0 |
| cg17331757 | 4 | 64 | 1 | 3 | 1255 | 0 | 0 | 0 | 0 | 0 | 0 | 0 |
| cg17363276 | 4 | 12 | 1 | 88 | 1222 | 0 | 0 | 0 | 0 | 0 | 0 | 0 |
| cg17455348 | 5 | 321 | 1 | 2 | 997 | 2 | 0 | 0 | 0 | 0 | 0 | 0 |
| cg17588704 | 2 | 251 | 1072 | 0 | 0 | 0 | 0 | 0 | 0 | 0 | 0 | 0 |
| cg17600943 | 4 | 158 | 1 | 606 | 558 | 0 | 0 | 0 | 0 | 0 | 0 | 0 |
| cg17717333 | 4 | 1205 | 116 | 1 | 1 | 0 | 0 | 0 | 0 | 0 | 0 | 0 |
| cg17977362 | 4 | 9 | 166 | 1 | 1147 | 0 | 0 | 0 | 0 | 0 | 0 | 0 |
| cg18015625 | 2 | 174 | 1149 | 0 | 0 | 0 | 0 | 0 | 0 | 0 | 0 | 0 |
| cg18121901 | 3 | 28 | 327 | 968 | 0 | 0 | 0 | 0 | 0 | 0 | 0 | 0 |
| cg18172516 | 3 | 145 | 1 | 1177 | 0 | 0 | 0 | 0 | 0 | 0 | 0 | 0 |
| cg18368960 | 2 | 155 | 1168 | 0 | 0 | 0 | 0 | 0 | 0 | 0 | 0 | 0 |
| cg18402783 | 4 | 6 | 83 | 1 | 1233 | 0 | 0 | 0 | 0 | 0 | 0 | 0 |
| cg18548165 | 3 | 23 | 99 | 1201 | 0 | 0 | 0 | 0 | 0 | 0 | 0 | 0 |
| cg18584561 | 4 | 581 | 1 | 579 | 162 | 0 | 0 | 0 | 0 | 0 | 0 | 0 |
| cg18662228 | 3 | 370 | 2 | 951 | 0 | 0 | 0 | 0 | 0 | 0 | 0 | 0 |
| cg18663897 | 5 | 16 | 1 | 311 | 3 | 992 | 0 | 0 | 0 | 0 | 0 | 0 |
| cg18959065 | 3 | 4 | 114 | 1205 | 0 | 0 | 0 | 0 | 0 | 0 | 0 | 0 |
| cg19187465 | 3 | 14 | 212 | 1097 | 0 | 0 | 0 | 0 | 0 | 0 | 0 | 0 |
| cg19285525 | 4 | 425 | 2 | 1 | 895 | 0 | 0 | 0 | 0 | 0 | 0 | 0 |
| cg19367293 | 3 | 17 | 235 | 1071 | 0 | 0 | 0 | 0 | 0 | 0 | 0 | 0 |
| cg19384241 | 6 | 109 | 2 | 1 | 1 | 519 | 691 | 0 | 0 | 0 | 0 | 0 |
| cg19491221 | 4 | 5 | 75 | 1 | 1242 | 0 | 0 | 0 | 0 | 0 | 0 | 0 |
| cg19697575 | 3 | 499 | 1 | 823 | 0 | 0 | 0 | 0 | 0 | 0 | 0 | 0 |
| cg19964403 | 3 | 66 | 1 | 1256 | 0 | 0 | 0 | 0 | 0 | 0 | 0 | 0 |
| cg20126741 | 4 | 5 | 1 | 127 | 1190 | 0 | 0 | 0 | 0 | 0 | 0 | 0 |
| cg20300784 | 6 | 106 | 2 | 1 | 425 | 1 | 788 | 0 | 0 | 0 | 0 | 0 |
| cg20396053 | 2 | 95 | 1228 | 0 | 0 | 0 | 0 | 0 | 0 | 0 | 0 | 0 |
| cg20421295 | 4 | 14 | 1 | 179 | 1129 | 0 | 0 | 0 | 0 | 0 | 0 | 0 |
| cg20685672 | 3 | 78 | 1 | 1244 | 0 | 0 | 0 | 0 | 0 | 0 | 0 | 0 |
| cg20695032 | 6 | 3 | 1 | 1 | 62 | 2 | 1254 | 0 | 0 | 0 | 0 | 0 |
| cg20704963 | 3 | 10 | 208 | 1105 | 0 | 0 | 0 | 0 | 0 | 0 | 0 | 0 |
| cg21037527 | 3 | 6 | 66 | 1251 | 0 | 0 | 0 | 0 | 0 | 0 | 0 | 0 |
| cg21446655 | 2 | 85 | 1238 | 0 | 0 | 0 | 0 | 0 | 0 | 0 | 0 | 0 |
| cg21514997 | 4 | 990 | 1 | 273 | 59 | 0 | 0 | 0 | 0 | 0 | 0 | 0 |
| cg21589417 | 4 | 242 | 1 | 1 | 1079 | 0 | 0 | 0 | 0 | 0 | 0 | 0 |
| cg21598489 | 5 | 21 | 1 | 330 | 1 | 970 | 0 | 0 | 0 | 0 | 0 | 0 |
| cg22140756 | 4 | 2 | 87 | 2 | 1232 | 0 | 0 | 0 | 0 | 0 | 0 | 0 |
| cg22276800 | 4 | 406 | 668 | 1 | 248 | 0 | 0 | 0 | 0 | 0 | 0 | 0 |
| cg22304519 | 4 | 344 | 2 | 593 | 384 | 0 | 0 | 0 | 0 | 0 | 0 | 0 |
| cg22305850 | 2 | 154 | 1169 | 0 | 0 | 0 | 0 | 0 | 0 | 0 | 0 | 0 |
| cg22767953 | 5 | 1 | 1 | 73 | 3 | 1245 | 0 | 0 | 0 | 0 | 0 | 0 |
| cg23128510 | 2 | 168 | 1155 | 0 | 0 | 0 | 0 | 0 | 0 | 0 | 0 | 0 |
| cg23148596 | 4 | 3 | 65 | 1 | 1254 | 0 | 0 | 0 | 0 | 0 | 0 | 0 |
| cg23233416 | 2 | 234 | 1089 | 0 | 0 | 0 | 0 | 0 | 0 | 0 | 0 | 0 |
| cg23392381 | 5 | 230 | 2 | 1 | 549 | 541 | 0 | 0 | 0 | 0 | 0 | 0 |
| cg23421392 | 3 | 325 | 4 | 994 | 0 | 0 | 0 | 0 | 0 | 0 | 0 | 0 |
| cg23482839 | 3 | 8 | 168 | 1147 | 0 | 0 | 0 | 0 | 0 | 0 | 0 | 0 |
| cg23648923 | 5 | 1 | 1 | 1 | 69 | 1251 | 0 | 0 | 0 | 0 | 0 | 0 |
| cg23813394 | 4 | 207 | 595 | 2 | 519 | 0 | 0 | 0 | 0 | 0 | 0 | 0 |
| cg23885472 | 4 | 35 | 1 | 245 | 1042 | 0 | 0 | 0 | 0 | 0 | 0 | 0 |
| cg23905015 | 3 | 1 | 678 | 644 | 0 | 0 | 0 | 0 | 0 | 0 | 0 | 0 |
| cg23947654 | 2 | 81 | 1242 | 0 | 0 | 0 | 0 | 0 | 0 | 0 | 0 | 0 |
| cg24305906 | 3 | 67 | 1 | 1255 | 0 | 0 | 0 | 0 | 0 | 0 | 0 | 0 |
| cg24543538 | 3 | 3 | 67 | 1253 | 0 | 0 | 0 | 0 | 0 | 0 | 0 | 0 |
| cg24588468 | 4 | 11 | 1 | 209 | 1102 | 0 | 0 | 0 | 0 | 0 | 0 | 0 |
| cg24906015 | 4 | 326 | 1 | 2 | 994 | 0 | 0 | 0 | 0 | 0 | 0 | 0 |
| cg24917131 | 3 | 128 | 1 | 1194 | 0 | 0 | 0 | 0 | 0 | 0 | 0 | 0 |
| cg25074023 | 3 | 1224 | 95 | 4 | 0 | 0 | 0 | 0 | 0 | 0 | 0 | 0 |
| cg25448687 | 3 | 1 | 85 | 1237 | 0 | 0 | 0 | 0 | 0 | 0 | 0 | 0 |
| cg25550823 | 3 | 22 | 230 | 1071 | 0 | 0 | 0 | 0 | 0 | 0 | 0 | 0 |
| cg25570222 | 5 | 28 | 1 | 1 | 167 | 1126 | 0 | 0 | 0 | 0 | 0 | 0 |
| cg25587481 | 3 | 24 | 312 | 987 | 0 | 0 | 0 | 0 | 0 | 0 | 0 | 0 |
| cg25951717 | 3 | 58 | 444 | 821 | 0 | 0 | 0 | 0 | 0 | 0 | 0 | 0 |
| cg26179400 | 5 | 18 | 3 | 203 | 2 | 1097 | 0 | 0 | 0 | 0 | 0 | 0 |
| cg26677549 | 3 | 4 | 104 | 1215 | 0 | 0 | 0 | 0 | 0 | 0 | 0 | 0 |
| cg26881691 | 4 | 43 | 4 | 300 | 976 | 0 | 0 | 0 | 0 | 0 | 0 | 0 |
| cg26990681 | 3 | 2 | 70 | 1251 | 0 | 0 | 0 | 0 | 0 | 0 | 0 | 0 |
| cg26995506 | 5 | 780 | 1 | 459 | 1 | 82 | 0 | 0 | 0 | 0 | 0 | 0 |
| cg00779294 | 4 | 3 | 1 | 67 | 1252 | 0 | 0 | 0 | 0 | 0 | 0 | 0 |
| cg01055691 | 2 | 132 | 1191 | 0 | 0 | 0 | 0 | 0 | 0 | 0 | 0 | 0 |
| cg01347786 | 3 | 6 | 213 | 1104 | 0 | 0 | 0 | 0 | 0 | 0 | 0 | 0 |
| cg01388693 | 3 | 163 | 522 | 638 | 0 | 0 | 0 | 0 | 0 | 0 | 0 | 0 |
| cg01883054 | 3 | 9 | 183 | 1131 | 0 | 0 | 0 | 0 | 0 | 0 | 0 | 0 |
| cg02262162 | 6 | 16 | 1 | 1 | 2 | 190 | 1113 | 0 | 0 | 0 | 0 | 0 |
| cg02507579 | 2 | 506 | 817 | 0 | 0 | 0 | 0 | 0 | 0 | 0 | 0 | 0 |
| cg02648923 | 4 | 63 | 242 | 1 | 1017 | 0 | 0 | 0 | 0 | 0 | 0 | 0 |
| cg02794920 | 3 | 10 | 122 | 1191 | 0 | 0 | 0 | 0 | 0 | 0 | 0 | 0 |
| cg03269667 | 3 | 4 | 65 | 1254 | 0 | 0 | 0 | 0 | 0 | 0 | 0 | 0 |
| cg03275851 | 4 | 18 | 149 | 1155 | 1 | 0 | 0 | 0 | 0 | 0 | 0 | 0 |
| cg03329597 | 3 | 801 | 427 | 95 | 0 | 0 | 0 | 0 | 0 | 0 | 0 | 0 |
| cg03432589 | 5 | 33 | 1 | 1 | 329 | 959 | 0 | 0 | 0 | 0 | 0 | 0 |
| cg03455225 | 3 | 3 | 151 | 1169 | 0 | 0 | 0 | 0 | 0 | 0 | 0 | 0 |
| cg03761891 | 5 | 3 | 1 | 85 | 2 | 1232 | 0 | 0 | 0 | 0 | 0 | 0 |
| cg04103706 | 4 | 3 | 77 | 1 | 1242 | 0 | 0 | 0 | 0 | 0 | 0 | 0 |
| cg04156077 | 3 | 154 | 1 | 1168 | 0 | 0 | 0 | 0 | 0 | 0 | 0 | 0 |
| cg04232098 | 4 | 2 | 5 | 73 | 1243 | 0 | 0 | 0 | 0 | 0 | 0 | 0 |
| cg04461143 | 2 | 145 | 1178 | 0 | 0 | 0 | 0 | 0 | 0 | 0 | 0 | 0 |
| cg04507495 | 4 | 1252 | 2 | 64 | 5 | 0 | 0 | 0 | 0 | 0 | 0 | 0 |
| cg04528326 | 4 | 117 | 16 | 1 | 1189 | 0 | 0 | 0 | 0 | 0 | 0 | 0 |
| cg04814784 | 4 | 463 | 2 | 587 | 271 | 0 | 0 | 0 | 0 | 0 | 0 | 0 |
| cg04872879 | 3 | 1 | 88 | 1234 | 0 | 0 | 0 | 0 | 0 | 0 | 0 | 0 |
| cg05084668 | 2 | 140 | 1183 | 0 | 0 | 0 | 0 | 0 | 0 | 0 | 0 | 0 |
| cg05126514 | 5 | 129 | 1 | 470 | 722 | 1 | 0 | 0 | 0 | 0 | 0 | 0 |
| cg05393861 | 4 | 153 | 523 | 1 | 646 | 0 | 0 | 0 | 0 | 0 | 0 | 0 |
| cg05449164 | 5 | 19 | 162 | 2 | 1 | 1139 | 0 | 0 | 0 | 0 | 0 | 0 |
| cg05478128 | 5 | 4 | 2 | 1 | 70 | 1246 | 0 | 0 | 0 | 0 | 0 | 0 |
| cg05799088 | 4 | 42 | 1 | 312 | 968 | 0 | 0 | 0 | 0 | 0 | 0 | 0 |
| cg05966078 | 2 | 100 | 1223 | 0 | 0 | 0 | 0 | 0 | 0 | 0 | 0 | 0 |
| cg06135765 | 3 | 8 | 88 | 1227 | 0 | 0 | 0 | 0 | 0 | 0 | 0 | 0 |
| cg06300880 | 5 | 46 | 1 | 406 | 1 | 869 | 0 | 0 | 0 | 0 | 0 | 0 |
| cg06378561 | 5 | 93 | 1 | 452 | 2 | 775 | 0 | 0 | 0 | 0 | 0 | 0 |
| cg06870118 | 4 | 43 | 334 | 1 | 945 | 0 | 0 | 0 | 0 | 0 | 0 | 0 |
| cg07093060 | 2 | 112 | 1211 | 0 | 0 | 0 | 0 | 0 | 0 | 0 | 0 | 0 |
| cg07133434 | 4 | 93 | 478 | 1 | 751 | 0 | 0 | 0 | 0 | 0 | 0 | 0 |
| cg07258715 | 5 | 171 | 1 | 1 | 2 | 1148 | 0 | 0 | 0 | 0 | 0 | 0 |
| cg07576186 | 4 | 95 | 1 | 2 | 1225 | 0 | 0 | 0 | 0 | 0 | 0 | 0 |
| cg07597816 | 2 | 319 | 1004 | 0 | 0 | 0 | 0 | 0 | 0 | 0 | 0 | 0 |
| cg07650392 | 6 | 8 | 1 | 2 | 148 | 1 | 1163 | 0 | 0 | 0 | 0 | 0 |
| cg08062959 | 5 | 1255 | 1 | 61 | 2 | 4 | 0 | 0 | 0 | 0 | 0 | 0 |
| cg08146865 | 4 | 54 | 364 | 1 | 904 | 0 | 0 | 0 | 0 | 0 | 0 | 0 |
| cg08476485 | 3 | 157 | 2 | 1164 | 0 | 0 | 0 | 0 | 0 | 0 | 0 | 0 |
| cg08522473 | 3 | 239 | 582 | 502 | 0 | 0 | 0 | 0 | 0 | 0 | 0 | 0 |
| cg08821998 | 5 | 1 | 7 | 68 | 2 | 1245 | 0 | 0 | 0 | 0 | 0 | 0 |
| cg08977311 | 3 | 392 | 1 | 930 | 0 | 0 | 0 | 0 | 0 | 0 | 0 | 0 |
| cg09014801 | 2 | 177 | 1146 | 0 | 0 | 0 | 0 | 0 | 0 | 0 | 0 | 0 |
| cg09102030 | 3 | 125 | 2 | 1196 | 0 | 0 | 0 | 0 | 0 | 0 | 0 | 0 |
| cg09627057 | 5 | 40 | 1 | 328 | 3 | 951 | 0 | 0 | 0 | 0 | 0 | 0 |
| cg09639108 | 4 | 36 | 338 | 1 | 948 | 0 | 0 | 0 | 0 | 0 | 0 | 0 |
| cg09866143 | 4 | 93 | 393 | 1 | 836 | 0 | 0 | 0 | 0 | 0 | 0 | 0 |
| cg10249221 | 3 | 1105 | 174 | 44 | 0 | 0 | 0 | 0 | 0 | 0 | 0 | 0 |
| cg10336025 | 4 | 6 | 105 | 2 | 1210 | 0 | 0 | 0 | 0 | 0 | 0 | 0 |
| cg10590338 | 4 | 701 | 1 | 618 | 3 | 0 | 0 | 0 | 0 | 0 | 0 | 0 |
| cg10666341 | 2 | 86 | 1237 | 0 | 0 | 0 | 0 | 0 | 0 | 0 | 0 | 0 |
| cg10681391 | 3 | 8 | 165 | 1150 | 0 | 0 | 0 | 0 | 0 | 0 | 0 | 0 |
| cg11014960 | 3 | 78 | 1 | 1244 | 0 | 0 | 0 | 0 | 0 | 0 | 0 | 0 |
| cg11035303 | 3 | 1060 | 231 | 32 | 0 | 0 | 0 | 0 | 0 | 0 | 0 | 0 |
| cg12077433 | 3 | 63 | 406 | 854 | 0 | 0 | 0 | 0 | 0 | 0 | 0 | 0 |
| cg12381531 | 3 | 213 | 4 | 1106 | 0 | 0 | 0 | 0 | 0 | 0 | 0 | 0 |
| cg12403190 | 3 | 2 | 109 | 1212 | 0 | 0 | 0 | 0 | 0 | 0 | 0 | 0 |
| cg12414339 | 4 | 9 | 1 | 176 | 1137 | 0 | 0 | 0 | 0 | 0 | 0 | 0 |
| cg12687426 | 5 | 25 | 1 | 1 | 221 | 1075 | 0 | 0 | 0 | 0 | 0 | 0 |
| cg12918464 | 3 | 2 | 72 | 1249 | 0 | 0 | 0 | 0 | 0 | 0 | 0 | 0 |
| cg12928933 | 4 | 35 | 1 | 315 | 972 | 0 | 0 | 0 | 0 | 0 | 0 | 0 |
| cg12981577 | 2 | 108 | 1215 | 0 | 0 | 0 | 0 | 0 | 0 | 0 | 0 | 0 |
| cg13282252 | 4 | 2 | 80 | 2 | 1239 | 0 | 0 | 0 | 0 | 0 | 0 | 0 |
| cg13284789 | 4 | 43 | 1 | 325 | 954 | 0 | 0 | 0 | 0 | 0 | 0 | 0 |
| cg13332114 | 4 | 6 | 1 | 122 | 1194 | 0 | 0 | 0 | 0 | 0 | 0 | 0 |
| cg14488913 | 3 | 2 | 161 | 1160 | 0 | 0 | 0 | 0 | 0 | 0 | 0 | 0 |
| cg14711243 | 3 | 1243 | 78 | 2 | 0 | 0 | 0 | 0 | 0 | 0 | 0 | 0 |
| cg15247483 | 3 | 119 | 550 | 654 | 0 | 0 | 0 | 0 | 0 | 0 | 0 | 0 |
| cg15295200 | 3 | 185 | 3 | 1135 | 0 | 0 | 0 | 0 | 0 | 0 | 0 | 0 |
| cg15591727 | 3 | 3 | 68 | 1252 | 0 | 0 | 0 | 0 | 0 | 0 | 0 | 0 |
| cg15847988 | 4 | 3 | 1 | 92 | 1227 | 0 | 0 | 0 | 0 | 0 | 0 | 0 |
| cg15954263 | 4 | 8 | 175 | 3 | 1137 | 0 | 0 | 0 | 0 | 0 | 0 | 0 |
| cg16140565 | 5 | 157 | 2 | 1 | 432 | 731 | 0 | 0 | 0 | 0 | 0 | 0 |
| cg16193029 | 3 | 3 | 72 | 1248 | 0 | 0 | 0 | 0 | 0 | 0 | 0 | 0 |
| cg16303788 | 4 | 3 | 72 | 3 | 1245 | 0 | 0 | 0 | 0 | 0 | 0 | 0 |
| cg16364629 | 3 | 30 | 289 | 1004 | 0 | 0 | 0 | 0 | 0 | 0 | 0 | 0 |
| cg16523115 | 7 | 9 | 124 | 1 | 2 | 1 | 1183 | 3 | 0 | 0 | 0 | 0 |
| cg16570885 | 4 | 42 | 1 | 314 | 966 | 0 | 0 | 0 | 0 | 0 | 0 | 0 |
| cg16788857 | 5 | 165 | 1 | 2 | 521 | 634 | 0 | 0 | 0 | 0 | 0 | 0 |
| cg16813459 | 4 | 10 | 1 | 87 | 1225 | 0 | 0 | 0 | 0 | 0 | 0 | 0 |
| cg17336044 | 4 | 1 | 14 | 169 | 1139 | 0 | 0 | 0 | 0 | 0 | 0 | 0 |
| cg17378686 | 4 | 4 | 66 | 1 | 1252 | 0 | 0 | 0 | 0 | 0 | 0 | 0 |
| cg17388779 | 3 | 603 | 341 | 379 | 0 | 0 | 0 | 0 | 0 | 0 | 0 | 0 |
| cg17573813 | 4 | 794 | 416 | 1 | 112 | 0 | 0 | 0 | 0 | 0 | 0 | 0 |
| cg17869311 | 5 | 74 | 1 | 1 | 355 | 892 | 0 | 0 | 0 | 0 | 0 | 0 |
| cg18557837 | 7 | 1020 | 2 | 1 | 266 | 1 | 1 | 32 | 0 | 0 | 0 | 0 |
| cg18819584 | 3 | 5 | 68 | 1250 | 0 | 0 | 0 | 0 | 0 | 0 | 0 | 0 |
| cg19726630 | 4 | 1046 | 1 | 245 | 31 | 0 | 0 | 0 | 0 | 0 | 0 | 0 |
| cg19976628 | 4 | 2 | 109 | 2 | 1210 | 0 | 0 | 0 | 0 | 0 | 0 | 0 |
| cg19996396 | 4 | 25 | 303 | 1 | 994 | 0 | 0 | 0 | 0 | 0 | 0 | 0 |
| cg20131219 | 3 | 125 | 1194 | 4 | 0 | 0 | 0 | 0 | 0 | 0 | 0 | 0 |
| cg20187719 | 3 | 926 | 365 | 32 | 0 | 0 | 0 | 0 | 0 | 0 | 0 | 0 |
| cg20679403 | 3 | 2 | 114 | 1207 | 0 | 0 | 0 | 0 | 0 | 0 | 0 | 0 |
| cg20832559 | 4 | 3 | 1 | 77 | 1242 | 0 | 0 | 0 | 0 | 0 | 0 | 0 |
| cg20979384 | 4 | 192 | 1 | 1 | 1129 | 0 | 0 | 0 | 0 | 0 | 0 | 0 |
| cg21594961 | 3 | 934 | 353 | 36 | 0 | 0 | 0 | 0 | 0 | 0 | 0 | 0 |
| cg21741515 | 2 | 192 | 1131 | 0 | 0 | 0 | 0 | 0 | 0 | 0 | 0 | 0 |
| cg21909391 | 3 | 3 | 113 | 1207 | 0 | 0 | 0 | 0 | 0 | 0 | 0 | 0 |
| cg22237644 | 2 | 280 | 1043 | 0 | 0 | 0 | 0 | 0 | 0 | 0 | 0 | 0 |
| cg22710716 | 3 | 205 | 588 | 530 | 0 | 0 | 0 | 0 | 0 | 0 | 0 | 0 |
| cg22926869 | 2 | 143 | 1180 | 0 | 0 | 0 | 0 | 0 | 0 | 0 | 0 | 0 |
| cg22953510 | 2 | 177 | 1146 | 0 | 0 | 0 | 0 | 0 | 0 | 0 | 0 | 0 |
| cg22984586 | 3 | 631 | 691 | 1 | 0 | 0 | 0 | 0 | 0 | 0 | 0 | 0 |
| cg23186955 | 3 | 641 | 623 | 59 | 0 | 0 | 0 | 0 | 0 | 0 | 0 | 0 |
| cg23468456 | 4 | 12 | 210 | 1 | 1100 | 0 | 0 | 0 | 0 | 0 | 0 | 0 |
| cg23737713 | 4 | 1 | 120 | 2 | 1200 | 0 | 0 | 0 | 0 | 0 | 0 | 0 |
| cg24310293 | 5 | 9 | 2 | 1 | 157 | 1154 | 0 | 0 | 0 | 0 | 0 | 0 |
| cg24629711 | 2 | 312 | 1011 | 0 | 0 | 0 | 0 | 0 | 0 | 0 | 0 | 0 |
| cg24723129 | 3 | 6 | 75 | 1242 | 0 | 0 | 0 | 0 | 0 | 0 | 0 | 0 |
| cg25158772 | 4 | 1 | 1 | 112 | 1209 | 0 | 0 | 0 | 0 | 0 | 0 | 0 |
| cg25188166 | 3 | 82 | 153 | 1088 | 0 | 0 | 0 | 0 | 0 | 0 | 0 | 0 |
| cg25644478 | 3 | 70 | 2 | 1251 | 0 | 0 | 0 | 0 | 0 | 0 | 0 | 0 |
| cg25649515 | 4 | 159 | 1 | 522 | 641 | 0 | 0 | 0 | 0 | 0 | 0 | 0 |
| cg25658612 | 2 | 146 | 1177 | 0 | 0 | 0 | 0 | 0 | 0 | 0 | 0 | 0 |
| cg25692928 | 4 | 138 | 1 | 1183 | 1 | 0 | 0 | 0 | 0 | 0 | 0 | 0 |
| cg25951177 | 3 | 10 | 164 | 1149 | 0 | 0 | 0 | 0 | 0 | 0 | 0 | 0 |
| cg26084667 | 4 | 3 | 102 | 2 | 1216 | 0 | 0 | 0 | 0 | 0 | 0 | 0 |
| cg26331135 | 6 | 22 | 1 | 227 | 1 | 1069 | 3 | 0 | 0 | 0 | 0 | 0 |
| cg26845082 | 3 | 698 | 502 | 123 | 0 | 0 | 0 | 0 | 0 | 0 | 0 | 0 |
| cg27141807 | 6 | 253 | 1 | 1 | 1 | 467 | 600 | 0 | 0 | 0 | 0 | 0 |
| cg27187580 | 3 | 261 | 1 | 1061 | 0 | 0 | 0 | 0 | 0 | 0 | 0 | 0 |
| cg27362167 | 3 | 4 | 73 | 1246 | 0 | 0 | 0 | 0 | 0 | 0 | 0 | 0 |
| cg00082729 | 3 | 171 | 4 | 1148 | 0 | 0 | 0 | 0 | 0 | 0 | 0 | 0 |
| cg00209612 | 3 | 24 | 231 | 1068 | 0 | 0 | 0 | 0 | 0 | 0 | 0 | 0 |
| cg00248861 | 4 | 1 | 111 | 1 | 1210 | 0 | 0 | 0 | 0 | 0 | 0 | 0 |
| cg00318166 | 5 | 41 | 2 | 352 | 927 | 1 | 0 | 0 | 0 | 0 | 0 | 0 |
| cg00320354 | 5 | 38 | 2 | 4 | 310 | 969 | 0 | 0 | 0 | 0 | 0 | 0 |
| cg00323861 | 3 | 60 | 9 | 1254 | 0 | 0 | 0 | 0 | 0 | 0 | 0 | 0 |
| cg00532122 | 5 | 170 | 3 | 2 | 1 | 1147 | 0 | 0 | 0 | 0 | 0 | 0 |
| cg00675157 | 6 | 74 | 1 | 1 | 1 | 398 | 848 | 0 | 0 | 0 | 0 | 0 |
| cg01406776 | 4 | 15 | 193 | 2 | 1113 | 0 | 0 | 0 | 0 | 0 | 0 | 0 |
| cg01818594 | 3 | 1251 | 70 | 2 | 0 | 0 | 0 | 0 | 0 | 0 | 0 | 0 |
| cg02096220 | 5 | 23 | 3 | 250 | 1 | 1046 | 0 | 0 | 0 | 0 | 0 | 0 |
| cg02099267 | 3 | 8 | 154 | 1161 | 0 | 0 | 0 | 0 | 0 | 0 | 0 | 0 |
| cg02722613 | 3 | 1086 | 1 | 236 | 0 | 0 | 0 | 0 | 0 | 0 | 0 | 0 |
| cg02877261 | 3 | 392 | 1 | 930 | 0 | 0 | 0 | 0 | 0 | 0 | 0 | 0 |
| cg02951719 | 3 | 15 | 180 | 1128 | 0 | 0 | 0 | 0 | 0 | 0 | 0 | 0 |
| cg03088219 | 3 | 439 | 2 | 882 | 0 | 0 | 0 | 0 | 0 | 0 | 0 | 0 |
| cg03301671 | 4 | 3 | 64 | 3 | 1253 | 0 | 0 | 0 | 0 | 0 | 0 | 0 |
| cg03639185 | 4 | 305 | 1 | 547 | 470 | 0 | 0 | 0 | 0 | 0 | 0 | 0 |
| cg03640465 | 3 | 2 | 259 | 1062 | 0 | 0 | 0 | 0 | 0 | 0 | 0 | 0 |
| cg03810282 | 4 | 66 | 1 | 1 | 1255 | 0 | 0 | 0 | 0 | 0 | 0 | 0 |
| cg03965172 | 3 | 140 | 481 | 702 | 0 | 0 | 0 | 0 | 0 | 0 | 0 | 0 |
| cg03988092 | 2 | 711 | 612 | 0 | 0 | 0 | 0 | 0 | 0 | 0 | 0 | 0 |
| cg04012354 | 3 | 94 | 1 | 1228 | 0 | 0 | 0 | 0 | 0 | 0 | 0 | 0 |
| cg04073914 | 4 | 111 | 567 | 644 | 1 | 0 | 0 | 0 | 0 | 0 | 0 | 0 |
| cg04118610 | 3 | 329 | 989 | 5 | 0 | 0 | 0 | 0 | 0 | 0 | 0 | 0 |
| cg04331561 | 3 | 64 | 298 | 961 | 0 | 0 | 0 | 0 | 0 | 0 | 0 | 0 |
| cg05093818 | 5 | 104 | 2 | 2 | 1 | 1214 | 0 | 0 | 0 | 0 | 0 | 0 |
| cg05366050 | 3 | 123 | 5 | 1195 | 0 | 0 | 0 | 0 | 0 | 0 | 0 | 0 |
| cg05526809 | 5 | 57 | 2 | 311 | 1 | 952 | 0 | 0 | 0 | 0 | 0 | 0 |
| cg05791544 | 4 | 8 | 2 | 156 | 1157 | 0 | 0 | 0 | 0 | 0 | 0 | 0 |
| cg05893845 | 6 | 21 | 1 | 1 | 225 | 3 | 1072 | 0 | 0 | 0 | 0 | 0 |
| cg06202802 | 3 | 28 | 237 | 1058 | 0 | 0 | 0 | 0 | 0 | 0 | 0 | 0 |
| cg06318935 | 4 | 183 | 1 | 486 | 653 | 0 | 0 | 0 | 0 | 0 | 0 | 0 |
| cg06758191 | 4 | 68 | 1 | 1 | 1253 | 0 | 0 | 0 | 0 | 0 | 0 | 0 |
| cg06855422 | 3 | 153 | 1168 | 2 | 0 | 0 | 0 | 0 | 0 | 0 | 0 | 0 |
| cg07189582 | 4 | 35 | 2 | 359 | 927 | 0 | 0 | 0 | 0 | 0 | 0 | 0 |
| cg07909498 | 3 | 359 | 4 | 960 | 0 | 0 | 0 | 0 | 0 | 0 | 0 | 0 |
| cg07951602 | 3 | 130 | 1188 | 5 | 0 | 0 | 0 | 0 | 0 | 0 | 0 | 0 |
| cg07973162 | 3 | 266 | 10 | 1047 | 0 | 0 | 0 | 0 | 0 | 0 | 0 | 0 |
| cg08061367 | 3 | 72 | 6 | 1245 | 0 | 0 | 0 | 0 | 0 | 0 | 0 | 0 |
| cg08365591 | 4 | 33 | 217 | 3 | 1070 | 0 | 0 | 0 | 0 | 0 | 0 | 0 |
| cg08395784 | 3 | 33 | 332 | 958 | 0 | 0 | 0 | 0 | 0 | 0 | 0 | 0 |
| cg08669168 | 3 | 99 | 477 | 747 | 0 | 0 | 0 | 0 | 0 | 0 | 0 | 0 |
| cg08684066 | 4 | 32 | 266 | 1 | 1024 | 0 | 0 | 0 | 0 | 0 | 0 | 0 |
| cg08891829 | 4 | 47 | 1 | 363 | 912 | 0 | 0 | 0 | 0 | 0 | 0 | 0 |
| cg08916385 | 3 | 143 | 1 | 1179 | 0 | 0 | 0 | 0 | 0 | 0 | 0 | 0 |
| cg09120722 | 3 | 137 | 2 | 1184 | 0 | 0 | 0 | 0 | 0 | 0 | 0 | 0 |
| cg09438069 | 3 | 125 | 493 | 705 | 0 | 0 | 0 | 0 | 0 | 0 | 0 | 0 |
| cg10563418 | 4 | 8 | 101 | 1213 | 1 | 0 | 0 | 0 | 0 | 0 | 0 | 0 |
| cg10590622 | 4 | 745 | 472 | 104 | 2 | 0 | 0 | 0 | 0 | 0 | 0 | 0 |
| cg10712884 | 4 | 29 | 1 | 265 | 1028 | 0 | 0 | 0 | 0 | 0 | 0 | 0 |
| cg10852718 | 3 | 41 | 271 | 1011 | 0 | 0 | 0 | 0 | 0 | 0 | 0 | 0 |
| cg11106864 | 4 | 5 | 1 | 120 | 1197 | 0 | 0 | 0 | 0 | 0 | 0 | 0 |
| cg11202023 | 4 | 11 | 112 | 1195 | 5 | 0 | 0 | 0 | 0 | 0 | 0 | 0 |
| cg11379315 | 2 | 93 | 1230 | 0 | 0 | 0 | 0 | 0 | 0 | 0 | 0 | 0 |
| cg11663691 | 3 | 397 | 1 | 925 | 0 | 0 | 0 | 0 | 0 | 0 | 0 | 0 |
| cg11674404 | 3 | 1212 | 105 | 6 | 0 | 0 | 0 | 0 | 0 | 0 | 0 | 0 |
| cg11791052 | 5 | 5 | 1 | 1 | 78 | 1238 | 0 | 0 | 0 | 0 | 0 | 0 |
| cg11956442 | 4 | 523 | 6 | 607 | 187 | 0 | 0 | 0 | 0 | 0 | 0 | 0 |
| cg12012426 | 2 | 791 | 532 | 0 | 0 | 0 | 0 | 0 | 0 | 0 | 0 | 0 |
| cg12214399 | 5 | 363 | 1 | 1 | 954 | 4 | 0 | 0 | 0 | 0 | 0 | 0 |
| cg12279734 | 3 | 95 | 1 | 1227 | 0 | 0 | 0 | 0 | 0 | 0 | 0 | 0 |
| cg12454925 | 5 | 5 | 1 | 1 | 109 | 1207 | 0 | 0 | 0 | 0 | 0 | 0 |
| cg12479705 | 2 | 446 | 877 | 0 | 0 | 0 | 0 | 0 | 0 | 0 | 0 | 0 |
| cg12579198 | 3 | 12 | 153 | 1158 | 0 | 0 | 0 | 0 | 0 | 0 | 0 | 0 |
| cg13248811 | 3 | 324 | 8 | 991 | 0 | 0 | 0 | 0 | 0 | 0 | 0 | 0 |
| cg14241748 | 3 | 107 | 1 | 1215 | 0 | 0 | 0 | 0 | 0 | 0 | 0 | 0 |
| cg14422932 | 3 | 157 | 4 | 1162 | 0 | 0 | 0 | 0 | 0 | 0 | 0 | 0 |
| cg14497649 | 4 | 51 | 387 | 1 | 884 | 0 | 0 | 0 | 0 | 0 | 0 | 0 |
| cg14724492 | 3 | 1 | 76 | 1246 | 0 | 0 | 0 | 0 | 0 | 0 | 0 | 0 |
| cg14887054 | 4 | 7 | 1 | 173 | 1142 | 0 | 0 | 0 | 0 | 0 | 0 | 0 |
| cg15248972 | 4 | 204 | 1 | 476 | 642 | 0 | 0 | 0 | 0 | 0 | 0 | 0 |
| cg15584084 | 5 | 9 | 1 | 2 | 97 | 1214 | 0 | 0 | 0 | 0 | 0 | 0 |
| cg15877769 | 6 | 66 | 1 | 1 | 1 | 304 | 950 | 0 | 0 | 0 | 0 | 0 |
| cg15897435 | 7 | 741 | 1 | 1 | 1 | 1 | 577 | 1 | 0 | 0 | 0 | 0 |
| cg15964593 | 4 | 126 | 4 | 1191 | 2 | 0 | 0 | 0 | 0 | 0 | 0 | 0 |
| cg15970640 | 3 | 17 | 234 | 1072 | 0 | 0 | 0 | 0 | 0 | 0 | 0 | 0 |
| cg16301894 | 2 | 70 | 1253 | 0 | 0 | 0 | 0 | 0 | 0 | 0 | 0 | 0 |
| cg16373817 | 5 | 32 | 1 | 1 | 362 | 927 | 0 | 0 | 0 | 0 | 0 | 0 |
| cg16854281 | 2 | 77 | 1246 | 0 | 0 | 0 | 0 | 0 | 0 | 0 | 0 | 0 |
| cg16871385 | 4 | 8 | 1 | 142 | 1172 | 0 | 0 | 0 | 0 | 0 | 0 | 0 |
| cg17004290 | 4 | 439 | 624 | 2 | 258 | 0 | 0 | 0 | 0 | 0 | 0 | 0 |
| cg17155524 | 4 | 444 | 3 | 3 | 873 | 0 | 0 | 0 | 0 | 0 | 0 | 0 |
| cg17858192 | 3 | 793 | 454 | 76 | 0 | 0 | 0 | 0 | 0 | 0 | 0 | 0 |
| cg18075755 | 5 | 376 | 1 | 1 | 541 | 404 | 0 | 0 | 0 | 0 | 0 | 0 |
| cg18093448 | 4 | 39 | 4 | 304 | 976 | 0 | 0 | 0 | 0 | 0 | 0 | 0 |
| cg18162908 | 4 | 8 | 81 | 1 | 1233 | 0 | 0 | 0 | 0 | 0 | 0 | 0 |
| cg18434356 | 3 | 13 | 207 | 1103 | 0 | 0 | 0 | 0 | 0 | 0 | 0 | 0 |
| cg19311470 | 4 | 701 | 489 | 4 | 129 | 0 | 0 | 0 | 0 | 0 | 0 | 0 |
| cg19484093 | 3 | 207 | 1 | 1115 | 0 | 0 | 0 | 0 | 0 | 0 | 0 | 0 |
| cg19935756 | 5 | 386 | 3 | 517 | 414 | 3 | 0 | 0 | 0 | 0 | 0 | 0 |
| cg19952704 | 4 | 1135 | 1 | 173 | 14 | 0 | 0 | 0 | 0 | 0 | 0 | 0 |
| cg20027946 | 3 | 78 | 1 | 1244 | 0 | 0 | 0 | 0 | 0 | 0 | 0 | 0 |
| cg20033591 | 2 | 141 | 1182 | 0 | 0 | 0 | 0 | 0 | 0 | 0 | 0 | 0 |
| cg20078646 | 2 | 254 | 1069 | 0 | 0 | 0 | 0 | 0 | 0 | 0 | 0 | 0 |
| cg20213329 | 2 | 986 | 337 | 0 | 0 | 0 | 0 | 0 | 0 | 0 | 0 | 0 |
| cg20992733 | 4 | 6 | 1 | 181 | 1135 | 0 | 0 | 0 | 0 | 0 | 0 | 0 |
| cg21005683 | 8 | 17 | 1 | 2 | 1 | 178 | 2 | 1121 | 1 | 0 | 0 | 0 |
| cg21524061 | 6 | 42 | 2 | 1 | 1 | 341 | 936 | 0 | 0 | 0 | 0 | 0 |
| cg21544585 | 3 | 12 | 211 | 1100 | 0 | 0 | 0 | 0 | 0 | 0 | 0 | 0 |
| cg21645759 | 3 | 23 | 256 | 1044 | 0 | 0 | 0 | 0 | 0 | 0 | 0 | 0 |
| cg21743826 | 4 | 9 | 1 | 100 | 1213 | 0 | 0 | 0 | 0 | 0 | 0 | 0 |
| cg21795255 | 5 | 312 | 1 | 575 | 2 | 433 | 0 | 0 | 0 | 0 | 0 | 0 |
| cg22007216 | 4 | 102 | 1 | 454 | 766 | 0 | 0 | 0 | 0 | 0 | 0 | 0 |
| cg22436195 | 3 | 107 | 1215 | 1 | 0 | 0 | 0 | 0 | 0 | 0 | 0 | 0 |
| cg23048051 | 3 | 20 | 175 | 1128 | 0 | 0 | 0 | 0 | 0 | 0 | 0 | 0 |
| cg23057687 | 5 | 17 | 1 | 1 | 141 | 1163 | 0 | 0 | 0 | 0 | 0 | 0 |
| cg23071009 | 3 | 12 | 118 | 1193 | 0 | 0 | 0 | 0 | 0 | 0 | 0 | 0 |
| cg24135923 | 4 | 13 | 128 | 2 | 1180 | 0 | 0 | 0 | 0 | 0 | 0 | 0 |
| cg25569462 | 2 | 190 | 1133 | 0 | 0 | 0 | 0 | 0 | 0 | 0 | 0 | 0 |
| cg25795606 | 3 | 7 | 80 | 1236 | 0 | 0 | 0 | 0 | 0 | 0 | 0 | 0 |
| cg26128129 | 5 | 22 | 1 | 243 | 2 | 1055 | 0 | 0 | 0 | 0 | 0 | 0 |
| cg26184765 | 4 | 29 | 1 | 339 | 954 | 0 | 0 | 0 | 0 | 0 | 0 | 0 |
| cg26278987 | 4 | 77 | 1 | 393 | 852 | 0 | 0 | 0 | 0 | 0 | 0 | 0 |
| cg26398228 | 4 | 98 | 1222 | 1 | 2 | 0 | 0 | 0 | 0 | 0 | 0 | 0 |
| cg26454724 | 4 | 2 | 1 | 65 | 1255 | 0 | 0 | 0 | 0 | 0 | 0 | 0 |
| cg26867200 | 4 | 7 | 1 | 104 | 1211 | 0 | 0 | 0 | 0 | 0 | 0 | 0 |
| cg26953326 | 3 | 67 | 1 | 1255 | 0 | 0 | 0 | 0 | 0 | 0 | 0 | 0 |
| cg27049827 | 3 | 75 | 1247 | 1 | 0 | 0 | 0 | 0 | 0 | 0 | 0 | 0 |
| cg27050912 | 6 | 15 | 6 | 2 | 2 | 126 | 1172 | 0 | 0 | 0 | 0 | 0 |
| cg27160931 | 2 | 206 | 1117 | 0 | 0 | 0 | 0 | 0 | 0 | 0 | 0 | 0 |
| cg27341708 | 3 | 425 | 523 | 375 | 0 | 0 | 0 | 0 | 0 | 0 | 0 | 0 |
| cg27581660 | 3 | 17 | 284 | 1022 | 0 | 0 | 0 | 0 | 0 | 0 | 0 | 0 |
| cg00546757 | 5 | 236 | 1 | 5 | 617 | 464 | 0 | 0 | 0 | 0 | 0 | 0 |
| cg00631759 | 3 | 83 | 74 | 1166 | 0 | 0 | 0 | 0 | 0 | 0 | 0 | 0 |
| cg00756172 | 3 | 2 | 68 | 1253 | 0 | 0 | 0 | 0 | 0 | 0 | 0 | 0 |
| cg00811771 | 3 | 1193 | 119 | 11 | 0 | 0 | 0 | 0 | 0 | 0 | 0 | 0 |
| cg00968488 | 2 | 315 | 1008 | 0 | 0 | 0 | 0 | 0 | 0 | 0 | 0 | 0 |
| cg01208126 | 2 | 67 | 1256 | 0 | 0 | 0 | 0 | 0 | 0 | 0 | 0 | 0 |
| cg01551388 | 4 | 28 | 170 | 1 | 1124 | 0 | 0 | 0 | 0 | 0 | 0 | 0 |
| cg01808284 | 3 | 82 | 1 | 1240 | 0 | 0 | 0 | 0 | 0 | 0 | 0 | 0 |
| cg02288345 | 4 | 76 | 381 | 2 | 864 | 0 | 0 | 0 | 0 | 0 | 0 | 0 |
| cg02351082 | 3 | 1203 | 1 | 119 | 0 | 0 | 0 | 0 | 0 | 0 | 0 | 0 |
| cg02352685 | 4 | 73 | 2 | 1 | 1247 | 0 | 0 | 0 | 0 | 0 | 0 | 0 |
| cg02550738 | 6 | 72 | 1 | 1 | 1 | 375 | 873 | 0 | 0 | 0 | 0 | 0 |
| cg02593734 | 4 | 4 | 1 | 99 | 1219 | 0 | 0 | 0 | 0 | 0 | 0 | 0 |
| cg02614045 | 4 | 27 | 1 | 265 | 1030 | 0 | 0 | 0 | 0 | 0 | 0 | 0 |
| cg02707593 | 4 | 12 | 2 | 171 | 1138 | 0 | 0 | 0 | 0 | 0 | 0 | 0 |
| cg02772171 | 4 | 186 | 2 | 499 | 636 | 0 | 0 | 0 | 0 | 0 | 0 | 0 |
| cg03979311 | 2 | 192 | 1131 | 0 | 0 | 0 | 0 | 0 | 0 | 0 | 0 | 0 |
| cg04497820 | 3 | 467 | 5 | 851 | 0 | 0 | 0 | 0 | 0 | 0 | 0 | 0 |
| cg04829448 | 3 | 2 | 280 | 1041 | 0 | 0 | 0 | 0 | 0 | 0 | 0 | 0 |
| cg05007442 | 3 | 1224 | 94 | 5 | 0 | 0 | 0 | 0 | 0 | 0 | 0 | 0 |
| cg05482050 | 2 | 1132 | 191 | 0 | 0 | 0 | 0 | 0 | 0 | 0 | 0 | 0 |
| cg06636485 | 3 | 9 | 177 | 1137 | 0 | 0 | 0 | 0 | 0 | 0 | 0 | 0 |
| cg06653140 | 2 | 542 | 781 | 0 | 0 | 0 | 0 | 0 | 0 | 0 | 0 | 0 |
| cg06849895 | 5 | 10 | 1 | 95 | 2 | 1215 | 0 | 0 | 0 | 0 | 0 | 0 |
| cg07124183 | 4 | 51 | 257 | 1 | 1014 | 0 | 0 | 0 | 0 | 0 | 0 | 0 |
| cg07664579 | 5 | 50 | 1 | 398 | 1 | 873 | 0 | 0 | 0 | 0 | 0 | 0 |
| cg07807373 | 3 | 242 | 1 | 1080 | 0 | 0 | 0 | 0 | 0 | 0 | 0 | 0 |
| cg08238319 | 5 | 171 | 1 | 1 | 525 | 625 | 0 | 0 | 0 | 0 | 0 | 0 |
| cg08238375 | 6 | 305 | 6 | 2 | 527 | 4 | 479 | 0 | 0 | 0 | 0 | 0 |
| cg08506672 | 4 | 594 | 1 | 561 | 167 | 0 | 0 | 0 | 0 | 0 | 0 | 0 |
| cg08558478 | 3 | 4 | 69 | 1250 | 0 | 0 | 0 | 0 | 0 | 0 | 0 | 0 |
| cg09101062 | 3 | 77 | 1245 | 1 | 0 | 0 | 0 | 0 | 0 | 0 | 0 | 0 |
| cg09434603 | 3 | 121 | 1201 | 1 | 0 | 0 | 0 | 0 | 0 | 0 | 0 | 0 |
| cg09483595 | 4 | 9 | 2 | 121 | 1191 | 0 | 0 | 0 | 0 | 0 | 0 | 0 |
| cg09672255 | 5 | 136 | 1 | 2 | 487 | 697 | 0 | 0 | 0 | 0 | 0 | 0 |
| cg09730955 | 5 | 7 | 1 | 107 | 2 | 1206 | 0 | 0 | 0 | 0 | 0 | 0 |
| cg10140678 | 4 | 257 | 4 | 1060 | 2 | 0 | 0 | 0 | 0 | 0 | 0 | 0 |
| cg10608636 | 5 | 7 | 1 | 2 | 81 | 1232 | 0 | 0 | 0 | 0 | 0 | 0 |
| cg10926851 | 5 | 76 | 1 | 437 | 2 | 807 | 0 | 0 | 0 | 0 | 0 | 0 |
| cg10934668 | 5 | 49 | 1 | 4 | 216 | 1053 | 0 | 0 | 0 | 0 | 0 | 0 |
| cg10942914 | 2 | 216 | 1107 | 0 | 0 | 0 | 0 | 0 | 0 | 0 | 0 | 0 |
| cg10946263 | 5 | 3 | 1 | 1 | 73 | 1245 | 0 | 0 | 0 | 0 | 0 | 0 |
| cg11019305 | 2 | 170 | 1153 | 0 | 0 | 0 | 0 | 0 | 0 | 0 | 0 | 0 |
| cg11128983 | 3 | 448 | 5 | 870 | 0 | 0 | 0 | 0 | 0 | 0 | 0 | 0 |
| cg11162116 | 3 | 19 | 161 | 1143 | 0 | 0 | 0 | 0 | 0 | 0 | 0 | 0 |
| cg11268585 | 4 | 142 | 4 | 1 | 1176 | 0 | 0 | 0 | 0 | 0 | 0 | 0 |
| cg11330282 | 5 | 14 | 110 | 1 | 1197 | 1 | 0 | 0 | 0 | 0 | 0 | 0 |
| cg11422312 | 4 | 32 | 2 | 219 | 1070 | 0 | 0 | 0 | 0 | 0 | 0 | 0 |
| cg11529236 | 3 | 23 | 174 | 1126 | 0 | 0 | 0 | 0 | 0 | 0 | 0 | 0 |
| cg11547201 | 4 | 7 | 116 | 1 | 1199 | 0 | 0 | 0 | 0 | 0 | 0 | 0 |
| cg11585022 | 7 | 380 | 1 | 1 | 1 | 626 | 310 | 4 | 0 | 0 | 0 | 0 |
| cg11685249 | 4 | 9 | 160 | 1 | 1153 | 0 | 0 | 0 | 0 | 0 | 0 | 0 |
| cg11786587 | 4 | 126 | 1 | 566 | 630 | 0 | 0 | 0 | 0 | 0 | 0 | 0 |
| cg11791078 | 3 | 143 | 6 | 1174 | 0 | 0 | 0 | 0 | 0 | 0 | 0 | 0 |
| cg12471283 | 3 | 95 | 1 | 1227 | 0 | 0 | 0 | 0 | 0 | 0 | 0 | 0 |
| cg12515659 | 3 | 388 | 1 | 934 | 0 | 0 | 0 | 0 | 0 | 0 | 0 | 0 |
| cg12599971 | 4 | 127 | 1189 | 4 | 3 | 0 | 0 | 0 | 0 | 0 | 0 | 0 |
| cg12869097 | 5 | 10 | 1 | 143 | 1 | 1168 | 0 | 0 | 0 | 0 | 0 | 0 |
| cg12908908 | 3 | 214 | 1 | 1108 | 0 | 0 | 0 | 0 | 0 | 0 | 0 | 0 |
| cg12974258 | 4 | 17 | 176 | 1129 | 1 | 0 | 0 | 0 | 0 | 0 | 0 | 0 |
| cg13284426 | 4 | 5 | 1 | 101 | 1216 | 0 | 0 | 0 | 0 | 0 | 0 | 0 |
| cg13386926 | 5 | 4 | 72 | 2 | 1 | 1244 | 0 | 0 | 0 | 0 | 0 | 0 |
| cg13591052 | 3 | 165 | 1154 | 4 | 0 | 0 | 0 | 0 | 0 | 0 | 0 | 0 |
| cg13612055 | 3 | 73 | 1 | 1249 | 0 | 0 | 0 | 0 | 0 | 0 | 0 | 0 |
| cg13653328 | 4 | 154 | 1 | 470 | 698 | 0 | 0 | 0 | 0 | 0 | 0 | 0 |
| cg13905298 | 4 | 130 | 1 | 503 | 689 | 0 | 0 | 0 | 0 | 0 | 0 | 0 |
| cg13972557 | 2 | 706 | 617 | 0 | 0 | 0 | 0 | 0 | 0 | 0 | 0 | 0 |
| cg14388237 | 3 | 1075 | 210 | 38 | 0 | 0 | 0 | 0 | 0 | 0 | 0 | 0 |
| cg14797147 | 5 | 199 | 1 | 1 | 576 | 546 | 0 | 0 | 0 | 0 | 0 | 0 |
| cg14872828 | 4 | 1 | 59 | 35 | 1228 | 0 | 0 | 0 | 0 | 0 | 0 | 0 |
| cg14983172 | 3 | 260 | 1062 | 1 | 0 | 0 | 0 | 0 | 0 | 0 | 0 | 0 |
| cg15016740 | 5 | 1 | 1 | 72 | 1 | 1248 | 0 | 0 | 0 | 0 | 0 | 0 |
| cg15180869 | 5 | 1 | 1 | 94 | 1 | 1226 | 0 | 0 | 0 | 0 | 0 | 0 |
| cg15402732 | 4 | 34 | 206 | 1 | 1082 | 0 | 0 | 0 | 0 | 0 | 0 | 0 |
| cg15421137 | 5 | 586 | 1 | 554 | 181 | 1 | 0 | 0 | 0 | 0 | 0 | 0 |
| cg15668967 | 2 | 133 | 1190 | 0 | 0 | 0 | 0 | 0 | 0 | 0 | 0 | 0 |
| cg15725238 | 3 | 8 | 93 | 1222 | 0 | 0 | 0 | 0 | 0 | 0 | 0 | 0 |
| cg15845365 | 3 | 668 | 2 | 653 | 0 | 0 | 0 | 0 | 0 | 0 | 0 | 0 |
| cg15909443 | 4 | 163 | 567 | 1 | 592 | 0 | 0 | 0 | 0 | 0 | 0 | 0 |
| cg16079430 | 3 | 102 | 1 | 1220 | 0 | 0 | 0 | 0 | 0 | 0 | 0 | 0 |
| cg16081854 | 3 | 174 | 474 | 675 | 0 | 0 | 0 | 0 | 0 | 0 | 0 | 0 |
| cg16167565 | 4 | 232 | 2 | 2 | 1087 | 0 | 0 | 0 | 0 | 0 | 0 | 0 |
| cg16534315 | 5 | 18 | 1 | 1 | 147 | 1156 | 0 | 0 | 0 | 0 | 0 | 0 |
| cg17221856 | 2 | 80 | 1243 | 0 | 0 | 0 | 0 | 0 | 0 | 0 | 0 | 0 |
| cg17386240 | 3 | 144 | 1178 | 1 | 0 | 0 | 0 | 0 | 0 | 0 | 0 | 0 |
| cg17534070 | 3 | 157 | 2 | 1164 | 0 | 0 | 0 | 0 | 0 | 0 | 0 | 0 |
| cg17670013 | 4 | 122 | 1 | 3 | 1197 | 0 | 0 | 0 | 0 | 0 | 0 | 0 |
| cg18104870 | 4 | 13 | 103 | 2 | 1205 | 0 | 0 | 0 | 0 | 0 | 0 | 0 |
| cg18394648 | 5 | 5 | 1 | 116 | 4 | 1197 | 0 | 0 | 0 | 0 | 0 | 0 |
| cg18409782 | 2 | 306 | 1017 | 0 | 0 | 0 | 0 | 0 | 0 | 0 | 0 | 0 |
| cg18790340 | 4 | 4 | 75 | 1 | 1243 | 0 | 0 | 0 | 0 | 0 | 0 | 0 |
| cg18797872 | 4 | 114 | 1 | 299 | 909 | 0 | 0 | 0 | 0 | 0 | 0 | 0 |
| cg18849725 | 3 | 13 | 161 | 1149 | 0 | 0 | 0 | 0 | 0 | 0 | 0 | 0 |
| cg18899797 | 3 | 2 | 79 | 1242 | 0 | 0 | 0 | 0 | 0 | 0 | 0 | 0 |
| cg19569170 | 4 | 13 | 1 | 123 | 1186 | 0 | 0 | 0 | 0 | 0 | 0 | 0 |
| cg19729949 | 5 | 2 | 71 | 1 | 1 | 1248 | 0 | 0 | 0 | 0 | 0 | 0 |
| cg19923485 | 4 | 3 | 73 | 1 | 1246 | 0 | 0 | 0 | 0 | 0 | 0 | 0 |
| cg20462978 | 6 | 9 | 1 | 2 | 155 | 1 | 1155 | 0 | 0 | 0 | 0 | 0 |
| cg20687255 | 3 | 9 | 117 | 1197 | 0 | 0 | 0 | 0 | 0 | 0 | 0 | 0 |
| cg20973649 | 3 | 10 | 131 | 1182 | 0 | 0 | 0 | 0 | 0 | 0 | 0 | 0 |
| cg21032945 | 3 | 6 | 104 | 1213 | 0 | 0 | 0 | 0 | 0 | 0 | 0 | 0 |
| cg21036194 | 2 | 104 | 1219 | 0 | 0 | 0 | 0 | 0 | 0 | 0 | 0 | 0 |
| cg21070081 | 5 | 449 | 1 | 2 | 426 | 445 | 0 | 0 | 0 | 0 | 0 | 0 |
| cg21226754 | 5 | 5 | 1 | 1 | 150 | 1166 | 0 | 0 | 0 | 0 | 0 | 0 |
| cg21543103 | 2 | 180 | 1143 | 0 | 0 | 0 | 0 | 0 | 0 | 0 | 0 | 0 |
| cg21610927 | 3 | 130 | 2 | 1191 | 0 | 0 | 0 | 0 | 0 | 0 | 0 | 0 |
| cg21743925 | 5 | 9 | 1 | 2 | 141 | 1170 | 0 | 0 | 0 | 0 | 0 | 0 |
| cg21874902 | 3 | 16 | 214 | 1093 | 0 | 0 | 0 | 0 | 0 | 0 | 0 | 0 |
| cg22306009 | 3 | 1059 | 233 | 31 | 0 | 0 | 0 | 0 | 0 | 0 | 0 | 0 |
| cg22561794 | 3 | 72 | 277 | 974 | 0 | 0 | 0 | 0 | 0 | 0 | 0 | 0 |
| cg22660197 | 5 | 15 | 1 | 146 | 1159 | 2 | 0 | 0 | 0 | 0 | 0 | 0 |
| cg22777560 | 5 | 386 | 1 | 5 | 1 | 930 | 0 | 0 | 0 | 0 | 0 | 0 |
| cg22851875 | 3 | 536 | 544 | 243 | 0 | 0 | 0 | 0 | 0 | 0 | 0 | 0 |
| cg22876446 | 5 | 8 | 1 | 94 | 1 | 1219 | 0 | 0 | 0 | 0 | 0 | 0 |
| cg22917366 | 4 | 98 | 1 | 2 | 1222 | 0 | 0 | 0 | 0 | 0 | 0 | 0 |
| cg22931151 | 5 | 90 | 1 | 1 | 444 | 787 | 0 | 0 | 0 | 0 | 0 | 0 |
| cg22946888 | 4 | 179 | 1 | 1 | 1142 | 0 | 0 | 0 | 0 | 0 | 0 | 0 |
| cg23019589 | 3 | 182 | 1139 | 2 | 0 | 0 | 0 | 0 | 0 | 0 | 0 | 0 |
| cg23027179 | 4 | 2 | 70 | 2 | 1249 | 0 | 0 | 0 | 0 | 0 | 0 | 0 |
| cg23517115 | 3 | 262 | 2 | 1059 | 0 | 0 | 0 | 0 | 0 | 0 | 0 | 0 |
| cg23943944 | 4 | 158 | 4 | 1159 | 2 | 0 | 0 | 0 | 0 | 0 | 0 | 0 |
| cg24009806 | 4 | 95 | 495 | 4 | 729 | 0 | 0 | 0 | 0 | 0 | 0 | 0 |
| cg24137123 | 5 | 26 | 2 | 264 | 1 | 1030 | 0 | 0 | 0 | 0 | 0 | 0 |
| cg24676664 | 4 | 17 | 1 | 174 | 1131 | 0 | 0 | 0 | 0 | 0 | 0 | 0 |
| cg24805360 | 3 | 2 | 88 | 1233 | 0 | 0 | 0 | 0 | 0 | 0 | 0 | 0 |
| cg24844518 | 5 | 355 | 1 | 545 | 1 | 421 | 0 | 0 | 0 | 0 | 0 | 0 |
| cg25399998 | 3 | 17 | 133 | 1173 | 0 | 0 | 0 | 0 | 0 | 0 | 0 | 0 |
| cg25673075 | 4 | 114 | 2 | 508 | 699 | 0 | 0 | 0 | 0 | 0 | 0 | 0 |
| cg25997988 | 5 | 61 | 1 | 376 | 3 | 882 | 0 | 0 | 0 | 0 | 0 | 0 |
| cg26201434 | 3 | 118 | 3 | 1202 | 0 | 0 | 0 | 0 | 0 | 0 | 0 | 0 |
| cg26320890 | 3 | 3 | 75 | 1245 | 0 | 0 | 0 | 0 | 0 | 0 | 0 | 0 |
| cg26440059 | 2 | 82 | 1241 | 0 | 0 | 0 | 0 | 0 | 0 | 0 | 0 | 0 |
| cg27032760 | 3 | 5 | 62 | 1256 | 0 | 0 | 0 | 0 | 0 | 0 | 0 | 0 |
| cg27126508 | 4 | 2 | 1 | 128 | 1192 | 0 | 0 | 0 | 0 | 0 | 0 | 0 |
| cg27141889 | 4 | 12 | 2 | 144 | 1165 | 0 | 0 | 0 | 0 | 0 | 0 | 0 |
| cg27586797 | 4 | 463 | 1 | 621 | 238 | 0 | 0 | 0 | 0 | 0 | 0 | 0 |
| cg27588076 | 3 | 8 | 127 | 1188 | 0 | 0 | 0 | 0 | 0 | 0 | 0 | 0 |
| cg00035449 | 3 | 297 | 603 | 423 | 0 | 0 | 0 | 0 | 0 | 0 | 0 | 0 |
| cg00047553 | 2 | 139 | 1184 | 0 | 0 | 0 | 0 | 0 | 0 | 0 | 0 | 0 |
| cg00122779 | 3 | 85 | 1235 | 3 | 0 | 0 | 0 | 0 | 0 | 0 | 0 | 0 |
| cg00156497 | 3 | 65 | 1256 | 2 | 0 | 0 | 0 | 0 | 0 | 0 | 0 | 0 |
| cg00188089 | 3 | 4 | 111 | 1208 | 0 | 0 | 0 | 0 | 0 | 0 | 0 | 0 |
| cg00326788 | 3 | 2 | 129 | 1192 | 0 | 0 | 0 | 0 | 0 | 0 | 0 | 0 |
| cg00328916 | 3 | 5 | 85 | 1233 | 0 | 0 | 0 | 0 | 0 | 0 | 0 | 0 |
| cg00332305 | 5 | 16 | 237 | 2 | 1067 | 1 | 0 | 0 | 0 | 0 | 0 | 0 |
| cg00366603 | 5 | 169 | 2 | 1 | 597 | 554 | 0 | 0 | 0 | 0 | 0 | 0 |
| cg00372886 | 3 | 1 | 77 | 1245 | 0 | 0 | 0 | 0 | 0 | 0 | 0 | 0 |
| cg00689685 | 4 | 65 | 1 | 1 | 1256 | 0 | 0 | 0 | 0 | 0 | 0 | 0 |
| cg01192112 | 4 | 6 | 1 | 86 | 1230 | 0 | 0 | 0 | 0 | 0 | 0 | 0 |
| cg01203766 | 3 | 96 | 1 | 1226 | 0 | 0 | 0 | 0 | 0 | 0 | 0 | 0 |
| cg01278975 | 4 | 28 | 271 | 1 | 1023 | 0 | 0 | 0 | 0 | 0 | 0 | 0 |
| cg01330831 | 3 | 77 | 1245 | 1 | 0 | 0 | 0 | 0 | 0 | 0 | 0 | 0 |
| cg01341801 | 2 | 934 | 389 | 0 | 0 | 0 | 0 | 0 | 0 | 0 | 0 | 0 |
| cg01395541 | 2 | 439 | 884 | 0 | 0 | 0 | 0 | 0 | 0 | 0 | 0 | 0 |
| cg01479031 | 4 | 4 | 135 | 1 | 1183 | 0 | 0 | 0 | 0 | 0 | 0 | 0 |
| cg01521131 | 2 | 775 | 548 | 0 | 0 | 0 | 0 | 0 | 0 | 0 | 0 | 0 |
| cg01591343 | 4 | 194 | 1 | 578 | 550 | 0 | 0 | 0 | 0 | 0 | 0 | 0 |
| cg01655658 | 2 | 915 | 408 | 0 | 0 | 0 | 0 | 0 | 0 | 0 | 0 | 0 |
| cg01818016 | 3 | 7 | 67 | 1249 | 0 | 0 | 0 | 0 | 0 | 0 | 0 | 0 |
| cg01836455 | 2 | 113 | 1210 | 0 | 0 | 0 | 0 | 0 | 0 | 0 | 0 | 0 |
| cg01991743 | 5 | 38 | 192 | 1 | 1 | 1091 | 0 | 0 | 0 | 0 | 0 | 0 |
| cg02340312 | 4 | 19 | 105 | 1 | 1198 | 0 | 0 | 0 | 0 | 0 | 0 | 0 |
| cg02355653 | 2 | 112 | 1211 | 0 | 0 | 0 | 0 | 0 | 0 | 0 | 0 | 0 |
| cg02379549 | 4 | 82 | 2 | 2 | 1237 | 0 | 0 | 0 | 0 | 0 | 0 | 0 |
| cg02478793 | 2 | 201 | 1122 | 0 | 0 | 0 | 0 | 0 | 0 | 0 | 0 | 0 |
| cg02504993 | 3 | 13 | 274 | 1036 | 0 | 0 | 0 | 0 | 0 | 0 | 0 | 0 |
| cg02556146 | 3 | 5 | 90 | 1228 | 0 | 0 | 0 | 0 | 0 | 0 | 0 | 0 |
| cg02715072 | 4 | 1 | 1 | 96 | 1225 | 0 | 0 | 0 | 0 | 0 | 0 | 0 |
| cg02749948 | 5 | 27 | 1 | 270 | 1023 | 2 | 0 | 0 | 0 | 0 | 0 | 0 |
| cg02753444 | 3 | 7 | 176 | 1140 | 0 | 0 | 0 | 0 | 0 | 0 | 0 | 0 |
| cg02836767 | 5 | 3 | 1 | 1 | 92 | 1226 | 0 | 0 | 0 | 0 | 0 | 0 |
| cg02956194 | 4 | 171 | 1 | 548 | 603 | 0 | 0 | 0 | 0 | 0 | 0 | 0 |
| cg03071582 | 5 | 147 | 1 | 1 | 1 | 1173 | 0 | 0 | 0 | 0 | 0 | 0 |
| cg03115532 | 4 | 178 | 1 | 471 | 673 | 0 | 0 | 0 | 0 | 0 | 0 | 0 |
| cg03126739 | 3 | 94 | 332 | 897 | 0 | 0 | 0 | 0 | 0 | 0 | 0 | 0 |
| cg03187614 | 3 | 51 | 339 | 933 | 0 | 0 | 0 | 0 | 0 | 0 | 0 | 0 |
| cg03317682 | 3 | 3 | 91 | 1229 | 0 | 0 | 0 | 0 | 0 | 0 | 0 | 0 |
| cg03392100 | 6 | 20 | 1 | 1 | 1 | 297 | 1003 | 0 | 0 | 0 | 0 | 0 |
| cg03395511 | 3 | 242 | 1079 | 2 | 0 | 0 | 0 | 0 | 0 | 0 | 0 | 0 |
| cg03403996 | 3 | 41 | 339 | 943 | 0 | 0 | 0 | 0 | 0 | 0 | 0 | 0 |
| cg03570263 | 5 | 98 | 3 | 1 | 436 | 785 | 0 | 0 | 0 | 0 | 0 | 0 |
| cg04027045 | 4 | 10 | 1 | 91 | 1221 | 0 | 0 | 0 | 0 | 0 | 0 | 0 |
| cg04054096 | 5 | 2 | 1 | 1 | 105 | 1214 | 0 | 0 | 0 | 0 | 0 | 0 |
| cg04132418 | 4 | 251 | 1 | 1 | 1070 | 0 | 0 | 0 | 0 | 0 | 0 | 0 |
| cg04145681 | 5 | 568 | 1 | 538 | 1 | 215 | 0 | 0 | 0 | 0 | 0 | 0 |
| cg04462132 | 4 | 2 | 83 | 4 | 1234 | 0 | 0 | 0 | 0 | 0 | 0 | 0 |
| cg04922606 | 4 | 636 | 5 | 4 | 678 | 0 | 0 | 0 | 0 | 0 | 0 | 0 |
| cg05064044 | 3 | 243 | 1079 | 1 | 0 | 0 | 0 | 0 | 0 | 0 | 0 | 0 |
| cg05111645 | 3 | 43 | 372 | 908 | 0 | 0 | 0 | 0 | 0 | 0 | 0 | 0 |
| cg05123933 | 5 | 39 | 1 | 5 | 365 | 913 | 0 | 0 | 0 | 0 | 0 | 0 |
| cg05265771 | 4 | 7 | 109 | 2 | 1205 | 0 | 0 | 0 | 0 | 0 | 0 | 0 |
| cg05372765 | 4 | 23 | 126 | 1 | 1173 | 0 | 0 | 0 | 0 | 0 | 0 | 0 |
| cg05412957 | 2 | 193 | 1130 | 0 | 0 | 0 | 0 | 0 | 0 | 0 | 0 | 0 |
| cg05509228 | 2 | 96 | 1227 | 0 | 0 | 0 | 0 | 0 | 0 | 0 | 0 | 0 |
| cg05509820 | 3 | 31 | 275 | 1017 | 0 | 0 | 0 | 0 | 0 | 0 | 0 | 0 |
| cg05751055 | 4 | 271 | 4 | 2 | 1046 | 0 | 0 | 0 | 0 | 0 | 0 | 0 |
| cg06032337 | 3 | 460 | 7 | 856 | 0 | 0 | 0 | 0 | 0 | 0 | 0 | 0 |
| cg06293782 | 3 | 848 | 5 | 470 | 0 | 0 | 0 | 0 | 0 | 0 | 0 | 0 |
| cg06314883 | 3 | 17 | 199 | 1107 | 0 | 0 | 0 | 0 | 0 | 0 | 0 | 0 |
| cg06330797 | 3 | 69 | 365 | 889 | 0 | 0 | 0 | 0 | 0 | 0 | 0 | 0 |
| cg06458771 | 3 | 73 | 371 | 879 | 0 | 0 | 0 | 0 | 0 | 0 | 0 | 0 |
| cg06484169 | 3 | 278 | 1 | 1044 | 0 | 0 | 0 | 0 | 0 | 0 | 0 | 0 |
| cg06503981 | 2 | 139 | 1184 | 0 | 0 | 0 | 0 | 0 | 0 | 0 | 0 | 0 |
| cg06699489 | 4 | 1 | 179 | 1 | 1142 | 0 | 0 | 0 | 0 | 0 | 0 | 0 |
| cg06736674 | 4 | 8 | 106 | 1 | 1208 | 0 | 0 | 0 | 0 | 0 | 0 | 0 |
| cg06773563 | 3 | 1 | 83 | 1239 | 0 | 0 | 0 | 0 | 0 | 0 | 0 | 0 |
| cg06807168 | 3 | 9 | 67 | 1247 | 0 | 0 | 0 | 0 | 0 | 0 | 0 | 0 |
| cg06833981 | 4 | 22 | 1 | 245 | 1055 | 0 | 0 | 0 | 0 | 0 | 0 | 0 |
| cg06937619 | 3 | 2 | 65 | 1256 | 0 | 0 | 0 | 0 | 0 | 0 | 0 | 0 |
| cg07332563 | 2 | 241 | 1082 | 0 | 0 | 0 | 0 | 0 | 0 | 0 | 0 | 0 |
| cg07365741 | 2 | 135 | 1188 | 0 | 0 | 0 | 0 | 0 | 0 | 0 | 0 | 0 |
| cg07383496 | 4 | 6 | 103 | 1 | 1213 | 0 | 0 | 0 | 0 | 0 | 0 | 0 |
| cg07389699 | 2 | 562 | 761 | 0 | 0 | 0 | 0 | 0 | 0 | 0 | 0 | 0 |
| cg07414487 | 2 | 90 | 1233 | 0 | 0 | 0 | 0 | 0 | 0 | 0 | 0 | 0 |
| cg07467861 | 3 | 2 | 90 | 1231 | 0 | 0 | 0 | 0 | 0 | 0 | 0 | 0 |
| cg07480955 | 2 | 236 | 1087 | 0 | 0 | 0 | 0 | 0 | 0 | 0 | 0 | 0 |
| cg07482223 | 6 | 104 | 1 | 2 | 446 | 1 | 769 | 0 | 0 | 0 | 0 | 0 |
| cg07586863 | 5 | 9 | 2 | 101 | 2 | 1209 | 0 | 0 | 0 | 0 | 0 | 0 |
| cg07732331 | 6 | 6 | 1 | 2 | 1 | 141 | 1172 | 0 | 0 | 0 | 0 | 0 |
| cg07791065 | 4 | 143 | 1 | 1 | 1178 | 0 | 0 | 0 | 0 | 0 | 0 | 0 |
| cg07895329 | 4 | 25 | 3 | 296 | 999 | 0 | 0 | 0 | 0 | 0 | 0 | 0 |
| cg08072458 | 3 | 1204 | 116 | 3 | 0 | 0 | 0 | 0 | 0 | 0 | 0 | 0 |
| cg08198851 | 2 | 299 | 1024 | 0 | 0 | 0 | 0 | 0 | 0 | 0 | 0 | 0 |
| cg08308214 | 4 | 2 | 1 | 75 | 1245 | 0 | 0 | 0 | 0 | 0 | 0 | 0 |
| cg08412936 | 3 | 93 | 2 | 1228 | 0 | 0 | 0 | 0 | 0 | 0 | 0 | 0 |
| cg08996597 | 5 | 233 | 1 | 1 | 440 | 648 | 0 | 0 | 0 | 0 | 0 | 0 |
| cg09050820 | 2 | 1089 | 234 | 0 | 0 | 0 | 0 | 0 | 0 | 0 | 0 | 0 |
| cg09199225 | 3 | 1 | 104 | 1218 | 0 | 0 | 0 | 0 | 0 | 0 | 0 | 0 |
| cg09247979 | 7 | 374 | 1 | 485 | 1 | 1 | 457 | 4 | 0 | 0 | 0 | 0 |
| cg09382842 | 2 | 772 | 551 | 0 | 0 | 0 | 0 | 0 | 0 | 0 | 0 | 0 |
| cg09510698 | 3 | 95 | 1 | 1227 | 0 | 0 | 0 | 0 | 0 | 0 | 0 | 0 |
| cg09741902 | 4 | 8 | 1 | 62 | 1252 | 0 | 0 | 0 | 0 | 0 | 0 | 0 |
| cg09900440 | 4 | 80 | 1 | 415 | 827 | 0 | 0 | 0 | 0 | 0 | 0 | 0 |
| cg10471638 | 3 | 112 | 2 | 1209 | 0 | 0 | 0 | 0 | 0 | 0 | 0 | 0 |
| cg10482512 | 2 | 308 | 1015 | 0 | 0 | 0 | 0 | 0 | 0 | 0 | 0 | 0 |
| cg10619365 | 3 | 5 | 85 | 1233 | 0 | 0 | 0 | 0 | 0 | 0 | 0 | 0 |
| cg10758676 | 3 | 8 | 80 | 1235 | 0 | 0 | 0 | 0 | 0 | 0 | 0 | 0 |
| cg10863737 | 4 | 61 | 1 | 434 | 827 | 0 | 0 | 0 | 0 | 0 | 0 | 0 |
| cg10985055 | 4 | 135 | 2 | 431 | 755 | 0 | 0 | 0 | 0 | 0 | 0 | 0 |
| cg10991855 | 6 | 11 | 1 | 1 | 1 | 151 | 1158 | 0 | 0 | 0 | 0 | 0 |
| cg11036359 | 3 | 230 | 3 | 1090 | 0 | 0 | 0 | 0 | 0 | 0 | 0 | 0 |
| cg11040238 | 3 | 4 | 109 | 1210 | 0 | 0 | 0 | 0 | 0 | 0 | 0 | 0 |
| cg11268327 | 4 | 121 | 1 | 399 | 802 | 0 | 0 | 0 | 0 | 0 | 0 | 0 |
| cg11290949 | 3 | 381 | 2 | 940 | 0 | 0 | 0 | 0 | 0 | 0 | 0 | 0 |
| cg11387340 | 3 | 1239 | 82 | 2 | 0 | 0 | 0 | 0 | 0 | 0 | 0 | 0 |
| cg11400162 | 4 | 451 | 1 | 603 | 268 | 0 | 0 | 0 | 0 | 0 | 0 | 0 |
| cg11401394 | 4 | 18 | 172 | 1 | 1132 | 0 | 0 | 0 | 0 | 0 | 0 | 0 |
| cg11404906 | 2 | 395 | 928 | 0 | 0 | 0 | 0 | 0 | 0 | 0 | 0 | 0 |
| cg11508013 | 4 | 142 | 10 | 2 | 1169 | 0 | 0 | 0 | 0 | 0 | 0 | 0 |
| cg11752699 | 2 | 938 | 385 | 0 | 0 | 0 | 0 | 0 | 0 | 0 | 0 | 0 |
| cg11784298 | 2 | 436 | 887 | 0 | 0 | 0 | 0 | 0 | 0 | 0 | 0 | 0 |
| cg11807238 | 5 | 7 | 1 | 1 | 153 | 1161 | 0 | 0 | 0 | 0 | 0 | 0 |
| cg11867651 | 2 | 773 | 550 | 0 | 0 | 0 | 0 | 0 | 0 | 0 | 0 | 0 |
| cg11881038 | 4 | 24 | 227 | 1071 | 1 | 0 | 0 | 0 | 0 | 0 | 0 | 0 |
| cg11884832 | 3 | 797 | 2 | 524 | 0 | 0 | 0 | 0 | 0 | 0 | 0 | 0 |
| cg12035144 | 2 | 268 | 1055 | 0 | 0 | 0 | 0 | 0 | 0 | 0 | 0 | 0 |
| cg12227626 | 4 | 2 | 126 | 2 | 1193 | 0 | 0 | 0 | 0 | 0 | 0 | 0 |
| cg12284268 | 3 | 2 | 78 | 1243 | 0 | 0 | 0 | 0 | 0 | 0 | 0 | 0 |
| cg12474013 | 2 | 184 | 1139 | 0 | 0 | 0 | 0 | 0 | 0 | 0 | 0 | 0 |
| cg12669314 | 2 | 80 | 1243 | 0 | 0 | 0 | 0 | 0 | 0 | 0 | 0 | 0 |
| cg12858166 | 3 | 220 | 1 | 1102 | 0 | 0 | 0 | 0 | 0 | 0 | 0 | 0 |
| cg12999291 | 5 | 24 | 1 | 2 | 329 | 967 | 0 | 0 | 0 | 0 | 0 | 0 |
| cg13354679 | 4 | 2 | 3 | 75 | 1243 | 0 | 0 | 0 | 0 | 0 | 0 | 0 |
| cg13604933 | 3 | 77 | 1 | 1245 | 0 | 0 | 0 | 0 | 0 | 0 | 0 | 0 |
| cg13661648 | 2 | 221 | 1102 | 0 | 0 | 0 | 0 | 0 | 0 | 0 | 0 | 0 |
| cg13864354 | 3 | 3 | 65 | 1255 | 0 | 0 | 0 | 0 | 0 | 0 | 0 | 0 |
| cg13885788 | 6 | 129 | 2 | 1 | 517 | 1 | 673 | 0 | 0 | 0 | 0 | 0 |
| cg13966843 | 6 | 20 | 1 | 1 | 1 | 262 | 1038 | 0 | 0 | 0 | 0 | 0 |
| cg14044669 | 3 | 243 | 6 | 1074 | 0 | 0 | 0 | 0 | 0 | 0 | 0 | 0 |
| cg14323910 | 4 | 463 | 1 | 12 | 847 | 0 | 0 | 0 | 0 | 0 | 0 | 0 |
| cg14366007 | 4 | 5 | 2 | 176 | 1140 | 0 | 0 | 0 | 0 | 0 | 0 | 0 |
| cg14373797 | 5 | 44 | 1 | 266 | 1 | 1011 | 0 | 0 | 0 | 0 | 0 | 0 |
| cg14782559 | 3 | 109 | 467 | 747 | 0 | 0 | 0 | 0 | 0 | 0 | 0 | 0 |
| cg15027633 | 4 | 2 | 109 | 3 | 1209 | 0 | 0 | 0 | 0 | 0 | 0 | 0 |
| cg15029183 | 3 | 172 | 2 | 1149 | 0 | 0 | 0 | 0 | 0 | 0 | 0 | 0 |
| cg15042082 | 4 | 12 | 1 | 225 | 1085 | 0 | 0 | 0 | 0 | 0 | 0 | 0 |
| cg15365500 | 2 | 459 | 864 | 0 | 0 | 0 | 0 | 0 | 0 | 0 | 0 | 0 |
| cg15383120 | 3 | 242 | 1080 | 1 | 0 | 0 | 0 | 0 | 0 | 0 | 0 | 0 |
| cg15790767 | 5 | 7 | 1 | 71 | 1 | 1243 | 0 | 0 | 0 | 0 | 0 | 0 |
| cg15831664 | 2 | 67 | 1256 | 0 | 0 | 0 | 0 | 0 | 0 | 0 | 0 | 0 |
| cg15979214 | 4 | 17 | 261 | 2 | 1043 | 0 | 0 | 0 | 0 | 0 | 0 | 0 |
| cg16035267 | 4 | 6 | 124 | 3 | 1190 | 0 | 0 | 0 | 0 | 0 | 0 | 0 |
| cg16083558 | 3 | 7 | 135 | 1181 | 0 | 0 | 0 | 0 | 0 | 0 | 0 | 0 |
| cg16107389 | 3 | 6 | 64 | 1253 | 0 | 0 | 0 | 0 | 0 | 0 | 0 | 0 |
| cg16149936 | 4 | 4 | 86 | 1 | 1232 | 0 | 0 | 0 | 0 | 0 | 0 | 0 |
| cg16241932 | 6 | 129 | 2 | 1 | 453 | 1 | 737 | 0 | 0 | 0 | 0 | 0 |
| cg16471877 | 4 | 237 | 613 | 1 | 472 | 0 | 0 | 0 | 0 | 0 | 0 | 0 |
| cg16507569 | 5 | 19 | 1 | 211 | 1 | 1091 | 0 | 0 | 0 | 0 | 0 | 0 |
| cg16527629 | 4 | 444 | 1 | 1 | 877 | 0 | 0 | 0 | 0 | 0 | 0 | 0 |
| cg16655343 | 5 | 207 | 1 | 617 | 497 | 1 | 0 | 0 | 0 | 0 | 0 | 0 |
| cg16870958 | 3 | 10 | 104 | 1209 | 0 | 0 | 0 | 0 | 0 | 0 | 0 | 0 |
| cg16899306 | 3 | 4 | 152 | 1167 | 0 | 0 | 0 | 0 | 0 | 0 | 0 | 0 |
| cg17009574 | 2 | 1159 | 164 | 0 | 0 | 0 | 0 | 0 | 0 | 0 | 0 | 0 |
| cg17018201 | 3 | 1 | 74 | 1248 | 0 | 0 | 0 | 0 | 0 | 0 | 0 | 0 |
| cg17129519 | 4 | 16 | 1 | 210 | 1096 | 0 | 0 | 0 | 0 | 0 | 0 | 0 |
| cg17273096 | 4 | 80 | 3 | 1239 | 1 | 0 | 0 | 0 | 0 | 0 | 0 | 0 |
| cg17351927 | 4 | 75 | 1 | 443 | 804 | 0 | 0 | 0 | 0 | 0 | 0 | 0 |
| cg17416722 | 2 | 929 | 394 | 0 | 0 | 0 | 0 | 0 | 0 | 0 | 0 | 0 |
| cg17678867 | 4 | 9 | 4 | 117 | 1193 | 0 | 0 | 0 | 0 | 0 | 0 | 0 |
| cg17729655 | 2 | 236 | 1087 | 0 | 0 | 0 | 0 | 0 | 0 | 0 | 0 | 0 |
| cg17763566 | 3 | 44 | 363 | 916 | 0 | 0 | 0 | 0 | 0 | 0 | 0 | 0 |
| cg17770035 | 2 | 658 | 665 | 0 | 0 | 0 | 0 | 0 | 0 | 0 | 0 | 0 |
| cg17857094 | 2 | 71 | 1252 | 0 | 0 | 0 | 0 | 0 | 0 | 0 | 0 | 0 |
| cg18026667 | 4 | 6 | 1 | 109 | 1207 | 0 | 0 | 0 | 0 | 0 | 0 | 0 |
| cg18093864 | 3 | 3 | 73 | 1247 | 0 | 0 | 0 | 0 | 0 | 0 | 0 | 0 |
| cg18182981 | 3 | 66 | 1 | 1256 | 0 | 0 | 0 | 0 | 0 | 0 | 0 | 0 |
| cg18572898 | 5 | 558 | 1 | 586 | 2 | 176 | 0 | 0 | 0 | 0 | 0 | 0 |
| cg18584440 | 5 | 25 | 1 | 1 | 346 | 950 | 0 | 0 | 0 | 0 | 0 | 0 |
| cg18686576 | 3 | 1231 | 88 | 4 | 0 | 0 | 0 | 0 | 0 | 0 | 0 | 0 |
| cg18698799 | 2 | 181 | 1142 | 0 | 0 | 0 | 0 | 0 | 0 | 0 | 0 | 0 |
| cg18816397 | 4 | 1 | 99 | 1222 | 1 | 0 | 0 | 0 | 0 | 0 | 0 | 0 |
| cg19178509 | 4 | 86 | 1 | 1235 | 1 | 0 | 0 | 0 | 0 | 0 | 0 | 0 |
| cg19179910 | 3 | 14 | 276 | 1033 | 0 | 0 | 0 | 0 | 0 | 0 | 0 | 0 |
| cg19236328 | 4 | 35 | 284 | 1 | 1003 | 0 | 0 | 0 | 0 | 0 | 0 | 0 |
| cg19300401 | 5 | 161 | 1 | 603 | 4 | 554 | 0 | 0 | 0 | 0 | 0 | 0 |
| cg19367859 | 5 | 15 | 1 | 198 | 1 | 1108 | 0 | 0 | 0 | 0 | 0 | 0 |
| cg19455396 | 5 | 84 | 2 | 1 | 1 | 1235 | 0 | 0 | 0 | 0 | 0 | 0 |
| cg19577958 | 4 | 318 | 1 | 584 | 420 | 0 | 0 | 0 | 0 | 0 | 0 | 0 |
| cg19596870 | 4 | 1 | 189 | 2 | 1131 | 0 | 0 | 0 | 0 | 0 | 0 | 0 |
| cg19774683 | 3 | 931 | 1 | 391 | 0 | 0 | 0 | 0 | 0 | 0 | 0 | 0 |
| cg19982221 | 3 | 1 | 66 | 1256 | 0 | 0 | 0 | 0 | 0 | 0 | 0 | 0 |
| cg20073472 | 4 | 76 | 1 | 449 | 797 | 0 | 0 | 0 | 0 | 0 | 0 | 0 |
| cg20106077 | 5 | 130 | 3 | 182 | 1 | 1007 | 0 | 0 | 0 | 0 | 0 | 0 |
| cg20370184 | 5 | 515 | 614 | 1 | 1 | 192 | 0 | 0 | 0 | 0 | 0 | 0 |
| cg20554557 | 2 | 69 | 1254 | 0 | 0 | 0 | 0 | 0 | 0 | 0 | 0 | 0 |
| cg20631714 | 5 | 1 | 1 | 1 | 73 | 1247 | 0 | 0 | 0 | 0 | 0 | 0 |
| cg20968290 | 2 | 136 | 1187 | 0 | 0 | 0 | 0 | 0 | 0 | 0 | 0 | 0 |
| cg20981163 | 2 | 274 | 1049 | 0 | 0 | 0 | 0 | 0 | 0 | 0 | 0 | 0 |
| cg21109045 | 5 | 3 | 9 | 1 | 87 | 1223 | 0 | 0 | 0 | 0 | 0 | 0 |
| cg21234342 | 4 | 176 | 2 | 5 | 1140 | 0 | 0 | 0 | 0 | 0 | 0 | 0 |
| cg21241195 | 4 | 18 | 272 | 1 | 1032 | 0 | 0 | 0 | 0 | 0 | 0 | 0 |
| cg21401376 | 4 | 3 | 66 | 1 | 1253 | 0 | 0 | 0 | 0 | 0 | 0 | 0 |
| cg21438527 | 4 | 1227 | 1 | 87 | 8 | 0 | 0 | 0 | 0 | 0 | 0 | 0 |
| cg21485521 | 4 | 1 | 4 | 116 | 1202 | 0 | 0 | 0 | 0 | 0 | 0 | 0 |
| cg21492127 | 3 | 99 | 284 | 940 | 0 | 0 | 0 | 0 | 0 | 0 | 0 | 0 |
| cg21548813 | 4 | 242 | 1079 | 1 | 1 | 0 | 0 | 0 | 0 | 0 | 0 | 0 |
| cg21696374 | 3 | 361 | 1 | 961 | 0 | 0 | 0 | 0 | 0 | 0 | 0 | 0 |
| cg21796170 | 4 | 38 | 306 | 1 | 978 | 0 | 0 | 0 | 0 | 0 | 0 | 0 |
| cg22274273 | 4 | 295 | 6 | 681 | 341 | 0 | 0 | 0 | 0 | 0 | 0 | 0 |
| cg22375320 | 5 | 8 | 1 | 1 | 113 | 1200 | 0 | 0 | 0 | 0 | 0 | 0 |
| cg22410568 | 4 | 1 | 1 | 70 | 1251 | 0 | 0 | 0 | 0 | 0 | 0 | 0 |
| cg22798247 | 3 | 1 | 80 | 1242 | 0 | 0 | 0 | 0 | 0 | 0 | 0 | 0 |
| cg22849321 | 6 | 1214 | 2 | 1 | 94 | 10 | 2 | 0 | 0 | 0 | 0 | 0 |
| cg22897634 | 4 | 1 | 2 | 71 | 1249 | 0 | 0 | 0 | 0 | 0 | 0 | 0 |
| cg22935422 | 4 | 4 | 80 | 2 | 1237 | 0 | 0 | 0 | 0 | 0 | 0 | 0 |
| cg23069046 | 3 | 38 | 277 | 1008 | 0 | 0 | 0 | 0 | 0 | 0 | 0 | 0 |
| cg23333490 | 2 | 274 | 1049 | 0 | 0 | 0 | 0 | 0 | 0 | 0 | 0 | 0 |
| cg23368162 | 4 | 1 | 66 | 1 | 1255 | 0 | 0 | 0 | 0 | 0 | 0 | 0 |
| cg23431098 | 4 | 7 | 1 | 81 | 1234 | 0 | 0 | 0 | 0 | 0 | 0 | 0 |
| cg23603995 | 4 | 181 | 1 | 491 | 650 | 0 | 0 | 0 | 0 | 0 | 0 | 0 |
| cg23687434 | 5 | 85 | 2 | 284 | 2 | 950 | 0 | 0 | 0 | 0 | 0 | 0 |
| cg23739027 | 3 | 6 | 75 | 1242 | 0 | 0 | 0 | 0 | 0 | 0 | 0 | 0 |
| cg23892028 | 3 | 943 | 340 | 40 | 0 | 0 | 0 | 0 | 0 | 0 | 0 | 0 |
| cg23974730 | 4 | 1167 | 146 | 1 | 9 | 0 | 0 | 0 | 0 | 0 | 0 | 0 |
| cg24018148 | 4 | 221 | 1 | 633 | 468 | 0 | 0 | 0 | 0 | 0 | 0 | 0 |
| cg24080129 | 6 | 717 | 1 | 509 | 1 | 1 | 94 | 0 | 0 | 0 | 0 | 0 |
| cg24100293 | 3 | 26 | 259 | 1038 | 0 | 0 | 0 | 0 | 0 | 0 | 0 | 0 |
| cg24131442 | 2 | 177 | 1146 | 0 | 0 | 0 | 0 | 0 | 0 | 0 | 0 | 0 |
| cg24299087 | 3 | 82 | 1 | 1240 | 0 | 0 | 0 | 0 | 0 | 0 | 0 | 0 |
| cg24392672 | 2 | 77 | 1246 | 0 | 0 | 0 | 0 | 0 | 0 | 0 | 0 | 0 |
| cg24407607 | 4 | 180 | 3 | 1 | 1139 | 0 | 0 | 0 | 0 | 0 | 0 | 0 |
| cg24697433 | 5 | 100 | 1 | 3 | 468 | 751 | 0 | 0 | 0 | 0 | 0 | 0 |
| cg24888609 | 3 | 8 | 83 | 1232 | 0 | 0 | 0 | 0 | 0 | 0 | 0 | 0 |
| cg24926791 | 4 | 86 | 535 | 1 | 701 | 0 | 0 | 0 | 0 | 0 | 0 | 0 |
| cg25046571 | 3 | 352 | 1 | 970 | 0 | 0 | 0 | 0 | 0 | 0 | 0 | 0 |
| cg25099095 | 5 | 392 | 1 | 645 | 3 | 282 | 0 | 0 | 0 | 0 | 0 | 0 |
| cg25140213 | 4 | 938 | 1 | 383 | 1 | 0 | 0 | 0 | 0 | 0 | 0 | 0 |
| cg25202367 | 4 | 32 | 108 | 1 | 1182 | 0 | 0 | 0 | 0 | 0 | 0 | 0 |
| cg25213055 | 4 | 7 | 106 | 1 | 1209 | 0 | 0 | 0 | 0 | 0 | 0 | 0 |
| cg25270367 | 6 | 81 | 3 | 1 | 3 | 1 | 1234 | 0 | 0 | 0 | 0 | 0 |
| cg26005482 | 3 | 5 | 74 | 1244 | 0 | 0 | 0 | 0 | 0 | 0 | 0 | 0 |
| cg26021304 | 3 | 1236 | 1 | 86 | 0 | 0 | 0 | 0 | 0 | 0 | 0 | 0 |
| cg26155617 | 3 | 9 | 82 | 1232 | 0 | 0 | 0 | 0 | 0 | 0 | 0 | 0 |
| cg26296371 | 5 | 195 | 2 | 1 | 1 | 1124 | 0 | 0 | 0 | 0 | 0 | 0 |
| cg26520712 | 3 | 4 | 64 | 1255 | 0 | 0 | 0 | 0 | 0 | 0 | 0 | 0 |
| cg26566189 | 4 | 1057 | 1 | 243 | 22 | 0 | 0 | 0 | 0 | 0 | 0 | 0 |
| cg26576978 | 4 | 53 | 1 | 349 | 920 | 0 | 0 | 0 | 0 | 0 | 0 | 0 |
| cg26739327 | 3 | 201 | 1 | 1121 | 0 | 0 | 0 | 0 | 0 | 0 | 0 | 0 |
| cg26820259 | 6 | 143 | 1 | 446 | 1 | 3 | 729 | 0 | 0 | 0 | 0 | 0 |
| cg26850117 | 3 | 177 | 1 | 1145 | 0 | 0 | 0 | 0 | 0 | 0 | 0 | 0 |
| cg27081049 | 2 | 1197 | 126 | 0 | 0 | 0 | 0 | 0 | 0 | 0 | 0 | 0 |
| cg27118080 | 7 | 1 | 1 | 1 | 2 | 2 | 99 | 1217 | 0 | 0 | 0 | 0 |
| cg27154731 | 5 | 168 | 2 | 2 | 479 | 672 | 0 | 0 | 0 | 0 | 0 | 0 |
| cg27225663 | 3 | 78 | 2 | 1243 | 0 | 0 | 0 | 0 | 0 | 0 | 0 | 0 |
| cg27230769 | 3 | 499 | 612 | 212 | 0 | 0 | 0 | 0 | 0 | 0 | 0 | 0 |
| cg27406070 | 4 | 12 | 238 | 1 | 1072 | 0 | 0 | 0 | 0 | 0 | 0 | 0 |
| cg27512707 | 5 | 3 | 3 | 77 | 1 | 1239 | 0 | 0 | 0 | 0 | 0 | 0 |
| cg00327544 | 4 | 12 | 1 | 94 | 1216 | 0 | 0 | 0 | 0 | 0 | 0 | 0 |
| cg00449715 | 4 | 3 | 1 | 79 | 1240 | 0 | 0 | 0 | 0 | 0 | 0 | 0 |
| cg00453190 | 2 | 143 | 1180 | 0 | 0 | 0 | 0 | 0 | 0 | 0 | 0 | 0 |
| cg00571519 | 2 | 120 | 1203 | 0 | 0 | 0 | 0 | 0 | 0 | 0 | 0 | 0 |
| cg00581720 | 3 | 13 | 116 | 1194 | 0 | 0 | 0 | 0 | 0 | 0 | 0 | 0 |
| cg00843795 | 4 | 22 | 2 | 250 | 1049 | 0 | 0 | 0 | 0 | 0 | 0 | 0 |
| cg01139696 | 5 | 2 | 3 | 61 | 5 | 1252 | 0 | 0 | 0 | 0 | 0 | 0 |
| cg01156747 | 4 | 120 | 532 | 7 | 664 | 0 | 0 | 0 | 0 | 0 | 0 | 0 |
| cg01213645 | 4 | 1 | 1 | 69 | 1252 | 0 | 0 | 0 | 0 | 0 | 0 | 0 |
| cg01338296 | 3 | 5 | 139 | 1179 | 0 | 0 | 0 | 0 | 0 | 0 | 0 | 0 |
| cg01350803 | 3 | 356 | 4 | 963 | 0 | 0 | 0 | 0 | 0 | 0 | 0 | 0 |
| cg01601712 | 3 | 115 | 1 | 1207 | 0 | 0 | 0 | 0 | 0 | 0 | 0 | 0 |
| cg01832665 | 6 | 5 | 2 | 1 | 133 | 1 | 1181 | 0 | 0 | 0 | 0 | 0 |
| cg02307470 | 4 | 2 | 1 | 71 | 1249 | 0 | 0 | 0 | 0 | 0 | 0 | 0 |
| cg02342754 | 5 | 1 | 6 | 1 | 111 | 1204 | 0 | 0 | 0 | 0 | 0 | 0 |
| cg02355809 | 3 | 189 | 2 | 1132 | 0 | 0 | 0 | 0 | 0 | 0 | 0 | 0 |
| cg02394572 | 4 | 140 | 1 | 1 | 1181 | 0 | 0 | 0 | 0 | 0 | 0 | 0 |
| cg02422282 | 4 | 3 | 65 | 1 | 1254 | 0 | 0 | 0 | 0 | 0 | 0 | 0 |
| cg02623234 | 5 | 38 | 1 | 1 | 323 | 960 | 0 | 0 | 0 | 0 | 0 | 0 |
| cg02627240 | 3 | 524 | 1 | 798 | 0 | 0 | 0 | 0 | 0 | 0 | 0 | 0 |
| cg02850468 | 4 | 14 | 2 | 156 | 1151 | 0 | 0 | 0 | 0 | 0 | 0 | 0 |
| cg02879083 | 2 | 82 | 1241 | 0 | 0 | 0 | 0 | 0 | 0 | 0 | 0 | 0 |
| cg02922510 | 3 | 75 | 1247 | 1 | 0 | 0 | 0 | 0 | 0 | 0 | 0 | 0 |
| cg03075889 | 6 | 152 | 2 | 473 | 1 | 2 | 693 | 0 | 0 | 0 | 0 | 0 |
| cg03119308 | 3 | 224 | 484 | 615 | 0 | 0 | 0 | 0 | 0 | 0 | 0 | 0 |
| cg03122926 | 4 | 337 | 526 | 84 | 376 | 0 | 0 | 0 | 0 | 0 | 0 | 0 |
| cg03723481 | 3 | 25 | 314 | 984 | 0 | 0 | 0 | 0 | 0 | 0 | 0 | 0 |
| cg03779213 | 3 | 4 | 92 | 1227 | 0 | 0 | 0 | 0 | 0 | 0 | 0 | 0 |
| cg03812172 | 3 | 56 | 378 | 889 | 0 | 0 | 0 | 0 | 0 | 0 | 0 | 0 |
| cg03900028 | 5 | 309 | 1 | 1 | 607 | 405 | 0 | 0 | 0 | 0 | 0 | 0 |
| cg04064735 | 5 | 74 | 1 | 2 | 1 | 1245 | 0 | 0 | 0 | 0 | 0 | 0 |
| cg04090468 | 3 | 79 | 442 | 802 | 0 | 0 | 0 | 0 | 0 | 0 | 0 | 0 |
| cg04263740 | 4 | 147 | 1 | 1 | 1174 | 0 | 0 | 0 | 0 | 0 | 0 | 0 |
| cg04456492 | 5 | 192 | 1 | 632 | 495 | 3 | 0 | 0 | 0 | 0 | 0 | 0 |
| cg04725144 | 3 | 4 | 89 | 1230 | 0 | 0 | 0 | 0 | 0 | 0 | 0 | 0 |
| cg04752818 | 3 | 16 | 153 | 1154 | 0 | 0 | 0 | 0 | 0 | 0 | 0 | 0 |
| cg04954056 | 3 | 2 | 88 | 1233 | 0 | 0 | 0 | 0 | 0 | 0 | 0 | 0 |
| cg05232852 | 4 | 9 | 1 | 89 | 1224 | 0 | 0 | 0 | 0 | 0 | 0 | 0 |
| cg05340866 | 5 | 292 | 1 | 1 | 1028 | 1 | 0 | 0 | 0 | 0 | 0 | 0 |
| cg05364766 | 3 | 202 | 2 | 1119 | 0 | 0 | 0 | 0 | 0 | 0 | 0 | 0 |
| cg05455372 | 3 | 163 | 2 | 1158 | 0 | 0 | 0 | 0 | 0 | 0 | 0 | 0 |
| cg05507414 | 4 | 1 | 1 | 92 | 1229 | 0 | 0 | 0 | 0 | 0 | 0 | 0 |
| cg05593887 | 5 | 127 | 1 | 1 | 555 | 639 | 0 | 0 | 0 | 0 | 0 | 0 |
| cg05675570 | 4 | 3 | 2 | 85 | 1233 | 0 | 0 | 0 | 0 | 0 | 0 | 0 |
| cg06012903 | 3 | 521 | 798 | 4 | 0 | 0 | 0 | 0 | 0 | 0 | 0 | 0 |
| cg06093861 | 2 | 297 | 1026 | 0 | 0 | 0 | 0 | 0 | 0 | 0 | 0 | 0 |
| cg06112204 | 3 | 17 | 271 | 1035 | 0 | 0 | 0 | 0 | 0 | 0 | 0 | 0 |
| cg06779232 | 5 | 1 | 1 | 76 | 1 | 1244 | 0 | 0 | 0 | 0 | 0 | 0 |
| cg06960717 | 5 | 103 | 3 | 1 | 1 | 1215 | 0 | 0 | 0 | 0 | 0 | 0 |
| cg07235057 | 3 | 16 | 134 | 1173 | 0 | 0 | 0 | 0 | 0 | 0 | 0 | 0 |
| cg07304760 | 3 | 66 | 353 | 904 | 0 | 0 | 0 | 0 | 0 | 0 | 0 | 0 |
| cg07480176 | 2 | 377 | 946 | 0 | 0 | 0 | 0 | 0 | 0 | 0 | 0 | 0 |
| cg07486666 | 3 | 9 | 101 | 1213 | 0 | 0 | 0 | 0 | 0 | 0 | 0 | 0 |
| cg07803860 | 4 | 13 | 157 | 2 | 1151 | 0 | 0 | 0 | 0 | 0 | 0 | 0 |
| cg07846874 | 3 | 123 | 2 | 1198 | 0 | 0 | 0 | 0 | 0 | 0 | 0 | 0 |
| cg07878625 | 2 | 383 | 940 | 0 | 0 | 0 | 0 | 0 | 0 | 0 | 0 | 0 |
| cg07998236 | 2 | 99 | 1224 | 0 | 0 | 0 | 0 | 0 | 0 | 0 | 0 | 0 |
| cg08119527 | 2 | 220 | 1103 | 0 | 0 | 0 | 0 | 0 | 0 | 0 | 0 | 0 |
| cg08320316 | 3 | 113 | 1 | 1209 | 0 | 0 | 0 | 0 | 0 | 0 | 0 | 0 |
| cg08355157 | 4 | 129 | 2 | 1189 | 3 | 0 | 0 | 0 | 0 | 0 | 0 | 0 |
| cg08458132 | 5 | 167 | 1 | 1 | 1 | 1153 | 0 | 0 | 0 | 0 | 0 | 0 |
| cg08570077 | 5 | 116 | 1 | 544 | 1 | 661 | 0 | 0 | 0 | 0 | 0 | 0 |
| cg08693140 | 2 | 197 | 1126 | 0 | 0 | 0 | 0 | 0 | 0 | 0 | 0 | 0 |
| cg09125311 | 3 | 3 | 116 | 1204 | 0 | 0 | 0 | 0 | 0 | 0 | 0 | 0 |
| cg09281805 | 3 | 262 | 542 | 519 | 0 | 0 | 0 | 0 | 0 | 0 | 0 | 0 |
| cg09295021 | 3 | 18 | 163 | 1142 | 0 | 0 | 0 | 0 | 0 | 0 | 0 | 0 |
| cg09714852 | 4 | 30 | 1 | 245 | 1047 | 0 | 0 | 0 | 0 | 0 | 0 | 0 |
| cg10030633 | 3 | 7 | 198 | 1118 | 0 | 0 | 0 | 0 | 0 | 0 | 0 | 0 |
| cg10117599 | 4 | 475 | 1 | 580 | 267 | 0 | 0 | 0 | 0 | 0 | 0 | 0 |
| cg10146442 | 4 | 32 | 1 | 346 | 944 | 0 | 0 | 0 | 0 | 0 | 0 | 0 |
| cg10187601 | 2 | 119 | 1204 | 0 | 0 | 0 | 0 | 0 | 0 | 0 | 0 | 0 |
| cg10528826 | 3 | 2 | 173 | 1148 | 0 | 0 | 0 | 0 | 0 | 0 | 0 | 0 |
| cg10736303 | 5 | 5 | 1 | 126 | 1 | 1190 | 0 | 0 | 0 | 0 | 0 | 0 |
| cg10750306 | 3 | 226 | 3 | 1094 | 0 | 0 | 0 | 0 | 0 | 0 | 0 | 0 |
| cg10823511 | 3 | 7 | 151 | 1165 | 0 | 0 | 0 | 0 | 0 | 0 | 0 | 0 |
| cg10900271 | 4 | 298 | 2 | 1 | 1022 | 0 | 0 | 0 | 0 | 0 | 0 | 0 |
| cg10978526 | 5 | 44 | 1 | 1 | 286 | 991 | 0 | 0 | 0 | 0 | 0 | 0 |
| cg11010744 | 3 | 379 | 1 | 943 | 0 | 0 | 0 | 0 | 0 | 0 | 0 | 0 |
| cg11276189 | 3 | 12 | 241 | 1070 | 0 | 0 | 0 | 0 | 0 | 0 | 0 | 0 |
| cg11607219 | 4 | 39 | 262 | 3 | 1019 | 0 | 0 | 0 | 0 | 0 | 0 | 0 |
| cg11627968 | 4 | 117 | 413 | 2 | 791 | 0 | 0 | 0 | 0 | 0 | 0 | 0 |
| cg11816001 | 4 | 9 | 90 | 1 | 1223 | 0 | 0 | 0 | 0 | 0 | 0 | 0 |
| cg11835544 | 3 | 78 | 1 | 1244 | 0 | 0 | 0 | 0 | 0 | 0 | 0 | 0 |
| cg11957130 | 3 | 449 | 3 | 871 | 0 | 0 | 0 | 0 | 0 | 0 | 0 | 0 |
| cg11973981 | 3 | 404 | 1 | 918 | 0 | 0 | 0 | 0 | 0 | 0 | 0 | 0 |
| cg12134602 | 3 | 83 | 429 | 811 | 0 | 0 | 0 | 0 | 0 | 0 | 0 | 0 |
| cg12169700 | 5 | 137 | 2 | 1 | 470 | 713 | 0 | 0 | 0 | 0 | 0 | 0 |
| cg12352225 | 4 | 36 | 2 | 318 | 967 | 0 | 0 | 0 | 0 | 0 | 0 | 0 |
| cg12469381 | 3 | 224 | 1 | 1098 | 0 | 0 | 0 | 0 | 0 | 0 | 0 | 0 |
| cg12543766 | 4 | 60 | 395 | 867 | 1 | 0 | 0 | 0 | 0 | 0 | 0 | 0 |
| cg12551908 | 3 | 154 | 3 | 1166 | 0 | 0 | 0 | 0 | 0 | 0 | 0 | 0 |
| cg12689021 | 4 | 121 | 2 | 1199 | 1 | 0 | 0 | 0 | 0 | 0 | 0 | 0 |
| cg12743416 | 3 | 787 | 427 | 109 | 0 | 0 | 0 | 0 | 0 | 0 | 0 | 0 |
| cg12784228 | 3 | 8 | 121 | 1194 | 0 | 0 | 0 | 0 | 0 | 0 | 0 | 0 |
| cg12917058 | 6 | 2 | 1 | 95 | 2 | 1 | 1222 | 0 | 0 | 0 | 0 | 0 |
| cg12952561 | 5 | 44 | 1 | 2 | 363 | 913 | 0 | 0 | 0 | 0 | 0 | 0 |
| cg13031029 | 6 | 133 | 1 | 1 | 2 | 497 | 689 | 0 | 0 | 0 | 0 | 0 |
| cg13047462 | 4 | 1217 | 103 | 1 | 2 | 0 | 0 | 0 | 0 | 0 | 0 | 0 |
| cg13211008 | 4 | 479 | 1 | 502 | 341 | 0 | 0 | 0 | 0 | 0 | 0 | 0 |
| cg13295089 | 5 | 84 | 3 | 373 | 3 | 860 | 0 | 0 | 0 | 0 | 0 | 0 |
| cg13322072 | 4 | 21 | 1 | 240 | 1061 | 0 | 0 | 0 | 0 | 0 | 0 | 0 |
| cg13523718 | 3 | 1019 | 269 | 35 | 0 | 0 | 0 | 0 | 0 | 0 | 0 | 0 |
| cg13569207 | 3 | 299 | 658 | 366 | 0 | 0 | 0 | 0 | 0 | 0 | 0 | 0 |
| cg13585334 | 5 | 25 | 1 | 1 | 127 | 1169 | 0 | 0 | 0 | 0 | 0 | 0 |
| cg13589109 | 4 | 28 | 1 | 259 | 1035 | 0 | 0 | 0 | 0 | 0 | 0 | 0 |
| cg13748354 | 2 | 195 | 1128 | 0 | 0 | 0 | 0 | 0 | 0 | 0 | 0 | 0 |
| cg13752708 | 3 | 3 | 90 | 1230 | 0 | 0 | 0 | 0 | 0 | 0 | 0 | 0 |
| cg13824456 | 3 | 123 | 1 | 1199 | 0 | 0 | 0 | 0 | 0 | 0 | 0 | 0 |
| cg13979581 | 3 | 34 | 276 | 1013 | 0 | 0 | 0 | 0 | 0 | 0 | 0 | 0 |
| cg14170201 | 5 | 2 | 1 | 67 | 2 | 1251 | 0 | 0 | 0 | 0 | 0 | 0 |
| cg14615128 | 3 | 192 | 2 | 1129 | 0 | 0 | 0 | 0 | 0 | 0 | 0 | 0 |
| cg14651435 | 3 | 182 | 538 | 603 | 0 | 0 | 0 | 0 | 0 | 0 | 0 | 0 |
| cg14928378 | 3 | 151 | 3 | 1169 | 0 | 0 | 0 | 0 | 0 | 0 | 0 | 0 |
| cg15391794 | 3 | 81 | 1237 | 5 | 0 | 0 | 0 | 0 | 0 | 0 | 0 | 0 |
| cg15561493 | 3 | 94 | 2 | 1227 | 0 | 0 | 0 | 0 | 0 | 0 | 0 | 0 |
| cg15567368 | 5 | 205 | 1 | 1 | 570 | 546 | 0 | 0 | 0 | 0 | 0 | 0 |
| cg15765638 | 3 | 65 | 366 | 892 | 0 | 0 | 0 | 0 | 0 | 0 | 0 | 0 |
| cg15768613 | 3 | 47 | 47 | 1229 | 0 | 0 | 0 | 0 | 0 | 0 | 0 | 0 |
| cg15800276 | 4 | 392 | 646 | 1 | 284 | 0 | 0 | 0 | 0 | 0 | 0 | 0 |
| cg16101574 | 2 | 1256 | 67 | 0 | 0 | 0 | 0 | 0 | 0 | 0 | 0 | 0 |
| cg16476432 | 3 | 7 | 153 | 1163 | 0 | 0 | 0 | 0 | 0 | 0 | 0 | 0 |
| cg16576544 | 5 | 7 | 1 | 126 | 1 | 1188 | 0 | 0 | 0 | 0 | 0 | 0 |
| cg16634737 | 5 | 303 | 1 | 1 | 628 | 390 | 0 | 0 | 0 | 0 | 0 | 0 |
| cg16792234 | 5 | 317 | 1 | 2 | 654 | 349 | 0 | 0 | 0 | 0 | 0 | 0 |
| cg16813187 | 2 | 68 | 1255 | 0 | 0 | 0 | 0 | 0 | 0 | 0 | 0 | 0 |
| cg16906555 | 2 | 161 | 1162 | 0 | 0 | 0 | 0 | 0 | 0 | 0 | 0 | 0 |
| cg16963093 | 4 | 160 | 3 | 582 | 578 | 0 | 0 | 0 | 0 | 0 | 0 | 0 |
| cg16964206 | 4 | 7 | 169 | 3 | 1144 | 0 | 0 | 0 | 0 | 0 | 0 | 0 |
| cg17196155 | 4 | 192 | 2 | 544 | 585 | 0 | 0 | 0 | 0 | 0 | 0 | 0 |
| cg17220237 | 5 | 81 | 1 | 1 | 475 | 765 | 0 | 0 | 0 | 0 | 0 | 0 |
| cg17329602 | 2 | 187 | 1136 | 0 | 0 | 0 | 0 | 0 | 0 | 0 | 0 | 0 |
| cg17348244 | 5 | 726 | 4 | 2 | 1 | 590 | 0 | 0 | 0 | 0 | 0 | 0 |
| cg17393140 | 7 | 78 | 1 | 1 | 1 | 402 | 1 | 839 | 0 | 0 | 0 | 0 |
| cg17466637 | 5 | 2 | 1 | 2 | 86 | 1232 | 0 | 0 | 0 | 0 | 0 | 0 |
| cg17626372 | 3 | 1 | 73 | 1249 | 0 | 0 | 0 | 0 | 0 | 0 | 0 | 0 |
| cg17802552 | 3 | 9 | 90 | 1224 | 0 | 0 | 0 | 0 | 0 | 0 | 0 | 0 |
| cg17803235 | 3 | 15 | 115 | 1193 | 0 | 0 | 0 | 0 | 0 | 0 | 0 | 0 |
| cg18088486 | 4 | 163 | 1 | 521 | 638 | 0 | 0 | 0 | 0 | 0 | 0 | 0 |
| cg18500967 | 3 | 421 | 3 | 899 | 0 | 0 | 0 | 0 | 0 | 0 | 0 | 0 |
| cg18669823 | 4 | 143 | 326 | 2 | 852 | 0 | 0 | 0 | 0 | 0 | 0 | 0 |
| cg18673341 | 5 | 426 | 2 | 1 | 598 | 296 | 0 | 0 | 0 | 0 | 0 | 0 |
| cg18883682 | 5 | 22 | 1 | 1 | 288 | 1011 | 0 | 0 | 0 | 0 | 0 | 0 |
| cg19106179 | 4 | 27 | 150 | 1 | 1145 | 0 | 0 | 0 | 0 | 0 | 0 | 0 |
| cg19119382 | 4 | 5 | 1 | 110 | 1207 | 0 | 0 | 0 | 0 | 0 | 0 | 0 |
| cg19214707 | 3 | 423 | 899 | 1 | 0 | 0 | 0 | 0 | 0 | 0 | 0 | 0 |
| cg19248407 | 3 | 185 | 2 | 1136 | 0 | 0 | 0 | 0 | 0 | 0 | 0 | 0 |
| cg19389973 | 3 | 126 | 1 | 1196 | 0 | 0 | 0 | 0 | 0 | 0 | 0 | 0 |
| cg19591135 | 3 | 4 | 63 | 1256 | 0 | 0 | 0 | 0 | 0 | 0 | 0 | 0 |
| cg19799454 | 4 | 1 | 200 | 6 | 1116 | 0 | 0 | 0 | 0 | 0 | 0 | 0 |
| cg20026367 | 2 | 87 | 1236 | 0 | 0 | 0 | 0 | 0 | 0 | 0 | 0 | 0 |
| cg20200629 | 3 | 13 | 95 | 1215 | 0 | 0 | 0 | 0 | 0 | 0 | 0 | 0 |
| cg20426275 | 3 | 12 | 190 | 1121 | 0 | 0 | 0 | 0 | 0 | 0 | 0 | 0 |
| cg20608215 | 3 | 5 | 101 | 1217 | 0 | 0 | 0 | 0 | 0 | 0 | 0 | 0 |
| cg20913114 | 2 | 80 | 1243 | 0 | 0 | 0 | 0 | 0 | 0 | 0 | 0 | 0 |
| cg20934445 | 4 | 10 | 115 | 1 | 1197 | 0 | 0 | 0 | 0 | 0 | 0 | 0 |
| cg20959002 | 3 | 17 | 179 | 1127 | 0 | 0 | 0 | 0 | 0 | 0 | 0 | 0 |
| cg21200382 | 4 | 1225 | 1 | 91 | 6 | 0 | 0 | 0 | 0 | 0 | 0 | 0 |
| cg21598190 | 4 | 34 | 1 | 277 | 1011 | 0 | 0 | 0 | 0 | 0 | 0 | 0 |
| cg21655171 | 3 | 8 | 177 | 1138 | 0 | 0 | 0 | 0 | 0 | 0 | 0 | 0 |
| cg21685655 | 2 | 75 | 1248 | 0 | 0 | 0 | 0 | 0 | 0 | 0 | 0 | 0 |
| cg21823080 | 4 | 76 | 2 | 1244 | 1 | 0 | 0 | 0 | 0 | 0 | 0 | 0 |
| cg22535849 | 3 | 211 | 429 | 683 | 0 | 0 | 0 | 0 | 0 | 0 | 0 | 0 |
| cg22804805 | 2 | 90 | 1233 | 0 | 0 | 0 | 0 | 0 | 0 | 0 | 0 | 0 |
| cg22953237 | 2 | 176 | 1147 | 0 | 0 | 0 | 0 | 0 | 0 | 0 | 0 | 0 |
| cg23024343 | 3 | 68 | 4 | 1251 | 0 | 0 | 0 | 0 | 0 | 0 | 0 | 0 |
| cg23099265 | 4 | 14 | 1 | 110 | 1198 | 0 | 0 | 0 | 0 | 0 | 0 | 0 |
| cg23162598 | 4 | 241 | 2 | 588 | 492 | 0 | 0 | 0 | 0 | 0 | 0 | 0 |
| cg23496178 | 5 | 63 | 2 | 1 | 1256 | 1 | 0 | 0 | 0 | 0 | 0 | 0 |
| cg23694597 | 5 | 10 | 1 | 1 | 223 | 1088 | 0 | 0 | 0 | 0 | 0 | 0 |
| cg23727079 | 4 | 79 | 1 | 1241 | 2 | 0 | 0 | 0 | 0 | 0 | 0 | 0 |
| cg23876203 | 4 | 18 | 1 | 212 | 1092 | 0 | 0 | 0 | 0 | 0 | 0 | 0 |
| cg23891049 | 3 | 3 | 95 | 1225 | 0 | 0 | 0 | 0 | 0 | 0 | 0 | 0 |
| cg24022528 | 5 | 10 | 1 | 242 | 3 | 1067 | 0 | 0 | 0 | 0 | 0 | 0 |
| cg24208588 | 4 | 996 | 1 | 270 | 56 | 0 | 0 | 0 | 0 | 0 | 0 | 0 |
| cg24398793 | 3 | 1 | 579 | 743 | 0 | 0 | 0 | 0 | 0 | 0 | 0 | 0 |
| cg24648384 | 3 | 83 | 447 | 793 | 0 | 0 | 0 | 0 | 0 | 0 | 0 | 0 |
| cg24764114 | 4 | 3 | 2 | 125 | 1193 | 0 | 0 | 0 | 0 | 0 | 0 | 0 |
| cg24927769 | 3 | 68 | 1 | 1254 | 0 | 0 | 0 | 0 | 0 | 0 | 0 | 0 |
| cg25037777 | 3 | 47 | 277 | 999 | 0 | 0 | 0 | 0 | 0 | 0 | 0 | 0 |
| cg25361454 | 6 | 151 | 1 | 1 | 3 | 1 | 1166 | 0 | 0 | 0 | 0 | 0 |
| cg25543264 | 3 | 207 | 5 | 1111 | 0 | 0 | 0 | 0 | 0 | 0 | 0 | 0 |
| cg25577212 | 4 | 2 | 66 | 1254 | 1 | 0 | 0 | 0 | 0 | 0 | 0 | 0 |
| cg25709790 | 3 | 150 | 511 | 662 | 0 | 0 | 0 | 0 | 0 | 0 | 0 | 0 |
| cg25977769 | 6 | 291 | 1 | 1 | 608 | 420 | 2 | 0 | 0 | 0 | 0 | 0 |
| cg26220323 | 3 | 2 | 105 | 1216 | 0 | 0 | 0 | 0 | 0 | 0 | 0 | 0 |
| cg26445985 | 2 | 430 | 893 | 0 | 0 | 0 | 0 | 0 | 0 | 0 | 0 | 0 |
| cg26542892 | 4 | 57 | 415 | 3 | 848 | 0 | 0 | 0 | 0 | 0 | 0 | 0 |
| cg26837768 | 4 | 2 | 1 | 69 | 1251 | 0 | 0 | 0 | 0 | 0 | 0 | 0 |
| cg26856631 | 4 | 793 | 4 | 435 | 91 | 0 | 0 | 0 | 0 | 0 | 0 | 0 |
| cg26859469 | 3 | 14 | 151 | 1158 | 0 | 0 | 0 | 0 | 0 | 0 | 0 | 0 |
| cg26968039 | 2 | 130 | 1193 | 0 | 0 | 0 | 0 | 0 | 0 | 0 | 0 | 0 |
| cg27135799 | 3 | 1 | 78 | 1244 | 0 | 0 | 0 | 0 | 0 | 0 | 0 | 0 |
| cg27481428 | 5 | 111 | 3 | 474 | 1 | 734 | 0 | 0 | 0 | 0 | 0 | 0 |
| cg00033213 | 4 | 406 | 1 | 589 | 327 | 0 | 0 | 0 | 0 | 0 | 0 | 0 |
| cg00295418 | 2 | 151 | 1172 | 0 | 0 | 0 | 0 | 0 | 0 | 0 | 0 | 0 |
| cg00695177 | 5 | 281 | 1 | 2 | 474 | 565 | 0 | 0 | 0 | 0 | 0 | 0 |
| cg01470456 | 5 | 2 | 1 | 84 | 3 | 1233 | 0 | 0 | 0 | 0 | 0 | 0 |
| cg01921484 | 3 | 15 | 228 | 1080 | 0 | 0 | 0 | 0 | 0 | 0 | 0 | 0 |
| cg01958928 | 2 | 67 | 1256 | 0 | 0 | 0 | 0 | 0 | 0 | 0 | 0 | 0 |
| cg02151632 | 3 | 4 | 122 | 1197 | 0 | 0 | 0 | 0 | 0 | 0 | 0 | 0 |
| cg02179473 | 2 | 179 | 1144 | 0 | 0 | 0 | 0 | 0 | 0 | 0 | 0 | 0 |
| cg02299007 | 4 | 920 | 1 | 2 | 400 | 0 | 0 | 0 | 0 | 0 | 0 | 0 |
| cg02612228 | 3 | 2 | 70 | 1251 | 0 | 0 | 0 | 0 | 0 | 0 | 0 | 0 |
| cg02658043 | 5 | 228 | 2 | 1 | 379 | 713 | 0 | 0 | 0 | 0 | 0 | 0 |
| cg02841798 | 5 | 4 | 2 | 1 | 79 | 1237 | 0 | 0 | 0 | 0 | 0 | 0 |
| cg02901522 | 4 | 126 | 4 | 502 | 691 | 0 | 0 | 0 | 0 | 0 | 0 | 0 |
| cg03066009 | 4 | 79 | 1 | 1242 | 1 | 0 | 0 | 0 | 0 | 0 | 0 | 0 |
| cg03067774 | 3 | 22 | 258 | 1043 | 0 | 0 | 0 | 0 | 0 | 0 | 0 | 0 |
| cg03143591 | 4 | 24 | 42 | 5 | 1252 | 0 | 0 | 0 | 0 | 0 | 0 | 0 |
| cg03183700 | 2 | 80 | 1243 | 0 | 0 | 0 | 0 | 0 | 0 | 0 | 0 | 0 |
| cg03978067 | 4 | 8 | 1 | 163 | 1151 | 0 | 0 | 0 | 0 | 0 | 0 | 0 |
| cg04028540 | 4 | 85 | 399 | 2 | 837 | 0 | 0 | 0 | 0 | 0 | 0 | 0 |
| cg04123498 | 3 | 94 | 468 | 761 | 0 | 0 | 0 | 0 | 0 | 0 | 0 | 0 |
| cg04178011 | 4 | 120 | 1 | 2 | 1200 | 0 | 0 | 0 | 0 | 0 | 0 | 0 |
| cg04450797 | 5 | 230 | 3 | 1 | 634 | 455 | 0 | 0 | 0 | 0 | 0 | 0 |
| cg04613734 | 3 | 138 | 1 | 1184 | 0 | 0 | 0 | 0 | 0 | 0 | 0 | 0 |
| cg04620750 | 3 | 4 | 66 | 1253 | 0 | 0 | 0 | 0 | 0 | 0 | 0 | 0 |
| cg05130642 | 4 | 156 | 1 | 1 | 1165 | 0 | 0 | 0 | 0 | 0 | 0 | 0 |
| cg05342816 | 3 | 74 | 2 | 1247 | 0 | 0 | 0 | 0 | 0 | 0 | 0 | 0 |
| cg05463326 | 3 | 3 | 115 | 1205 | 0 | 0 | 0 | 0 | 0 | 0 | 0 | 0 |
| cg05502360 | 4 | 18 | 102 | 1202 | 1 | 0 | 0 | 0 | 0 | 0 | 0 | 0 |
| cg05890457 | 5 | 168 | 1 | 565 | 1 | 588 | 0 | 0 | 0 | 0 | 0 | 0 |
| cg05956943 | 4 | 7 | 139 | 1 | 1176 | 0 | 0 | 0 | 0 | 0 | 0 | 0 |
| cg06035200 | 3 | 19 | 260 | 1044 | 0 | 0 | 0 | 0 | 0 | 0 | 0 | 0 |
| cg06548479 | 5 | 864 | 403 | 2 | 53 | 1 | 0 | 0 | 0 | 0 | 0 | 0 |
| cg06703844 | 3 | 21 | 157 | 1145 | 0 | 0 | 0 | 0 | 0 | 0 | 0 | 0 |
| cg07518837 | 4 | 1255 | 2 | 62 | 4 | 0 | 0 | 0 | 0 | 0 | 0 | 0 |
| cg07777042 | 3 | 105 | 1217 | 1 | 0 | 0 | 0 | 0 | 0 | 0 | 0 | 0 |
| cg08225549 | 3 | 170 | 2 | 1151 | 0 | 0 | 0 | 0 | 0 | 0 | 0 | 0 |
| cg08270148 | 3 | 152 | 2 | 1169 | 0 | 0 | 0 | 0 | 0 | 0 | 0 | 0 |
| cg08542066 | 3 | 41 | 302 | 980 | 0 | 0 | 0 | 0 | 0 | 0 | 0 | 0 |
| cg08603678 | 3 | 355 | 634 | 334 | 0 | 0 | 0 | 0 | 0 | 0 | 0 | 0 |
| cg08775595 | 5 | 187 | 2 | 1 | 540 | 593 | 0 | 0 | 0 | 0 | 0 | 0 |
| cg09019154 | 3 | 1240 | 2 | 81 | 0 | 0 | 0 | 0 | 0 | 0 | 0 | 0 |
| cg09174817 | 3 | 6 | 147 | 1170 | 0 | 0 | 0 | 0 | 0 | 0 | 0 | 0 |
| cg09422696 | 3 | 31 | 300 | 992 | 0 | 0 | 0 | 0 | 0 | 0 | 0 | 0 |
| cg09533869 | 3 | 594 | 559 | 170 | 0 | 0 | 0 | 0 | 0 | 0 | 0 | 0 |
| cg10410513 | 3 | 10 | 194 | 1119 | 0 | 0 | 0 | 0 | 0 | 0 | 0 | 0 |
| cg10695549 | 5 | 360 | 2 | 666 | 1 | 294 | 0 | 0 | 0 | 0 | 0 | 0 |
| cg10712578 | 5 | 128 | 3 | 1 | 518 | 673 | 0 | 0 | 0 | 0 | 0 | 0 |
| cg10960626 | 4 | 1 | 100 | 1 | 1221 | 0 | 0 | 0 | 0 | 0 | 0 | 0 |
| cg11164659 | 3 | 63 | 396 | 864 | 0 | 0 | 0 | 0 | 0 | 0 | 0 | 0 |
| cg11420142 | 4 | 210 | 1 | 569 | 543 | 0 | 0 | 0 | 0 | 0 | 0 | 0 |
| cg11586249 | 4 | 7 | 1 | 100 | 1215 | 0 | 0 | 0 | 0 | 0 | 0 | 0 |
| cg11651932 | 5 | 58 | 2 | 315 | 1 | 947 | 0 | 0 | 0 | 0 | 0 | 0 |
| cg12175087 | 4 | 4 | 67 | 2 | 1250 | 0 | 0 | 0 | 0 | 0 | 0 | 0 |
| cg12293347 | 4 | 29 | 2 | 378 | 914 | 0 | 0 | 0 | 0 | 0 | 0 | 0 |
| cg12744694 | 4 | 63 | 3 | 302 | 955 | 0 | 0 | 0 | 0 | 0 | 0 | 0 |
| cg13180375 | 3 | 1244 | 77 | 2 | 0 | 0 | 0 | 0 | 0 | 0 | 0 | 0 |
| cg13289413 | 4 | 11 | 237 | 2 | 1073 | 0 | 0 | 0 | 0 | 0 | 0 | 0 |
| cg13453244 | 3 | 26 | 154 | 1143 | 0 | 0 | 0 | 0 | 0 | 0 | 0 | 0 |
| cg14044167 | 4 | 53 | 355 | 1 | 914 | 0 | 0 | 0 | 0 | 0 | 0 | 0 |
| cg14476852 | 3 | 4 | 71 | 1248 | 0 | 0 | 0 | 0 | 0 | 0 | 0 | 0 |
| cg14485877 | 4 | 4 | 104 | 5 | 1210 | 0 | 0 | 0 | 0 | 0 | 0 | 0 |
| cg15794987 | 6 | 473 | 6 | 1 | 1 | 3 | 839 | 0 | 0 | 0 | 0 | 0 |
| cg16191297 | 3 | 50 | 425 | 848 | 0 | 0 | 0 | 0 | 0 | 0 | 0 | 0 |
| cg16312212 | 6 | 1 | 146 | 1 | 2 | 1 | 1172 | 0 | 0 | 0 | 0 | 0 |
| cg16464506 | 5 | 6 | 1 | 2 | 62 | 1252 | 0 | 0 | 0 | 0 | 0 | 0 |
| cg16576930 | 3 | 178 | 3 | 1142 | 0 | 0 | 0 | 0 | 0 | 0 | 0 | 0 |
| cg16748433 | 3 | 248 | 633 | 442 | 0 | 0 | 0 | 0 | 0 | 0 | 0 | 0 |
| cg16814680 | 2 | 404 | 919 | 0 | 0 | 0 | 0 | 0 | 0 | 0 | 0 | 0 |
| cg16880779 | 3 | 23 | 156 | 1144 | 0 | 0 | 0 | 0 | 0 | 0 | 0 | 0 |
| cg16970850 | 6 | 2 | 1 | 4 | 1 | 122 | 1193 | 0 | 0 | 0 | 0 | 0 |
| cg17531142 | 4 | 8 | 1 | 158 | 1156 | 0 | 0 | 0 | 0 | 0 | 0 | 0 |
| cg17635970 | 5 | 64 | 2 | 1 | 473 | 783 | 0 | 0 | 0 | 0 | 0 | 0 |
| cg17975000 | 4 | 56 | 3 | 365 | 899 | 0 | 0 | 0 | 0 | 0 | 0 | 0 |
| cg18032891 | 4 | 1203 | 113 | 1 | 6 | 0 | 0 | 0 | 0 | 0 | 0 | 0 |
| cg18118834 | 3 | 75 | 1 | 1247 | 0 | 0 | 0 | 0 | 0 | 0 | 0 | 0 |
| cg19128026 | 2 | 322 | 1001 | 0 | 0 | 0 | 0 | 0 | 0 | 0 | 0 | 0 |
| cg20100002 | 4 | 1 | 70 | 1 | 1251 | 0 | 0 | 0 | 0 | 0 | 0 | 0 |
| cg20791291 | 4 | 18 | 267 | 3 | 1035 | 0 | 0 | 0 | 0 | 0 | 0 | 0 |
| cg20859841 | 4 | 42 | 1 | 329 | 951 | 0 | 0 | 0 | 0 | 0 | 0 | 0 |
| cg21028319 | 3 | 199 | 1 | 1123 | 0 | 0 | 0 | 0 | 0 | 0 | 0 | 0 |
| cg21035907 | 6 | 1 | 153 | 1 | 1 | 4 | 1163 | 0 | 0 | 0 | 0 | 0 |
| cg21160551 | 3 | 5 | 68 | 1250 | 0 | 0 | 0 | 0 | 0 | 0 | 0 | 0 |
| cg21297996 | 4 | 12 | 1 | 171 | 1139 | 0 | 0 | 0 | 0 | 0 | 0 | 0 |
| cg21498547 | 4 | 641 | 1 | 532 | 149 | 0 | 0 | 0 | 0 | 0 | 0 | 0 |
| cg21927991 | 5 | 113 | 1 | 538 | 1 | 670 | 0 | 0 | 0 | 0 | 0 | 0 |
| cg22029879 | 4 | 11 | 223 | 1 | 1088 | 0 | 0 | 0 | 0 | 0 | 0 | 0 |
| cg22661129 | 4 | 5 | 1 | 71 | 1246 | 0 | 0 | 0 | 0 | 0 | 0 | 0 |
| cg22861029 | 4 | 4 | 1 | 88 | 1230 | 0 | 0 | 0 | 0 | 0 | 0 | 0 |
| cg23283667 | 3 | 16 | 208 | 1099 | 0 | 0 | 0 | 0 | 0 | 0 | 0 | 0 |
| cg24849373 | 3 | 103 | 484 | 736 | 0 | 0 | 0 | 0 | 0 | 0 | 0 | 0 |
| cg25012649 | 3 | 6 | 73 | 1244 | 0 | 0 | 0 | 0 | 0 | 0 | 0 | 0 |
| cg25174338 | 4 | 49 | 408 | 2 | 864 | 0 | 0 | 0 | 0 | 0 | 0 | 0 |
| cg25953952 | 4 | 24 | 108 | 36 | 1155 | 0 | 0 | 0 | 0 | 0 | 0 | 0 |
| cg26038514 | 4 | 219 | 2 | 1 | 1101 | 0 | 0 | 0 | 0 | 0 | 0 | 0 |
| cg26077133 | 4 | 356 | 1 | 668 | 298 | 0 | 0 | 0 | 0 | 0 | 0 | 0 |
| cg26281303 | 5 | 7 | 1 | 4 | 163 | 1148 | 0 | 0 | 0 | 0 | 0 | 0 |
| cg26702098 | 4 | 37 | 269 | 1 | 1016 | 0 | 0 | 0 | 0 | 0 | 0 | 0 |
| cg26903052 | 2 | 89 | 1234 | 0 | 0 | 0 | 0 | 0 | 0 | 0 | 0 | 0 |
| cg26999501 | 3 | 69 | 1 | 1253 | 0 | 0 | 0 | 0 | 0 | 0 | 0 | 0 |
| cg27024127 | 2 | 847 | 476 | 0 | 0 | 0 | 0 | 0 | 0 | 0 | 0 | 0 |
| cg27450744 | 5 | 41 | 395 | 1 | 884 | 2 | 0 | 0 | 0 | 0 | 0 | 0 |
| cg27467876 | 4 | 280 | 595 | 1 | 447 | 0 | 0 | 0 | 0 | 0 | 0 | 0 |
| cg27538206 | 3 | 13 | 156 | 1154 | 0 | 0 | 0 | 0 | 0 | 0 | 0 | 0 |
| cg27628370 | 3 | 3 | 68 | 1252 | 0 | 0 | 0 | 0 | 0 | 0 | 0 | 0 |
| cg00102615 | 3 | 7 | 121 | 1195 | 0 | 0 | 0 | 0 | 0 | 0 | 0 | 0 |
| cg00540295 | 3 | 421 | 4 | 898 | 0 | 0 | 0 | 0 | 0 | 0 | 0 | 0 |
| cg01127608 | 5 | 804 | 1 | 436 | 1 | 81 | 0 | 0 | 0 | 0 | 0 | 0 |
| cg01474677 | 4 | 9 | 1 | 129 | 1184 | 0 | 0 | 0 | 0 | 0 | 0 | 0 |
| cg02014396 | 4 | 16 | 168 | 1 | 1138 | 0 | 0 | 0 | 0 | 0 | 0 | 0 |
| cg02945674 | 3 | 59 | 377 | 887 | 0 | 0 | 0 | 0 | 0 | 0 | 0 | 0 |
| cg03018489 | 3 | 15 | 233 | 1075 | 0 | 0 | 0 | 0 | 0 | 0 | 0 | 0 |
| cg03108651 | 2 | 131 | 1192 | 0 | 0 | 0 | 0 | 0 | 0 | 0 | 0 | 0 |
| cg03466780 | 3 | 140 | 533 | 650 | 0 | 0 | 0 | 0 | 0 | 0 | 0 | 0 |
| cg03531853 | 3 | 1223 | 97 | 3 | 0 | 0 | 0 | 0 | 0 | 0 | 0 | 0 |
| cg04563766 | 3 | 82 | 1 | 1240 | 0 | 0 | 0 | 0 | 0 | 0 | 0 | 0 |
| cg07056794 | 5 | 71 | 1 | 367 | 2 | 882 | 0 | 0 | 0 | 0 | 0 | 0 |
| cg08549829 | 4 | 25 | 1 | 209 | 1088 | 0 | 0 | 0 | 0 | 0 | 0 | 0 |
| cg09307883 | 3 | 34 | 263 | 1026 | 0 | 0 | 0 | 0 | 0 | 0 | 0 | 0 |
| cg10975354 | 4 | 1083 | 1 | 228 | 11 | 0 | 0 | 0 | 0 | 0 | 0 | 0 |
| cg12657416 | 4 | 499 | 591 | 1 | 232 | 0 | 0 | 0 | 0 | 0 | 0 | 0 |
| cg13396134 | 5 | 8 | 78 | 1 | 1 | 1235 | 0 | 0 | 0 | 0 | 0 | 0 |
| cg13431688 | 5 | 113 | 5 | 2 | 524 | 679 | 0 | 0 | 0 | 0 | 0 | 0 |
| cg13452812 | 4 | 132 | 1 | 439 | 751 | 0 | 0 | 0 | 0 | 0 | 0 | 0 |
| cg13511888 | 2 | 68 | 1255 | 0 | 0 | 0 | 0 | 0 | 0 | 0 | 0 | 0 |
| cg13522370 | 2 | 488 | 835 | 0 | 0 | 0 | 0 | 0 | 0 | 0 | 0 | 0 |
| cg13532421 | 6 | 1 | 71 | 2 | 2 | 1 | 1246 | 0 | 0 | 0 | 0 | 0 |
| cg13564529 | 5 | 155 | 1 | 511 | 2 | 654 | 0 | 0 | 0 | 0 | 0 | 0 |
| cg13633881 | 3 | 5 | 112 | 1206 | 0 | 0 | 0 | 0 | 0 | 0 | 0 | 0 |
| cg13641645 | 3 | 86 | 1233 | 4 | 0 | 0 | 0 | 0 | 0 | 0 | 0 | 0 |
| cg13651207 | 2 | 113 | 1210 | 0 | 0 | 0 | 0 | 0 | 0 | 0 | 0 | 0 |
| cg13683939 | 5 | 64 | 412 | 1 | 2 | 844 | 0 | 0 | 0 | 0 | 0 | 0 |
| cg13725087 | 2 | 140 | 1183 | 0 | 0 | 0 | 0 | 0 | 0 | 0 | 0 | 0 |
| cg13996562 | 5 | 10 | 1 | 146 | 1 | 1165 | 0 | 0 | 0 | 0 | 0 | 0 |
| cg14007688 | 6 | 133 | 1 | 1 | 547 | 2 | 639 | 0 | 0 | 0 | 0 | 0 |
| cg14021373 | 4 | 8 | 150 | 1 | 1164 | 0 | 0 | 0 | 0 | 0 | 0 | 0 |
| cg14056849 | 4 | 348 | 639 | 334 | 2 | 0 | 0 | 0 | 0 | 0 | 0 | 0 |
| cg14065526 | 2 | 82 | 1241 | 0 | 0 | 0 | 0 | 0 | 0 | 0 | 0 | 0 |
| cg14068184 | 3 | 147 | 4 | 1172 | 0 | 0 | 0 | 0 | 0 | 0 | 0 | 0 |
| cg14114910 | 6 | 136 | 1 | 2 | 430 | 2 | 752 | 0 | 0 | 0 | 0 | 0 |
| cg14179288 | 5 | 405 | 3 | 3 | 533 | 379 | 0 | 0 | 0 | 0 | 0 | 0 |
| cg14230280 | 3 | 6 | 133 | 1184 | 0 | 0 | 0 | 0 | 0 | 0 | 0 | 0 |
| cg14294971 | 3 | 17 | 90 | 1216 | 0 | 0 | 0 | 0 | 0 | 0 | 0 | 0 |
| cg14341177 | 3 | 102 | 43 | 1178 | 0 | 0 | 0 | 0 | 0 | 0 | 0 | 0 |
| cg14361804 | 4 | 490 | 1 | 831 | 1 | 0 | 0 | 0 | 0 | 0 | 0 | 0 |
| cg14369518 | 5 | 2 | 6 | 58 | 5 | 1252 | 0 | 0 | 0 | 0 | 0 | 0 |
| cg14417873 | 5 | 1 | 75 | 5 | 1 | 1241 | 0 | 0 | 0 | 0 | 0 | 0 |
| cg14451627 | 4 | 5 | 1 | 114 | 1203 | 0 | 0 | 0 | 0 | 0 | 0 | 0 |
| cg14460215 | 3 | 178 | 1 | 1144 | 0 | 0 | 0 | 0 | 0 | 0 | 0 | 0 |
| cg14511393 | 3 | 19 | 160 | 1144 | 0 | 0 | 0 | 0 | 0 | 0 | 0 | 0 |
| cg14638919 | 4 | 3 | 100 | 1 | 1219 | 0 | 0 | 0 | 0 | 0 | 0 | 0 |
| cg14655569 | 5 | 211 | 1 | 456 | 2 | 653 | 0 | 0 | 0 | 0 | 0 | 0 |
| cg19297688 | 2 | 73 | 1250 | 0 | 0 | 0 | 0 | 0 | 0 | 0 | 0 | 0 |
| cg21177183 | 3 | 15 | 219 | 1089 | 0 | 0 | 0 | 0 | 0 | 0 | 0 | 0 |
| cg21203249 | 4 | 152 | 1 | 535 | 635 | 0 | 0 | 0 | 0 | 0 | 0 | 0 |
| cg21219268 | 4 | 18 | 197 | 2 | 1106 | 0 | 0 | 0 | 0 | 0 | 0 | 0 |
| cg21242448 | 4 | 335 | 600 | 2 | 386 | 0 | 0 | 0 | 0 | 0 | 0 | 0 |
| cg21243064 | 6 | 23 | 1 | 3 | 285 | 2 | 1009 | 0 | 0 | 0 | 0 | 0 |
| cg26981881 | 4 | 1 | 1 | 86 | 1235 | 0 | 0 | 0 | 0 | 0 | 0 | 0 |
| cg00231519 | 4 | 108 | 1 | 504 | 710 | 0 | 0 | 0 | 0 | 0 | 0 | 0 |
| cg00696044 | 3 | 317 | 4 | 1002 | 0 | 0 | 0 | 0 | 0 | 0 | 0 | 0 |
| cg01006048 | 4 | 95 | 1 | 312 | 915 | 0 | 0 | 0 | 0 | 0 | 0 | 0 |
| cg01128042 | 6 | 54 | 1 | 1 | 374 | 2 | 891 | 0 | 0 | 0 | 0 | 0 |
| cg01191806 | 4 | 43 | 389 | 1 | 890 | 0 | 0 | 0 | 0 | 0 | 0 | 0 |
| cg01512466 | 4 | 9 | 1 | 169 | 1144 | 0 | 0 | 0 | 0 | 0 | 0 | 0 |
| cg01516887 | 3 | 19 | 208 | 1096 | 0 | 0 | 0 | 0 | 0 | 0 | 0 | 0 |
| cg02113055 | 6 | 221 | 1 | 1 | 596 | 1 | 503 | 0 | 0 | 0 | 0 | 0 |
| cg02140517 | 3 | 7 | 185 | 1131 | 0 | 0 | 0 | 0 | 0 | 0 | 0 | 0 |
| cg02188665 | 2 | 209 | 1114 | 0 | 0 | 0 | 0 | 0 | 0 | 0 | 0 | 0 |
| cg02201753 | 5 | 108 | 1 | 1 | 1 | 1212 | 0 | 0 | 0 | 0 | 0 | 0 |
| cg02271943 | 2 | 215 | 1108 | 0 | 0 | 0 | 0 | 0 | 0 | 0 | 0 | 0 |
| cg02372404 | 4 | 657 | 424 | 1 | 241 | 0 | 0 | 0 | 0 | 0 | 0 | 0 |
| cg02401524 | 4 | 1 | 75 | 1 | 1246 | 0 | 0 | 0 | 0 | 0 | 0 | 0 |
| cg02533724 | 3 | 195 | 1 | 1127 | 0 | 0 | 0 | 0 | 0 | 0 | 0 | 0 |
| cg03293837 | 3 | 10 | 148 | 1165 | 0 | 0 | 0 | 0 | 0 | 0 | 0 | 0 |
| cg03643559 | 2 | 84 | 1239 | 0 | 0 | 0 | 0 | 0 | 0 | 0 | 0 | 0 |
| cg03751055 | 4 | 1 | 1 | 76 | 1245 | 0 | 0 | 0 | 0 | 0 | 0 | 0 |
| cg03816081 | 4 | 641 | 1 | 679 | 2 | 0 | 0 | 0 | 0 | 0 | 0 | 0 |
| cg03958058 | 3 | 45 | 369 | 909 | 0 | 0 | 0 | 0 | 0 | 0 | 0 | 0 |
| cg04034998 | 4 | 13 | 1 | 164 | 1145 | 0 | 0 | 0 | 0 | 0 | 0 | 0 |
| cg04194432 | 5 | 686 | 1 | 2 | 490 | 144 | 0 | 0 | 0 | 0 | 0 | 0 |
| cg04683516 | 2 | 493 | 830 | 0 | 0 | 0 | 0 | 0 | 0 | 0 | 0 | 0 |
| cg04686117 | 3 | 26 | 151 | 1146 | 0 | 0 | 0 | 0 | 0 | 0 | 0 | 0 |
| cg04852694 | 5 | 1 | 5 | 1 | 72 | 1244 | 0 | 0 | 0 | 0 | 0 | 0 |
| cg04944931 | 3 | 13 | 201 | 1109 | 0 | 0 | 0 | 0 | 0 | 0 | 0 | 0 |
| cg05096415 | 3 | 232 | 1 | 1090 | 0 | 0 | 0 | 0 | 0 | 0 | 0 | 0 |
| cg05273049 | 3 | 1017 | 247 | 59 | 0 | 0 | 0 | 0 | 0 | 0 | 0 | 0 |
| cg05601623 | 5 | 970 | 323 | 1 | 2 | 27 | 0 | 0 | 0 | 0 | 0 | 0 |
| cg05818501 | 2 | 74 | 1249 | 0 | 0 | 0 | 0 | 0 | 0 | 0 | 0 | 0 |
| cg05919621 | 4 | 4 | 3 | 96 | 1220 | 0 | 0 | 0 | 0 | 0 | 0 | 0 |
| cg06051619 | 4 | 973 | 307 | 2 | 41 | 0 | 0 | 0 | 0 | 0 | 0 | 0 |
| cg06076610 | 3 | 3 | 88 | 1232 | 0 | 0 | 0 | 0 | 0 | 0 | 0 | 0 |
| cg06115576 | 5 | 4 | 77 | 1 | 1 | 1240 | 0 | 0 | 0 | 0 | 0 | 0 |
| cg06702884 | 4 | 6 | 74 | 1 | 1242 | 0 | 0 | 0 | 0 | 0 | 0 | 0 |
| cg06749524 | 3 | 173 | 1 | 1149 | 0 | 0 | 0 | 0 | 0 | 0 | 0 | 0 |
| cg06971224 | 6 | 404 | 3 | 2 | 3 | 622 | 289 | 0 | 0 | 0 | 0 | 0 |
| cg06995503 | 3 | 62 | 398 | 863 | 0 | 0 | 0 | 0 | 0 | 0 | 0 | 0 |
| cg07240846 | 3 | 164 | 537 | 622 | 0 | 0 | 0 | 0 | 0 | 0 | 0 | 0 |
| cg07413656 | 2 | 150 | 1173 | 0 | 0 | 0 | 0 | 0 | 0 | 0 | 0 | 0 |
| cg07414422 | 5 | 6 | 109 | 1 | 1 | 1206 | 0 | 0 | 0 | 0 | 0 | 0 |
| cg07498088 | 5 | 138 | 1 | 411 | 1 | 772 | 0 | 0 | 0 | 0 | 0 | 0 |
| cg07506153 | 4 | 51 | 2 | 419 | 851 | 0 | 0 | 0 | 0 | 0 | 0 | 0 |
| cg07559420 | 3 | 3 | 65 | 1255 | 0 | 0 | 0 | 0 | 0 | 0 | 0 | 0 |
| cg07844442 | 3 | 481 | 3 | 839 | 0 | 0 | 0 | 0 | 0 | 0 | 0 | 0 |
| cg08265181 | 4 | 136 | 4 | 2 | 1181 | 0 | 0 | 0 | 0 | 0 | 0 | 0 |
| cg09349613 | 4 | 14 | 2 | 159 | 1148 | 0 | 0 | 0 | 0 | 0 | 0 | 0 |
| cg09387681 | 3 | 14 | 178 | 1131 | 0 | 0 | 0 | 0 | 0 | 0 | 0 | 0 |
| cg09399869 | 4 | 17 | 130 | 2 | 1174 | 0 | 0 | 0 | 0 | 0 | 0 | 0 |
| cg09480417 | 3 | 199 | 1 | 1123 | 0 | 0 | 0 | 0 | 0 | 0 | 0 | 0 |
| cg09504384 | 4 | 24 | 300 | 1 | 998 | 0 | 0 | 0 | 0 | 0 | 0 | 0 |
| cg09667067 | 3 | 10 | 172 | 1141 | 0 | 0 | 0 | 0 | 0 | 0 | 0 | 0 |
| cg09854620 | 3 | 68 | 1 | 1254 | 0 | 0 | 0 | 0 | 0 | 0 | 0 | 0 |
| cg09867002 | 4 | 102 | 1 | 1 | 1219 | 0 | 0 | 0 | 0 | 0 | 0 | 0 |
| cg09931872 | 4 | 5 | 1 | 99 | 1218 | 0 | 0 | 0 | 0 | 0 | 0 | 0 |
| cg09993319 | 2 | 410 | 913 | 0 | 0 | 0 | 0 | 0 | 0 | 0 | 0 | 0 |
| cg10058204 | 3 | 277 | 636 | 410 | 0 | 0 | 0 | 0 | 0 | 0 | 0 | 0 |
| cg10100585 | 2 | 108 | 1215 | 0 | 0 | 0 | 0 | 0 | 0 | 0 | 0 | 0 |
| cg10102162 | 5 | 1 | 62 | 1 | 3 | 1256 | 0 | 0 | 0 | 0 | 0 | 0 |
| cg10309386 | 2 | 394 | 929 | 0 | 0 | 0 | 0 | 0 | 0 | 0 | 0 | 0 |
| cg10507965 | 3 | 622 | 528 | 173 | 0 | 0 | 0 | 0 | 0 | 0 | 0 | 0 |
| cg10786572 | 3 | 175 | 1145 | 3 | 0 | 0 | 0 | 0 | 0 | 0 | 0 | 0 |
| cg10920758 | 4 | 544 | 605 | 1 | 173 | 0 | 0 | 0 | 0 | 0 | 0 | 0 |
| cg10954330 | 6 | 24 | 2 | 314 | 2 | 1 | 980 | 0 | 0 | 0 | 0 | 0 |
| cg10989317 | 4 | 1 | 1 | 798 | 523 | 0 | 0 | 0 | 0 | 0 | 0 | 0 |
| cg11123613 | 4 | 1 | 68 | 4 | 1250 | 0 | 0 | 0 | 0 | 0 | 0 | 0 |
| cg11454325 | 5 | 111 | 1 | 1 | 1 | 1209 | 0 | 0 | 0 | 0 | 0 | 0 |
| cg11477175 | 3 | 17 | 122 | 1184 | 0 | 0 | 0 | 0 | 0 | 0 | 0 | 0 |
| cg11480019 | 3 | 1132 | 160 | 31 | 0 | 0 | 0 | 0 | 0 | 0 | 0 | 0 |
| cg11715594 | 5 | 8 | 2 | 1 | 85 | 1227 | 0 | 0 | 0 | 0 | 0 | 0 |
| cg11752430 | 4 | 8 | 1 | 96 | 1218 | 0 | 0 | 0 | 0 | 0 | 0 | 0 |
| cg11818589 | 4 | 444 | 1 | 620 | 258 | 0 | 0 | 0 | 0 | 0 | 0 | 0 |
| cg12226682 | 3 | 20 | 173 | 1130 | 0 | 0 | 0 | 0 | 0 | 0 | 0 | 0 |
| cg12421087 | 4 | 48 | 1 | 320 | 954 | 0 | 0 | 0 | 0 | 0 | 0 | 0 |
| cg12501287 | 4 | 105 | 2 | 444 | 772 | 0 | 0 | 0 | 0 | 0 | 0 | 0 |
| cg13181019 | 3 | 5 | 77 | 1241 | 0 | 0 | 0 | 0 | 0 | 0 | 0 | 0 |
| cg13379757 | 5 | 139 | 1 | 242 | 1 | 940 | 0 | 0 | 0 | 0 | 0 | 0 |
| cg13488137 | 2 | 89 | 1234 | 0 | 0 | 0 | 0 | 0 | 0 | 0 | 0 | 0 |
| cg13530320 | 3 | 12 | 254 | 1057 | 0 | 0 | 0 | 0 | 0 | 0 | 0 | 0 |
| cg13655986 | 4 | 36 | 280 | 2 | 1005 | 0 | 0 | 0 | 0 | 0 | 0 | 0 |
| cg13854498 | 3 | 9 | 207 | 1107 | 0 | 0 | 0 | 0 | 0 | 0 | 0 | 0 |
| cg14144366 | 4 | 49 | 1 | 362 | 911 | 0 | 0 | 0 | 0 | 0 | 0 | 0 |
| cg14293674 | 4 | 55 | 1 | 366 | 901 | 0 | 0 | 0 | 0 | 0 | 0 | 0 |
| cg14464133 | 2 | 116 | 1207 | 0 | 0 | 0 | 0 | 0 | 0 | 0 | 0 | 0 |
| cg14877834 | 3 | 202 | 1 | 1120 | 0 | 0 | 0 | 0 | 0 | 0 | 0 | 0 |
| cg14964115 | 2 | 1228 | 95 | 0 | 0 | 0 | 0 | 0 | 0 | 0 | 0 | 0 |
| cg15083522 | 3 | 61 | 400 | 862 | 0 | 0 | 0 | 0 | 0 | 0 | 0 | 0 |
| cg15633912 | 3 | 231 | 5 | 1087 | 0 | 0 | 0 | 0 | 0 | 0 | 0 | 0 |
| cg15988569 | 4 | 316 | 4 | 2 | 1001 | 0 | 0 | 0 | 0 | 0 | 0 | 0 |
| cg16397968 | 2 | 83 | 1240 | 0 | 0 | 0 | 0 | 0 | 0 | 0 | 0 | 0 |
| cg16432908 | 5 | 52 | 1 | 454 | 1 | 815 | 0 | 0 | 0 | 0 | 0 | 0 |
| cg16645815 | 5 | 40 | 1 | 290 | 3 | 989 | 0 | 0 | 0 | 0 | 0 | 0 |
| cg17646571 | 3 | 40 | 185 | 1098 | 0 | 0 | 0 | 0 | 0 | 0 | 0 | 0 |
| cg17686260 | 6 | 47 | 1 | 2 | 308 | 2 | 963 | 0 | 0 | 0 | 0 | 0 |
| cg17723206 | 3 | 323 | 569 | 431 | 0 | 0 | 0 | 0 | 0 | 0 | 0 | 0 |
| cg18001722 | 4 | 24 | 1 | 295 | 1003 | 0 | 0 | 0 | 0 | 0 | 0 | 0 |
| cg18135502 | 3 | 4 | 116 | 1203 | 0 | 0 | 0 | 0 | 0 | 0 | 0 | 0 |
| cg18860280 | 3 | 12 | 143 | 1168 | 0 | 0 | 0 | 0 | 0 | 0 | 0 | 0 |
| cg18861767 | 3 | 304 | 1 | 1018 | 0 | 0 | 0 | 0 | 0 | 0 | 0 | 0 |
| cg19263847 | 3 | 5 | 92 | 1226 | 0 | 0 | 0 | 0 | 0 | 0 | 0 | 0 |
| cg19301501 | 3 | 5 | 119 | 1199 | 0 | 0 | 0 | 0 | 0 | 0 | 0 | 0 |
| cg19360212 | 4 | 28 | 300 | 1 | 994 | 0 | 0 | 0 | 0 | 0 | 0 | 0 |
| cg19377607 | 3 | 392 | 1 | 930 | 0 | 0 | 0 | 0 | 0 | 0 | 0 | 0 |
| cg19415746 | 4 | 125 | 1 | 1 | 1196 | 0 | 0 | 0 | 0 | 0 | 0 | 0 |
| cg19492498 | 2 | 127 | 1196 | 0 | 0 | 0 | 0 | 0 | 0 | 0 | 0 | 0 |
| cg19848924 | 5 | 121 | 1 | 323 | 1 | 877 | 0 | 0 | 0 | 0 | 0 | 0 |
| cg20205188 | 3 | 96 | 474 | 753 | 0 | 0 | 0 | 0 | 0 | 0 | 0 | 0 |
| cg20245361 | 4 | 2 | 73 | 1 | 1247 | 0 | 0 | 0 | 0 | 0 | 0 | 0 |
| cg20313873 | 3 | 1232 | 87 | 4 | 0 | 0 | 0 | 0 | 0 | 0 | 0 | 0 |
| cg20653859 | 2 | 116 | 1207 | 0 | 0 | 0 | 0 | 0 | 0 | 0 | 0 | 0 |
| cg20657903 | 2 | 68 | 1255 | 0 | 0 | 0 | 0 | 0 | 0 | 0 | 0 | 0 |
| cg20722327 | 5 | 4 | 3 | 76 | 1238 | 2 | 0 | 0 | 0 | 0 | 0 | 0 |
| cg21587006 | 4 | 157 | 1 | 577 | 588 | 0 | 0 | 0 | 0 | 0 | 0 | 0 |
| cg22645355 | 6 | 23 | 2 | 2 | 251 | 4 | 1041 | 0 | 0 | 0 | 0 | 0 |
| cg22710156 | 3 | 70 | 1 | 1252 | 0 | 0 | 0 | 0 | 0 | 0 | 0 | 0 |
| cg22836207 | 3 | 109 | 3 | 1211 | 0 | 0 | 0 | 0 | 0 | 0 | 0 | 0 |
| cg23052585 | 5 | 326 | 5 | 581 | 3 | 408 | 0 | 0 | 0 | 0 | 0 | 0 |
| cg23098789 | 4 | 30 | 1 | 325 | 967 | 0 | 0 | 0 | 0 | 0 | 0 | 0 |
| cg23126094 | 4 | 8 | 1 | 92 | 1222 | 0 | 0 | 0 | 0 | 0 | 0 | 0 |
| cg23128967 | 3 | 15 | 151 | 1157 | 0 | 0 | 0 | 0 | 0 | 0 | 0 | 0 |
| cg23175573 | 6 | 77 | 3 | 1 | 1 | 474 | 767 | 0 | 0 | 0 | 0 | 0 |
| cg23187802 | 4 | 95 | 518 | 709 | 1 | 0 | 0 | 0 | 0 | 0 | 0 | 0 |
| cg23291158 | 3 | 12 | 143 | 1168 | 0 | 0 | 0 | 0 | 0 | 0 | 0 | 0 |
| cg23492225 | 3 | 15 | 236 | 1072 | 0 | 0 | 0 | 0 | 0 | 0 | 0 | 0 |
| cg23513018 | 2 | 404 | 919 | 0 | 0 | 0 | 0 | 0 | 0 | 0 | 0 | 0 |
| cg23657215 | 3 | 36 | 289 | 998 | 0 | 0 | 0 | 0 | 0 | 0 | 0 | 0 |
| cg23693569 | 4 | 53 | 235 | 2 | 1033 | 0 | 0 | 0 | 0 | 0 | 0 | 0 |
| cg23698271 | 3 | 104 | 509 | 710 | 0 | 0 | 0 | 0 | 0 | 0 | 0 | 0 |
| cg24051234 | 3 | 10 | 151 | 1162 | 0 | 0 | 0 | 0 | 0 | 0 | 0 | 0 |
| cg24323552 | 2 | 83 | 1240 | 0 | 0 | 0 | 0 | 0 | 0 | 0 | 0 | 0 |
| cg24682073 | 4 | 3 | 70 | 1 | 1249 | 0 | 0 | 0 | 0 | 0 | 0 | 0 |
| cg24697097 | 2 | 123 | 1200 | 0 | 0 | 0 | 0 | 0 | 0 | 0 | 0 | 0 |
| cg24968869 | 3 | 18 | 218 | 1087 | 0 | 0 | 0 | 0 | 0 | 0 | 0 | 0 |
| cg25013753 | 5 | 376 | 2 | 3 | 604 | 338 | 0 | 0 | 0 | 0 | 0 | 0 |
| cg25014286 | 5 | 10 | 1 | 2 | 157 | 1153 | 0 | 0 | 0 | 0 | 0 | 0 |
| cg25542438 | 5 | 17 | 155 | 1 | 2 | 1148 | 0 | 0 | 0 | 0 | 0 | 0 |
| cg25601713 | 2 | 478 | 845 | 0 | 0 | 0 | 0 | 0 | 0 | 0 | 0 | 0 |
| cg25626166 | 3 | 8 | 116 | 1199 | 0 | 0 | 0 | 0 | 0 | 0 | 0 | 0 |
| cg25654326 | 2 | 184 | 1139 | 0 | 0 | 0 | 0 | 0 | 0 | 0 | 0 | 0 |
| cg25804357 | 2 | 104 | 1219 | 0 | 0 | 0 | 0 | 0 | 0 | 0 | 0 | 0 |
| cg26077897 | 6 | 6 | 1 | 1 | 1 | 77 | 1237 | 0 | 0 | 0 | 0 | 0 |
| cg26161528 | 3 | 2 | 84 | 1237 | 0 | 0 | 0 | 0 | 0 | 0 | 0 | 0 |
| cg26690318 | 2 | 197 | 1126 | 0 | 0 | 0 | 0 | 0 | 0 | 0 | 0 | 0 |
| cg27076160 | 2 | 119 | 1204 | 0 | 0 | 0 | 0 | 0 | 0 | 0 | 0 | 0 |
| cg27275103 | 4 | 13 | 1 | 174 | 1135 | 0 | 0 | 0 | 0 | 0 | 0 | 0 |
| cg27308738 | 3 | 254 | 615 | 454 | 0 | 0 | 0 | 0 | 0 | 0 | 0 | 0 |
| cg27346510 | 3 | 3 | 73 | 1247 | 0 | 0 | 0 | 0 | 0 | 0 | 0 | 0 |
| cg27455098 | 3 | 1 | 93 | 1229 | 0 | 0 | 0 | 0 | 0 | 0 | 0 | 0 |
| cg01192554 | 5 | 67 | 1 | 1 | 370 | 884 | 0 | 0 | 0 | 0 | 0 | 0 |
| cg01223071 | 2 | 306 | 1017 | 0 | 0 | 0 | 0 | 0 | 0 | 0 | 0 | 0 |
| cg01264919 | 4 | 25 | 183 | 1 | 1114 | 0 | 0 | 0 | 0 | 0 | 0 | 0 |
| cg01584086 | 3 | 596 | 726 | 1 | 0 | 0 | 0 | 0 | 0 | 0 | 0 | 0 |
| cg01835922 | 4 | 721 | 2 | 598 | 2 | 0 | 0 | 0 | 0 | 0 | 0 | 0 |
| cg02143936 | 5 | 21 | 9 | 189 | 1 | 1103 | 0 | 0 | 0 | 0 | 0 | 0 |
| cg02217616 | 4 | 2 | 82 | 1 | 1238 | 0 | 0 | 0 | 0 | 0 | 0 | 0 |
| cg02275226 | 3 | 83 | 2 | 1238 | 0 | 0 | 0 | 0 | 0 | 0 | 0 | 0 |
| cg02560739 | 3 | 101 | 2 | 1220 | 0 | 0 | 0 | 0 | 0 | 0 | 0 | 0 |
| cg02621636 | 4 | 3 | 72 | 1 | 1247 | 0 | 0 | 0 | 0 | 0 | 0 | 0 |
| cg02730303 | 4 | 837 | 4 | 387 | 95 | 0 | 0 | 0 | 0 | 0 | 0 | 0 |
| cg02814135 | 6 | 83 | 3 | 460 | 1 | 775 | 1 | 0 | 0 | 0 | 0 | 0 |
| cg03188793 | 3 | 11 | 168 | 1144 | 0 | 0 | 0 | 0 | 0 | 0 | 0 | 0 |
| cg03493520 | 3 | 277 | 1044 | 2 | 0 | 0 | 0 | 0 | 0 | 0 | 0 | 0 |
| cg03848831 | 4 | 5 | 82 | 2 | 1234 | 0 | 0 | 0 | 0 | 0 | 0 | 0 |
| cg03951394 | 4 | 23 | 1 | 248 | 1051 | 0 | 0 | 0 | 0 | 0 | 0 | 0 |
| cg04152793 | 3 | 188 | 1134 | 1 | 0 | 0 | 0 | 0 | 0 | 0 | 0 | 0 |
| cg04270414 | 4 | 19 | 1 | 237 | 1066 | 0 | 0 | 0 | 0 | 0 | 0 | 0 |
| cg04297660 | 4 | 31 | 1 | 337 | 954 | 0 | 0 | 0 | 0 | 0 | 0 | 0 |
| cg04467639 | 3 | 184 | 2 | 1137 | 0 | 0 | 0 | 0 | 0 | 0 | 0 | 0 |
| cg04878146 | 3 | 28 | 159 | 1136 | 0 | 0 | 0 | 0 | 0 | 0 | 0 | 0 |
| cg04934246 | 7 | 1 | 1 | 1 | 2 | 3 | 62 | 1253 | 0 | 0 | 0 | 0 |
| cg04998327 | 4 | 148 | 1 | 480 | 694 | 0 | 0 | 0 | 0 | 0 | 0 | 0 |
| cg05237503 | 5 | 163 | 1 | 1 | 601 | 557 | 0 | 0 | 0 | 0 | 0 | 0 |
| cg05276972 | 3 | 463 | 2 | 858 | 0 | 0 | 0 | 0 | 0 | 0 | 0 | 0 |
| cg05407200 | 2 | 271 | 1052 | 0 | 0 | 0 | 0 | 0 | 0 | 0 | 0 | 0 |
| cg05545891 | 5 | 1 | 5 | 1 | 94 | 1222 | 0 | 0 | 0 | 0 | 0 | 0 |
| cg05792312 | 4 | 71 | 2 | 441 | 809 | 0 | 0 | 0 | 0 | 0 | 0 | 0 |
| cg05960806 | 4 | 1238 | 83 | 1 | 1 | 0 | 0 | 0 | 0 | 0 | 0 | 0 |
| cg06120313 | 3 | 32 | 317 | 974 | 0 | 0 | 0 | 0 | 0 | 0 | 0 | 0 |
| cg06258939 | 3 | 36 | 301 | 986 | 0 | 0 | 0 | 0 | 0 | 0 | 0 | 0 |
| cg06394820 | 4 | 165 | 431 | 1 | 726 | 0 | 0 | 0 | 0 | 0 | 0 | 0 |
| cg06409538 | 2 | 1191 | 132 | 0 | 0 | 0 | 0 | 0 | 0 | 0 | 0 | 0 |
| cg06550274 | 3 | 101 | 1 | 1221 | 0 | 0 | 0 | 0 | 0 | 0 | 0 | 0 |
| cg06640773 | 4 | 64 | 2 | 1 | 1256 | 0 | 0 | 0 | 0 | 0 | 0 | 0 |
| cg06894070 | 5 | 1036 | 4 | 2 | 253 | 28 | 0 | 0 | 0 | 0 | 0 | 0 |
| cg07080031 | 4 | 5 | 132 | 1184 | 2 | 0 | 0 | 0 | 0 | 0 | 0 | 0 |
| cg07125829 | 4 | 10 | 176 | 1 | 1136 | 0 | 0 | 0 | 0 | 0 | 0 | 0 |
| cg07146321 | 4 | 7 | 142 | 1 | 1173 | 0 | 0 | 0 | 0 | 0 | 0 | 0 |
| cg07205452 | 6 | 13 | 1 | 119 | 1 | 1 | 1188 | 0 | 0 | 0 | 0 | 0 |
| cg07686394 | 3 | 170 | 2 | 1151 | 0 | 0 | 0 | 0 | 0 | 0 | 0 | 0 |
| cg08459368 | 3 | 3 | 70 | 1250 | 0 | 0 | 0 | 0 | 0 | 0 | 0 | 0 |
| cg08849813 | 2 | 139 | 1184 | 0 | 0 | 0 | 0 | 0 | 0 | 0 | 0 | 0 |
| cg09157251 | 3 | 671 | 1 | 651 | 0 | 0 | 0 | 0 | 0 | 0 | 0 | 0 |
| cg09233619 | 5 | 60 | 1 | 382 | 1 | 879 | 0 | 0 | 0 | 0 | 0 | 0 |
| cg09263513 | 4 | 56 | 1 | 273 | 993 | 0 | 0 | 0 | 0 | 0 | 0 | 0 |
| cg09428846 | 3 | 91 | 2 | 1230 | 0 | 0 | 0 | 0 | 0 | 0 | 0 | 0 |
| cg09520904 | 3 | 1169 | 146 | 8 | 0 | 0 | 0 | 0 | 0 | 0 | 0 | 0 |
| cg09663736 | 2 | 106 | 1217 | 0 | 0 | 0 | 0 | 0 | 0 | 0 | 0 | 0 |
| cg09737095 | 2 | 628 | 695 | 0 | 0 | 0 | 0 | 0 | 0 | 0 | 0 | 0 |
| cg09993718 | 3 | 399 | 5 | 919 | 0 | 0 | 0 | 0 | 0 | 0 | 0 | 0 |
| cg10098373 | 3 | 16 | 217 | 1090 | 0 | 0 | 0 | 0 | 0 | 0 | 0 | 0 |
| cg10306192 | 3 | 819 | 448 | 56 | 0 | 0 | 0 | 0 | 0 | 0 | 0 | 0 |
| cg10321395 | 3 | 56 | 308 | 959 | 0 | 0 | 0 | 0 | 0 | 0 | 0 | 0 |
| cg10415021 | 4 | 312 | 1 | 554 | 456 | 0 | 0 | 0 | 0 | 0 | 0 | 0 |
| cg10470368 | 4 | 15 | 1 | 192 | 1115 | 0 | 0 | 0 | 0 | 0 | 0 | 0 |
| cg10662047 | 2 | 205 | 1118 | 0 | 0 | 0 | 0 | 0 | 0 | 0 | 0 | 0 |
| cg10738003 | 2 | 194 | 1129 | 0 | 0 | 0 | 0 | 0 | 0 | 0 | 0 | 0 |
| cg11075353 | 3 | 147 | 460 | 716 | 0 | 0 | 0 | 0 | 0 | 0 | 0 | 0 |
| cg11143152 | 4 | 9 | 69 | 2 | 1243 | 0 | 0 | 0 | 0 | 0 | 0 | 0 |
| cg11475788 | 4 | 20 | 1 | 175 | 1127 | 0 | 0 | 0 | 0 | 0 | 0 | 0 |
| cg11720054 | 3 | 3 | 71 | 1249 | 0 | 0 | 0 | 0 | 0 | 0 | 0 | 0 |
| cg11820026 | 2 | 77 | 1246 | 0 | 0 | 0 | 0 | 0 | 0 | 0 | 0 | 0 |
| cg11936536 | 4 | 37 | 2 | 276 | 1008 | 0 | 0 | 0 | 0 | 0 | 0 | 0 |
| cg12062782 | 2 | 1253 | 70 | 0 | 0 | 0 | 0 | 0 | 0 | 0 | 0 | 0 |
| cg12186981 | 5 | 17 | 1 | 266 | 4 | 1035 | 0 | 0 | 0 | 0 | 0 | 0 |
| cg12208638 | 4 | 343 | 1 | 562 | 417 | 0 | 0 | 0 | 0 | 0 | 0 | 0 |
| cg12324426 | 5 | 2 | 1 | 63 | 2 | 1255 | 0 | 0 | 0 | 0 | 0 | 0 |
| cg12556569 | 5 | 567 | 2 | 569 | 1 | 184 | 0 | 0 | 0 | 0 | 0 | 0 |
| cg12568536 | 3 | 436 | 1 | 886 | 0 | 0 | 0 | 0 | 0 | 0 | 0 | 0 |
| cg13037187 | 4 | 1 | 86 | 1 | 1235 | 0 | 0 | 0 | 0 | 0 | 0 | 0 |
| cg13066461 | 2 | 125 | 1198 | 0 | 0 | 0 | 0 | 0 | 0 | 0 | 0 | 0 |
| cg13160852 | 5 | 451 | 1 | 1 | 869 | 1 | 0 | 0 | 0 | 0 | 0 | 0 |
| cg13275129 | 4 | 144 | 1 | 518 | 660 | 0 | 0 | 0 | 0 | 0 | 0 | 0 |
| cg13332350 | 3 | 101 | 513 | 709 | 0 | 0 | 0 | 0 | 0 | 0 | 0 | 0 |
| cg13361506 | 2 | 139 | 1184 | 0 | 0 | 0 | 0 | 0 | 0 | 0 | 0 | 0 |
| cg13518121 | 3 | 8 | 109 | 1206 | 0 | 0 | 0 | 0 | 0 | 0 | 0 | 0 |
| cg13916633 | 4 | 14 | 2 | 231 | 1076 | 0 | 0 | 0 | 0 | 0 | 0 | 0 |
| cg13996476 | 3 | 5 | 90 | 1228 | 0 | 0 | 0 | 0 | 0 | 0 | 0 | 0 |
| cg14089103 | 4 | 77 | 323 | 1 | 922 | 0 | 0 | 0 | 0 | 0 | 0 | 0 |
| cg14167033 | 5 | 42 | 1 | 326 | 1 | 953 | 0 | 0 | 0 | 0 | 0 | 0 |
| cg14897096 | 3 | 1 | 86 | 1236 | 0 | 0 | 0 | 0 | 0 | 0 | 0 | 0 |
| cg15268920 | 2 | 69 | 1254 | 0 | 0 | 0 | 0 | 0 | 0 | 0 | 0 | 0 |
| cg15392147 | 2 | 519 | 804 | 0 | 0 | 0 | 0 | 0 | 0 | 0 | 0 | 0 |
| cg15627180 | 4 | 96 | 3 | 1222 | 2 | 0 | 0 | 0 | 0 | 0 | 0 | 0 |
| cg15677681 | 4 | 30 | 296 | 1 | 996 | 0 | 0 | 0 | 0 | 0 | 0 | 0 |
| cg15828613 | 4 | 160 | 1 | 515 | 647 | 0 | 0 | 0 | 0 | 0 | 0 | 0 |
| cg16729283 | 6 | 6 | 1 | 2 | 1 | 75 | 1238 | 0 | 0 | 0 | 0 | 0 |
| cg16810310 | 3 | 41 | 367 | 915 | 0 | 0 | 0 | 0 | 0 | 0 | 0 | 0 |
| cg16954525 | 2 | 324 | 999 | 0 | 0 | 0 | 0 | 0 | 0 | 0 | 0 | 0 |
| cg16956665 | 3 | 239 | 1 | 1083 | 0 | 0 | 0 | 0 | 0 | 0 | 0 | 0 |
| cg16968851 | 4 | 4 | 77 | 1 | 1241 | 0 | 0 | 0 | 0 | 0 | 0 | 0 |
| cg17040924 | 4 | 333 | 1 | 620 | 369 | 0 | 0 | 0 | 0 | 0 | 0 | 0 |
| cg17044529 | 2 | 176 | 1147 | 0 | 0 | 0 | 0 | 0 | 0 | 0 | 0 | 0 |
| cg17095460 | 3 | 549 | 2 | 772 | 0 | 0 | 0 | 0 | 0 | 0 | 0 | 0 |
| cg17187785 | 4 | 27 | 1 | 305 | 990 | 0 | 0 | 0 | 0 | 0 | 0 | 0 |
| cg17271269 | 2 | 109 | 1214 | 0 | 0 | 0 | 0 | 0 | 0 | 0 | 0 | 0 |
| cg17953636 | 4 | 1244 | 2 | 71 | 6 | 0 | 0 | 0 | 0 | 0 | 0 | 0 |
| cg18422632 | 4 | 36 | 4 | 203 | 1080 | 0 | 0 | 0 | 0 | 0 | 0 | 0 |
| cg18493115 | 4 | 4 | 107 | 2 | 1210 | 0 | 0 | 0 | 0 | 0 | 0 | 0 |
| cg18828306 | 3 | 230 | 2 | 1091 | 0 | 0 | 0 | 0 | 0 | 0 | 0 | 0 |
| cg18875674 | 4 | 25 | 227 | 1 | 1070 | 0 | 0 | 0 | 0 | 0 | 0 | 0 |
| cg19380371 | 3 | 4 | 98 | 1221 | 0 | 0 | 0 | 0 | 0 | 0 | 0 | 0 |
| cg19471911 | 3 | 297 | 2 | 1024 | 0 | 0 | 0 | 0 | 0 | 0 | 0 | 0 |
| cg19750116 | 4 | 13 | 1 | 149 | 1160 | 0 | 0 | 0 | 0 | 0 | 0 | 0 |
| cg20090066 | 4 | 73 | 1 | 348 | 901 | 0 | 0 | 0 | 0 | 0 | 0 | 0 |
| cg20147224 | 3 | 1005 | 3 | 315 | 0 | 0 | 0 | 0 | 0 | 0 | 0 | 0 |
| cg20704148 | 2 | 243 | 1080 | 0 | 0 | 0 | 0 | 0 | 0 | 0 | 0 | 0 |
| cg20805368 | 3 | 9 | 150 | 1164 | 0 | 0 | 0 | 0 | 0 | 0 | 0 | 0 |
| cg21757617 | 3 | 126 | 1196 | 1 | 0 | 0 | 0 | 0 | 0 | 0 | 0 | 0 |
| cg22251955 | 5 | 210 | 1 | 600 | 511 | 1 | 0 | 0 | 0 | 0 | 0 | 0 |
| cg22280068 | 4 | 33 | 229 | 2 | 1059 | 0 | 0 | 0 | 0 | 0 | 0 | 0 |
| cg22355889 | 5 | 988 | 1 | 302 | 1 | 31 | 0 | 0 | 0 | 0 | 0 | 0 |
| cg22620689 | 4 | 8 | 1 | 93 | 1221 | 0 | 0 | 0 | 0 | 0 | 0 | 0 |
| cg22907103 | 4 | 4 | 1 | 72 | 1246 | 0 | 0 | 0 | 0 | 0 | 0 | 0 |
| cg23404737 | 6 | 9 | 2 | 2 | 1 | 127 | 1182 | 0 | 0 | 0 | 0 | 0 |
| cg23418965 | 2 | 91 | 1232 | 0 | 0 | 0 | 0 | 0 | 0 | 0 | 0 | 0 |
| cg24189340 | 2 | 116 | 1207 | 0 | 0 | 0 | 0 | 0 | 0 | 0 | 0 | 0 |
| cg24426391 | 4 | 256 | 598 | 468 | 1 | 0 | 0 | 0 | 0 | 0 | 0 | 0 |
| cg24643105 | 4 | 91 | 1 | 393 | 838 | 0 | 0 | 0 | 0 | 0 | 0 | 0 |
| cg24769381 | 3 | 130 | 1 | 1192 | 0 | 0 | 0 | 0 | 0 | 0 | 0 | 0 |
| cg24851651 | 3 | 248 | 498 | 577 | 0 | 0 | 0 | 0 | 0 | 0 | 0 | 0 |
| cg24872173 | 4 | 185 | 2 | 1 | 1135 | 0 | 0 | 0 | 0 | 0 | 0 | 0 |
| cg24993471 | 3 | 4 | 136 | 1183 | 0 | 0 | 0 | 0 | 0 | 0 | 0 | 0 |
| cg25174111 | 5 | 271 | 1 | 1 | 3 | 1047 | 0 | 0 | 0 | 0 | 0 | 0 |
| cg25203245 | 2 | 394 | 929 | 0 | 0 | 0 | 0 | 0 | 0 | 0 | 0 | 0 |
| cg25497175 | 3 | 66 | 2 | 1255 | 0 | 0 | 0 | 0 | 0 | 0 | 0 | 0 |
| cg25899729 | 3 | 7 | 86 | 1230 | 0 | 0 | 0 | 0 | 0 | 0 | 0 | 0 |
| cg25963939 | 3 | 44 | 345 | 934 | 0 | 0 | 0 | 0 | 0 | 0 | 0 | 0 |
| cg26069044 | 5 | 296 | 1 | 522 | 1 | 503 | 0 | 0 | 0 | 0 | 0 | 0 |
| cg26155681 | 2 | 881 | 442 | 0 | 0 | 0 | 0 | 0 | 0 | 0 | 0 | 0 |
| cg26338473 | 4 | 4 | 1 | 128 | 1190 | 0 | 0 | 0 | 0 | 0 | 0 | 0 |
| cg26387458 | 3 | 276 | 1046 | 1 | 0 | 0 | 0 | 0 | 0 | 0 | 0 | 0 |
| cg26450240 | 2 | 77 | 1246 | 0 | 0 | 0 | 0 | 0 | 0 | 0 | 0 | 0 |
| cg26465155 | 4 | 88 | 471 | 4 | 760 | 0 | 0 | 0 | 0 | 0 | 0 | 0 |
| cg26676440 | 4 | 4 | 1 | 125 | 1193 | 0 | 0 | 0 | 0 | 0 | 0 | 0 |
| cg26757229 | 3 | 105 | 2 | 1216 | 0 | 0 | 0 | 0 | 0 | 0 | 0 | 0 |
| cg26839410 | 4 | 7 | 1 | 156 | 1159 | 0 | 0 | 0 | 0 | 0 | 0 | 0 |
| cg26840043 | 3 | 115 | 1207 | 1 | 0 | 0 | 0 | 0 | 0 | 0 | 0 | 0 |
| cg26853071 | 4 | 60 | 1 | 396 | 866 | 0 | 0 | 0 | 0 | 0 | 0 | 0 |
| cg26864826 | 3 | 624 | 529 | 170 | 0 | 0 | 0 | 0 | 0 | 0 | 0 | 0 |
| cg26881268 | 4 | 4 | 4 | 90 | 1225 | 0 | 0 | 0 | 0 | 0 | 0 | 0 |
| cg27049594 | 4 | 261 | 2 | 635 | 425 | 0 | 0 | 0 | 0 | 0 | 0 | 0 |
| cg27079096 | 5 | 198 | 2 | 1 | 654 | 468 | 0 | 0 | 0 | 0 | 0 | 0 |
| cg27649396 | 3 | 93 | 3 | 1227 | 0 | 0 | 0 | 0 | 0 | 0 | 0 | 0 |
| cg00087746 | 2 | 125 | 1198 | 0 | 0 | 0 | 0 | 0 | 0 | 0 | 0 | 0 |
| cg00218628 | 4 | 72 | 1 | 390 | 860 | 0 | 0 | 0 | 0 | 0 | 0 | 0 |
| cg00267207 | 4 | 80 | 1 | 2 | 1240 | 0 | 0 | 0 | 0 | 0 | 0 | 0 |
| cg00474091 | 4 | 11 | 1 | 177 | 1134 | 0 | 0 | 0 | 0 | 0 | 0 | 0 |
| cg00476882 | 4 | 87 | 2 | 1232 | 2 | 0 | 0 | 0 | 0 | 0 | 0 | 0 |
| cg00631877 | 3 | 165 | 1 | 1157 | 0 | 0 | 0 | 0 | 0 | 0 | 0 | 0 |
| cg00693253 | 3 | 239 | 4 | 1080 | 0 | 0 | 0 | 0 | 0 | 0 | 0 | 0 |
| cg01012836 | 4 | 213 | 1 | 1108 | 1 | 0 | 0 | 0 | 0 | 0 | 0 | 0 |
| cg01120761 | 4 | 10 | 171 | 1 | 1141 | 0 | 0 | 0 | 0 | 0 | 0 | 0 |
| cg01201512 | 2 | 1063 | 260 | 0 | 0 | 0 | 0 | 0 | 0 | 0 | 0 | 0 |
| cg01613406 | 6 | 9 | 1 | 1 | 155 | 2 | 1155 | 0 | 0 | 0 | 0 | 0 |
| cg01639943 | 4 | 1 | 1 | 90 | 1231 | 0 | 0 | 0 | 0 | 0 | 0 | 0 |
| cg01869224 | 4 | 1 | 83 | 3 | 1236 | 0 | 0 | 0 | 0 | 0 | 0 | 0 |
| cg02418195 | 3 | 12 | 191 | 1120 | 0 | 0 | 0 | 0 | 0 | 0 | 0 | 0 |
| cg02516696 | 2 | 127 | 1196 | 0 | 0 | 0 | 0 | 0 | 0 | 0 | 0 | 0 |
| cg02571042 | 3 | 20 | 125 | 1178 | 0 | 0 | 0 | 0 | 0 | 0 | 0 | 0 |
| cg02668233 | 3 | 127 | 1 | 1195 | 0 | 0 | 0 | 0 | 0 | 0 | 0 | 0 |
| cg03802120 | 4 | 3 | 76 | 4 | 1240 | 0 | 0 | 0 | 0 | 0 | 0 | 0 |
| cg03870234 | 3 | 72 | 5 | 1246 | 0 | 0 | 0 | 0 | 0 | 0 | 0 | 0 |
| cg04105166 | 4 | 4 | 73 | 1 | 1245 | 0 | 0 | 0 | 0 | 0 | 0 | 0 |
| cg04464523 | 3 | 6 | 152 | 1165 | 0 | 0 | 0 | 0 | 0 | 0 | 0 | 0 |
| cg05134736 | 3 | 791 | 454 | 78 | 0 | 0 | 0 | 0 | 0 | 0 | 0 | 0 |
| cg05308244 | 2 | 320 | 1003 | 0 | 0 | 0 | 0 | 0 | 0 | 0 | 0 | 0 |
| cg05471616 | 2 | 111 | 1212 | 0 | 0 | 0 | 0 | 0 | 0 | 0 | 0 | 0 |
| cg05483487 | 2 | 77 | 1246 | 0 | 0 | 0 | 0 | 0 | 0 | 0 | 0 | 0 |
| cg05575399 | 3 | 1217 | 98 | 8 | 0 | 0 | 0 | 0 | 0 | 0 | 0 | 0 |
| cg05603011 | 5 | 2 | 1 | 2 | 72 | 1246 | 0 | 0 | 0 | 0 | 0 | 0 |
| cg05948955 | 5 | 14 | 1 | 161 | 1 | 1146 | 0 | 0 | 0 | 0 | 0 | 0 |
| cg05990366 | 4 | 28 | 314 | 2 | 979 | 0 | 0 | 0 | 0 | 0 | 0 | 0 |
| cg06352538 | 4 | 165 | 488 | 2 | 668 | 0 | 0 | 0 | 0 | 0 | 0 | 0 |
| cg06801483 | 4 | 8 | 1 | 88 | 1226 | 0 | 0 | 0 | 0 | 0 | 0 | 0 |
| cg07091544 | 3 | 5 | 82 | 1236 | 0 | 0 | 0 | 0 | 0 | 0 | 0 | 0 |
| cg07183970 | 3 | 92 | 3 | 1228 | 0 | 0 | 0 | 0 | 0 | 0 | 0 | 0 |
| cg07222420 | 3 | 1 | 70 | 1252 | 0 | 0 | 0 | 0 | 0 | 0 | 0 | 0 |
| cg07249488 | 5 | 111 | 2 | 1 | 2 | 1207 | 0 | 0 | 0 | 0 | 0 | 0 |
| cg07474670 | 4 | 184 | 586 | 1 | 552 | 0 | 0 | 0 | 0 | 0 | 0 | 0 |
| cg07636477 | 3 | 5 | 84 | 1234 | 0 | 0 | 0 | 0 | 0 | 0 | 0 | 0 |
| cg07741840 | 6 | 142 | 2 | 1 | 499 | 1 | 678 | 0 | 0 | 0 | 0 | 0 |
| cg08629394 | 3 | 258 | 3 | 1062 | 0 | 0 | 0 | 0 | 0 | 0 | 0 | 0 |
| cg09081941 | 3 | 18 | 119 | 1186 | 0 | 0 | 0 | 0 | 0 | 0 | 0 | 0 |
| cg09084244 | 4 | 428 | 1 | 480 | 414 | 0 | 0 | 0 | 0 | 0 | 0 | 0 |
| cg09284209 | 3 | 106 | 1 | 1216 | 0 | 0 | 0 | 0 | 0 | 0 | 0 | 0 |
| cg09339156 | 3 | 22 | 290 | 1011 | 0 | 0 | 0 | 0 | 0 | 0 | 0 | 0 |
| cg10738648 | 2 | 330 | 993 | 0 | 0 | 0 | 0 | 0 | 0 | 0 | 0 | 0 |
| cg10957001 | 4 | 28 | 1 | 224 | 1070 | 0 | 0 | 0 | 0 | 0 | 0 | 0 |
| cg11066365 | 3 | 77 | 1245 | 1 | 0 | 0 | 0 | 0 | 0 | 0 | 0 | 0 |
| cg11132120 | 3 | 135 | 4 | 1184 | 0 | 0 | 0 | 0 | 0 | 0 | 0 | 0 |
| cg11701583 | 3 | 1152 | 160 | 11 | 0 | 0 | 0 | 0 | 0 | 0 | 0 | 0 |
| cg11902380 | 4 | 5 | 103 | 1 | 1214 | 0 | 0 | 0 | 0 | 0 | 0 | 0 |
| cg12087615 | 3 | 228 | 1 | 1094 | 0 | 0 | 0 | 0 | 0 | 0 | 0 | 0 |
| cg12765935 | 4 | 10 | 190 | 2 | 1121 | 0 | 0 | 0 | 0 | 0 | 0 | 0 |
| cg12801863 | 3 | 17 | 148 | 1158 | 0 | 0 | 0 | 0 | 0 | 0 | 0 | 0 |
| cg13011003 | 5 | 25 | 1 | 1 | 267 | 1029 | 0 | 0 | 0 | 0 | 0 | 0 |
| cg13127920 | 2 | 330 | 993 | 0 | 0 | 0 | 0 | 0 | 0 | 0 | 0 | 0 |
| cg13380890 | 2 | 866 | 457 | 0 | 0 | 0 | 0 | 0 | 0 | 0 | 0 | 0 |
| cg13523561 | 5 | 5 | 1 | 75 | 1 | 1241 | 0 | 0 | 0 | 0 | 0 | 0 |
| cg13621101 | 4 | 4 | 86 | 1 | 1232 | 0 | 0 | 0 | 0 | 0 | 0 | 0 |
| cg13815695 | 5 | 129 | 3 | 536 | 2 | 653 | 0 | 0 | 0 | 0 | 0 | 0 |
| cg14240634 | 3 | 8 | 119 | 1196 | 0 | 0 | 0 | 0 | 0 | 0 | 0 | 0 |
| cg14739785 | 4 | 4 | 112 | 1 | 1206 | 0 | 0 | 0 | 0 | 0 | 0 | 0 |
| cg14912045 | 4 | 1 | 96 | 4 | 1222 | 0 | 0 | 0 | 0 | 0 | 0 | 0 |
| cg15007809 | 6 | 3 | 2 | 1 | 103 | 2 | 1212 | 0 | 0 | 0 | 0 | 0 |
| cg15068593 | 2 | 261 | 1062 | 0 | 0 | 0 | 0 | 0 | 0 | 0 | 0 | 0 |
| cg15411736 | 3 | 113 | 1 | 1209 | 0 | 0 | 0 | 0 | 0 | 0 | 0 | 0 |
| cg15775217 | 4 | 39 | 284 | 1 | 999 | 0 | 0 | 0 | 0 | 0 | 0 | 0 |
| cg16196728 | 3 | 1 | 66 | 1256 | 0 | 0 | 0 | 0 | 0 | 0 | 0 | 0 |
| cg16268937 | 5 | 224 | 1 | 1 | 1 | 1096 | 0 | 0 | 0 | 0 | 0 | 0 |
| cg16470760 | 3 | 2 | 66 | 1255 | 0 | 0 | 0 | 0 | 0 | 0 | 0 | 0 |
| cg17003970 | 3 | 3 | 93 | 1227 | 0 | 0 | 0 | 0 | 0 | 0 | 0 | 0 |
| cg17057702 | 3 | 1 | 83 | 1239 | 0 | 0 | 0 | 0 | 0 | 0 | 0 | 0 |
| cg17530337 | 3 | 137 | 416 | 770 | 0 | 0 | 0 | 0 | 0 | 0 | 0 | 0 |
| cg18443741 | 4 | 337 | 3 | 662 | 321 | 0 | 0 | 0 | 0 | 0 | 0 | 0 |
| cg18618674 | 4 | 2 | 89 | 4 | 1228 | 0 | 0 | 0 | 0 | 0 | 0 | 0 |
| cg18997918 | 4 | 83 | 315 | 1 | 924 | 0 | 0 | 0 | 0 | 0 | 0 | 0 |
| cg19235645 | 2 | 694 | 629 | 0 | 0 | 0 | 0 | 0 | 0 | 0 | 0 | 0 |
| cg19353052 | 4 | 45 | 3 | 281 | 994 | 0 | 0 | 0 | 0 | 0 | 0 | 0 |
| cg19393008 | 5 | 440 | 1 | 2 | 574 | 306 | 0 | 0 | 0 | 0 | 0 | 0 |
| cg19611616 | 4 | 1234 | 1 | 1 | 87 | 0 | 0 | 0 | 0 | 0 | 0 | 0 |
| cg19789919 | 4 | 1112 | 1 | 204 | 6 | 0 | 0 | 0 | 0 | 0 | 0 | 0 |
| cg19816075 | 2 | 83 | 1240 | 0 | 0 | 0 | 0 | 0 | 0 | 0 | 0 | 0 |
| cg20040891 | 3 | 41 | 417 | 865 | 0 | 0 | 0 | 0 | 0 | 0 | 0 | 0 |
| cg20089799 | 5 | 574 | 2 | 601 | 1 | 145 | 0 | 0 | 0 | 0 | 0 | 0 |
| cg20139683 | 2 | 415 | 908 | 0 | 0 | 0 | 0 | 0 | 0 | 0 | 0 | 0 |
| cg20503652 | 4 | 67 | 1 | 1 | 1254 | 0 | 0 | 0 | 0 | 0 | 0 | 0 |
| cg21046080 | 5 | 491 | 1 | 1 | 608 | 222 | 0 | 0 | 0 | 0 | 0 | 0 |
| cg21053147 | 3 | 5 | 67 | 1251 | 0 | 0 | 0 | 0 | 0 | 0 | 0 | 0 |
| cg21074476 | 4 | 9 | 107 | 2 | 1205 | 0 | 0 | 0 | 0 | 0 | 0 | 0 |
| cg21442528 | 3 | 45 | 292 | 986 | 0 | 0 | 0 | 0 | 0 | 0 | 0 | 0 |
| cg22026089 | 4 | 367 | 2 | 1 | 953 | 0 | 0 | 0 | 0 | 0 | 0 | 0 |
| cg22138998 | 5 | 117 | 1 | 1 | 468 | 736 | 0 | 0 | 0 | 0 | 0 | 0 |
| cg22543924 | 4 | 175 | 1 | 2 | 1145 | 0 | 0 | 0 | 0 | 0 | 0 | 0 |
| cg23075597 | 4 | 1 | 100 | 1 | 1221 | 0 | 0 | 0 | 0 | 0 | 0 | 0 |
| cg23159970 | 2 | 530 | 793 | 0 | 0 | 0 | 0 | 0 | 0 | 0 | 0 | 0 |
| cg23432430 | 4 | 23 | 264 | 1 | 1035 | 0 | 0 | 0 | 0 | 0 | 0 | 0 |
| cg23556238 | 3 | 46 | 150 | 1127 | 0 | 0 | 0 | 0 | 0 | 0 | 0 | 0 |
| cg23591463 | 5 | 1093 | 1 | 208 | 1 | 20 | 0 | 0 | 0 | 0 | 0 | 0 |
| cg23601664 | 3 | 48 | 373 | 902 | 0 | 0 | 0 | 0 | 0 | 0 | 0 | 0 |
| cg23764766 | 2 | 260 | 1063 | 0 | 0 | 0 | 0 | 0 | 0 | 0 | 0 | 0 |
| cg23836570 | 4 | 122 | 1 | 520 | 680 | 0 | 0 | 0 | 0 | 0 | 0 | 0 |
| cg23878260 | 3 | 70 | 366 | 887 | 0 | 0 | 0 | 0 | 0 | 0 | 0 | 0 |
| cg23914255 | 4 | 36 | 1 | 353 | 933 | 0 | 0 | 0 | 0 | 0 | 0 | 0 |
| cg24135151 | 5 | 19 | 1 | 2 | 164 | 1137 | 0 | 0 | 0 | 0 | 0 | 0 |
| cg24309769 | 3 | 96 | 513 | 714 | 0 | 0 | 0 | 0 | 0 | 0 | 0 | 0 |
| cg24730756 | 4 | 1127 | 3 | 1 | 192 | 0 | 0 | 0 | 0 | 0 | 0 | 0 |
| cg25124058 | 4 | 6 | 1 | 156 | 1160 | 0 | 0 | 0 | 0 | 0 | 0 | 0 |
| cg25134647 | 4 | 299 | 3 | 1 | 1020 | 0 | 0 | 0 | 0 | 0 | 0 | 0 |
| cg25247689 | 2 | 354 | 969 | 0 | 0 | 0 | 0 | 0 | 0 | 0 | 0 | 0 |
| cg25601709 | 3 | 1 | 216 | 1106 | 0 | 0 | 0 | 0 | 0 | 0 | 0 | 0 |
| cg25669110 | 5 | 6 | 1 | 1 | 92 | 1223 | 0 | 0 | 0 | 0 | 0 | 0 |
| cg25674027 | 4 | 340 | 1 | 552 | 430 | 0 | 0 | 0 | 0 | 0 | 0 | 0 |
| cg25713370 | 5 | 25 | 2 | 1 | 226 | 1069 | 0 | 0 | 0 | 0 | 0 | 0 |
| cg26076233 | 2 | 137 | 1186 | 0 | 0 | 0 | 0 | 0 | 0 | 0 | 0 | 0 |
| cg26311454 | 4 | 18 | 174 | 1 | 1130 | 0 | 0 | 0 | 0 | 0 | 0 | 0 |
| cg26843567 | 4 | 5 | 1 | 146 | 1171 | 0 | 0 | 0 | 0 | 0 | 0 | 0 |
| cg26864661 | 5 | 768 | 2 | 475 | 1 | 77 | 0 | 0 | 0 | 0 | 0 | 0 |
| cg26941787 | 4 | 226 | 2 | 611 | 484 | 0 | 0 | 0 | 0 | 0 | 0 | 0 |
| cg27114706 | 5 | 89 | 1 | 441 | 3 | 789 | 0 | 0 | 0 | 0 | 0 | 0 |
| cg27321466 | 4 | 102 | 1 | 1219 | 1 | 0 | 0 | 0 | 0 | 0 | 0 | 0 |
| cg27504269 | 6 | 4 | 1 | 1 | 1 | 62 | 1254 | 0 | 0 | 0 | 0 | 0 |
| cg00502469 | 4 | 20 | 1 | 269 | 1033 | 0 | 0 | 0 | 0 | 0 | 0 | 0 |
| cg00667948 | 5 | 11 | 1 | 183 | 3 | 1125 | 0 | 0 | 0 | 0 | 0 | 0 |
| cg00898013 | 2 | 79 | 1244 | 0 | 0 | 0 | 0 | 0 | 0 | 0 | 0 | 0 |
| cg00915663 | 3 | 62 | 1254 | 7 | 0 | 0 | 0 | 0 | 0 | 0 | 0 | 0 |
| cg01758122 | 4 | 20 | 1 | 268 | 1034 | 0 | 0 | 0 | 0 | 0 | 0 | 0 |
| cg03272499 | 4 | 1 | 96 | 1222 | 4 | 0 | 0 | 0 | 0 | 0 | 0 | 0 |
| cg03372334 | 5 | 95 | 1 | 3 | 477 | 747 | 0 | 0 | 0 | 0 | 0 | 0 |
| cg03531388 | 3 | 73 | 5 | 1245 | 0 | 0 | 0 | 0 | 0 | 0 | 0 | 0 |
| cg03651054 | 3 | 1 | 941 | 381 | 0 | 0 | 0 | 0 | 0 | 0 | 0 | 0 |
| cg04438098 | 3 | 9 | 203 | 1111 | 0 | 0 | 0 | 0 | 0 | 0 | 0 | 0 |
| cg04520693 | 5 | 597 | 1 | 1 | 558 | 166 | 0 | 0 | 0 | 0 | 0 | 0 |
| cg04566473 | 5 | 62 | 1 | 1 | 245 | 1014 | 0 | 0 | 0 | 0 | 0 | 0 |
| cg05059349 | 2 | 583 | 740 | 0 | 0 | 0 | 0 | 0 | 0 | 0 | 0 | 0 |
| cg05279330 | 5 | 91 | 3 | 1 | 421 | 807 | 0 | 0 | 0 | 0 | 0 | 0 |
| cg05639937 | 6 | 4 | 4 | 5 | 1 | 108 | 1201 | 0 | 0 | 0 | 0 | 0 |
| cg05730108 | 4 | 144 | 1 | 1 | 1177 | 0 | 0 | 0 | 0 | 0 | 0 | 0 |
| cg05918715 | 3 | 222 | 3 | 1098 | 0 | 0 | 0 | 0 | 0 | 0 | 0 | 0 |
| cg06955954 | 2 | 255 | 1068 | 0 | 0 | 0 | 0 | 0 | 0 | 0 | 0 | 0 |
| cg07271471 | 3 | 84 | 1 | 1238 | 0 | 0 | 0 | 0 | 0 | 0 | 0 | 0 |
| cg07618759 | 3 | 417 | 5 | 901 | 0 | 0 | 0 | 0 | 0 | 0 | 0 | 0 |
| cg07876831 | 3 | 74 | 374 | 875 | 0 | 0 | 0 | 0 | 0 | 0 | 0 | 0 |
| cg08600378 | 2 | 210 | 1113 | 0 | 0 | 0 | 0 | 0 | 0 | 0 | 0 | 0 |
| cg09196346 | 2 | 855 | 468 | 0 | 0 | 0 | 0 | 0 | 0 | 0 | 0 | 0 |
| cg09397997 | 3 | 68 | 3 | 1252 | 0 | 0 | 0 | 0 | 0 | 0 | 0 | 0 |
| cg09746326 | 3 | 112 | 471 | 740 | 0 | 0 | 0 | 0 | 0 | 0 | 0 | 0 |
| cg10919109 | 3 | 22 | 283 | 1018 | 0 | 0 | 0 | 0 | 0 | 0 | 0 | 0 |
| cg10993865 | 4 | 112 | 1 | 1 | 1209 | 0 | 0 | 0 | 0 | 0 | 0 | 0 |
| cg11315081 | 3 | 4 | 91 | 1228 | 0 | 0 | 0 | 0 | 0 | 0 | 0 | 0 |
| cg11438287 | 3 | 77 | 1 | 1245 | 0 | 0 | 0 | 0 | 0 | 0 | 0 | 0 |
| cg11723923 | 5 | 92 | 1 | 1 | 479 | 750 | 0 | 0 | 0 | 0 | 0 | 0 |
| cg11842073 | 4 | 47 | 4 | 334 | 938 | 0 | 0 | 0 | 0 | 0 | 0 | 0 |
| cg11935063 | 5 | 3 | 68 | 2 | 1 | 1249 | 0 | 0 | 0 | 0 | 0 | 0 |
| cg12195446 | 3 | 120 | 457 | 746 | 0 | 0 | 0 | 0 | 0 | 0 | 0 | 0 |
| cg12472473 | 4 | 2 | 72 | 2 | 1247 | 0 | 0 | 0 | 0 | 0 | 0 | 0 |
| cg12513911 | 4 | 1192 | 1 | 127 | 3 | 0 | 0 | 0 | 0 | 0 | 0 | 0 |
| cg13033971 | 4 | 104 | 2 | 1 | 1216 | 0 | 0 | 0 | 0 | 0 | 0 | 0 |
| cg13506281 | 5 | 131 | 1 | 489 | 3 | 699 | 0 | 0 | 0 | 0 | 0 | 0 |
| cg13730219 | 3 | 919 | 2 | 402 | 0 | 0 | 0 | 0 | 0 | 0 | 0 | 0 |
| cg15335418 | 4 | 1236 | 1 | 85 | 1 | 0 | 0 | 0 | 0 | 0 | 0 | 0 |
| cg15388766 | 3 | 139 | 1 | 1183 | 0 | 0 | 0 | 0 | 0 | 0 | 0 | 0 |
| cg16151959 | 3 | 37 | 226 | 1060 | 0 | 0 | 0 | 0 | 0 | 0 | 0 | 0 |
| cg16503053 | 4 | 10 | 182 | 1 | 1130 | 0 | 0 | 0 | 0 | 0 | 0 | 0 |
| cg17707870 | 5 | 947 | 1 | 3 | 316 | 56 | 0 | 0 | 0 | 0 | 0 | 0 |
| cg17738613 | 4 | 225 | 1 | 554 | 543 | 0 | 0 | 0 | 0 | 0 | 0 | 0 |
| cg18105134 | 2 | 78 | 1245 | 0 | 0 | 0 | 0 | 0 | 0 | 0 | 0 | 0 |
| cg18446441 | 4 | 37 | 2 | 362 | 922 | 0 | 0 | 0 | 0 | 0 | 0 | 0 |
| cg18756657 | 4 | 9 | 127 | 1 | 1186 | 0 | 0 | 0 | 0 | 0 | 0 | 0 |
| cg19086603 | 5 | 13 | 1 | 246 | 1 | 1062 | 0 | 0 | 0 | 0 | 0 | 0 |
| cg19105674 | 4 | 2 | 81 | 1 | 1239 | 0 | 0 | 0 | 0 | 0 | 0 | 0 |
| cg19156046 | 7 | 1 | 134 | 2 | 2 | 1 | 1177 | 6 | 0 | 0 | 0 | 0 |
| cg20536971 | 4 | 258 | 668 | 1 | 396 | 0 | 0 | 0 | 0 | 0 | 0 | 0 |
| cg20622565 | 4 | 71 | 2 | 250 | 1000 | 0 | 0 | 0 | 0 | 0 | 0 | 0 |
| cg21128953 | 4 | 2 | 64 | 1 | 1256 | 0 | 0 | 0 | 0 | 0 | 0 | 0 |
| cg21463262 | 4 | 233 | 2 | 606 | 482 | 0 | 0 | 0 | 0 | 0 | 0 | 0 |
| cg22274196 | 4 | 504 | 610 | 1 | 208 | 0 | 0 | 0 | 0 | 0 | 0 | 0 |
| cg22491001 | 4 | 8 | 4 | 98 | 1213 | 0 | 0 | 0 | 0 | 0 | 0 | 0 |
| cg22646054 | 3 | 26 | 163 | 1134 | 0 | 0 | 0 | 0 | 0 | 0 | 0 | 0 |
| cg22917346 | 4 | 1255 | 1 | 62 | 5 | 0 | 0 | 0 | 0 | 0 | 0 | 0 |
| cg23168520 | 4 | 38 | 1 | 234 | 1050 | 0 | 0 | 0 | 0 | 0 | 0 | 0 |
| cg23630179 | 4 | 111 | 1210 | 1 | 1 | 0 | 0 | 0 | 0 | 0 | 0 | 0 |
| cg23947138 | 3 | 14 | 165 | 1144 | 0 | 0 | 0 | 0 | 0 | 0 | 0 | 0 |
| cg24738115 | 4 | 4 | 2 | 82 | 1235 | 0 | 0 | 0 | 0 | 0 | 0 | 0 |
| cg24941469 | 3 | 351 | 971 | 1 | 0 | 0 | 0 | 0 | 0 | 0 | 0 | 0 |
| cg24967131 | 5 | 1 | 1 | 78 | 2 | 1241 | 0 | 0 | 0 | 0 | 0 | 0 |
| cg25425994 | 3 | 7 | 158 | 1158 | 0 | 0 | 0 | 0 | 0 | 0 | 0 | 0 |
| cg26705599 | 4 | 236 | 2 | 570 | 515 | 0 | 0 | 0 | 0 | 0 | 0 | 0 |
| cg26773954 | 4 | 13 | 1 | 266 | 1043 | 0 | 0 | 0 | 0 | 0 | 0 | 0 |
| cg26813483 | 4 | 382 | 5 | 534 | 402 | 0 | 0 | 0 | 0 | 0 | 0 | 0 |
| cg27237477 | 3 | 5 | 83 | 1235 | 0 | 0 | 0 | 0 | 0 | 0 | 0 | 0 |
| cg27389908 | 3 | 11 | 161 | 1151 | 0 | 0 | 0 | 0 | 0 | 0 | 0 | 0 |
| cg27625131 | 4 | 236 | 1 | 623 | 463 | 0 | 0 | 0 | 0 | 0 | 0 | 0 |
| cg00377727 | 5 | 22 | 1 | 193 | 1 | 1106 | 0 | 0 | 0 | 0 | 0 | 0 |
| cg00814218 | 3 | 326 | 3 | 994 | 0 | 0 | 0 | 0 | 0 | 0 | 0 | 0 |
| cg00939438 | 6 | 24 | 1 | 1 | 2 | 228 | 1067 | 0 | 0 | 0 | 0 | 0 |
| cg00993903 | 3 | 79 | 1 | 1243 | 0 | 0 | 0 | 0 | 0 | 0 | 0 | 0 |
| cg01022501 | 5 | 47 | 2 | 1 | 228 | 1045 | 0 | 0 | 0 | 0 | 0 | 0 |
| cg01118640 | 3 | 203 | 1 | 1119 | 0 | 0 | 0 | 0 | 0 | 0 | 0 | 0 |
| cg01221582 | 4 | 28 | 1 | 164 | 1130 | 0 | 0 | 0 | 0 | 0 | 0 | 0 |
| cg01225004 | 3 | 48 | 406 | 869 | 0 | 0 | 0 | 0 | 0 | 0 | 0 | 0 |
| cg01517188 | 3 | 10 | 115 | 1198 | 0 | 0 | 0 | 0 | 0 | 0 | 0 | 0 |
| cg01543583 | 3 | 458 | 3 | 862 | 0 | 0 | 0 | 0 | 0 | 0 | 0 | 0 |
| cg01882774 | 2 | 78 | 1245 | 0 | 0 | 0 | 0 | 0 | 0 | 0 | 0 | 0 |
| cg01943931 | 3 | 33 | 285 | 1005 | 0 | 0 | 0 | 0 | 0 | 0 | 0 | 0 |
| cg02898977 | 4 | 17 | 1 | 187 | 1118 | 0 | 0 | 0 | 0 | 0 | 0 | 0 |
| cg02907150 | 3 | 208 | 2 | 1113 | 0 | 0 | 0 | 0 | 0 | 0 | 0 | 0 |
| cg03004222 | 2 | 88 | 1235 | 0 | 0 | 0 | 0 | 0 | 0 | 0 | 0 | 0 |
| cg03542797 | 5 | 20 | 2 | 6 | 156 | 1139 | 0 | 0 | 0 | 0 | 0 | 0 |
| cg04187846 | 4 | 4 | 75 | 1 | 1243 | 0 | 0 | 0 | 0 | 0 | 0 | 0 |
| cg05097165 | 3 | 26 | 255 | 1042 | 0 | 0 | 0 | 0 | 0 | 0 | 0 | 0 |
| cg05111898 | 3 | 12 | 175 | 1136 | 0 | 0 | 0 | 0 | 0 | 0 | 0 | 0 |
| cg05865327 | 4 | 39 | 1 | 341 | 942 | 0 | 0 | 0 | 0 | 0 | 0 | 0 |
| cg05865331 | 3 | 4 | 261 | 1058 | 0 | 0 | 0 | 0 | 0 | 0 | 0 | 0 |
| cg05940425 | 4 | 91 | 1 | 345 | 886 | 0 | 0 | 0 | 0 | 0 | 0 | 0 |
| cg07170252 | 2 | 104 | 1219 | 0 | 0 | 0 | 0 | 0 | 0 | 0 | 0 | 0 |
| cg07456585 | 3 | 192 | 1 | 1130 | 0 | 0 | 0 | 0 | 0 | 0 | 0 | 0 |
| cg07572984 | 3 | 115 | 2 | 1206 | 0 | 0 | 0 | 0 | 0 | 0 | 0 | 0 |
| cg08210706 | 4 | 112 | 1 | 505 | 705 | 0 | 0 | 0 | 0 | 0 | 0 | 0 |
| cg08253809 | 3 | 580 | 524 | 219 | 0 | 0 | 0 | 0 | 0 | 0 | 0 | 0 |
| cg09214175 | 2 | 484 | 839 | 0 | 0 | 0 | 0 | 0 | 0 | 0 | 0 | 0 |
| cg09241561 | 3 | 5 | 73 | 1245 | 0 | 0 | 0 | 0 | 0 | 0 | 0 | 0 |
| cg09849871 | 2 | 120 | 1203 | 0 | 0 | 0 | 0 | 0 | 0 | 0 | 0 | 0 |
| cg09856996 | 3 | 115 | 567 | 641 | 0 | 0 | 0 | 0 | 0 | 0 | 0 | 0 |
| cg09924366 | 3 | 4 | 77 | 1242 | 0 | 0 | 0 | 0 | 0 | 0 | 0 | 0 |
| cg09998151 | 3 | 554 | 2 | 767 | 0 | 0 | 0 | 0 | 0 | 0 | 0 | 0 |
| cg10110027 | 3 | 1 | 67 | 1255 | 0 | 0 | 0 | 0 | 0 | 0 | 0 | 0 |
| cg10744079 | 5 | 1040 | 2 | 246 | 1 | 34 | 0 | 0 | 0 | 0 | 0 | 0 |
| cg11054397 | 4 | 5 | 1 | 167 | 1150 | 0 | 0 | 0 | 0 | 0 | 0 | 0 |
| cg11857805 | 4 | 301 | 2 | 535 | 485 | 0 | 0 | 0 | 0 | 0 | 0 | 0 |
| cg12090994 | 3 | 9 | 82 | 1232 | 0 | 0 | 0 | 0 | 0 | 0 | 0 | 0 |
| cg12196570 | 3 | 23 | 198 | 1102 | 0 | 0 | 0 | 0 | 0 | 0 | 0 | 0 |
| cg12969729 | 3 | 3 | 108 | 1212 | 0 | 0 | 0 | 0 | 0 | 0 | 0 | 0 |
| cg13251750 | 5 | 148 | 3 | 490 | 3 | 679 | 0 | 0 | 0 | 0 | 0 | 0 |
| cg13655169 | 4 | 790 | 449 | 1 | 83 | 0 | 0 | 0 | 0 | 0 | 0 | 0 |
| cg13685349 | 3 | 80 | 2 | 1241 | 0 | 0 | 0 | 0 | 0 | 0 | 0 | 0 |
| cg13811867 | 3 | 75 | 1 | 1247 | 0 | 0 | 0 | 0 | 0 | 0 | 0 | 0 |
| cg14113778 | 3 | 8 | 93 | 1222 | 0 | 0 | 0 | 0 | 0 | 0 | 0 | 0 |
| cg14175932 | 3 | 221 | 1 | 1101 | 0 | 0 | 0 | 0 | 0 | 0 | 0 | 0 |
| cg14218851 | 2 | 181 | 1142 | 0 | 0 | 0 | 0 | 0 | 0 | 0 | 0 | 0 |
| cg15425276 | 2 | 116 | 1207 | 0 | 0 | 0 | 0 | 0 | 0 | 0 | 0 | 0 |
| cg15876198 | 5 | 13 | 1 | 213 | 2 | 1094 | 0 | 0 | 0 | 0 | 0 | 0 |
| cg16147201 | 2 | 129 | 1194 | 0 | 0 | 0 | 0 | 0 | 0 | 0 | 0 | 0 |
| cg16303048 | 3 | 91 | 497 | 735 | 0 | 0 | 0 | 0 | 0 | 0 | 0 | 0 |
| cg16412745 | 2 | 215 | 1108 | 0 | 0 | 0 | 0 | 0 | 0 | 0 | 0 | 0 |
| cg16702660 | 3 | 461 | 596 | 266 | 0 | 0 | 0 | 0 | 0 | 0 | 0 | 0 |
| cg17370616 | 3 | 182 | 615 | 526 | 0 | 0 | 0 | 0 | 0 | 0 | 0 | 0 |
| cg18239511 | 4 | 699 | 2 | 447 | 175 | 0 | 0 | 0 | 0 | 0 | 0 | 0 |
| cg18486231 | 3 | 68 | 1 | 1254 | 0 | 0 | 0 | 0 | 0 | 0 | 0 | 0 |
| cg18709904 | 3 | 112 | 531 | 680 | 0 | 0 | 0 | 0 | 0 | 0 | 0 | 0 |
| cg18949721 | 3 | 1 | 829 | 493 | 0 | 0 | 0 | 0 | 0 | 0 | 0 | 0 |
| cg19503462 | 4 | 68 | 1 | 1248 | 6 | 0 | 0 | 0 | 0 | 0 | 0 | 0 |
| cg19539986 | 6 | 89 | 1 | 1 | 360 | 2 | 870 | 0 | 0 | 0 | 0 | 0 |
| cg19555075 | 4 | 137 | 3 | 2 | 1181 | 0 | 0 | 0 | 0 | 0 | 0 | 0 |
| cg19653246 | 4 | 131 | 605 | 2 | 585 | 0 | 0 | 0 | 0 | 0 | 0 | 0 |
| cg20904336 | 4 | 95 | 2 | 463 | 763 | 0 | 0 | 0 | 0 | 0 | 0 | 0 |
| cg20963002 | 3 | 2 | 72 | 1249 | 0 | 0 | 0 | 0 | 0 | 0 | 0 | 0 |
| cg21193926 | 4 | 522 | 3 | 567 | 231 | 0 | 0 | 0 | 0 | 0 | 0 | 0 |
| cg21903981 | 2 | 414 | 909 | 0 | 0 | 0 | 0 | 0 | 0 | 0 | 0 | 0 |
| cg22857670 | 2 | 101 | 1222 | 0 | 0 | 0 | 0 | 0 | 0 | 0 | 0 | 0 |
| cg23022053 | 4 | 47 | 373 | 1 | 902 | 0 | 0 | 0 | 0 | 0 | 0 | 0 |
| cg23418075 | 5 | 1053 | 1 | 252 | 1 | 16 | 0 | 0 | 0 | 0 | 0 | 0 |
| cg23421896 | 6 | 22 | 1 | 1 | 1 | 205 | 1093 | 0 | 0 | 0 | 0 | 0 |
| cg23423607 | 3 | 84 | 2 | 1237 | 0 | 0 | 0 | 0 | 0 | 0 | 0 | 0 |
| cg23438516 | 4 | 696 | 2 | 624 | 1 | 0 | 0 | 0 | 0 | 0 | 0 | 0 |
| cg23691406 | 3 | 82 | 1 | 1240 | 0 | 0 | 0 | 0 | 0 | 0 | 0 | 0 |
| cg24014990 | 4 | 1 | 67 | 1 | 1254 | 0 | 0 | 0 | 0 | 0 | 0 | 0 |
| cg24019144 | 5 | 8 | 84 | 1 | 1 | 1229 | 0 | 0 | 0 | 0 | 0 | 0 |
| cg24087071 | 5 | 160 | 1 | 506 | 3 | 653 | 0 | 0 | 0 | 0 | 0 | 0 |
| cg25061027 | 5 | 2 | 2 | 1 | 75 | 1243 | 0 | 0 | 0 | 0 | 0 | 0 |
| cg26103168 | 4 | 14 | 1 | 151 | 1157 | 0 | 0 | 0 | 0 | 0 | 0 | 0 |
| cg26936989 | 3 | 158 | 6 | 1159 | 0 | 0 | 0 | 0 | 0 | 0 | 0 | 0 |
| cg27035997 | 3 | 8 | 181 | 1134 | 0 | 0 | 0 | 0 | 0 | 0 | 0 | 0 |
| cg27056740 | 2 | 417 | 906 | 0 | 0 | 0 | 0 | 0 | 0 | 0 | 0 | 0 |
| cg27662838 | 2 | 84 | 1239 | 0 | 0 | 0 | 0 | 0 | 0 | 0 | 0 | 0 |
| cg00146240 | 4 | 214 | 1 | 625 | 483 | 0 | 0 | 0 | 0 | 0 | 0 | 0 |
| cg01451277 | 4 | 11 | 111 | 1 | 1200 | 0 | 0 | 0 | 0 | 0 | 0 | 0 |
| cg01702501 | 2 | 112 | 1211 | 0 | 0 | 0 | 0 | 0 | 0 | 0 | 0 | 0 |
| cg02031121 | 4 | 1 | 88 | 2 | 1232 | 0 | 0 | 0 | 0 | 0 | 0 | 0 |
| cg02074316 | 5 | 921 | 3 | 340 | 1 | 58 | 0 | 0 | 0 | 0 | 0 | 0 |
| cg02401352 | 3 | 168 | 482 | 673 | 0 | 0 | 0 | 0 | 0 | 0 | 0 | 0 |
| cg02757577 | 3 | 1 | 258 | 1064 | 0 | 0 | 0 | 0 | 0 | 0 | 0 | 0 |
| cg03020684 | 4 | 105 | 1 | 1 | 1216 | 0 | 0 | 0 | 0 | 0 | 0 | 0 |
| cg03358735 | 4 | 6 | 1 | 154 | 1162 | 0 | 0 | 0 | 0 | 0 | 0 | 0 |
| cg04480376 | 3 | 213 | 1108 | 2 | 0 | 0 | 0 | 0 | 0 | 0 | 0 | 0 |
| cg04579183 | 3 | 164 | 1 | 1158 | 0 | 0 | 0 | 0 | 0 | 0 | 0 | 0 |
| cg04775232 | 5 | 9 | 105 | 1 | 1204 | 4 | 0 | 0 | 0 | 0 | 0 | 0 |
| cg04875706 | 3 | 297 | 1 | 1025 | 0 | 0 | 0 | 0 | 0 | 0 | 0 | 0 |
| cg05320460 | 3 | 218 | 1 | 1104 | 0 | 0 | 0 | 0 | 0 | 0 | 0 | 0 |
| cg05472380 | 5 | 9 | 1 | 147 | 2 | 1164 | 0 | 0 | 0 | 0 | 0 | 0 |
| cg06899226 | 5 | 6 | 1 | 3 | 106 | 1207 | 0 | 0 | 0 | 0 | 0 | 0 |
| cg07084345 | 4 | 10 | 1 | 240 | 1072 | 0 | 0 | 0 | 0 | 0 | 0 | 0 |
| cg07216619 | 3 | 256 | 2 | 1065 | 0 | 0 | 0 | 0 | 0 | 0 | 0 | 0 |
| cg07318335 | 6 | 6 | 1 | 2 | 1 | 150 | 1163 | 0 | 0 | 0 | 0 | 0 |
| cg07882838 | 5 | 124 | 1 | 493 | 1 | 704 | 0 | 0 | 0 | 0 | 0 | 0 |
| cg08049519 | 5 | 357 | 1 | 3 | 577 | 385 | 0 | 0 | 0 | 0 | 0 | 0 |
| cg08103472 | 2 | 346 | 977 | 0 | 0 | 0 | 0 | 0 | 0 | 0 | 0 | 0 |
| cg09785377 | 2 | 99 | 1224 | 0 | 0 | 0 | 0 | 0 | 0 | 0 | 0 | 0 |
| cg09908042 | 4 | 94 | 1 | 323 | 905 | 0 | 0 | 0 | 0 | 0 | 0 | 0 |
| cg10095033 | 3 | 6 | 156 | 1161 | 0 | 0 | 0 | 0 | 0 | 0 | 0 | 0 |
| cg10167978 | 3 | 7 | 143 | 1173 | 0 | 0 | 0 | 0 | 0 | 0 | 0 | 0 |
| cg10389148 | 6 | 3 | 1 | 80 | 1 | 1234 | 4 | 0 | 0 | 0 | 0 | 0 |
| cg10530344 | 3 | 124 | 1198 | 1 | 0 | 0 | 0 | 0 | 0 | 0 | 0 | 0 |
| cg10648125 | 5 | 40 | 167 | 1 | 2 | 1113 | 0 | 0 | 0 | 0 | 0 | 0 |
| cg11314779 | 5 | 102 | 3 | 534 | 1 | 683 | 0 | 0 | 0 | 0 | 0 | 0 |
| cg11418607 | 4 | 362 | 1 | 624 | 336 | 0 | 0 | 0 | 0 | 0 | 0 | 0 |
| cg12036633 | 3 | 393 | 556 | 374 | 0 | 0 | 0 | 0 | 0 | 0 | 0 | 0 |
| cg12339131 | 3 | 101 | 4 | 1218 | 0 | 0 | 0 | 0 | 0 | 0 | 0 | 0 |
| cg12391372 | 3 | 133 | 1 | 1189 | 0 | 0 | 0 | 0 | 0 | 0 | 0 | 0 |
| cg12562232 | 5 | 2 | 100 | 1 | 1 | 1219 | 0 | 0 | 0 | 0 | 0 | 0 |
| cg13067974 | 4 | 16 | 51 | 2 | 1254 | 0 | 0 | 0 | 0 | 0 | 0 | 0 |
| cg13194353 | 4 | 9 | 1 | 123 | 1190 | 0 | 0 | 0 | 0 | 0 | 0 | 0 |
| cg13782322 | 4 | 27 | 1 | 210 | 1085 | 0 | 0 | 0 | 0 | 0 | 0 | 0 |
| cg14923124 | 4 | 5 | 59 | 3 | 1256 | 0 | 0 | 0 | 0 | 0 | 0 | 0 |
| cg15329552 | 2 | 67 | 1256 | 0 | 0 | 0 | 0 | 0 | 0 | 0 | 0 | 0 |
| cg15456414 | 4 | 11 | 1 | 168 | 1143 | 0 | 0 | 0 | 0 | 0 | 0 | 0 |
| cg16416584 | 3 | 8 | 105 | 1210 | 0 | 0 | 0 | 0 | 0 | 0 | 0 | 0 |
| cg16590643 | 3 | 1 | 67 | 1255 | 0 | 0 | 0 | 0 | 0 | 0 | 0 | 0 |
| cg17876294 | 2 | 138 | 1185 | 0 | 0 | 0 | 0 | 0 | 0 | 0 | 0 | 0 |
| cg18258571 | 4 | 18 | 243 | 1 | 1061 | 0 | 0 | 0 | 0 | 0 | 0 | 0 |
| cg18746157 | 3 | 12 | 212 | 1099 | 0 | 0 | 0 | 0 | 0 | 0 | 0 | 0 |
| cg19495614 | 3 | 108 | 1 | 1214 | 0 | 0 | 0 | 0 | 0 | 0 | 0 | 0 |
| cg19949776 | 4 | 334 | 1 | 660 | 328 | 0 | 0 | 0 | 0 | 0 | 0 | 0 |
| cg20386487 | 2 | 1212 | 111 | 0 | 0 | 0 | 0 | 0 | 0 | 0 | 0 | 0 |
| cg20976286 | 3 | 334 | 1 | 988 | 0 | 0 | 0 | 0 | 0 | 0 | 0 | 0 |
| cg21566433 | 4 | 210 | 636 | 1 | 476 | 0 | 0 | 0 | 0 | 0 | 0 | 0 |
| cg21575308 | 3 | 333 | 986 | 4 | 0 | 0 | 0 | 0 | 0 | 0 | 0 | 0 |
| cg22142142 | 4 | 110 | 1 | 533 | 679 | 0 | 0 | 0 | 0 | 0 | 0 | 0 |
| cg22166325 | 4 | 1249 | 71 | 1 | 2 | 0 | 0 | 0 | 0 | 0 | 0 | 0 |
| cg22603433 | 3 | 1198 | 113 | 12 | 0 | 0 | 0 | 0 | 0 | 0 | 0 | 0 |
| cg22681495 | 2 | 84 | 1239 | 0 | 0 | 0 | 0 | 0 | 0 | 0 | 0 | 0 |
| cg22835630 | 3 | 35 | 304 | 984 | 0 | 0 | 0 | 0 | 0 | 0 | 0 | 0 |
| cg23450377 | 5 | 1 | 1 | 1 | 65 | 1255 | 0 | 0 | 0 | 0 | 0 | 0 |
| cg23741255 | 4 | 9 | 4 | 131 | 1179 | 0 | 0 | 0 | 0 | 0 | 0 | 0 |
| cg23840008 | 6 | 416 | 1 | 3 | 532 | 370 | 1 | 0 | 0 | 0 | 0 | 0 |
| cg24961286 | 2 | 186 | 1137 | 0 | 0 | 0 | 0 | 0 | 0 | 0 | 0 | 0 |
| cg25450321 | 3 | 579 | 2 | 742 | 0 | 0 | 0 | 0 | 0 | 0 | 0 | 0 |
| cg25879395 | 5 | 106 | 1 | 498 | 1 | 717 | 0 | 0 | 0 | 0 | 0 | 0 |
| cg26217827 | 4 | 81 | 3 | 471 | 768 | 0 | 0 | 0 | 0 | 0 | 0 | 0 |
| cg26889118 | 4 | 115 | 2 | 513 | 693 | 0 | 0 | 0 | 0 | 0 | 0 | 0 |
| cg27018984 | 3 | 194 | 1 | 1128 | 0 | 0 | 0 | 0 | 0 | 0 | 0 | 0 |
| cg27224751 | 2 | 79 | 1244 | 0 | 0 | 0 | 0 | 0 | 0 | 0 | 0 | 0 |
| cg27395839 | 4 | 2 | 98 | 5 | 1218 | 0 | 0 | 0 | 0 | 0 | 0 | 0 |
| cg27639199 | 4 | 365 | 1 | 581 | 376 | 0 | 0 | 0 | 0 | 0 | 0 | 0 |
| cg00027155 | 4 | 17 | 210 | 1 | 1095 | 0 | 0 | 0 | 0 | 0 | 0 | 0 |
| cg00274640 | 4 | 209 | 3 | 1110 | 1 | 0 | 0 | 0 | 0 | 0 | 0 | 0 |
| cg00586531 | 3 | 97 | 1 | 1225 | 0 | 0 | 0 | 0 | 0 | 0 | 0 | 0 |
| cg00616572 | 6 | 116 | 1 | 1 | 399 | 1 | 805 | 0 | 0 | 0 | 0 | 0 |
| cg00713204 | 2 | 571 | 752 | 0 | 0 | 0 | 0 | 0 | 0 | 0 | 0 | 0 |
| cg01052274 | 3 | 34 | 207 | 1082 | 0 | 0 | 0 | 0 | 0 | 0 | 0 | 0 |
| cg01074083 | 4 | 12 | 242 | 1 | 1068 | 0 | 0 | 0 | 0 | 0 | 0 | 0 |
| cg01262217 | 3 | 5 | 89 | 1229 | 0 | 0 | 0 | 0 | 0 | 0 | 0 | 0 |
| cg01283685 | 3 | 5 | 83 | 1235 | 0 | 0 | 0 | 0 | 0 | 0 | 0 | 0 |
| cg01491428 | 4 | 154 | 2 | 447 | 720 | 0 | 0 | 0 | 0 | 0 | 0 | 0 |
| cg01608400 | 4 | 3 | 88 | 2 | 1230 | 0 | 0 | 0 | 0 | 0 | 0 | 0 |
| cg01733958 | 4 | 383 | 7 | 3 | 930 | 0 | 0 | 0 | 0 | 0 | 0 | 0 |
| cg01891583 | 5 | 308 | 1 | 552 | 1 | 461 | 0 | 0 | 0 | 0 | 0 | 0 |
| cg02248103 | 4 | 23 | 2 | 194 | 1104 | 0 | 0 | 0 | 0 | 0 | 0 | 0 |
| cg02316445 | 2 | 198 | 1125 | 0 | 0 | 0 | 0 | 0 | 0 | 0 | 0 | 0 |
| cg02361439 | 4 | 1 | 81 | 1 | 1240 | 0 | 0 | 0 | 0 | 0 | 0 | 0 |
| cg02389264 | 4 | 30 | 1 | 290 | 1002 | 0 | 0 | 0 | 0 | 0 | 0 | 0 |
| cg02772880 | 3 | 208 | 3 | 1112 | 0 | 0 | 0 | 0 | 0 | 0 | 0 | 0 |
| cg02909570 | 3 | 139 | 1 | 1183 | 0 | 0 | 0 | 0 | 0 | 0 | 0 | 0 |
| cg02978201 | 5 | 431 | 1 | 1 | 561 | 329 | 0 | 0 | 0 | 0 | 0 | 0 |
| cg03051742 | 3 | 16 | 179 | 1128 | 0 | 0 | 0 | 0 | 0 | 0 | 0 | 0 |
| cg03146649 | 4 | 17 | 254 | 1 | 1051 | 0 | 0 | 0 | 0 | 0 | 0 | 0 |
| cg03424554 | 2 | 116 | 1207 | 0 | 0 | 0 | 0 | 0 | 0 | 0 | 0 | 0 |
| cg03448017 | 4 | 73 | 1 | 362 | 887 | 0 | 0 | 0 | 0 | 0 | 0 | 0 |
| cg03553529 | 4 | 9 | 98 | 2 | 1214 | 0 | 0 | 0 | 0 | 0 | 0 | 0 |
| cg03697766 | 4 | 2 | 74 | 1 | 1246 | 0 | 0 | 0 | 0 | 0 | 0 | 0 |
| cg03940883 | 2 | 203 | 1120 | 0 | 0 | 0 | 0 | 0 | 0 | 0 | 0 | 0 |
| cg04245870 | 3 | 20 | 219 | 1084 | 0 | 0 | 0 | 0 | 0 | 0 | 0 | 0 |
| cg04362614 | 3 | 1 | 1234 | 88 | 0 | 0 | 0 | 0 | 0 | 0 | 0 | 0 |
| cg04412904 | 3 | 905 | 357 | 61 | 0 | 0 | 0 | 0 | 0 | 0 | 0 | 0 |
| cg04453550 | 4 | 102 | 1 | 387 | 833 | 0 | 0 | 0 | 0 | 0 | 0 | 0 |
| cg04507446 | 3 | 6 | 116 | 1201 | 0 | 0 | 0 | 0 | 0 | 0 | 0 | 0 |
| cg04612030 | 3 | 10 | 170 | 1143 | 0 | 0 | 0 | 0 | 0 | 0 | 0 | 0 |
| cg04768488 | 4 | 9 | 1 | 216 | 1097 | 0 | 0 | 0 | 0 | 0 | 0 | 0 |
| cg04787784 | 3 | 130 | 515 | 678 | 0 | 0 | 0 | 0 | 0 | 0 | 0 | 0 |
| cg04845171 | 4 | 9 | 1 | 99 | 1214 | 0 | 0 | 0 | 0 | 0 | 0 | 0 |
| cg04963199 | 5 | 210 | 2 | 2 | 1 | 1108 | 0 | 0 | 0 | 0 | 0 | 0 |
| cg05146307 | 3 | 65 | 2 | 1256 | 0 | 0 | 0 | 0 | 0 | 0 | 0 | 0 |
| cg05179499 | 4 | 14 | 192 | 1 | 1116 | 0 | 0 | 0 | 0 | 0 | 0 | 0 |
| cg05208607 | 5 | 482 | 8 | 4 | 2 | 827 | 0 | 0 | 0 | 0 | 0 | 0 |
| cg05287064 | 3 | 9 | 111 | 1203 | 0 | 0 | 0 | 0 | 0 | 0 | 0 | 0 |
| cg05450979 | 4 | 73 | 1 | 425 | 824 | 0 | 0 | 0 | 0 | 0 | 0 | 0 |
| cg05515244 | 4 | 323 | 2 | 2 | 996 | 0 | 0 | 0 | 0 | 0 | 0 | 0 |
| cg05522042 | 2 | 73 | 1250 | 0 | 0 | 0 | 0 | 0 | 0 | 0 | 0 | 0 |
| cg05876883 | 3 | 46 | 360 | 917 | 0 | 0 | 0 | 0 | 0 | 0 | 0 | 0 |
| cg05924729 | 2 | 110 | 1213 | 0 | 0 | 0 | 0 | 0 | 0 | 0 | 0 | 0 |
| cg05977333 | 4 | 55 | 1 | 352 | 915 | 0 | 0 | 0 | 0 | 0 | 0 | 0 |
| cg06052372 | 3 | 150 | 554 | 619 | 0 | 0 | 0 | 0 | 0 | 0 | 0 | 0 |
| cg06103394 | 3 | 1131 | 165 | 27 | 0 | 0 | 0 | 0 | 0 | 0 | 0 | 0 |
| cg06294384 | 3 | 34 | 213 | 1076 | 0 | 0 | 0 | 0 | 0 | 0 | 0 | 0 |
| cg06334689 | 5 | 88 | 1 | 1 | 394 | 839 | 0 | 0 | 0 | 0 | 0 | 0 |
| cg06464078 | 4 | 116 | 1 | 420 | 786 | 0 | 0 | 0 | 0 | 0 | 0 | 0 |
| cg06520095 | 4 | 91 | 1 | 444 | 787 | 0 | 0 | 0 | 0 | 0 | 0 | 0 |
| cg06521835 | 3 | 75 | 1 | 1247 | 0 | 0 | 0 | 0 | 0 | 0 | 0 | 0 |
| cg06578063 | 4 | 2 | 68 | 1 | 1252 | 0 | 0 | 0 | 0 | 0 | 0 | 0 |
| cg06711418 | 3 | 688 | 535 | 100 | 0 | 0 | 0 | 0 | 0 | 0 | 0 | 0 |
| cg06743454 | 4 | 6 | 164 | 2 | 1151 | 0 | 0 | 0 | 0 | 0 | 0 | 0 |
| cg06872709 | 3 | 10 | 119 | 1194 | 0 | 0 | 0 | 0 | 0 | 0 | 0 | 0 |
| cg06907930 | 3 | 5 | 102 | 1216 | 0 | 0 | 0 | 0 | 0 | 0 | 0 | 0 |
| cg06979386 | 3 | 88 | 5 | 1230 | 0 | 0 | 0 | 0 | 0 | 0 | 0 | 0 |
| cg07028768 | 4 | 53 | 446 | 1 | 823 | 0 | 0 | 0 | 0 | 0 | 0 | 0 |
| cg07128503 | 5 | 86 | 1 | 483 | 1 | 752 | 0 | 0 | 0 | 0 | 0 | 0 |
| cg07327347 | 4 | 1 | 1 | 99 | 1222 | 0 | 0 | 0 | 0 | 0 | 0 | 0 |
| cg07437923 | 4 | 607 | 577 | 3 | 136 | 0 | 0 | 0 | 0 | 0 | 0 | 0 |
| cg07460804 | 3 | 167 | 12 | 1144 | 0 | 0 | 0 | 0 | 0 | 0 | 0 | 0 |
| cg07542540 | 3 | 2 | 122 | 1199 | 0 | 0 | 0 | 0 | 0 | 0 | 0 | 0 |
| cg07684647 | 4 | 115 | 2 | 1 | 1205 | 0 | 0 | 0 | 0 | 0 | 0 | 0 |
| cg07869343 | 3 | 18 | 214 | 1091 | 0 | 0 | 0 | 0 | 0 | 0 | 0 | 0 |
| cg07903626 | 3 | 102 | 2 | 1219 | 0 | 0 | 0 | 0 | 0 | 0 | 0 | 0 |
| cg08084984 | 3 | 640 | 408 | 275 | 0 | 0 | 0 | 0 | 0 | 0 | 0 | 0 |
| cg08136432 | 4 | 184 | 140 | 1 | 998 | 0 | 0 | 0 | 0 | 0 | 0 | 0 |
| cg08227260 | 4 | 10 | 148 | 1 | 1164 | 0 | 0 | 0 | 0 | 0 | 0 | 0 |
| cg08581004 | 3 | 37 | 284 | 1002 | 0 | 0 | 0 | 0 | 0 | 0 | 0 | 0 |
| cg08624915 | 4 | 109 | 1 | 431 | 782 | 0 | 0 | 0 | 0 | 0 | 0 | 0 |
| cg08702011 | 4 | 8 | 85 | 1 | 1229 | 0 | 0 | 0 | 0 | 0 | 0 | 0 |
| cg08717807 | 3 | 290 | 629 | 404 | 0 | 0 | 0 | 0 | 0 | 0 | 0 | 0 |
| cg08914678 | 3 | 36 | 284 | 1003 | 0 | 0 | 0 | 0 | 0 | 0 | 0 | 0 |
| cg09351263 | 3 | 462 | 3 | 858 | 0 | 0 | 0 | 0 | 0 | 0 | 0 | 0 |
| cg09610891 | 2 | 92 | 1231 | 0 | 0 | 0 | 0 | 0 | 0 | 0 | 0 | 0 |
| cg09916840 | 3 | 707 | 422 | 194 | 0 | 0 | 0 | 0 | 0 | 0 | 0 | 0 |
| cg10209474 | 6 | 2 | 1 | 1 | 73 | 1 | 1245 | 0 | 0 | 0 | 0 | 0 |
| cg10462897 | 3 | 30 | 259 | 1034 | 0 | 0 | 0 | 0 | 0 | 0 | 0 | 0 |
| cg13782134 | 3 | 7 | 101 | 1215 | 0 | 0 | 0 | 0 | 0 | 0 | 0 | 0 |
| cg16380681 | 3 | 20 | 219 | 1084 | 0 | 0 | 0 | 0 | 0 | 0 | 0 | 0 |
| cg16490805 | 4 | 87 | 1 | 461 | 774 | 0 | 0 | 0 | 0 | 0 | 0 | 0 |
| cg16623098 | 6 | 1 | 2 | 130 | 1 | 1 | 1188 | 0 | 0 | 0 | 0 | 0 |
| cg16658177 | 4 | 12 | 100 | 1 | 1210 | 0 | 0 | 0 | 0 | 0 | 0 | 0 |
| cg16730716 | 3 | 1 | 66 | 1256 | 0 | 0 | 0 | 0 | 0 | 0 | 0 | 0 |
| cg16791832 | 4 | 75 | 1 | 322 | 925 | 0 | 0 | 0 | 0 | 0 | 0 | 0 |
| cg19130301 | 3 | 12 | 112 | 1199 | 0 | 0 | 0 | 0 | 0 | 0 | 0 | 0 |
| cg26359240 | 3 | 6 | 102 | 1215 | 0 | 0 | 0 | 0 | 0 | 0 | 0 | 0 |
| cg26477117 | 5 | 36 | 1 | 1 | 328 | 957 | 0 | 0 | 0 | 0 | 0 | 0 |
| cg26651280 | 4 | 6 | 109 | 1 | 1207 | 0 | 0 | 0 | 0 | 0 | 0 | 0 |
| cg26722972 | 3 | 78 | 281 | 964 | 0 | 0 | 0 | 0 | 0 | 0 | 0 | 0 |
| cg26776784 | 3 | 193 | 1 | 1129 | 0 | 0 | 0 | 0 | 0 | 0 | 0 | 0 |
| cg26786615 | 3 | 185 | 3 | 1135 | 0 | 0 | 0 | 0 | 0 | 0 | 0 | 0 |
| cg26805113 | 5 | 171 | 1 | 1 | 562 | 588 | 0 | 0 | 0 | 0 | 0 | 0 |
| cg26846609 | 4 | 267 | 556 | 1 | 499 | 0 | 0 | 0 | 0 | 0 | 0 | 0 |
| cg27107150 | 5 | 1 | 1 | 1 | 78 | 1242 | 0 | 0 | 0 | 0 | 0 | 0 |
| cg27525020 | 3 | 1 | 82 | 1240 | 0 | 0 | 0 | 0 | 0 | 0 | 0 | 0 |
| cg00095677 | 4 | 38 | 300 | 1 | 984 | 0 | 0 | 0 | 0 | 0 | 0 | 0 |
| cg00232388 | 4 | 8 | 67 | 1 | 1247 | 0 | 0 | 0 | 0 | 0 | 0 | 0 |
| cg00247094 | 4 | 73 | 1 | 375 | 874 | 0 | 0 | 0 | 0 | 0 | 0 | 0 |
| cg00443543 | 4 | 64 | 2 | 443 | 814 | 0 | 0 | 0 | 0 | 0 | 0 | 0 |
| cg00461299 | 3 | 7 | 91 | 1225 | 0 | 0 | 0 | 0 | 0 | 0 | 0 | 0 |
| cg00797779 | 4 | 73 | 201 | 1 | 1048 | 0 | 0 | 0 | 0 | 0 | 0 | 0 |
| cg00830755 | 3 | 3 | 90 | 1230 | 0 | 0 | 0 | 0 | 0 | 0 | 0 | 0 |
| cg00956971 | 3 | 1253 | 68 | 2 | 0 | 0 | 0 | 0 | 0 | 0 | 0 | 0 |
| cg01248855 | 4 | 24 | 1 | 313 | 985 | 0 | 0 | 0 | 0 | 0 | 0 | 0 |
| cg01275521 | 3 | 108 | 3 | 1212 | 0 | 0 | 0 | 0 | 0 | 0 | 0 | 0 |
| cg01979298 | 3 | 191 | 4 | 1128 | 0 | 0 | 0 | 0 | 0 | 0 | 0 | 0 |
| cg02144954 | 5 | 1046 | 1 | 231 | 1 | 44 | 0 | 0 | 0 | 0 | 0 | 0 |
| cg02219949 | 2 | 1024 | 299 | 0 | 0 | 0 | 0 | 0 | 0 | 0 | 0 | 0 |
| cg02619116 | 5 | 1 | 1 | 203 | 1 | 1117 | 0 | 0 | 0 | 0 | 0 | 0 |
| cg02839725 | 4 | 58 | 1 | 409 | 855 | 0 | 0 | 0 | 0 | 0 | 0 | 0 |
| cg03051617 | 3 | 5 | 107 | 1211 | 0 | 0 | 0 | 0 | 0 | 0 | 0 | 0 |
| cg03810198 | 4 | 474 | 847 | 1 | 1 | 0 | 0 | 0 | 0 | 0 | 0 | 0 |
| cg03936229 | 3 | 4 | 71 | 1248 | 0 | 0 | 0 | 0 | 0 | 0 | 0 | 0 |
| cg04029134 | 4 | 5 | 12 | 283 | 1023 | 0 | 0 | 0 | 0 | 0 | 0 | 0 |
| cg04166638 | 5 | 21 | 1 | 281 | 1 | 1019 | 0 | 0 | 0 | 0 | 0 | 0 |
| cg04172345 | 2 | 72 | 1251 | 0 | 0 | 0 | 0 | 0 | 0 | 0 | 0 | 0 |
| cg04212500 | 2 | 653 | 670 | 0 | 0 | 0 | 0 | 0 | 0 | 0 | 0 | 0 |
| cg04368836 | 4 | 34 | 1 | 308 | 980 | 0 | 0 | 0 | 0 | 0 | 0 | 0 |
| cg04417708 | 4 | 36 | 299 | 1 | 987 | 0 | 0 | 0 | 0 | 0 | 0 | 0 |
| cg04487202 | 4 | 131 | 3 | 2 | 1187 | 0 | 0 | 0 | 0 | 0 | 0 | 0 |
| cg04497611 | 5 | 82 | 1 | 1 | 496 | 743 | 0 | 0 | 0 | 0 | 0 | 0 |
| cg04589021 | 3 | 146 | 1 | 1176 | 0 | 0 | 0 | 0 | 0 | 0 | 0 | 0 |
| cg05033939 | 3 | 21 | 151 | 1151 | 0 | 0 | 0 | 0 | 0 | 0 | 0 | 0 |
| cg05161773 | 2 | 128 | 1195 | 0 | 0 | 0 | 0 | 0 | 0 | 0 | 0 | 0 |
| cg05187322 | 4 | 6 | 178 | 2 | 1137 | 0 | 0 | 0 | 0 | 0 | 0 | 0 |
| cg05280794 | 5 | 108 | 1 | 2 | 369 | 843 | 0 | 0 | 0 | 0 | 0 | 0 |
| cg05305893 | 5 | 207 | 1 | 2 | 1 | 1112 | 0 | 0 | 0 | 0 | 0 | 0 |
| cg05331763 | 4 | 132 | 1 | 477 | 713 | 0 | 0 | 0 | 0 | 0 | 0 | 0 |
| cg05357619 | 3 | 1 | 71 | 1251 | 0 | 0 | 0 | 0 | 0 | 0 | 0 | 0 |
| cg05813498 | 4 | 56 | 419 | 1 | 847 | 0 | 0 | 0 | 0 | 0 | 0 | 0 |
| cg06147194 | 4 | 4 | 77 | 2 | 1240 | 0 | 0 | 0 | 0 | 0 | 0 | 0 |
| cg06589596 | 3 | 3 | 78 | 1242 | 0 | 0 | 0 | 0 | 0 | 0 | 0 | 0 |
| cg06929471 | 4 | 5 | 100 | 1 | 1217 | 0 | 0 | 0 | 0 | 0 | 0 | 0 |
| cg06939759 | 2 | 123 | 1200 | 0 | 0 | 0 | 0 | 0 | 0 | 0 | 0 | 0 |
| cg07073561 | 2 | 552 | 771 | 0 | 0 | 0 | 0 | 0 | 0 | 0 | 0 | 0 |
| cg07446795 | 4 | 16 | 142 | 1 | 1164 | 0 | 0 | 0 | 0 | 0 | 0 | 0 |
| cg07760800 | 2 | 129 | 1194 | 0 | 0 | 0 | 0 | 0 | 0 | 0 | 0 | 0 |
| cg07879897 | 3 | 1173 | 140 | 10 | 0 | 0 | 0 | 0 | 0 | 0 | 0 | 0 |
| cg07973125 | 3 | 14 | 238 | 1071 | 0 | 0 | 0 | 0 | 0 | 0 | 0 | 0 |
| cg08002427 | 3 | 116 | 1 | 1206 | 0 | 0 | 0 | 0 | 0 | 0 | 0 | 0 |
| cg08024264 | 2 | 480 | 843 | 0 | 0 | 0 | 0 | 0 | 0 | 0 | 0 | 0 |
| cg08102564 | 4 | 102 | 1 | 479 | 741 | 0 | 0 | 0 | 0 | 0 | 0 | 0 |
| cg08699174 | 3 | 3 | 88 | 1232 | 0 | 0 | 0 | 0 | 0 | 0 | 0 | 0 |
| cg09015880 | 3 | 45 | 315 | 963 | 0 | 0 | 0 | 0 | 0 | 0 | 0 | 0 |
| cg09813647 | 3 | 16 | 226 | 1081 | 0 | 0 | 0 | 0 | 0 | 0 | 0 | 0 |
| cg10760240 | 5 | 9 | 1 | 144 | 1 | 1168 | 0 | 0 | 0 | 0 | 0 | 0 |
| cg10791966 | 3 | 1 | 91 | 1231 | 0 | 0 | 0 | 0 | 0 | 0 | 0 | 0 |
| cg11144103 | 4 | 490 | 1 | 514 | 318 | 0 | 0 | 0 | 0 | 0 | 0 | 0 |
| cg11189272 | 6 | 9 | 1 | 151 | 1 | 1 | 1160 | 0 | 0 | 0 | 0 | 0 |
| cg11331837 | 3 | 357 | 556 | 410 | 0 | 0 | 0 | 0 | 0 | 0 | 0 | 0 |
| cg11620135 | 3 | 24 | 283 | 1016 | 0 | 0 | 0 | 0 | 0 | 0 | 0 | 0 |
| cg12209881 | 4 | 2 | 84 | 1 | 1236 | 0 | 0 | 0 | 0 | 0 | 0 | 0 |
| cg12432807 | 4 | 28 | 1 | 254 | 1040 | 0 | 0 | 0 | 0 | 0 | 0 | 0 |
| cg13098428 | 3 | 67 | 1 | 1255 | 0 | 0 | 0 | 0 | 0 | 0 | 0 | 0 |
| cg13133387 | 3 | 5 | 131 | 1187 | 0 | 0 | 0 | 0 | 0 | 0 | 0 | 0 |
| cg13183651 | 4 | 134 | 1 | 1186 | 2 | 0 | 0 | 0 | 0 | 0 | 0 | 0 |
| cg13213536 | 3 | 72 | 7 | 1244 | 0 | 0 | 0 | 0 | 0 | 0 | 0 | 0 |
| cg13347397 | 3 | 9 | 203 | 1111 | 0 | 0 | 0 | 0 | 0 | 0 | 0 | 0 |
| cg13603318 | 4 | 426 | 2 | 1 | 894 | 0 | 0 | 0 | 0 | 0 | 0 | 0 |
| cg13616314 | 6 | 2 | 1 | 1 | 92 | 2 | 1225 | 0 | 0 | 0 | 0 | 0 |
| cg13619177 | 3 | 5 | 83 | 1235 | 0 | 0 | 0 | 0 | 0 | 0 | 0 | 0 |
| cg13723217 | 4 | 1205 | 111 | 1 | 6 | 0 | 0 | 0 | 0 | 0 | 0 | 0 |
| cg13916177 | 3 | 67 | 1 | 1255 | 0 | 0 | 0 | 0 | 0 | 0 | 0 | 0 |
| cg13989295 | 4 | 539 | 1 | 556 | 227 | 0 | 0 | 0 | 0 | 0 | 0 | 0 |
| cg14001750 | 2 | 216 | 1107 | 0 | 0 | 0 | 0 | 0 | 0 | 0 | 0 | 0 |
| cg14192979 | 6 | 553 | 2 | 1 | 561 | 205 | 1 | 0 | 0 | 0 | 0 | 0 |
| cg14195178 | 5 | 98 | 1 | 1 | 515 | 708 | 0 | 0 | 0 | 0 | 0 | 0 |
| cg14885973 | 3 | 45 | 304 | 974 | 0 | 0 | 0 | 0 | 0 | 0 | 0 | 0 |
| cg15132295 | 4 | 136 | 485 | 1 | 701 | 0 | 0 | 0 | 0 | 0 | 0 | 0 |
| cg15292356 | 2 | 89 | 1234 | 0 | 0 | 0 | 0 | 0 | 0 | 0 | 0 | 0 |
| cg16144436 | 4 | 307 | 2 | 3 | 1011 | 0 | 0 | 0 | 0 | 0 | 0 | 0 |
| cg16145187 | 3 | 292 | 1030 | 1 | 0 | 0 | 0 | 0 | 0 | 0 | 0 | 0 |
| cg16187528 | 4 | 11 | 1 | 160 | 1151 | 0 | 0 | 0 | 0 | 0 | 0 | 0 |
| cg16338321 | 3 | 81 | 3 | 1239 | 0 | 0 | 0 | 0 | 0 | 0 | 0 | 0 |
| cg16377738 | 3 | 84 | 2 | 1237 | 0 | 0 | 0 | 0 | 0 | 0 | 0 | 0 |
| cg16446617 | 4 | 3 | 95 | 1 | 1224 | 0 | 0 | 0 | 0 | 0 | 0 | 0 |
| cg16515381 | 5 | 68 | 1 | 2 | 2 | 1250 | 0 | 0 | 0 | 0 | 0 | 0 |
| cg16518167 | 3 | 5 | 88 | 1230 | 0 | 0 | 0 | 0 | 0 | 0 | 0 | 0 |
| cg16836675 | 3 | 122 | 1 | 1200 | 0 | 0 | 0 | 0 | 0 | 0 | 0 | 0 |
| cg17225604 | 3 | 116 | 4 | 1203 | 0 | 0 | 0 | 0 | 0 | 0 | 0 | 0 |
| cg17735193 | 3 | 199 | 1 | 1123 | 0 | 0 | 0 | 0 | 0 | 0 | 0 | 0 |
| cg18527716 | 4 | 4 | 150 | 1 | 1168 | 0 | 0 | 0 | 0 | 0 | 0 | 0 |
| cg19014289 | 4 | 26 | 2 | 200 | 1095 | 0 | 0 | 0 | 0 | 0 | 0 | 0 |
| cg19026811 | 4 | 1 | 95 | 2 | 1225 | 0 | 0 | 0 | 0 | 0 | 0 | 0 |
| cg19079513 | 5 | 111 | 1 | 1 | 524 | 686 | 0 | 0 | 0 | 0 | 0 | 0 |
| cg19759847 | 3 | 4 | 181 | 1138 | 0 | 0 | 0 | 0 | 0 | 0 | 0 | 0 |
| cg19791271 | 3 | 135 | 1 | 1187 | 0 | 0 | 0 | 0 | 0 | 0 | 0 | 0 |
| cg19832565 | 2 | 136 | 1187 | 0 | 0 | 0 | 0 | 0 | 0 | 0 | 0 | 0 |
| cg20086657 | 4 | 456 | 1 | 607 | 259 | 0 | 0 | 0 | 0 | 0 | 0 | 0 |
| cg20459037 | 5 | 522 | 1 | 2 | 402 | 396 | 0 | 0 | 0 | 0 | 0 | 0 |
| cg20502501 | 3 | 303 | 6 | 1014 | 0 | 0 | 0 | 0 | 0 | 0 | 0 | 0 |
| cg20756026 | 5 | 332 | 1 | 523 | 1 | 466 | 0 | 0 | 0 | 0 | 0 | 0 |
| cg20777128 | 3 | 1214 | 107 | 2 | 0 | 0 | 0 | 0 | 0 | 0 | 0 | 0 |
| cg21035183 | 3 | 1 | 92 | 1230 | 0 | 0 | 0 | 0 | 0 | 0 | 0 | 0 |
| cg21320242 | 4 | 7 | 1 | 137 | 1178 | 0 | 0 | 0 | 0 | 0 | 0 | 0 |
| cg21578644 | 2 | 129 | 1194 | 0 | 0 | 0 | 0 | 0 | 0 | 0 | 0 | 0 |
| cg21978135 | 2 | 250 | 1073 | 0 | 0 | 0 | 0 | 0 | 0 | 0 | 0 | 0 |
| cg21997403 | 4 | 2 | 1 | 105 | 1215 | 0 | 0 | 0 | 0 | 0 | 0 | 0 |
| cg22094923 | 3 | 6 | 72 | 1245 | 0 | 0 | 0 | 0 | 0 | 0 | 0 | 0 |
| cg22222799 | 3 | 12 | 242 | 1069 | 0 | 0 | 0 | 0 | 0 | 0 | 0 | 0 |
| cg22309983 | 4 | 140 | 230 | 1 | 952 | 0 | 0 | 0 | 0 | 0 | 0 | 0 |
| cg23117085 | 5 | 12 | 1 | 164 | 1 | 1145 | 0 | 0 | 0 | 0 | 0 | 0 |
| cg23246815 | 4 | 67 | 1 | 1 | 1254 | 0 | 0 | 0 | 0 | 0 | 0 | 0 |
| cg23767840 | 4 | 7 | 148 | 1 | 1167 | 0 | 0 | 0 | 0 | 0 | 0 | 0 |
| cg24057558 | 2 | 149 | 1174 | 0 | 0 | 0 | 0 | 0 | 0 | 0 | 0 | 0 |
| cg24402183 | 4 | 3 | 1 | 74 | 1245 | 0 | 0 | 0 | 0 | 0 | 0 | 0 |
| cg24801230 | 4 | 14 | 1 | 245 | 1063 | 0 | 0 | 0 | 0 | 0 | 0 | 0 |
| cg24930059 | 5 | 6 | 1 | 80 | 1 | 1235 | 0 | 0 | 0 | 0 | 0 | 0 |
| cg25388952 | 2 | 334 | 989 | 0 | 0 | 0 | 0 | 0 | 0 | 0 | 0 | 0 |
| cg25450121 | 4 | 803 | 428 | 1 | 91 | 0 | 0 | 0 | 0 | 0 | 0 | 0 |
| cg25873514 | 3 | 22 | 344 | 957 | 0 | 0 | 0 | 0 | 0 | 0 | 0 | 0 |
| cg25929399 | 3 | 871 | 370 | 82 | 0 | 0 | 0 | 0 | 0 | 0 | 0 | 0 |
| cg25988106 | 3 | 1 | 91 | 1231 | 0 | 0 | 0 | 0 | 0 | 0 | 0 | 0 |
| cg26287080 | 3 | 81 | 1 | 1241 | 0 | 0 | 0 | 0 | 0 | 0 | 0 | 0 |
| cg26846076 | 5 | 105 | 1 | 469 | 1 | 747 | 0 | 0 | 0 | 0 | 0 | 0 |
| cg26848300 | 3 | 141 | 1 | 1181 | 0 | 0 | 0 | 0 | 0 | 0 | 0 | 0 |
| cg26932839 | 4 | 99 | 3 | 3 | 1218 | 0 | 0 | 0 | 0 | 0 | 0 | 0 |
| cg26935330 | 4 | 6 | 9 | 163 | 1145 | 0 | 0 | 0 | 0 | 0 | 0 | 0 |
| cg26966808 | 5 | 1 | 79 | 1 | 1 | 1241 | 0 | 0 | 0 | 0 | 0 | 0 |
| cg27500337 | 3 | 15 | 227 | 1081 | 0 | 0 | 0 | 0 | 0 | 0 | 0 | 0 |
| cg27659796 | 2 | 261 | 1062 | 0 | 0 | 0 | 0 | 0 | 0 | 0 | 0 | 0 |
| cg00254095 | 2 | 348 | 975 | 0 | 0 | 0 | 0 | 0 | 0 | 0 | 0 | 0 |
| cg00348031 | 2 | 217 | 1106 | 0 | 0 | 0 | 0 | 0 | 0 | 0 | 0 | 0 |
| cg00694040 | 3 | 15 | 190 | 1118 | 0 | 0 | 0 | 0 | 0 | 0 | 0 | 0 |
| cg01016092 | 3 | 257 | 1 | 1065 | 0 | 0 | 0 | 0 | 0 | 0 | 0 | 0 |
| cg03721887 | 3 | 207 | 7 | 1109 | 0 | 0 | 0 | 0 | 0 | 0 | 0 | 0 |
| cg05416337 | 3 | 151 | 1169 | 3 | 0 | 0 | 0 | 0 | 0 | 0 | 0 | 0 |
| cg05779523 | 3 | 18 | 165 | 1140 | 0 | 0 | 0 | 0 | 0 | 0 | 0 | 0 |
| cg06493806 | 4 | 24 | 2 | 272 | 1025 | 0 | 0 | 0 | 0 | 0 | 0 | 0 |
| cg07258983 | 5 | 159 | 1 | 1 | 524 | 638 | 0 | 0 | 0 | 0 | 0 | 0 |
| cg07495405 | 2 | 100 | 1223 | 0 | 0 | 0 | 0 | 0 | 0 | 0 | 0 | 0 |
| cg09636756 | 5 | 109 | 1 | 2 | 442 | 769 | 0 | 0 | 0 | 0 | 0 | 0 |
| cg11986743 | 2 | 133 | 1190 | 0 | 0 | 0 | 0 | 0 | 0 | 0 | 0 | 0 |
| cg12259892 | 3 | 253 | 1 | 1069 | 0 | 0 | 0 | 0 | 0 | 0 | 0 | 0 |
| cg13103259 | 3 | 65 | 77 | 1181 | 0 | 0 | 0 | 0 | 0 | 0 | 0 | 0 |
| cg14123034 | 2 | 133 | 1190 | 0 | 0 | 0 | 0 | 0 | 0 | 0 | 0 | 0 |
| cg14141786 | 3 | 8 | 135 | 1180 | 0 | 0 | 0 | 0 | 0 | 0 | 0 | 0 |
| cg14408831 | 5 | 40 | 1 | 1 | 306 | 975 | 0 | 0 | 0 | 0 | 0 | 0 |
| cg15129815 | 3 | 62 | 396 | 865 | 0 | 0 | 0 | 0 | 0 | 0 | 0 | 0 |
| cg16061099 | 2 | 68 | 1255 | 0 | 0 | 0 | 0 | 0 | 0 | 0 | 0 | 0 |
| cg18121684 | 4 | 3 | 69 | 4 | 1247 | 0 | 0 | 0 | 0 | 0 | 0 | 0 |
| cg18444757 | 5 | 270 | 1 | 1 | 4 | 1047 | 0 | 0 | 0 | 0 | 0 | 0 |
| cg18971282 | 3 | 67 | 222 | 1034 | 0 | 0 | 0 | 0 | 0 | 0 | 0 | 0 |
| cg20218571 | 4 | 24 | 294 | 1 | 1004 | 0 | 0 | 0 | 0 | 0 | 0 | 0 |
| cg21367586 | 4 | 51 | 1 | 323 | 948 | 0 | 0 | 0 | 0 | 0 | 0 | 0 |
| cg23936477 | 4 | 101 | 1 | 457 | 764 | 0 | 0 | 0 | 0 | 0 | 0 | 0 |
| cg24883219 | 2 | 404 | 919 | 0 | 0 | 0 | 0 | 0 | 0 | 0 | 0 | 0 |
| cg25130590 | 2 | 69 | 1254 | 0 | 0 | 0 | 0 | 0 | 0 | 0 | 0 | 0 |
| cg27353962 | 3 | 10 | 97 | 1216 | 0 | 0 | 0 | 0 | 0 | 0 | 0 | 0 |
| cg00506198 | 5 | 2 | 1 | 1 | 135 | 1184 | 0 | 0 | 0 | 0 | 0 | 0 |
| cg00869309 | 2 | 120 | 1203 | 0 | 0 | 0 | 0 | 0 | 0 | 0 | 0 | 0 |
| cg01282508 | 3 | 21 | 251 | 1051 | 0 | 0 | 0 | 0 | 0 | 0 | 0 | 0 |
| cg01454153 | 3 | 9 | 134 | 1180 | 0 | 0 | 0 | 0 | 0 | 0 | 0 | 0 |
| cg01462799 | 3 | 248 | 655 | 420 | 0 | 0 | 0 | 0 | 0 | 0 | 0 | 0 |
| cg01512132 | 3 | 3 | 85 | 1235 | 0 | 0 | 0 | 0 | 0 | 0 | 0 | 0 |
| cg01567113 | 2 | 572 | 751 | 0 | 0 | 0 | 0 | 0 | 0 | 0 | 0 | 0 |
| cg01800061 | 4 | 5 | 1 | 100 | 1217 | 0 | 0 | 0 | 0 | 0 | 0 | 0 |
| cg02100397 | 3 | 226 | 570 | 527 | 0 | 0 | 0 | 0 | 0 | 0 | 0 | 0 |
| cg02305164 | 4 | 21 | 206 | 1 | 1095 | 0 | 0 | 0 | 0 | 0 | 0 | 0 |
| cg02345758 | 3 | 10 | 124 | 1189 | 0 | 0 | 0 | 0 | 0 | 0 | 0 | 0 |
| cg02890235 | 2 | 261 | 1062 | 0 | 0 | 0 | 0 | 0 | 0 | 0 | 0 | 0 |
| cg03020379 | 3 | 120 | 1 | 1202 | 0 | 0 | 0 | 0 | 0 | 0 | 0 | 0 |
| cg03101345 | 4 | 24 | 2 | 147 | 1150 | 0 | 0 | 0 | 0 | 0 | 0 | 0 |
| cg03112518 | 2 | 89 | 1234 | 0 | 0 | 0 | 0 | 0 | 0 | 0 | 0 | 0 |
| cg03162251 | 3 | 244 | 1 | 1078 | 0 | 0 | 0 | 0 | 0 | 0 | 0 | 0 |
| cg04388792 | 4 | 287 | 1 | 8 | 1027 | 0 | 0 | 0 | 0 | 0 | 0 | 0 |
| cg04576491 | 4 | 4 | 88 | 1 | 1230 | 0 | 0 | 0 | 0 | 0 | 0 | 0 |
| cg04610028 | 6 | 179 | 1 | 1 | 566 | 1 | 575 | 0 | 0 | 0 | 0 | 0 |
| cg04613452 | 4 | 1 | 73 | 1 | 1248 | 0 | 0 | 0 | 0 | 0 | 0 | 0 |
| cg04657146 | 3 | 767 | 458 | 98 | 0 | 0 | 0 | 0 | 0 | 0 | 0 | 0 |
| cg04864083 | 3 | 11 | 118 | 1194 | 0 | 0 | 0 | 0 | 0 | 0 | 0 | 0 |
| cg05900567 | 3 | 408 | 2 | 913 | 0 | 0 | 0 | 0 | 0 | 0 | 0 | 0 |
| cg06210526 | 5 | 9 | 2 | 138 | 4 | 1170 | 0 | 0 | 0 | 0 | 0 | 0 |
| cg06417478 | 4 | 767 | 1 | 458 | 97 | 0 | 0 | 0 | 0 | 0 | 0 | 0 |
| cg06590444 | 4 | 63 | 1 | 350 | 909 | 0 | 0 | 0 | 0 | 0 | 0 | 0 |
| cg06684911 | 3 | 135 | 6 | 1182 | 0 | 0 | 0 | 0 | 0 | 0 | 0 | 0 |
| cg07848485 | 3 | 27 | 190 | 1106 | 0 | 0 | 0 | 0 | 0 | 0 | 0 | 0 |
| cg07851262 | 4 | 26 | 256 | 1 | 1040 | 0 | 0 | 0 | 0 | 0 | 0 | 0 |
| cg08429705 | 5 | 261 | 1 | 1 | 573 | 487 | 0 | 0 | 0 | 0 | 0 | 0 |
| cg08436396 | 2 | 101 | 1222 | 0 | 0 | 0 | 0 | 0 | 0 | 0 | 0 | 0 |
| cg08879390 | 3 | 11 | 101 | 1211 | 0 | 0 | 0 | 0 | 0 | 0 | 0 | 0 |
| cg09925682 | 3 | 145 | 1 | 1177 | 0 | 0 | 0 | 0 | 0 | 0 | 0 | 0 |
| cg10148932 | 3 | 36 | 327 | 960 | 0 | 0 | 0 | 0 | 0 | 0 | 0 | 0 |
| cg10771931 | 5 | 161 | 1 | 427 | 1 | 733 | 0 | 0 | 0 | 0 | 0 | 0 |
| cg10776061 | 3 | 283 | 528 | 512 | 0 | 0 | 0 | 0 | 0 | 0 | 0 | 0 |
| cg11051221 | 4 | 2 | 79 | 1 | 1241 | 0 | 0 | 0 | 0 | 0 | 0 | 0 |
| cg11381655 | 3 | 9 | 116 | 1198 | 0 | 0 | 0 | 0 | 0 | 0 | 0 | 0 |
| cg11594160 | 3 | 4 | 72 | 1247 | 0 | 0 | 0 | 0 | 0 | 0 | 0 | 0 |
| cg11699126 | 4 | 15 | 1 | 83 | 1224 | 0 | 0 | 0 | 0 | 0 | 0 | 0 |
| cg11942206 | 2 | 228 | 1095 | 0 | 0 | 0 | 0 | 0 | 0 | 0 | 0 | 0 |
| cg12111149 | 6 | 58 | 1 | 1 | 386 | 1 | 876 | 0 | 0 | 0 | 0 | 0 |
| cg12155450 | 3 | 126 | 439 | 758 | 0 | 0 | 0 | 0 | 0 | 0 | 0 | 0 |
| cg12566301 | 3 | 93 | 1 | 1229 | 0 | 0 | 0 | 0 | 0 | 0 | 0 | 0 |
| cg13509147 | 3 | 1179 | 128 | 16 | 0 | 0 | 0 | 0 | 0 | 0 | 0 | 0 |
| cg13524588 | 3 | 6 | 157 | 1160 | 0 | 0 | 0 | 0 | 0 | 0 | 0 | 0 |
| cg13553703 | 4 | 72 | 1 | 239 | 1011 | 0 | 0 | 0 | 0 | 0 | 0 | 0 |
| cg13573375 | 4 | 634 | 2 | 1 | 686 | 0 | 0 | 0 | 0 | 0 | 0 | 0 |
| cg13829625 | 3 | 13 | 177 | 1133 | 0 | 0 | 0 | 0 | 0 | 0 | 0 | 0 |
| cg13935315 | 5 | 153 | 1 | 1 | 622 | 546 | 0 | 0 | 0 | 0 | 0 | 0 |
| cg14117565 | 3 | 19 | 256 | 1048 | 0 | 0 | 0 | 0 | 0 | 0 | 0 | 0 |
| cg14245898 | 3 | 79 | 1 | 1243 | 0 | 0 | 0 | 0 | 0 | 0 | 0 | 0 |
| cg14444099 | 5 | 67 | 1 | 1 | 1253 | 1 | 0 | 0 | 0 | 0 | 0 | 0 |
| cg14714057 | 3 | 3 | 86 | 1234 | 0 | 0 | 0 | 0 | 0 | 0 | 0 | 0 |
| cg15183649 | 3 | 3 | 66 | 1254 | 0 | 0 | 0 | 0 | 0 | 0 | 0 | 0 |
| cg15460046 | 3 | 4 | 90 | 1229 | 0 | 0 | 0 | 0 | 0 | 0 | 0 | 0 |
| cg15793258 | 3 | 2 | 92 | 1229 | 0 | 0 | 0 | 0 | 0 | 0 | 0 | 0 |
| cg15975960 | 4 | 37 | 1 | 327 | 958 | 0 | 0 | 0 | 0 | 0 | 0 | 0 |
| cg16034991 | 2 | 140 | 1183 | 0 | 0 | 0 | 0 | 0 | 0 | 0 | 0 | 0 |
| cg16569309 | 6 | 19 | 1 | 1 | 278 | 1 | 1023 | 0 | 0 | 0 | 0 | 0 |
| cg17323604 | 3 | 5 | 84 | 1234 | 0 | 0 | 0 | 0 | 0 | 0 | 0 | 0 |
| cg17671604 | 2 | 132 | 1191 | 0 | 0 | 0 | 0 | 0 | 0 | 0 | 0 | 0 |
| cg17830140 | 5 | 14 | 1 | 183 | 3 | 1122 | 0 | 0 | 0 | 0 | 0 | 0 |
| cg18618432 | 2 | 90 | 1233 | 0 | 0 | 0 | 0 | 0 | 0 | 0 | 0 | 0 |
| cg18624102 | 4 | 307 | 2 | 641 | 373 | 0 | 0 | 0 | 0 | 0 | 0 | 0 |
| cg18628853 | 3 | 66 | 1 | 1256 | 0 | 0 | 0 | 0 | 0 | 0 | 0 | 0 |
| cg19225953 | 2 | 117 | 1206 | 0 | 0 | 0 | 0 | 0 | 0 | 0 | 0 | 0 |
| cg19360425 | 3 | 9 | 121 | 1193 | 0 | 0 | 0 | 0 | 0 | 0 | 0 | 0 |
| cg19446264 | 3 | 5 | 77 | 1241 | 0 | 0 | 0 | 0 | 0 | 0 | 0 | 0 |
| cg19504860 | 5 | 1065 | 2 | 226 | 1 | 29 | 0 | 0 | 0 | 0 | 0 | 0 |
| cg19564375 | 4 | 21 | 1 | 205 | 1096 | 0 | 0 | 0 | 0 | 0 | 0 | 0 |
| cg19736040 | 4 | 6 | 102 | 1 | 1214 | 0 | 0 | 0 | 0 | 0 | 0 | 0 |
| cg20218135 | 2 | 250 | 1073 | 0 | 0 | 0 | 0 | 0 | 0 | 0 | 0 | 0 |
| cg20231299 | 4 | 41 | 1 | 375 | 906 | 0 | 0 | 0 | 0 | 0 | 0 | 0 |
| cg21414902 | 3 | 17 | 114 | 1192 | 0 | 0 | 0 | 0 | 0 | 0 | 0 | 0 |
| cg21653913 | 2 | 138 | 1185 | 0 | 0 | 0 | 0 | 0 | 0 | 0 | 0 | 0 |
| cg22316634 | 2 | 102 | 1221 | 0 | 0 | 0 | 0 | 0 | 0 | 0 | 0 | 0 |
| cg22336867 | 4 | 567 | 520 | 1 | 235 | 0 | 0 | 0 | 0 | 0 | 0 | 0 |
| cg22508145 | 3 | 224 | 537 | 562 | 0 | 0 | 0 | 0 | 0 | 0 | 0 | 0 |
| cg22671798 | 4 | 534 | 1 | 532 | 256 | 0 | 0 | 0 | 0 | 0 | 0 | 0 |
| cg22781764 | 5 | 24 | 1 | 252 | 1 | 1045 | 0 | 0 | 0 | 0 | 0 | 0 |
| cg22798261 | 3 | 114 | 1208 | 1 | 0 | 0 | 0 | 0 | 0 | 0 | 0 | 0 |
| cg22996768 | 3 | 785 | 399 | 139 | 0 | 0 | 0 | 0 | 0 | 0 | 0 | 0 |
| cg23499373 | 3 | 166 | 568 | 589 | 0 | 0 | 0 | 0 | 0 | 0 | 0 | 0 |
| cg23803868 | 4 | 171 | 570 | 1 | 581 | 0 | 0 | 0 | 0 | 0 | 0 | 0 |
| cg24077401 | 2 | 127 | 1196 | 0 | 0 | 0 | 0 | 0 | 0 | 0 | 0 | 0 |
| cg24401199 | 5 | 33 | 1 | 1 | 330 | 958 | 0 | 0 | 0 | 0 | 0 | 0 |
| cg24419602 | 2 | 107 | 1216 | 0 | 0 | 0 | 0 | 0 | 0 | 0 | 0 | 0 |
| cg24747107 | 3 | 45 | 213 | 1065 | 0 | 0 | 0 | 0 | 0 | 0 | 0 | 0 |
| cg24813710 | 4 | 3 | 101 | 2 | 1217 | 0 | 0 | 0 | 0 | 0 | 0 | 0 |
| cg24863815 | 3 | 1146 | 3 | 174 | 0 | 0 | 0 | 0 | 0 | 0 | 0 | 0 |
| cg25432425 | 4 | 6 | 1 | 76 | 1240 | 0 | 0 | 0 | 0 | 0 | 0 | 0 |
| cg26264314 | 5 | 1 | 130 | 3 | 1 | 1188 | 0 | 0 | 0 | 0 | 0 | 0 |
| cg26548410 | 2 | 148 | 1175 | 0 | 0 | 0 | 0 | 0 | 0 | 0 | 0 | 0 |
| cg26642774 | 2 | 91 | 1232 | 0 | 0 | 0 | 0 | 0 | 0 | 0 | 0 | 0 |
| cg26727538 | 4 | 4 | 123 | 1195 | 1 | 0 | 0 | 0 | 0 | 0 | 0 | 0 |
| cg27288829 | 2 | 475 | 848 | 0 | 0 | 0 | 0 | 0 | 0 | 0 | 0 | 0 |
| cg27322675 | 4 | 1 | 1 | 73 | 1248 | 0 | 0 | 0 | 0 | 0 | 0 | 0 |
| cg27581706 | 3 | 2 | 88 | 1233 | 0 | 0 | 0 | 0 | 0 | 0 | 0 | 0 |
| cg00264578 | 4 | 16 | 1 | 170 | 1136 | 0 | 0 | 0 | 0 | 0 | 0 | 0 |
| cg00704664 | 3 | 380 | 521 | 422 | 0 | 0 | 0 | 0 | 0 | 0 | 0 | 0 |
| cg01236565 | 2 | 186 | 1137 | 0 | 0 | 0 | 0 | 0 | 0 | 0 | 0 | 0 |
| cg01326421 | 2 | 242 | 1081 | 0 | 0 | 0 | 0 | 0 | 0 | 0 | 0 | 0 |
| cg01471923 | 5 | 114 | 2 | 1 | 2 | 1204 | 0 | 0 | 0 | 0 | 0 | 0 |
| cg01861555 | 4 | 87 | 1 | 2 | 1233 | 0 | 0 | 0 | 0 | 0 | 0 | 0 |
| cg02239586 | 3 | 2 | 129 | 1192 | 0 | 0 | 0 | 0 | 0 | 0 | 0 | 0 |
| cg02288165 | 3 | 1 | 131 | 1191 | 0 | 0 | 0 | 0 | 0 | 0 | 0 | 0 |
| cg02506728 | 4 | 1214 | 1 | 101 | 7 | 0 | 0 | 0 | 0 | 0 | 0 | 0 |
| cg03114748 | 4 | 6 | 69 | 3 | 1245 | 0 | 0 | 0 | 0 | 0 | 0 | 0 |
| cg03279219 | 3 | 175 | 4 | 1144 | 0 | 0 | 0 | 0 | 0 | 0 | 0 | 0 |
| cg03375067 | 4 | 3 | 1 | 78 | 1241 | 0 | 0 | 0 | 0 | 0 | 0 | 0 |
| cg03467235 | 5 | 72 | 2 | 1 | 297 | 951 | 0 | 0 | 0 | 0 | 0 | 0 |
| cg03606258 | 2 | 1224 | 99 | 0 | 0 | 0 | 0 | 0 | 0 | 0 | 0 | 0 |
| cg03734225 | 3 | 2 | 65 | 1256 | 0 | 0 | 0 | 0 | 0 | 0 | 0 | 0 |
| cg04990378 | 5 | 100 | 1 | 453 | 1 | 768 | 0 | 0 | 0 | 0 | 0 | 0 |
| cg05146395 | 2 | 77 | 1246 | 0 | 0 | 0 | 0 | 0 | 0 | 0 | 0 | 0 |
| cg05840533 | 2 | 140 | 1183 | 0 | 0 | 0 | 0 | 0 | 0 | 0 | 0 | 0 |
| cg05867245 | 3 | 115 | 470 | 738 | 0 | 0 | 0 | 0 | 0 | 0 | 0 | 0 |
| cg06305758 | 3 | 105 | 3 | 1215 | 0 | 0 | 0 | 0 | 0 | 0 | 0 | 0 |
| cg07052950 | 4 | 65 | 1 | 1 | 1256 | 0 | 0 | 0 | 0 | 0 | 0 | 0 |
| cg07091478 | 4 | 111 | 1 | 1 | 1210 | 0 | 0 | 0 | 0 | 0 | 0 | 0 |
| cg07545427 | 4 | 3 | 1 | 88 | 1231 | 0 | 0 | 0 | 0 | 0 | 0 | 0 |
| cg08331829 | 3 | 254 | 613 | 456 | 0 | 0 | 0 | 0 | 0 | 0 | 0 | 0 |
| cg08657228 | 2 | 465 | 858 | 0 | 0 | 0 | 0 | 0 | 0 | 0 | 0 | 0 |
| cg09182085 | 3 | 111 | 1 | 1211 | 0 | 0 | 0 | 0 | 0 | 0 | 0 | 0 |
| cg09595245 | 4 | 5 | 1 | 68 | 1249 | 0 | 0 | 0 | 0 | 0 | 0 | 0 |
| cg09845806 | 2 | 83 | 1240 | 0 | 0 | 0 | 0 | 0 | 0 | 0 | 0 | 0 |
| cg09864671 | 4 | 86 | 2 | 1232 | 3 | 0 | 0 | 0 | 0 | 0 | 0 | 0 |
| cg11366696 | 3 | 5 | 80 | 1238 | 0 | 0 | 0 | 0 | 0 | 0 | 0 | 0 |
| cg11897887 | 4 | 194 | 1 | 547 | 581 | 0 | 0 | 0 | 0 | 0 | 0 | 0 |
| cg13301327 | 4 | 85 | 357 | 1 | 880 | 0 | 0 | 0 | 0 | 0 | 0 | 0 |
| cg13696012 | 4 | 9 | 1 | 109 | 1204 | 0 | 0 | 0 | 0 | 0 | 0 | 0 |
| cg14324838 | 3 | 1 | 74 | 1248 | 0 | 0 | 0 | 0 | 0 | 0 | 0 | 0 |
| cg14943816 | 2 | 91 | 1232 | 0 | 0 | 0 | 0 | 0 | 0 | 0 | 0 | 0 |
| cg15550113 | 3 | 2 | 142 | 1179 | 0 | 0 | 0 | 0 | 0 | 0 | 0 | 0 |
| cg15557840 | 3 | 72 | 5 | 1246 | 0 | 0 | 0 | 0 | 0 | 0 | 0 | 0 |
| cg15834841 | 3 | 1 | 99 | 1223 | 0 | 0 | 0 | 0 | 0 | 0 | 0 | 0 |
| cg16844425 | 3 | 20 | 157 | 1146 | 0 | 0 | 0 | 0 | 0 | 0 | 0 | 0 |
| cg17341969 | 4 | 129 | 2 | 540 | 652 | 0 | 0 | 0 | 0 | 0 | 0 | 0 |
| cg17343385 | 3 | 9 | 151 | 1163 | 0 | 0 | 0 | 0 | 0 | 0 | 0 | 0 |
| cg17661798 | 3 | 171 | 557 | 595 | 0 | 0 | 0 | 0 | 0 | 0 | 0 | 0 |
| cg17811452 | 2 | 254 | 1069 | 0 | 0 | 0 | 0 | 0 | 0 | 0 | 0 | 0 |
| cg17884856 | 4 | 2 | 1 | 80 | 1240 | 0 | 0 | 0 | 0 | 0 | 0 | 0 |
| cg18032705 | 4 | 23 | 251 | 2 | 1047 | 0 | 0 | 0 | 0 | 0 | 0 | 0 |
| cg18674961 | 2 | 660 | 663 | 0 | 0 | 0 | 0 | 0 | 0 | 0 | 0 | 0 |
| cg18819889 | 5 | 2 | 1 | 71 | 4 | 1245 | 0 | 0 | 0 | 0 | 0 | 0 |
| cg19497388 | 3 | 4 | 88 | 1231 | 0 | 0 | 0 | 0 | 0 | 0 | 0 | 0 |
| cg20388329 | 3 | 6 | 143 | 1174 | 0 | 0 | 0 | 0 | 0 | 0 | 0 | 0 |
| cg20592836 | 5 | 656 | 1 | 522 | 1 | 143 | 0 | 0 | 0 | 0 | 0 | 0 |
| cg21130926 | 4 | 212 | 634 | 2 | 475 | 0 | 0 | 0 | 0 | 0 | 0 | 0 |
| cg22797354 | 5 | 1 | 1 | 69 | 1 | 1251 | 0 | 0 | 0 | 0 | 0 | 0 |
| cg23629496 | 3 | 4 | 216 | 1103 | 0 | 0 | 0 | 0 | 0 | 0 | 0 | 0 |
| cg24859648 | 3 | 618 | 453 | 252 | 0 | 0 | 0 | 0 | 0 | 0 | 0 | 0 |
| cg26089705 | 3 | 143 | 1 | 1179 | 0 | 0 | 0 | 0 | 0 | 0 | 0 | 0 |
| cg26365090 | 4 | 316 | 1 | 521 | 485 | 0 | 0 | 0 | 0 | 0 | 0 | 0 |
| cg00159953 | 4 | 1 | 3 | 134 | 1185 | 0 | 0 | 0 | 0 | 0 | 0 | 0 |
| cg00272795 | 4 | 35 | 2 | 353 | 933 | 0 | 0 | 0 | 0 | 0 | 0 | 0 |
| cg00762003 | 4 | 2 | 303 | 1 | 1017 | 0 | 0 | 0 | 0 | 0 | 0 | 0 |
| cg01199931 | 3 | 35 | 192 | 1096 | 0 | 0 | 0 | 0 | 0 | 0 | 0 | 0 |
| cg02188142 | 3 | 86 | 3 | 1234 | 0 | 0 | 0 | 0 | 0 | 0 | 0 | 0 |
| cg02464073 | 5 | 358 | 1 | 589 | 1 | 374 | 0 | 0 | 0 | 0 | 0 | 0 |
| cg03706056 | 2 | 1075 | 248 | 0 | 0 | 0 | 0 | 0 | 0 | 0 | 0 | 0 |
| cg04226110 | 3 | 108 | 1 | 1214 | 0 | 0 | 0 | 0 | 0 | 0 | 0 | 0 |
| cg05809586 | 5 | 154 | 1 | 1 | 506 | 661 | 0 | 0 | 0 | 0 | 0 | 0 |
| cg06212876 | 4 | 8 | 1 | 132 | 1182 | 0 | 0 | 0 | 0 | 0 | 0 | 0 |
| cg08407901 | 4 | 484 | 548 | 1 | 290 | 0 | 0 | 0 | 0 | 0 | 0 | 0 |
| cg08514194 | 2 | 399 | 924 | 0 | 0 | 0 | 0 | 0 | 0 | 0 | 0 | 0 |
| cg08880261 | 3 | 174 | 565 | 584 | 0 | 0 | 0 | 0 | 0 | 0 | 0 | 0 |
| cg09026600 | 5 | 8 | 6 | 106 | 1 | 1202 | 0 | 0 | 0 | 0 | 0 | 0 |
| cg11401796 | 5 | 36 | 1 | 1 | 349 | 936 | 0 | 0 | 0 | 0 | 0 | 0 |
| cg13974865 | 3 | 9 | 98 | 1216 | 0 | 0 | 0 | 0 | 0 | 0 | 0 | 0 |
| cg17839758 | 2 | 409 | 914 | 0 | 0 | 0 | 0 | 0 | 0 | 0 | 0 | 0 |
| cg20349024 | 3 | 1197 | 122 | 4 | 0 | 0 | 0 | 0 | 0 | 0 | 0 | 0 |
| cg22373097 | 5 | 68 | 2 | 3 | 2 | 1248 | 0 | 0 | 0 | 0 | 0 | 0 |
| cg26386968 | 4 | 11 | 85 | 1226 | 1 | 0 | 0 | 0 | 0 | 0 | 0 | 0 |
| cg26790209 | 4 | 2 | 96 | 1 | 1224 | 0 | 0 | 0 | 0 | 0 | 0 | 0 |
| cg00243527 | 4 | 120 | 418 | 1 | 784 | 0 | 0 | 0 | 0 | 0 | 0 | 0 |
| cg00408876 | 4 | 12 | 2 | 129 | 1180 | 0 | 0 | 0 | 0 | 0 | 0 | 0 |
| cg00740510 | 2 | 140 | 1183 | 0 | 0 | 0 | 0 | 0 | 0 | 0 | 0 | 0 |
| cg00944631 | 5 | 309 | 1 | 2 | 1 | 1010 | 0 | 0 | 0 | 0 | 0 | 0 |
| cg01529207 | 4 | 17 | 1 | 94 | 1211 | 0 | 0 | 0 | 0 | 0 | 0 | 0 |
| cg01744396 | 2 | 1254 | 69 | 0 | 0 | 0 | 0 | 0 | 0 | 0 | 0 | 0 |
| cg02940070 | 4 | 63 | 1 | 344 | 915 | 0 | 0 | 0 | 0 | 0 | 0 | 0 |
| cg02975187 | 3 | 3 | 73 | 1247 | 0 | 0 | 0 | 0 | 0 | 0 | 0 | 0 |
| cg03873518 | 2 | 281 | 1042 | 0 | 0 | 0 | 0 | 0 | 0 | 0 | 0 | 0 |
| cg04234412 | 3 | 998 | 1 | 324 | 0 | 0 | 0 | 0 | 0 | 0 | 0 | 0 |
| cg05338731 | 5 | 610 | 538 | 1 | 1 | 173 | 0 | 0 | 0 | 0 | 0 | 0 |
| cg06803201 | 3 | 1236 | 79 | 8 | 0 | 0 | 0 | 0 | 0 | 0 | 0 | 0 |
| cg07244980 | 3 | 256 | 1065 | 2 | 0 | 0 | 0 | 0 | 0 | 0 | 0 | 0 |
| cg07397612 | 4 | 4 | 85 | 1 | 1233 | 0 | 0 | 0 | 0 | 0 | 0 | 0 |
| cg07422735 | 4 | 12 | 1 | 172 | 1138 | 0 | 0 | 0 | 0 | 0 | 0 | 0 |
| cg11019791 | 3 | 193 | 528 | 602 | 0 | 0 | 0 | 0 | 0 | 0 | 0 | 0 |
| cg11466708 | 3 | 22 | 226 | 1075 | 0 | 0 | 0 | 0 | 0 | 0 | 0 | 0 |
| cg12363840 | 4 | 69 | 1 | 1252 | 1 | 0 | 0 | 0 | 0 | 0 | 0 | 0 |
| cg16122736 | 2 | 204 | 1119 | 0 | 0 | 0 | 0 | 0 | 0 | 0 | 0 | 0 |
| cg17205386 | 3 | 21 | 249 | 1053 | 0 | 0 | 0 | 0 | 0 | 0 | 0 | 0 |
| cg17662493 | 4 | 388 | 1 | 592 | 342 | 0 | 0 | 0 | 0 | 0 | 0 | 0 |
| cg18455772 | 3 | 107 | 2 | 1214 | 0 | 0 | 0 | 0 | 0 | 0 | 0 | 0 |
| cg18514595 | 4 | 507 | 2 | 589 | 225 | 0 | 0 | 0 | 0 | 0 | 0 | 0 |
| cg18761958 | 4 | 3 | 102 | 1217 | 1 | 0 | 0 | 0 | 0 | 0 | 0 | 0 |
| cg19021236 | 3 | 120 | 3 | 1200 | 0 | 0 | 0 | 0 | 0 | 0 | 0 | 0 |
| cg19470385 | 2 | 92 | 1231 | 0 | 0 | 0 | 0 | 0 | 0 | 0 | 0 | 0 |
| cg19707653 | 4 | 116 | 3 | 1203 | 1 | 0 | 0 | 0 | 0 | 0 | 0 | 0 |
| cg19800640 | 3 | 23 | 187 | 1113 | 0 | 0 | 0 | 0 | 0 | 0 | 0 | 0 |
| cg20014942 | 4 | 14 | 1 | 203 | 1105 | 0 | 0 | 0 | 0 | 0 | 0 | 0 |
| cg21445911 | 3 | 2 | 99 | 1222 | 0 | 0 | 0 | 0 | 0 | 0 | 0 | 0 |
| cg24048283 | 5 | 1 | 2 | 108 | 1 | 1211 | 0 | 0 | 0 | 0 | 0 | 0 |
| cg24694833 | 4 | 97 | 1 | 521 | 704 | 0 | 0 | 0 | 0 | 0 | 0 | 0 |
| cg25059408 | 4 | 13 | 1 | 139 | 1170 | 0 | 0 | 0 | 0 | 0 | 0 | 0 |
| cg25599641 | 3 | 7 | 85 | 1231 | 0 | 0 | 0 | 0 | 0 | 0 | 0 | 0 |
| cg26822175 | 4 | 3 | 114 | 1 | 1205 | 0 | 0 | 0 | 0 | 0 | 0 | 0 |
| cg27179498 | 4 | 5 | 1 | 175 | 1142 | 0 | 0 | 0 | 0 | 0 | 0 | 0 |
| cg27376941 | 5 | 25 | 1 | 2 | 206 | 1089 | 0 | 0 | 0 | 0 | 0 | 0 |
| cg27415324 | 2 | 74 | 1249 | 0 | 0 | 0 | 0 | 0 | 0 | 0 | 0 | 0 |
