# Supplementary material for: Gaps in current methods to detect polymorphic CpGs from Illumina Infinium human methylation microarrays and exploring their potential impact in multi-EWAS analyses
Source: Epigenetics. 2023 Nov 20;18(1):2281153. doi: 10.1080/15592294.2023.2281153 (PMC10732615; doi:10.1080/15592294.2023.2281153)
Supplement: Supplementary_File_1.docx [file KEPI_A_2281153_SM9088.docx]

| id | Age | Ethnicity | Sex | Tissue |
| --- | --- | --- | --- | --- |
| GSM2808247_2808247 | 6.4 | Chinese | M | Blood |
| GSM2808245_2808245 | 6.7 | Chinese | F | Blood |
| GSM2808253_2808253 | 7 | Chinese | M | Blood |
| GSM2808250_2808250 | 7.1 | Chinese | M | Blood |
| GSM2808241_2808241 | 7.2 | Chinese | F | Blood |
| GSM2808255_2808255 | 7.2 | Chinese | M | Blood |
| GSM2808249_2808249 | 7.3 | Chinese | F | Blood |
| GSM2808280_2808280 | 7.3 | Chinese | M | Blood |
| GSM2808266_2808266 | 7.4 | Chinese | F | Blood |
| GSM2808263_2808263 | 7.5 | Chinese | M | Blood |
| GSM2808273_2808273 | 7.5 | Chinese | F | Blood |
| GSM2808285_2808285 | 7.6 | Chinese | M | Blood |
| GSM2808246_2808246 | 7.7 | Chinese | F | Blood |
| GSM2808257_2808257 | 7.7 | Chinese | M | Blood |
| GSM2808269_2808269 | 7.9 | Chinese | M | Blood |
| GSM2808277_2808277 | 7.9 | Chinese | F | Blood |
| GSM2808244_2808244 | 8 | Chinese | F | Blood |
| GSM2808275_2808275 | 8.1 | Chinese | M | Blood |
| GSM2808261_2808261 | 8.2 | Chinese | M | Blood |
| GSM2808282_2808282 | 8.2 | Chinese | F | Blood |
| GSM2808239_2808239 | 8.3 | Chinese | M | Blood |
| GSM2808276_2808276 | 8.7 | Chinese | M | Blood |
| GSM2808262_2808262 | 8.9 | Chinese | M | Blood |
| GSM2808258_2808258 | 9 | Chinese | F | Blood |
| GSM2808279_2808279 | 9 | Chinese | M | Blood |
| GSM2808281_2808281 | 9 | Chinese | M | Blood |
| GSM2808243_2808243 | 9.1 | Chinese | F | Blood |
| GSM2808254_2808254 | 9.1 | Chinese | F | Blood |
| GSM2808271_2808271 | 9.1 | Chinese | M | Blood |
| GSM2808284_2808284 | 9.1 | Chinese | F | Blood |
| GSM2808251_2808251 | 9.4 | Chinese | M | Blood |
| GSM2808272_2808272 | 9.5 | Chinese | M | Blood |
| GSM2808264_2808264 | 9.6 | Chinese | F | Blood |
| GSM2808286_2808286 | 9.7 | Chinese | F | Blood |
| GSM2808274_2808274 | 9.8 | Chinese | M | Blood |
| GSM2808256_2808256 | 10.1 | Chinese | M | Blood |
| GSM2808268_2808268 | 10.1 | Chinese | F | Blood |
| GSM2808278_2808278 | 10.2 | Chinese | M | Blood |
| GSM2808270_2808270 | 10.5 | Chinese | F | Blood |
| GSM2808240_2808240 | 10.7 | Chinese | M | Blood |
| GSM2808267_2808267 | 10.7 | Chinese | M | Blood |
| GSM2808259_2808259 | 10.9 | Chinese | M | Blood |
| GSM2808242_2808242 | 11.1 | Chinese | M | Blood |
| GSM2808252_2808252 | 11.1 | Chinese | M | Blood |
| GSM2808283_2808283 | 11.2 | Chinese | F | Blood |
| GSM2808265_2808265 | 12.1 | Chinese | M | Blood |
| GSM2808260_2808260 | 13.3 | Chinese | F | Blood |
| GSM2808248_2808248 | 14.6 | Chinese | M | Blood |
| GSM6057846_6057846 | 17 | Han_Chinese | M | Blood |
| GSM6057852_6057852 | 17 | Han_Chinese | M | Blood |
| GSM6057847_6057847 | 19 | Han_Chinese | F | Blood |
| GSM6057854_6057854 | 19 | Han_Chinese | M | Blood |
| GSM3155944_3155944 | 20 | Hispanic | F | Blood |
| GSM3156474_3156474 | 20 | Hispanic | F | Blood |
| GSM6057865_6057865 | 20 | Han_Chinese | M | Blood |
| GSM6057878_6057878 | 20 | Han_Chinese | M | Blood |
| GSM1602324_1602324 | 21 | Chinese | F | Blood |
| GSM1602325_1602325 | 21 | Chinese | F | Blood |
| GSM3155931_3155931 | 21 | African | F | Blood |
| GSM3155939_3155939 | 21 | Hispanic | F | Blood |
| GSM3155948_3155948 | 21 | Hispanic | F | Blood |
| GSM3156414_3156414 | 21 | African | F | Blood |
| GSM3156422_3156422 | 21 | Hispanic | F | Blood |
| GSM3156475_3156475 | 21 | Hispanic | F | Blood |
| GSM6057849_6057849 | 21 | Han_Chinese | F | Blood |
| GSM6057866_6057866 | 21 | Han_Chinese | M | Blood |
| GSM6057881_6057881 | 21 | Han_Chinese | M | Blood |
| GSM6057883_6057883 | 21 | Han_Chinese | M | Blood |
| GSM1602322_1602322 | 22 | Chinese | F | Blood |
| GSM1602323_1602323 | 22 | Chinese | F | Blood |
| GSM1602330_1602330 | 22 | Chinese | F | Blood |
| GSM1602331_1602331 | 22 | Chinese | F | Blood |
| GSM3155933_3155933 | 22 | White | F | Blood |
| GSM3156186_3156186 | 22 | Hispanic | F | Blood |
| GSM3156416_3156416 | 22 | White | F | Blood |
| GSM3156481_3156481 | 22 | Hispanic | F | Blood |
| GSM6057813_6057813 | 22 | Han_Chinese | F | Blood |
| GSM6057855_6057855 | 22 | Han_Chinese | F | Blood |
| GSM6057877_6057877 | 22 | Han_Chinese | M | Blood |
| GSM6057858_6057858 | 23 | Han_Chinese | F | Blood |
| GSM6057860_6057860 | 23 | Han_Chinese | F | Blood |
| GSM6057863_6057863 | 23 | Han_Chinese | M | Blood |
| GSM6057864_6057864 | 23 | Han_Chinese | F | Blood |
| GSM6057867_6057867 | 23 | Han_Chinese | F | Blood |
| GSM6057874_6057874 | 23 | Han_Chinese | M | Blood |
| GSM6057887_6057887 | 23 | Han_Chinese | F | Blood |
| GSM6057890_6057890 | 23 | Han_Chinese | F | Blood |
| GSM3156181_3156181 | 24 | Hispanic | F | Blood |
| GSM3156190_3156190 | 24 | White | F | Blood |
| GSM3156194_3156194 | 24 | White | F | Blood |
| GSM3156478_3156478 | 24 | Hispanic | F | Blood |
| GSM3156483_3156483 | 24 | White | F | Blood |
| GSM3156485_3156485 | 24 | White | F | Blood |
| GSM6057815_6057815 | 24 | Han_Chinese | M | Blood |
| GSM6057861_6057861 | 24 | Han_Chinese | F | Blood |
| GSM6057868_6057868 | 24 | Han_Chinese | F | Blood |
| GSM6057870_6057870 | 24 | Han_Chinese | F | Blood |
| GSM6057882_6057882 | 24 | Han_Chinese | F | Blood |
| GSM6057888_6057888 | 24 | Han_Chinese | F | Blood |
| GSM1602328_1602328 | 25 | Chinese | F | Blood |
| GSM1602329_1602329 | 25 | Chinese | F | Blood |
| GSM3156408_3156408 | 25 | Hispanic | F | Blood |
| GSM3156488_3156488 | 25 | Hispanic | F | Blood |
| GSM6057818_6057818 | 25 | Han_Chinese | M | Blood |
| GSM6057820_6057820 | 25 | Han_Chinese | F | Blood |
| GSM6057835_6057835 | 25 | Han_Chinese | F | Blood |
| GSM6057851_6057851 | 25 | Han_Chinese | F | Blood |
| GSM6057872_6057872 | 25 | Han_Chinese | M | Blood |
| GSM6057884_6057884 | 25 | Han_Chinese | F | Blood |
| GSM6057886_6057886 | 25 | Han_Chinese | F | Blood |
| GSM1602316_1602316 | 26 | Chinese | F | Blood |
| GSM1602317_1602317 | 26 | Chinese | F | Blood |
| GSM3156179_3156179 | 26 | Hispanic | F | Blood |
| GSM3156188_3156188 | 26 | White | F | Blood |
| GSM3156477_3156477 | 26 | Hispanic | F | Blood |
| GSM3156482_3156482 | 26 | White | F | Blood |
| GSM6057853_6057853 | 26 | Han_Chinese | M | Blood |
| GSM6057857_6057857 | 26 | Han_Chinese | F | Blood |
| GSM6057869_6057869 | 26 | Han_Chinese | M | Blood |
| GSM6057880_6057880 | 26 | Han_Chinese | M | Blood |
| GSM6057876_6057876 | 27 | Han_Chinese | M | Blood |
| GSM6057885_6057885 | 27 | Han_Chinese | M | Blood |
| GSM6057889_6057889 | 27 | Han_Chinese | F | Blood |
| GSM3156410_3156410 | 28 | White | F | Blood |
| GSM3156489_3156489 | 28 | White | F | Blood |
| GSM6057824_6057824 | 28 | Han_Chinese | F | Blood |
| GSM6057836_6057836 | 28 | Han_Chinese | F | Blood |
| GSM6057871_6057871 | 28 | Han_Chinese | M | Blood |
| GSM6057875_6057875 | 28 | Han_Chinese | F | Blood |
| GSM6057879_6057879 | 28 | Han_Chinese | M | Blood |
| GSM1602318_1602318 | 30 | Chinese | F | Blood |
| GSM1602319_1602319 | 30 | Chinese | F | Blood |
| GSM6057819_6057819 | 30 | Han_Chinese | M | Blood |
| GSM3155941_3155941 | 31 | White | F | Blood |
| GSM3156406_3156406 | 31 | White | F | Blood |
| GSM3156473_3156473 | 31 | White | F | Blood |
| GSM3156487_3156487 | 31 | White | F | Blood |
| GSM1602320_1602320 | 32 | Chinese | F | Blood |
| GSM1602321_1602321 | 32 | Chinese | F | Blood |
| GSM1602326_1602326 | 32 | Chinese | F | Blood |
| GSM1602327_1602327 | 32 | Chinese | F | Blood |
| GSM3155946_3155946 | 32 | White | F | Blood |
| GSM6057821_6057821 | 32 | Han_Chinese | M | Blood |
| GSM3155928_3155928 | 33 | White | F | Blood |
| GSM3156183_3156183 | 33 | African | F | Blood |
| GSM3156404_3156404 | 33 | White | F | Blood |
| GSM3156412_3156412 | 33 | White | F | Blood |
| GSM3156479_3156479 | 33 | African | F | Blood |
| GSM3156486_3156486 | 33 | White | F | Blood |
| GSM6057817_6057817 | 33 | Han_Chinese | F | Blood |
| GSM3156184_3156184 | 34 | White | F | Blood |
| GSM3156480_3156480 | 34 | White | F | Blood |
| GSM6057833_6057833 | 34 | Han_Chinese | F | Blood |
| GSM6424777_6424777 | 34 | African_American | F | Blood |
| GSM3035660_3035660 | 35 | Caucasian | M | Blood |
| GSM3155934_3155934 | 35 | Hispanic | F | Blood |
| GSM3155937_3155937 | 35 | Hispanic | F | Blood |
| GSM3156418_3156418 | 35 | Hispanic | F | Blood |
| GSM3156420_3156420 | 35 | Hispanic | F | Blood |
| GSM6057862_6057862 | 35 | Han_Chinese | M | Blood |
| GSM3035502_3035502 | 36 | Hispanic | F | Blood |
| GSM3035584_3035584 | 36 | Hispanic | M | Blood |
| GSM3035665_3035665 | 36 | Caucasian | M | Blood |
| GSM6057816_6057816 | 36 | Han_Chinese | F | Blood |
| GSM6057850_6057850 | 36 | Han_Chinese | F | Blood |
| GSM3035426_3035426 | 37 | Caucasian | F | Blood |
| GSM3035497_3035497 | 37 | Hispanic | M | Blood |
| GSM3035635_3035635 | 37 | Caucasian | F | Blood |
| GSM6057831_6057831 | 37 | Han_Chinese | M | Blood |
| GSM6057859_6057859 | 37 | Han_Chinese | M | Blood |
| GSM6424785_6424785 | 38 | African_American | F | Blood |
| GSM6057856_6057856 | 39 | Han_Chinese | F | Blood |
| GSM6424821_6424821 | 39 | African_American | F | Blood |
| GSM3035586_3035586 | 40 | Caucasian | M | Blood |
| GSM3035671_3035671 | 40 | Caucasian | F | Blood |
| GSM6057848_6057848 | 40 | Han_Chinese | F | Blood |
| GSM3035423_3035423 | 41 | Hispanic | M | Blood |
| GSM3035492_3035492 | 41 | Caucasian | M | Blood |
| GSM3035504_3035504 | 41 | Caucasian | F | Blood |
| GSM3035647_3035647 | 41 | Caucasian | F | Blood |
| GSM3035428_3035428 | 42 | Caucasian | F | Blood |
| GSM3035585_3035585 | 42 | Hispanic | M | Blood |
| GSM3035649_3035649 | 42 | Caucasian | F | Blood |
| GSM3035668_3035668 | 42 | Caucasian | M | Blood |
| GSM3035669_3035669 | 42 | Hispanic | M | Blood |
| GSM6424781_6424781 | 42 | African_American | F | Blood |
| GSM6425125_6425125 | 42 | African_American | F | Blood |
| GSM6425144_6425144 | 42 | African_American | M | Blood |
| GSM3035424_3035424 | 43 | Caucasian | F | Blood |
| GSM3035429_3035429 | 43 | Caucasian | F | Blood |
| GSM3035536_3035536 | 43 | Caucasian | F | Blood |
| GSM3035637_3035637 | 43 | Caucasian | M | Blood |
| GSM6057811_6057811 | 43 | Han_Chinese | M | Blood |
| GSM6057827_6057827 | 43 | Han_Chinese | F | Blood |
| GSM6424793_6424793 | 43 | African_American | F | Blood |
| GSM3035537_3035537 | 44 | Caucasian | M | Blood |
| GSM3035639_3035639 | 44 | Hispanic | F | Blood |
| GSM3035661_3035661 | 44 | Caucasian | M | Blood |
| GSM6057814_6057814 | 44 | Han_Chinese | F | Blood |
| GSM6057845_6057845 | 44 | Han_Chinese | F | Blood |
| GSM6424816_6424816 | 44 | African_American | F | Blood |
| GSM3035494_3035494 | 45 | Caucasian | M | Blood |
| GSM3035663_3035663 | 45 | Hispanic | F | Blood |
| GSM3035673_3035673 | 45 | Caucasian | F | Blood |
| GSM6057823_6057823 | 45 | Han_Chinese | M | Blood |
| GSM6057873_6057873 | 45 | Han_Chinese | F | Blood |
| GSM6425122_6425122 | 45 | African_American | M | Blood |
| GSM6425135_6425135 | 45 | African_American | F | Blood |
| GSM3035419_3035419 | 46 | Caucasian | F | Blood |
| GSM3035496_3035496 | 46 | Caucasian | F | Blood |
| GSM3035498_3035498 | 46 | Caucasian | F | Blood |
| GSM3035505_3035505 | 46 | Caucasian | F | Blood |
| GSM3035506_3035506 | 46 | Caucasian | F | Blood |
| GSM6424775_6424775 | 46 | African_American | F | Blood |
| GSM6424776_6424776 | 46 | African_American | F | Blood |
| GSM6424805_6424805 | 46 | African_American | F | Blood |
| GSM6424812_6424812 | 46 | African_American | F | Blood |
| GSM6425139_6425139 | 46 | African_American | F | Blood |
| GSM3035421_3035421 | 47 | Caucasian | M | Blood |
| GSM3035501_3035501 | 47 | Caucasian | M | Blood |
| GSM3035507_3035507 | 47 | Caucasian | F | Blood |
| GSM3035539_3035539 | 47 | Caucasian | F | Blood |
| GSM3035587_3035587 | 47 | Caucasian | F | Blood |
| GSM3035674_3035674 | 47 | Caucasian | F | Blood |
| GSM6057822_6057822 | 47 | Han_Chinese | M | Blood |
| GSM6057829_6057829 | 47 | Han_Chinese | F | Blood |
| GSM6424792_6424792 | 47 | African_American | M | Blood |
| GSM6424817_6424817 | 47 | African_American | M | Blood |
| GSM6425113_6425113 | 47 | African_American | F | Blood |
| GSM6425152_6425152 | 47 | African_American | M | Blood |
| GSM3035431_3035431 | 48 | Caucasian | M | Blood |
| GSM3035449_3035449 | 48 | Caucasian | F | Blood |
| GSM3035493_3035493 | 48 | Caucasian | M | Blood |
| GSM3035515_3035515 | 48 | Caucasian | M | Blood |
| GSM3035588_3035588 | 48 | Hispanic | M | Blood |
| GSM3035652_3035652 | 48 | Hispanic | F | Blood |
| GSM6057832_6057832 | 48 | Han_Chinese | M | Blood |
| GSM6057842_6057842 | 48 | Han_Chinese | M | Blood |
| GSM6424774_6424774 | 48 | African_American | F | Blood |
| GSM6424779_6424779 | 48 | African_American | M | Blood |
| GSM6424803_6424803 | 48 | African_American | F | Blood |
| GSM6424826_6424826 | 48 | African_American | M | Blood |
| GSM6425109_6425109 | 48 | African_American | F | Blood |
| GSM6425143_6425143 | 48 | African_American | M | Blood |
| GSM3035468_3035468 | 49 | Caucasian | F | Blood |
| GSM3035641_3035641 | 49 | Caucasian | M | Blood |
| GSM3035651_3035651 | 49 | Caucasian | M | Blood |
| GSM6424780_6424780 | 49 | African_American | F | Blood |
| GSM6424794_6424794 | 49 | African_American | F | Blood |
| GSM6424802_6424802 | 49 | African_American | M | Blood |
| GSM6424822_6424822 | 49 | African_American | F | Blood |
| GSM6424829_6424829 | 49 | African_American | F | Blood |
| GSM6425118_6425118 | 49 | African_American | F | Blood |
| GSM6425127_6425127 | 49 | African_American | F | Blood |
| GSM6425145_6425145 | 49 | African_American | F | Blood |
| GSM3035466_3035466 | 50 | Caucasian | F | Blood |
| GSM3035633_3035633 | 50 | Caucasian | F | Blood |
| GSM3035653_3035653 | 50 | Caucasian | F | Blood |
| GSM3035798_3035798 | 50 | Caucasian | M | Blood |
| GSM3035802_3035802 | 50 | Caucasian | M | Blood |
| GSM6057844_6057844 | 50 | Han_Chinese | M | Blood |
| GSM6424773_6424773 | 50 | African_American | F | Blood |
| GSM6424798_6424798 | 50 | African_American | F | Blood |
| GSM6424810_6424810 | 50 | African_American | F | Blood |
| GSM6425128_6425128 | 50 | African_American | M | Blood |
| GSM6425141_6425141 | 50 | African_American | F | Blood |
| GSM6425148_6425148 | 50 | African_American | F | Blood |
| GSM3035451_3035451 | 51 | Hispanic | F | Blood |
| GSM3035748_3035748 | 51 | Caucasian | F | Blood |
| GSM3035872_3035872 | 51 | Caucasian | F | Blood |
| GSM6057825_6057825 | 51 | Han_Chinese | M | Blood |
| GSM6424763_6424763 | 51 | African_American | F | Blood |
| GSM6424769_6424769 | 51 | African_American | F | Blood |
| GSM6424771_6424771 | 51 | African_American | F | Blood |
| GSM6424787_6424787 | 51 | African_American | F | Blood |
| GSM6424789_6424789 | 51 | African_American | M | Blood |
| GSM6424804_6424804 | 51 | African_American | F | Blood |
| GSM6424823_6424823 | 51 | African_American | M | Blood |
| GSM6424824_6424824 | 51 | African_American | F | Blood |
| GSM6424828_6424828 | 51 | African_American | F | Blood |
| GSM6425115_6425115 | 51 | African_American | F | Blood |
| GSM6425121_6425121 | 51 | African_American | F | Blood |
| GSM6425123_6425123 | 51 | African_American | F | Blood |
| GSM6425133_6425133 | 51 | African_American | F | Blood |
| GSM6425140_6425140 | 51 | African_American | M | Blood |
| GSM3035425_3035425 | 52 | Hispanic | M | Blood |
| GSM3035443_3035443 | 52 | Caucasian | M | Blood |
| GSM3035448_3035448 | 52 | Caucasian | M | Blood |
| GSM3035695_3035695 | 52 | Caucasian | F | Blood |
| GSM3035752_3035752 | 52 | Caucasian | F | Blood |
| GSM6057828_6057828 | 52 | Han_Chinese | F | Blood |
| GSM6057830_6057830 | 52 | Han_Chinese | M | Blood |
| GSM6424761_6424761 | 52 | African_American | M | Blood |
| GSM6424765_6424765 | 52 | African_American | M | Blood |
| GSM6424767_6424767 | 52 | African_American | M | Blood |
| GSM6424791_6424791 | 52 | African_American | F | Blood |
| GSM6424799_6424799 | 52 | African_American | F | Blood |
| GSM6424814_6424814 | 52 | African_American | F | Blood |
| GSM6424830_6424830 | 52 | African_American | M | Blood |
| GSM6425111_6425111 | 52 | African_American | F | Blood |
| GSM6425116_6425116 | 52 | African_American | F | Blood |
| GSM6425131_6425131 | 52 | African_American | F | Blood |
| GSM6425146_6425146 | 52 | African_American | F | Blood |
| GSM6425149_6425149 | 52 | African_American | M | Blood |
| GSM3035503_3035503 | 53 | Caucasian | M | Blood |
| GSM3035512_3035512 | 53 | Caucasian | M | Blood |
| GSM3035535_3035535 | 53 | Hispanic | F | Blood |
| GSM3035551_3035551 | 53 | Caucasian | M | Blood |
| GSM3035574_3035574 | 53 | Caucasian | F | Blood |
| GSM3035686_3035686 | 53 | Caucasian | F | Blood |
| GSM3035688_3035688 | 53 | Caucasian | M | Blood |
| GSM3035716_3035716 | 53 | Caucasian | F | Blood |
| GSM3035738_3035738 | 53 | Caucasian | F | Blood |
| GSM3035833_3035833 | 53 | Caucasian | F | Blood |
| GSM3035938_3035938 | 53 | Caucasian | F | Blood |
| GSM6057826_6057826 | 53 | Han_Chinese | F | Blood |
| GSM6424783_6424783 | 53 | African_American | F | Blood |
| GSM6424796_6424796 | 53 | African_American | F | Blood |
| GSM6424806_6424806 | 53 | African_American | M | Blood |
| GSM6424820_6424820 | 53 | African_American | M | Blood |
| GSM6424827_6424827 | 53 | African_American | M | Blood |
| GSM6424897_6424897 | 53 | African_American | F | Blood |
| GSM6424961_6424961 | 53 | African_American | F | Blood |
| GSM6425035_6425035 | 53 | African_American | F | Blood |
| GSM6425054_6425054 | 53 | African_American | M | Blood |
| GSM6425063_6425063 | 53 | African_American | M | Blood |
| GSM6425081_6425081 | 53 | African_American | F | Blood |
| GSM6425126_6425126 | 53 | African_American | F | Blood |
| GSM6425129_6425129 | 53 | African_American | F | Blood |
| GSM6425151_6425151 | 53 | African_American | F | Blood |
| GSM3035508_3035508 | 54 | Caucasian | M | Blood |
| GSM3035656_3035656 | 54 | Caucasian | M | Blood |
| GSM3035670_3035670 | 54 | Hispanic | F | Blood |
| GSM3035838_3035838 | 54 | Caucasian | M | Blood |
| GSM3035910_3035910 | 54 | Caucasian | F | Blood |
| GSM6057812_6057812 | 54 | Han_Chinese | M | Blood |
| GSM6057843_6057843 | 54 | Han_Chinese | F | Blood |
| GSM6424757_6424757 | 54 | African_American | F | Blood |
| GSM6424927_6424927 | 54 | African_American | F | Blood |
| GSM6424960_6424960 | 54 | African_American | F | Blood |
| GSM6424962_6424962 | 54 | African_American | F | Blood |
| GSM6424969_6424969 | 54 | African_American | M | Blood |
| GSM6425007_6425007 | 54 | African_American | M | Blood |
| GSM6425062_6425062 | 54 | African_American | F | Blood |
| GSM6425078_6425078 | 54 | African_American | F | Blood |
| GSM6425096_6425096 | 54 | African_American | F | Blood |
| GSM6425153_6425153 | 54 | African_American | F | Blood |
| GSM3035407_3035407 | 55 | Caucasian | F | Blood |
| GSM3035534_3035534 | 55 | Caucasian | M | Blood |
| GSM3035589_3035589 | 55 | Caucasian | F | Blood |
| GSM3035749_3035749 | 55 | Caucasian | F | Blood |
| GSM3035935_3035935 | 55 | Caucasian | F | Blood |
| GSM6057841_6057841 | 55 | Han_Chinese | F | Blood |
| GSM6424849_6424849 | 55 | African_American | F | Blood |
| GSM6424871_6424871 | 55 | African_American | F | Blood |
| GSM6424873_6424873 | 55 | African_American | M | Blood |
| GSM6424877_6424877 | 55 | African_American | M | Blood |
| GSM6424884_6424884 | 55 | African_American | F | Blood |
| GSM6424894_6424894 | 55 | African_American | M | Blood |
| GSM6424939_6424939 | 55 | African_American | F | Blood |
| GSM6424984_6424984 | 55 | African_American | M | Blood |
| GSM6424986_6424986 | 55 | African_American | F | Blood |
| GSM6425000_6425000 | 55 | African_American | F | Blood |
| GSM6425023_6425023 | 55 | African_American | M | Blood |
| GSM6425031_6425031 | 55 | African_American | M | Blood |
| GSM6425033_6425033 | 55 | African_American | M | Blood |
| GSM6425046_6425046 | 55 | African_American | F | Blood |
| GSM6425059_6425059 | 55 | African_American | F | Blood |
| GSM3035594_3035594 | 56 | Caucasian | M | Blood |
| GSM3035597_3035597 | 56 | Caucasian | F | Blood |
| GSM3035634_3035634 | 56 | Hispanic | M | Blood |
| GSM3035737_3035737 | 56 | Caucasian | M | Blood |
| GSM3035845_3035845 | 56 | Caucasian | F | Blood |
| GSM3035922_3035922 | 56 | Caucasian | F | Blood |
| GSM6057839_6057839 | 56 | Han_Chinese | F | Blood |
| GSM6057840_6057840 | 56 | Han_Chinese | M | Blood |
| GSM6424744_6424744 | 56 | African_American | F | Blood |
| GSM6424854_6424854 | 56 | African_American | F | Blood |
| GSM6424901_6424901 | 56 | African_American | F | Blood |
| GSM6424911_6424911 | 56 | African_American | F | Blood |
| GSM6424982_6424982 | 56 | African_American | F | Blood |
| GSM6425029_6425029 | 56 | African_American | F | Blood |
| GSM6425042_6425042 | 56 | African_American | F | Blood |
| GSM6425060_6425060 | 56 | African_American | F | Blood |
| GSM6425067_6425067 | 56 | African_American | F | Blood |
| GSM6425074_6425074 | 56 | African_American | F | Blood |
| GSM6425088_6425088 | 56 | African_American | M | Blood |
| GSM6425093_6425093 | 56 | African_American | F | Blood |
| GSM3035520_3035520 | 57 | Hispanic | M | Blood |
| GSM3035730_3035730 | 57 | Caucasian | M | Blood |
| GSM3035739_3035739 | 57 | Caucasian | F | Blood |
| GSM3035784_3035784 | 57 | Caucasian | F | Blood |
| GSM3035954_3035954 | 57 | Caucasian | M | Blood |
| GSM6424835_6424835 | 57 | African_American | F | Blood |
| GSM6424836_6424836 | 57 | African_American | F | Blood |
| GSM6424845_6424845 | 57 | African_American | F | Blood |
| GSM6424847_6424847 | 57 | African_American | F | Blood |
| GSM6424859_6424859 | 57 | African_American | F | Blood |
| GSM6424860_6424860 | 57 | African_American | F | Blood |
| GSM6424890_6424890 | 57 | African_American | F | Blood |
| GSM6424900_6424900 | 57 | African_American | F | Blood |
| GSM6424902_6424902 | 57 | African_American | F | Blood |
| GSM6424910_6424910 | 57 | African_American | F | Blood |
| GSM6424915_6424915 | 57 | African_American | F | Blood |
| GSM6424923_6424923 | 57 | African_American | F | Blood |
| GSM6424941_6424941 | 57 | African_American | F | Blood |
| GSM6424964_6424964 | 57 | African_American | M | Blood |
| GSM6424968_6424968 | 57 | African_American | F | Blood |
| GSM6424972_6424972 | 57 | African_American | F | Blood |
| GSM6424973_6424973 | 57 | African_American | F | Blood |
| GSM6424993_6424993 | 57 | African_American | F | Blood |
| GSM6425003_6425003 | 57 | African_American | F | Blood |
| GSM6425006_6425006 | 57 | African_American | F | Blood |
| GSM6425012_6425012 | 57 | African_American | F | Blood |
| GSM6425014_6425014 | 57 | African_American | F | Blood |
| GSM6425017_6425017 | 57 | African_American | F | Blood |
| GSM6425036_6425036 | 57 | African_American | F | Blood |
| GSM6425053_6425053 | 57 | African_American | F | Blood |
| GSM6425068_6425068 | 57 | African_American | F | Blood |
| GSM6425069_6425069 | 57 | African_American | M | Blood |
| GSM6425071_6425071 | 57 | African_American | F | Blood |
| GSM6425087_6425087 | 57 | African_American | F | Blood |
| GSM6425100_6425100 | 57 | African_American | F | Blood |
| GSM6425103_6425103 | 57 | African_American | F | Blood |
| GSM3035418_3035418 | 58 | Caucasian | F | Blood |
| GSM3035422_3035422 | 58 | Hispanic | F | Blood |
| GSM3035581_3035581 | 58 | Caucasian | M | Blood |
| GSM3035795_3035795 | 58 | Caucasian | M | Blood |
| GSM3035815_3035815 | 58 | Caucasian | F | Blood |
| GSM3035830_3035830 | 58 | Caucasian | F | Blood |
| GSM6057838_6057838 | 58 | Han_Chinese | F | Blood |
| GSM6424736_6424736 | 58 | African_American | M | Blood |
| GSM6424749_6424749 | 58 | African_American | M | Blood |
| GSM6424755_6424755 | 58 | African_American | F | Blood |
| GSM6424759_6424759 | 58 | African_American | F | Blood |
| GSM6424852_6424852 | 58 | African_American | F | Blood |
| GSM6424868_6424868 | 58 | African_American | F | Blood |
| GSM6424869_6424869 | 58 | African_American | M | Blood |
| GSM6424878_6424878 | 58 | African_American | M | Blood |
| GSM6424882_6424882 | 58 | African_American | F | Blood |
| GSM6424905_6424905 | 58 | African_American | F | Blood |
| GSM6424907_6424907 | 58 | African_American | F | Blood |
| GSM6424908_6424908 | 58 | African_American | F | Blood |
| GSM6424944_6424944 | 58 | African_American | F | Blood |
| GSM6424947_6424947 | 58 | African_American | F | Blood |
| GSM6424977_6424977 | 58 | African_American | F | Blood |
| GSM6425015_6425015 | 58 | African_American | F | Blood |
| GSM6425027_6425027 | 58 | African_American | F | Blood |
| GSM6425049_6425049 | 58 | African_American | F | Blood |
| GSM6425058_6425058 | 58 | African_American | M | Blood |
| GSM6425102_6425102 | 58 | African_American | F | Blood |
| GSM3035417_3035417 | 59 | Caucasian | M | Blood |
| GSM3035427_3035427 | 59 | Caucasian | M | Blood |
| GSM3035550_3035550 | 59 | Caucasian | M | Blood |
| GSM3035630_3035630 | 59 | Caucasian | M | Blood |
| GSM3035704_3035704 | 59 | Caucasian | F | Blood |
| GSM3035900_3035900 | 59 | Caucasian | F | Blood |
| GSM6057834_6057834 | 59 | Han_Chinese | F | Blood |
| GSM6057837_6057837 | 59 | Han_Chinese | F | Blood |
| GSM6424738_6424738 | 59 | African_American | F | Blood |
| GSM6424740_6424740 | 59 | African_American | F | Blood |
| GSM6424743_6424743 | 59 | African_American | M | Blood |
| GSM6424831_6424831 | 59 | African_American | M | Blood |
| GSM6424833_6424833 | 59 | African_American | F | Blood |
| GSM6424837_6424837 | 59 | African_American | F | Blood |
| GSM6424865_6424865 | 59 | African_American | F | Blood |
| GSM6424880_6424880 | 59 | African_American | F | Blood |
| GSM6424903_6424903 | 59 | African_American | M | Blood |
| GSM6424909_6424909 | 59 | African_American | F | Blood |
| GSM6424912_6424912 | 59 | African_American | M | Blood |
| GSM6424913_6424913 | 59 | African_American | F | Blood |
| GSM6424926_6424926 | 59 | African_American | F | Blood |
| GSM6424936_6424936 | 59 | African_American | F | Blood |
| GSM6424942_6424942 | 59 | African_American | F | Blood |
| GSM6424945_6424945 | 59 | African_American | F | Blood |
| GSM6424956_6424956 | 59 | African_American | F | Blood |
| GSM6424991_6424991 | 59 | African_American | M | Blood |
| GSM6424999_6424999 | 59 | African_American | F | Blood |
| GSM6425001_6425001 | 59 | African_American | F | Blood |
| GSM6425004_6425004 | 59 | African_American | F | Blood |
| GSM6425016_6425016 | 59 | African_American | M | Blood |
| GSM6425032_6425032 | 59 | African_American | F | Blood |
| GSM6425038_6425038 | 59 | African_American | F | Blood |
| GSM6425045_6425045 | 59 | African_American | F | Blood |
| GSM6425051_6425051 | 59 | African_American | F | Blood |
| GSM6425065_6425065 | 59 | African_American | F | Blood |
| GSM6425066_6425066 | 59 | African_American | M | Blood |
| GSM6425091_6425091 | 59 | African_American | F | Blood |
| GSM3035411_3035411 | 60 | Caucasian | F | Blood |
| GSM3035467_3035467 | 60 | Caucasian | F | Blood |
| GSM3035546_3035546 | 60 | Caucasian | M | Blood |
| GSM3035565_3035565 | 60 | Caucasian | F | Blood |
| GSM3035774_3035774 | 60 | Caucasian | F | Blood |
| GSM3035851_3035851 | 60 | Caucasian | M | Blood |
| GSM3035887_3035887 | 60 | Caucasian | M | Blood |
| GSM3035927_3035927 | 60 | Caucasian | M | Blood |
| GSM3035947_3035947 | 60 | Caucasian | M | Blood |
| GSM6424758_6424758 | 60 | African_American | F | Blood |
| GSM6424846_6424846 | 60 | African_American | M | Blood |
| GSM6424853_6424853 | 60 | African_American | F | Blood |
| GSM6424864_6424864 | 60 | African_American | F | Blood |
| GSM6424888_6424888 | 60 | African_American | F | Blood |
| GSM6424889_6424889 | 60 | African_American | F | Blood |
| GSM6424914_6424914 | 60 | African_American | F | Blood |
| GSM6424918_6424918 | 60 | African_American | M | Blood |
| GSM6424928_6424928 | 60 | African_American | F | Blood |
| GSM6424953_6424953 | 60 | African_American | F | Blood |
| GSM6424963_6424963 | 60 | African_American | F | Blood |
| GSM6424967_6424967 | 60 | African_American | F | Blood |
| GSM6424974_6424974 | 60 | African_American | F | Blood |
| GSM6424988_6424988 | 60 | African_American | M | Blood |
| GSM6424992_6424992 | 60 | African_American | F | Blood |
| GSM6424994_6424994 | 60 | African_American | F | Blood |
| GSM6425005_6425005 | 60 | African_American | M | Blood |
| GSM6425008_6425008 | 60 | African_American | F | Blood |
| GSM6425020_6425020 | 60 | African_American | F | Blood |
| GSM6425022_6425022 | 60 | African_American | F | Blood |
| GSM6425037_6425037 | 60 | African_American | F | Blood |
| GSM6425047_6425047 | 60 | African_American | M | Blood |
| GSM6425048_6425048 | 60 | African_American | F | Blood |
| GSM6425061_6425061 | 60 | African_American | F | Blood |
| GSM6425094_6425094 | 60 | African_American | F | Blood |
| GSM6425101_6425101 | 60 | African_American | F | Blood |
| GSM3035435_3035435 | 61 | Caucasian | F | Blood |
| GSM3035462_3035462 | 61 | Caucasian | F | Blood |
| GSM3035577_3035577 | 61 | Caucasian | M | Blood |
| GSM3035591_3035591 | 61 | Caucasian | M | Blood |
| GSM3035689_3035689 | 61 | Caucasian | M | Blood |
| GSM3035711_3035711 | 61 | Caucasian | M | Blood |
| GSM3035731_3035731 | 61 | Caucasian | M | Blood |
| GSM3035764_3035764 | 61 | Caucasian | F | Blood |
| GSM3035804_3035804 | 61 | Caucasian | M | Blood |
| GSM3035918_3035918 | 61 | Caucasian | F | Blood |
| GSM6424737_6424737 | 61 | African_American | F | Blood |
| GSM6424748_6424748 | 61 | African_American | F | Blood |
| GSM6424750_6424750 | 61 | African_American | F | Blood |
| GSM6424838_6424838 | 61 | African_American | F | Blood |
| GSM6424851_6424851 | 61 | African_American | F | Blood |
| GSM6424858_6424858 | 61 | African_American | M | Blood |
| GSM6424887_6424887 | 61 | African_American | M | Blood |
| GSM6424938_6424938 | 61 | African_American | F | Blood |
| GSM6424949_6424949 | 61 | African_American | M | Blood |
| GSM6424957_6424957 | 61 | African_American | F | Blood |
| GSM6424958_6424958 | 61 | African_American | F | Blood |
| GSM6424976_6424976 | 61 | African_American | M | Blood |
| GSM6424987_6424987 | 61 | African_American | F | Blood |
| GSM6425002_6425002 | 61 | African_American | F | Blood |
| GSM6425009_6425009 | 61 | African_American | M | Blood |
| GSM6425011_6425011 | 61 | African_American | M | Blood |
| GSM6425025_6425025 | 61 | African_American | F | Blood |
| GSM6425040_6425040 | 61 | African_American | F | Blood |
| GSM6425056_6425056 | 61 | African_American | F | Blood |
| GSM3035403_3035403 | 62 | Caucasian | M | Blood |
| GSM3035455_3035455 | 62 | Caucasian | F | Blood |
| GSM3035481_3035481 | 62 | Hispanic | M | Blood |
| GSM3035549_3035549 | 62 | Caucasian | F | Blood |
| GSM3035556_3035556 | 62 | Caucasian | M | Blood |
| GSM3035566_3035566 | 62 | Caucasian | F | Blood |
| GSM3035631_3035631 | 62 | Hispanic | F | Blood |
| GSM3035636_3035636 | 62 | Hispanic | M | Blood |
| GSM3035655_3035655 | 62 | Caucasian | F | Blood |
| GSM3035672_3035672 | 62 | Hispanic | M | Blood |
| GSM3035875_3035875 | 62 | Caucasian | M | Blood |
| GSM3035930_3035930 | 62 | Caucasian | F | Blood |
| GSM3035937_3035937 | 62 | Caucasian | F | Blood |
| GSM3035970_3035970 | 62 | Caucasian | F | Blood |
| GSM6424756_6424756 | 62 | African_American | F | Blood |
| GSM6424841_6424841 | 62 | African_American | F | Blood |
| GSM6424842_6424842 | 62 | African_American | M | Blood |
| GSM6424843_6424843 | 62 | African_American | F | Blood |
| GSM6424866_6424866 | 62 | African_American | M | Blood |
| GSM6424916_6424916 | 62 | African_American | M | Blood |
| GSM6424924_6424924 | 62 | African_American | F | Blood |
| GSM6424935_6424935 | 62 | African_American | F | Blood |
| GSM6424937_6424937 | 62 | African_American | M | Blood |
| GSM6424979_6424979 | 62 | African_American | F | Blood |
| GSM6424995_6424995 | 62 | African_American | F | Blood |
| GSM6425013_6425013 | 62 | African_American | M | Blood |
| GSM6425019_6425019 | 62 | African_American | M | Blood |
| GSM6425024_6425024 | 62 | African_American | F | Blood |
| GSM6425064_6425064 | 62 | African_American | F | Blood |
| GSM6425070_6425070 | 62 | African_American | F | Blood |
| GSM6425083_6425083 | 62 | African_American | F | Blood |
| GSM6425089_6425089 | 62 | African_American | F | Blood |
| GSM6425092_6425092 | 62 | African_American | F | Blood |
| GSM6425097_6425097 | 62 | African_American | M | Blood |
| GSM3035430_3035430 | 63 | Caucasian | F | Blood |
| GSM3035521_3035521 | 63 | Caucasian | F | Blood |
| GSM3035557_3035557 | 63 | Caucasian | M | Blood |
| GSM3035609_3035609 | 63 | Caucasian | F | Blood |
| GSM3035703_3035703 | 63 | Caucasian | M | Blood |
| GSM3035708_3035708 | 63 | Caucasian | M | Blood |
| GSM3035717_3035717 | 63 | Caucasian | F | Blood |
| GSM3035741_3035741 | 63 | Caucasian | M | Blood |
| GSM3035750_3035750 | 63 | Caucasian | M | Blood |
| GSM3035761_3035761 | 63 | Caucasian | F | Blood |
| GSM3035783_3035783 | 63 | Caucasian | M | Blood |
| GSM3035850_3035850 | 63 | Caucasian | M | Blood |
| GSM3035892_3035892 | 63 | Caucasian | F | Blood |
| GSM3035905_3035905 | 63 | Caucasian | M | Blood |
| GSM3035962_3035962 | 63 | Caucasian | F | Blood |
| GSM6424739_6424739 | 63 | African_American | F | Blood |
| GSM6424741_6424741 | 63 | African_American | F | Blood |
| GSM6424752_6424752 | 63 | African_American | F | Blood |
| GSM6424754_6424754 | 63 | African_American | M | Blood |
| GSM6424840_6424840 | 63 | African_American | F | Blood |
| GSM6424855_6424855 | 63 | African_American | M | Blood |
| GSM6424856_6424856 | 63 | African_American | M | Blood |
| GSM6424876_6424876 | 63 | African_American | F | Blood |
| GSM6424883_6424883 | 63 | African_American | F | Blood |
| GSM6424966_6424966 | 63 | African_American | F | Blood |
| GSM6424983_6424983 | 63 | African_American | F | Blood |
| GSM6424989_6424989 | 63 | African_American | M | Blood |
| GSM6424996_6424996 | 63 | African_American | M | Blood |
| GSM6424998_6424998 | 63 | African_American | F | Blood |
| GSM6425010_6425010 | 63 | African_American | M | Blood |
| GSM6425028_6425028 | 63 | African_American | F | Blood |
| GSM6425086_6425086 | 63 | African_American | F | Blood |
| GSM3035538_3035538 | 64 | Caucasian | F | Blood |
| GSM3035552_3035552 | 64 | Caucasian | M | Blood |
| GSM3035555_3035555 | 64 | Caucasian | M | Blood |
| GSM3035625_3035625 | 64 | Caucasian | M | Blood |
| GSM3035685_3035685 | 64 | Caucasian | M | Blood |
| GSM3035702_3035702 | 64 | Caucasian | M | Blood |
| GSM3035725_3035725 | 64 | Caucasian | M | Blood |
| GSM3035729_3035729 | 64 | Caucasian | F | Blood |
| GSM3035740_3035740 | 64 | Caucasian | M | Blood |
| GSM3035758_3035758 | 64 | Caucasian | M | Blood |
| GSM3035788_3035788 | 64 | Caucasian | M | Blood |
| GSM3035893_3035893 | 64 | Caucasian | F | Blood |
| GSM3035968_3035968 | 64 | Hispanic | M | Blood |
| GSM6424742_6424742 | 64 | African_American | M | Blood |
| GSM6424751_6424751 | 64 | African_American | F | Blood |
| GSM6424753_6424753 | 64 | African_American | F | Blood |
| GSM6424839_6424839 | 64 | African_American | M | Blood |
| GSM6424844_6424844 | 64 | African_American | F | Blood |
| GSM6424848_6424848 | 64 | African_American | F | Blood |
| GSM6424867_6424867 | 64 | African_American | F | Blood |
| GSM6424874_6424874 | 64 | African_American | F | Blood |
| GSM6424893_6424893 | 64 | African_American | F | Blood |
| GSM6424895_6424895 | 64 | African_American | F | Blood |
| GSM6424919_6424919 | 64 | African_American | F | Blood |
| GSM6424931_6424931 | 64 | African_American | F | Blood |
| GSM6424943_6424943 | 64 | African_American | F | Blood |
| GSM6424948_6424948 | 64 | African_American | F | Blood |
| GSM6424959_6424959 | 64 | African_American | F | Blood |
| GSM6424978_6424978 | 64 | African_American | F | Blood |
| GSM6424980_6424980 | 64 | African_American | F | Blood |
| GSM6424985_6424985 | 64 | African_American | M | Blood |
| GSM6424990_6424990 | 64 | African_American | M | Blood |
| GSM6425018_6425018 | 64 | African_American | F | Blood |
| GSM6425072_6425072 | 64 | African_American | F | Blood |
| GSM6425090_6425090 | 64 | African_American | F | Blood |
| GSM3035499_3035499 | 65 | Caucasian | M | Blood |
| GSM3035510_3035510 | 65 | Caucasian | M | Blood |
| GSM3035734_3035734 | 65 | Caucasian | M | Blood |
| GSM3035814_3035814 | 65 | Caucasian | M | Blood |
| GSM3035885_3035885 | 65 | Caucasian | F | Blood |
| GSM3035907_3035907 | 65 | Caucasian | F | Blood |
| GSM3035920_3035920 | 65 | Caucasian | M | Blood |
| GSM3035956_3035956 | 65 | Caucasian | F | Blood |
| GSM6424857_6424857 | 65 | African_American | F | Blood |
| GSM6424862_6424862 | 65 | African_American | F | Blood |
| GSM6424863_6424863 | 65 | African_American | M | Blood |
| GSM6424881_6424881 | 65 | African_American | F | Blood |
| GSM6424898_6424898 | 65 | African_American | F | Blood |
| GSM6424906_6424906 | 65 | African_American | F | Blood |
| GSM6424929_6424929 | 65 | African_American | M | Blood |
| GSM6424930_6424930 | 65 | African_American | M | Blood |
| GSM6424950_6424950 | 65 | African_American | F | Blood |
| GSM6425030_6425030 | 65 | African_American | F | Blood |
| GSM6425055_6425055 | 65 | African_American | M | Blood |
| GSM6425080_6425080 | 65 | African_American | M | Blood |
| GSM6425082_6425082 | 65 | African_American | M | Blood |
| GSM6425085_6425085 | 65 | African_American | F | Blood |
| GSM6425095_6425095 | 65 | African_American | F | Blood |
| GSM3035432_3035432 | 66 | Caucasian | M | Blood |
| GSM3035483_3035483 | 66 | Hispanic | F | Blood |
| GSM3035488_3035488 | 66 | Hispanic | M | Blood |
| GSM3035500_3035500 | 66 | Caucasian | M | Blood |
| GSM3035509_3035509 | 66 | Caucasian | F | Blood |
| GSM3035553_3035553 | 66 | Caucasian | M | Blood |
| GSM3035607_3035607 | 66 | Caucasian | M | Blood |
| GSM3035643_3035643 | 66 | Hispanic | M | Blood |
| GSM3035682_3035682 | 66 | Caucasian | M | Blood |
| GSM3035684_3035684 | 66 | Caucasian | M | Blood |
| GSM3035709_3035709 | 66 | Caucasian | F | Blood |
| GSM3035796_3035796 | 66 | Caucasian | F | Blood |
| GSM6424747_6424747 | 66 | African_American | F | Blood |
| GSM6424870_6424870 | 66 | African_American | F | Blood |
| GSM6424872_6424872 | 66 | African_American | F | Blood |
| GSM6424875_6424875 | 66 | African_American | M | Blood |
| GSM6424879_6424879 | 66 | African_American | F | Blood |
| GSM6424886_6424886 | 66 | African_American | F | Blood |
| GSM6424891_6424891 | 66 | African_American | F | Blood |
| GSM6424904_6424904 | 66 | African_American | F | Blood |
| GSM6424921_6424921 | 66 | African_American | F | Blood |
| GSM6424925_6424925 | 66 | African_American | F | Blood |
| GSM6424934_6424934 | 66 | African_American | F | Blood |
| GSM6424971_6424971 | 66 | African_American | M | Blood |
| GSM6424997_6424997 | 66 | African_American | M | Blood |
| GSM6425021_6425021 | 66 | African_American | F | Blood |
| GSM6425026_6425026 | 66 | African_American | F | Blood |
| GSM6425043_6425043 | 66 | African_American | F | Blood |
| GSM6425052_6425052 | 66 | African_American | F | Blood |
| GSM6425073_6425073 | 66 | African_American | M | Blood |
| GSM6425098_6425098 | 66 | African_American | M | Blood |
| GSM3035450_3035450 | 67 | Caucasian | F | Blood |
| GSM3035485_3035485 | 67 | Hispanic | F | Blood |
| GSM3035511_3035511 | 67 | Caucasian | M | Blood |
| GSM3035518_3035518 | 67 | Caucasian | M | Blood |
| GSM3035544_3035544 | 67 | Caucasian | M | Blood |
| GSM3035560_3035560 | 67 | Caucasian | M | Blood |
| GSM3035622_3035622 | 67 | Caucasian | F | Blood |
| GSM3035638_3035638 | 67 | Caucasian | F | Blood |
| GSM3035640_3035640 | 67 | Caucasian | M | Blood |
| GSM3035762_3035762 | 67 | Caucasian | M | Blood |
| GSM3035773_3035773 | 67 | Caucasian | F | Blood |
| GSM3035819_3035819 | 67 | Caucasian | M | Blood |
| GSM3035866_3035866 | 67 | Caucasian | F | Blood |
| GSM6424746_6424746 | 67 | African_American | F | Blood |
| GSM6424834_6424834 | 67 | African_American | F | Blood |
| GSM6424899_6424899 | 67 | African_American | F | Blood |
| GSM6424917_6424917 | 67 | African_American | F | Blood |
| GSM6424920_6424920 | 67 | African_American | F | Blood |
| GSM6424933_6424933 | 67 | African_American | F | Blood |
| GSM6424951_6424951 | 67 | African_American | F | Blood |
| GSM6424952_6424952 | 67 | African_American | M | Blood |
| GSM6424954_6424954 | 67 | African_American | F | Blood |
| GSM6424965_6424965 | 67 | African_American | M | Blood |
| GSM6424975_6424975 | 67 | African_American | F | Blood |
| GSM6425034_6425034 | 67 | African_American | M | Blood |
| GSM6425041_6425041 | 67 | African_American | F | Blood |
| GSM6425075_6425075 | 67 | African_American | F | Blood |
| GSM6425076_6425076 | 67 | African_American | F | Blood |
| GSM6425079_6425079 | 67 | African_American | M | Blood |
| GSM6425105_6425105 | 67 | African_American | F | Blood |
| GSM3035415_3035415 | 68 | Caucasian | M | Blood |
| GSM3035453_3035453 | 68 | Caucasian | M | Blood |
| GSM3035469_3035469 | 68 | Caucasian | F | Blood |
| GSM3035478_3035478 | 68 | Caucasian | M | Blood |
| GSM3035526_3035526 | 68 | Hispanic | F | Blood |
| GSM3035531_3035531 | 68 | Hispanic | M | Blood |
| GSM3035558_3035558 | 68 | Caucasian | M | Blood |
| GSM3035590_3035590 | 68 | Hispanic | M | Blood |
| GSM3035603_3035603 | 68 | Caucasian | F | Blood |
| GSM3035627_3035627 | 68 | Hispanic | M | Blood |
| GSM3035645_3035645 | 68 | Caucasian | M | Blood |
| GSM3035710_3035710 | 68 | Caucasian | M | Blood |
| GSM3035722_3035722 | 68 | Caucasian | M | Blood |
| GSM3035726_3035726 | 68 | Caucasian | F | Blood |
| GSM3035742_3035742 | 68 | Caucasian | M | Blood |
| GSM3035756_3035756 | 68 | Caucasian | M | Blood |
| GSM3035760_3035760 | 68 | Caucasian | M | Blood |
| GSM3035768_3035768 | 68 | Caucasian | M | Blood |
| GSM3035797_3035797 | 68 | Caucasian | F | Blood |
| GSM3035803_3035803 | 68 | Caucasian | M | Blood |
| GSM3035812_3035812 | 68 | Caucasian | F | Blood |
| GSM3035853_3035853 | 68 | Hispanic | M | Blood |
| GSM3035881_3035881 | 68 | Caucasian | M | Blood |
| GSM3035886_3035886 | 68 | Caucasian | F | Blood |
| GSM3035936_3035936 | 68 | Caucasian | F | Blood |
| GSM6424885_6424885 | 68 | African_American | F | Blood |
| GSM6424892_6424892 | 68 | African_American | M | Blood |
| GSM6424896_6424896 | 68 | African_American | F | Blood |
| GSM6424932_6424932 | 68 | African_American | M | Blood |
| GSM6424940_6424940 | 68 | African_American | M | Blood |
| GSM6424955_6424955 | 68 | African_American | F | Blood |
| GSM6424970_6424970 | 68 | African_American | F | Blood |
| GSM6425039_6425039 | 68 | African_American | M | Blood |
| GSM6425044_6425044 | 68 | African_American | F | Blood |
| GSM6425050_6425050 | 68 | African_American | F | Blood |
| GSM6425057_6425057 | 68 | African_American | M | Blood |
| GSM6425077_6425077 | 68 | African_American | M | Blood |
| GSM6425084_6425084 | 68 | African_American | F | Blood |
| GSM3035406_3035406 | 69 | Caucasian | M | Blood |
| GSM3035527_3035527 | 69 | Hispanic | M | Blood |
| GSM3035580_3035580 | 69 | Caucasian | M | Blood |
| GSM3035619_3035619 | 69 | Caucasian | M | Blood |
| GSM3035621_3035621 | 69 | Caucasian | M | Blood |
| GSM3035659_3035659 | 69 | Caucasian | M | Blood |
| GSM3035723_3035723 | 69 | Caucasian | F | Blood |
| GSM3035728_3035728 | 69 | Caucasian | M | Blood |
| GSM3035732_3035732 | 69 | Caucasian | M | Blood |
| GSM3035769_3035769 | 69 | Caucasian | M | Blood |
| GSM3035780_3035780 | 69 | Caucasian | F | Blood |
| GSM3035789_3035789 | 69 | Caucasian | M | Blood |
| GSM3035805_3035805 | 69 | Caucasian | M | Blood |
| GSM3035806_3035806 | 69 | Caucasian | M | Blood |
| GSM3035861_3035861 | 69 | Hispanic | M | Blood |
| GSM3035896_3035896 | 69 | Caucasian | F | Blood |
| GSM3035897_3035897 | 69 | Caucasian | M | Blood |
| GSM6424745_6424745 | 69 | African_American | F | Blood |
| GSM6424762_6424762 | 69 | African_American | M | Blood |
| GSM6424797_6424797 | 69 | African_American | F | Blood |
| GSM6424813_6424813 | 69 | African_American | M | Blood |
| GSM6424832_6424832 | 69 | African_American | F | Blood |
| GSM6424850_6424850 | 69 | African_American | M | Blood |
| GSM6424861_6424861 | 69 | African_American | F | Blood |
| GSM6424922_6424922 | 69 | African_American | F | Blood |
| GSM6424946_6424946 | 69 | African_American | F | Blood |
| GSM6424981_6424981 | 69 | African_American | M | Blood |
| GSM6425104_6425104 | 69 | African_American | M | Blood |
| GSM6425106_6425106 | 69 | African_American | F | Blood |
| GSM3035514_3035514 | 70 | Caucasian | F | Blood |
| GSM3035608_3035608 | 70 | Caucasian | M | Blood |
| GSM3035642_3035642 | 70 | Caucasian | F | Blood |
| GSM3035648_3035648 | 70 | Caucasian | M | Blood |
| GSM3035664_3035664 | 70 | Caucasian | F | Blood |
| GSM3035696_3035696 | 70 | Caucasian | M | Blood |
| GSM3035791_3035791 | 70 | Caucasian | M | Blood |
| GSM3035843_3035843 | 70 | Caucasian | M | Blood |
| GSM3035855_3035855 | 70 | Hispanic | M | Blood |
| GSM3035856_3035856 | 70 | Caucasian | F | Blood |
| GSM3035857_3035857 | 70 | Hispanic | M | Blood |
| GSM3035873_3035873 | 70 | Caucasian | F | Blood |
| GSM3035921_3035921 | 70 | Caucasian | F | Blood |
| GSM3035924_3035924 | 70 | Caucasian | M | Blood |
| GSM3035933_3035933 | 70 | Caucasian | F | Blood |
| GSM6424766_6424766 | 70 | African_American | M | Blood |
| GSM6424770_6424770 | 70 | African_American | F | Blood |
| GSM6424788_6424788 | 70 | African_American | M | Blood |
| GSM6424801_6424801 | 70 | African_American | M | Blood |
| GSM6424815_6424815 | 70 | African_American | F | Blood |
| GSM6425112_6425112 | 70 | African_American | F | Blood |
| GSM6425132_6425132 | 70 | African_American | F | Blood |
| GSM6425134_6425134 | 70 | African_American | F | Blood |
| GSM3035410_3035410 | 71 | Caucasian | F | Blood |
| GSM3035454_3035454 | 71 | Caucasian | M | Blood |
| GSM3035457_3035457 | 71 | Caucasian | M | Blood |
| GSM3035479_3035479 | 71 | Caucasian | M | Blood |
| GSM3035524_3035524 | 71 | Caucasian | M | Blood |
| GSM3035540_3035540 | 71 | Caucasian | M | Blood |
| GSM3035541_3035541 | 71 | Caucasian | M | Blood |
| GSM3035596_3035596 | 71 | Caucasian | M | Blood |
| GSM3035606_3035606 | 71 | Caucasian | F | Blood |
| GSM3035629_3035629 | 71 | Caucasian | F | Blood |
| GSM3035658_3035658 | 71 | Caucasian | M | Blood |
| GSM3035667_3035667 | 71 | Caucasian | F | Blood |
| GSM3035745_3035745 | 71 | Caucasian | F | Blood |
| GSM3035755_3035755 | 71 | Caucasian | F | Blood |
| GSM3035808_3035808 | 71 | Caucasian | M | Blood |
| GSM3035810_3035810 | 71 | Hispanic | M | Blood |
| GSM3035824_3035824 | 71 | Caucasian | M | Blood |
| GSM3035852_3035852 | 71 | Caucasian | M | Blood |
| GSM3035865_3035865 | 71 | Caucasian | M | Blood |
| GSM3035931_3035931 | 71 | Caucasian | M | Blood |
| GSM3035940_3035940 | 71 | Caucasian | M | Blood |
| GSM3035957_3035957 | 71 | Caucasian | F | Blood |
| GSM3035964_3035964 | 71 | Hispanic | F | Blood |
| GSM6424772_6424772 | 71 | African_American | M | Blood |
| GSM6424786_6424786 | 71 | African_American | F | Blood |
| GSM6424790_6424790 | 71 | African_American | F | Blood |
| GSM6424807_6424807 | 71 | African_American | F | Blood |
| GSM6424811_6424811 | 71 | African_American | M | Blood |
| GSM6425117_6425117 | 71 | African_American | F | Blood |
| GSM6425120_6425120 | 71 | African_American | M | Blood |
| GSM6425136_6425136 | 71 | African_American | M | Blood |
| GSM6425137_6425137 | 71 | African_American | M | Blood |
| GSM3035404_3035404 | 72 | Caucasian | M | Blood |
| GSM3035405_3035405 | 72 | Caucasian | F | Blood |
| GSM3035412_3035412 | 72 | Caucasian | M | Blood |
| GSM3035460_3035460 | 72 | Caucasian | M | Blood |
| GSM3035489_3035489 | 72 | Caucasian | F | Blood |
| GSM3035522_3035522 | 72 | Caucasian | M | Blood |
| GSM3035582_3035582 | 72 | Caucasian | M | Blood |
| GSM3035610_3035610 | 72 | Caucasian | M | Blood |
| GSM3035626_3035626 | 72 | Hispanic | F | Blood |
| GSM3035646_3035646 | 72 | Caucasian | M | Blood |
| GSM3035662_3035662 | 72 | Caucasian | F | Blood |
| GSM3035675_3035675 | 72 | Caucasian | F | Blood |
| GSM3035691_3035691 | 72 | Caucasian | F | Blood |
| GSM3035701_3035701 | 72 | Caucasian | F | Blood |
| GSM3035778_3035778 | 72 | Caucasian | M | Blood |
| GSM3035792_3035792 | 72 | Caucasian | M | Blood |
| GSM3035799_3035799 | 72 | Caucasian | F | Blood |
| GSM3035849_3035849 | 72 | Caucasian | M | Blood |
| GSM3035862_3035862 | 72 | Hispanic | M | Blood |
| GSM3035902_3035902 | 72 | Caucasian | M | Blood |
| GSM3035904_3035904 | 72 | Caucasian | F | Blood |
| GSM3035916_3035916 | 72 | Caucasian | M | Blood |
| GSM3035925_3035925 | 72 | Caucasian | M | Blood |
| GSM3035959_3035959 | 72 | Caucasian | F | Blood |
| GSM3035967_3035967 | 72 | Caucasian | M | Blood |
| GSM6424760_6424760 | 72 | African_American | F | Blood |
| GSM6424764_6424764 | 72 | African_American | F | Blood |
| GSM6424784_6424784 | 72 | African_American | F | Blood |
| GSM6424808_6424808 | 72 | African_American | F | Blood |
| GSM6425110_6425110 | 72 | African_American | M | Blood |
| GSM6425130_6425130 | 72 | African_American | M | Blood |
| GSM3035402_3035402 | 73 | Caucasian | F | Blood |
| GSM3035439_3035439 | 73 | Caucasian | M | Blood |
| GSM3035470_3035470 | 73 | Caucasian | M | Blood |
| GSM3035482_3035482 | 73 | Hispanic | M | Blood |
| GSM3035487_3035487 | 73 | Hispanic | F | Blood |
| GSM3035490_3035490 | 73 | Hispanic | M | Blood |
| GSM3035532_3035532 | 73 | Caucasian | F | Blood |
| GSM3035599_3035599 | 73 | Caucasian | M | Blood |
| GSM3035611_3035611 | 73 | Caucasian | M | Blood |
| GSM3035616_3035616 | 73 | Caucasian | M | Blood |
| GSM3035628_3035628 | 73 | Hispanic | M | Blood |
| GSM3035676_3035676 | 73 | Caucasian | F | Blood |
| GSM3035697_3035697 | 73 | Caucasian | M | Blood |
| GSM3035712_3035712 | 73 | Caucasian | F | Blood |
| GSM3035715_3035715 | 73 | Caucasian | F | Blood |
| GSM3035727_3035727 | 73 | Caucasian | F | Blood |
| GSM3035744_3035744 | 73 | Caucasian | F | Blood |
| GSM3035766_3035766 | 73 | Caucasian | F | Blood |
| GSM3035787_3035787 | 73 | Caucasian | F | Blood |
| GSM3035790_3035790 | 73 | Caucasian | M | Blood |
| GSM3035801_3035801 | 73 | Caucasian | M | Blood |
| GSM3035820_3035820 | 73 | Caucasian | M | Blood |
| GSM3035863_3035863 | 73 | Caucasian | M | Blood |
| GSM3035899_3035899 | 73 | Caucasian | M | Blood |
| GSM3035914_3035914 | 73 | Caucasian | F | Blood |
| GSM3035929_3035929 | 73 | Caucasian | M | Blood |
| GSM3035932_3035932 | 73 | Caucasian | M | Blood |
| GSM3035969_3035969 | 73 | Hispanic | F | Blood |
| GSM3035971_3035971 | 73 | Caucasian | F | Blood |
| GSM6424768_6424768 | 73 | African_American | M | Blood |
| GSM6424809_6424809 | 73 | African_American | F | Blood |
| GSM6425114_6425114 | 73 | African_American | F | Blood |
| GSM6425142_6425142 | 73 | African_American | F | Blood |
| GSM3035401_3035401 | 74 | Caucasian | F | Blood |
| GSM3035458_3035458 | 74 | Caucasian | M | Blood |
| GSM3035474_3035474 | 74 | Hispanic | F | Blood |
| GSM3035476_3035476 | 74 | Hispanic | F | Blood |
| GSM3035519_3035519 | 74 | Caucasian | F | Blood |
| GSM3035525_3035525 | 74 | Hispanic | M | Blood |
| GSM3035529_3035529 | 74 | Hispanic | M | Blood |
| GSM3035533_3035533 | 74 | Hispanic | M | Blood |
| GSM3035576_3035576 | 74 | Caucasian | M | Blood |
| GSM3035583_3035583 | 74 | Hispanic | M | Blood |
| GSM3035613_3035613 | 74 | Caucasian | F | Blood |
| GSM3035620_3035620 | 74 | Caucasian | M | Blood |
| GSM3035666_3035666 | 74 | Caucasian | M | Blood |
| GSM3035713_3035713 | 74 | Caucasian | F | Blood |
| GSM3035736_3035736 | 74 | Caucasian | F | Blood |
| GSM3035751_3035751 | 74 | Caucasian | F | Blood |
| GSM3035757_3035757 | 74 | Caucasian | M | Blood |
| GSM3035777_3035777 | 74 | Caucasian | M | Blood |
| GSM3035786_3035786 | 74 | Caucasian | F | Blood |
| GSM3035829_3035829 | 74 | Caucasian | F | Blood |
| GSM3035835_3035835 | 74 | Caucasian | F | Blood |
| GSM3035836_3035836 | 74 | Caucasian | M | Blood |
| GSM3035840_3035840 | 74 | Caucasian | M | Blood |
| GSM3035870_3035870 | 74 | Caucasian | F | Blood |
| GSM3035915_3035915 | 74 | Caucasian | M | Blood |
| GSM3035939_3035939 | 74 | Caucasian | F | Blood |
| GSM3035942_3035942 | 74 | Caucasian | F | Blood |
| GSM3035965_3035965 | 74 | Caucasian | F | Blood |
| GSM6424800_6424800 | 74 | African_American | M | Blood |
| GSM6424818_6424818 | 74 | African_American | M | Blood |
| GSM6425108_6425108 | 74 | African_American | M | Blood |
| GSM3035434_3035434 | 75 | Caucasian | M | Blood |
| GSM3035441_3035441 | 75 | Caucasian | F | Blood |
| GSM3035461_3035461 | 75 | Caucasian | F | Blood |
| GSM3035491_3035491 | 75 | Hispanic | M | Blood |
| GSM3035516_3035516 | 75 | Caucasian | F | Blood |
| GSM3035548_3035548 | 75 | Caucasian | F | Blood |
| GSM3035623_3035623 | 75 | Caucasian | M | Blood |
| GSM3035650_3035650 | 75 | Caucasian | M | Blood |
| GSM3035690_3035690 | 75 | Caucasian | F | Blood |
| GSM3035719_3035719 | 75 | Caucasian | F | Blood |
| GSM3035720_3035720 | 75 | Caucasian | M | Blood |
| GSM3035721_3035721 | 75 | Caucasian | F | Blood |
| GSM3035772_3035772 | 75 | Caucasian | M | Blood |
| GSM3035781_3035781 | 75 | Caucasian | F | Blood |
| GSM3035825_3035825 | 75 | Caucasian | F | Blood |
| GSM3035832_3035832 | 75 | Caucasian | M | Blood |
| GSM3035847_3035847 | 75 | Caucasian | M | Blood |
| GSM3035869_3035869 | 75 | Caucasian | F | Blood |
| GSM3035882_3035882 | 75 | Caucasian | F | Blood |
| GSM3035926_3035926 | 75 | Caucasian | F | Blood |
| GSM6424782_6424782 | 75 | African_American | F | Blood |
| GSM6424795_6424795 | 75 | African_American | F | Blood |
| GSM6424825_6424825 | 75 | African_American | F | Blood |
| GSM6425124_6425124 | 75 | African_American | M | Blood |
| GSM6425138_6425138 | 75 | African_American | M | Blood |
| GSM3035416_3035416 | 76 | Caucasian | M | Blood |
| GSM3035442_3035442 | 76 | Caucasian | M | Blood |
| GSM3035446_3035446 | 76 | Caucasian | M | Blood |
| GSM3035465_3035465 | 76 | Caucasian | F | Blood |
| GSM3035471_3035471 | 76 | Caucasian | F | Blood |
| GSM3035473_3035473 | 76 | Caucasian | F | Blood |
| GSM3035513_3035513 | 76 | Caucasian | M | Blood |
| GSM3035564_3035564 | 76 | Caucasian | M | Blood |
| GSM3035579_3035579 | 76 | Caucasian | M | Blood |
| GSM3035593_3035593 | 76 | Caucasian | F | Blood |
| GSM3035614_3035614 | 76 | Caucasian | M | Blood |
| GSM3035624_3035624 | 76 | Hispanic | F | Blood |
| GSM3035644_3035644 | 76 | Hispanic | F | Blood |
| GSM3035677_3035677 | 76 | Caucasian | M | Blood |
| GSM3035735_3035735 | 76 | Caucasian | M | Blood |
| GSM3035765_3035765 | 76 | Caucasian | M | Blood |
| GSM3035767_3035767 | 76 | Caucasian | M | Blood |
| GSM3035776_3035776 | 76 | Caucasian | F | Blood |
| GSM3035785_3035785 | 76 | Caucasian | M | Blood |
| GSM3035807_3035807 | 76 | Caucasian | M | Blood |
| GSM3035817_3035817 | 76 | Caucasian | M | Blood |
| GSM3035822_3035822 | 76 | Caucasian | F | Blood |
| GSM3035848_3035848 | 76 | Caucasian | F | Blood |
| GSM3035858_3035858 | 76 | Hispanic | F | Blood |
| GSM3035867_3035867 | 76 | Caucasian | M | Blood |
| GSM3035877_3035877 | 76 | Caucasian | F | Blood |
| GSM3035890_3035890 | 76 | Caucasian | M | Blood |
| GSM3035898_3035898 | 76 | Caucasian | F | Blood |
| GSM3035909_3035909 | 76 | Caucasian | F | Blood |
| GSM3035912_3035912 | 76 | Caucasian | M | Blood |
| GSM3035943_3035943 | 76 | Caucasian | F | Blood |
| GSM3035955_3035955 | 76 | Caucasian | F | Blood |
| GSM3035960_3035960 | 76 | Caucasian | M | Blood |
| GSM3035444_3035444 | 77 | Caucasian | F | Blood |
| GSM3035517_3035517 | 77 | Caucasian | F | Blood |
| GSM3035545_3035545 | 77 | Caucasian | M | Blood |
| GSM3035567_3035567 | 77 | Caucasian | F | Blood |
| GSM3035572_3035572 | 77 | Caucasian | M | Blood |
| GSM3035612_3035612 | 77 | Caucasian | M | Blood |
| GSM3035617_3035617 | 77 | Caucasian | M | Blood |
| GSM3035679_3035679 | 77 | Caucasian | M | Blood |
| GSM3035687_3035687 | 77 | Caucasian | M | Blood |
| GSM3035706_3035706 | 77 | Caucasian | M | Blood |
| GSM3035707_3035707 | 77 | Caucasian | F | Blood |
| GSM3035753_3035753 | 77 | Caucasian | F | Blood |
| GSM3035763_3035763 | 77 | Caucasian | F | Blood |
| GSM3035823_3035823 | 77 | Caucasian | M | Blood |
| GSM3035826_3035826 | 77 | Caucasian | M | Blood |
| GSM3035842_3035842 | 77 | Caucasian | F | Blood |
| GSM3035864_3035864 | 77 | Caucasian | F | Blood |
| GSM3035879_3035879 | 77 | Caucasian | F | Blood |
| GSM3035884_3035884 | 77 | Caucasian | M | Blood |
| GSM3035903_3035903 | 77 | Caucasian | M | Blood |
| GSM3035906_3035906 | 77 | Caucasian | M | Blood |
| GSM3035923_3035923 | 77 | Caucasian | M | Blood |
| GSM3035951_3035951 | 77 | Caucasian | M | Blood |
| GSM3035952_3035952 | 77 | Caucasian | M | Blood |
| GSM6425147_6425147 | 77 | African_American | F | Blood |
| GSM3035547_3035547 | 78 | Hispanic | M | Blood |
| GSM3035562_3035562 | 78 | Caucasian | M | Blood |
| GSM3035569_3035569 | 78 | Caucasian | M | Blood |
| GSM3035592_3035592 | 78 | Caucasian | M | Blood |
| GSM3035601_3035601 | 78 | Caucasian | F | Blood |
| GSM3035615_3035615 | 78 | Caucasian | M | Blood |
| GSM3035680_3035680 | 78 | Caucasian | F | Blood |
| GSM3035733_3035733 | 78 | Caucasian | M | Blood |
| GSM3035759_3035759 | 78 | Caucasian | M | Blood |
| GSM3035794_3035794 | 78 | Caucasian | F | Blood |
| GSM3035800_3035800 | 78 | Caucasian | F | Blood |
| GSM3035809_3035809 | 78 | Caucasian | M | Blood |
| GSM3035818_3035818 | 78 | Caucasian | M | Blood |
| GSM3035821_3035821 | 78 | Caucasian | M | Blood |
| GSM3035827_3035827 | 78 | Caucasian | M | Blood |
| GSM3035844_3035844 | 78 | Caucasian | M | Blood |
| GSM3035846_3035846 | 78 | Caucasian | M | Blood |
| GSM3035860_3035860 | 78 | Hispanic | M | Blood |
| GSM3035878_3035878 | 78 | Caucasian | M | Blood |
| GSM3035883_3035883 | 78 | Caucasian | F | Blood |
| GSM3035901_3035901 | 78 | Caucasian | M | Blood |
| GSM3035913_3035913 | 78 | Caucasian | F | Blood |
| GSM3035919_3035919 | 78 | Caucasian | M | Blood |
| GSM3035945_3035945 | 78 | Caucasian | F | Blood |
| GSM3035953_3035953 | 78 | Caucasian | F | Blood |
| GSM3035963_3035963 | 78 | Caucasian | M | Blood |
| GSM3035972_3035972 | 78 | Caucasian | M | Blood |
| GSM6424778_6424778 | 78 | African_American | F | Blood |
| GSM6425099_6425099 | 78 | African_American | M | Blood |
| GSM3035408_3035408 | 79 | Caucasian | M | Blood |
| GSM3035414_3035414 | 79 | Hispanic | M | Blood |
| GSM3035438_3035438 | 79 | Caucasian | M | Blood |
| GSM3035464_3035464 | 79 | Caucasian | F | Blood |
| GSM3035530_3035530 | 79 | Caucasian | M | Blood |
| GSM3035543_3035543 | 79 | Caucasian | F | Blood |
| GSM3035563_3035563 | 79 | Caucasian | F | Blood |
| GSM3035602_3035602 | 79 | Caucasian | F | Blood |
| GSM3035604_3035604 | 79 | Caucasian | F | Blood |
| GSM3035632_3035632 | 79 | Caucasian | F | Blood |
| GSM3035683_3035683 | 79 | Caucasian | F | Blood |
| GSM3035747_3035747 | 79 | Caucasian | M | Blood |
| GSM3035837_3035837 | 79 | Caucasian | M | Blood |
| GSM3035841_3035841 | 79 | Caucasian | F | Blood |
| GSM3035854_3035854 | 79 | Caucasian | M | Blood |
| GSM3035859_3035859 | 79 | Hispanic | F | Blood |
| GSM3035889_3035889 | 79 | Caucasian | M | Blood |
| GSM6424819_6424819 | 79 | African_American | F | Blood |
| GSM3035436_3035436 | 80 | Caucasian | M | Blood |
| GSM3035484_3035484 | 80 | Hispanic | M | Blood |
| GSM3035528_3035528 | 80 | Hispanic | M | Blood |
| GSM3035571_3035571 | 80 | Caucasian | M | Blood |
| GSM3035678_3035678 | 80 | Caucasian | F | Blood |
| GSM3035714_3035714 | 80 | Caucasian | F | Blood |
| GSM3035743_3035743 | 80 | Caucasian | M | Blood |
| GSM3035746_3035746 | 80 | Caucasian | F | Blood |
| GSM3035770_3035770 | 80 | Caucasian | F | Blood |
| GSM3035782_3035782 | 80 | Caucasian | F | Blood |
| GSM3035793_3035793 | 80 | Caucasian | M | Blood |
| GSM3035811_3035811 | 80 | Hispanic | M | Blood |
| GSM3035895_3035895 | 80 | Caucasian | M | Blood |
| GSM3035911_3035911 | 80 | Caucasian | M | Blood |
| GSM3035928_3035928 | 80 | Caucasian | M | Blood |
| GSM3035941_3035941 | 80 | Caucasian | M | Blood |
| GSM3035949_3035949 | 80 | Caucasian | F | Blood |
| GSM6425107_6425107 | 80 | African_American | M | Blood |
| GSM3035409_3035409 | 81 | Caucasian | M | Blood |
| GSM3035437_3035437 | 81 | Caucasian | M | Blood |
| GSM3035452_3035452 | 81 | Caucasian | F | Blood |
| GSM3035463_3035463 | 81 | Caucasian | M | Blood |
| GSM3035542_3035542 | 81 | Caucasian | F | Blood |
| GSM3035561_3035561 | 81 | Caucasian | F | Blood |
| GSM3035578_3035578 | 81 | Caucasian | F | Blood |
| GSM3035618_3035618 | 81 | Caucasian | M | Blood |
| GSM3035681_3035681 | 81 | Caucasian | F | Blood |
| GSM3035699_3035699 | 81 | Caucasian | M | Blood |
| GSM3035771_3035771 | 81 | Caucasian | M | Blood |
| GSM3035831_3035831 | 81 | Caucasian | M | Blood |
| GSM3035908_3035908 | 81 | Caucasian | M | Blood |
| GSM3035917_3035917 | 81 | Caucasian | M | Blood |
| GSM3035948_3035948 | 81 | Caucasian | M | Blood |
| GSM3035413_3035413 | 82 | Caucasian | M | Blood |
| GSM3035445_3035445 | 82 | Caucasian | M | Blood |
| GSM3035480_3035480 | 82 | Caucasian | F | Blood |
| GSM3035495_3035495 | 82 | Caucasian | M | Blood |
| GSM3035693_3035693 | 82 | Caucasian | F | Blood |
| GSM3035700_3035700 | 82 | Caucasian | M | Blood |
| GSM3035724_3035724 | 82 | Caucasian | M | Blood |
| GSM3035754_3035754 | 82 | Caucasian | M | Blood |
| GSM3035934_3035934 | 82 | Caucasian | F | Blood |
| GSM3035946_3035946 | 82 | Caucasian | F | Blood |
| GSM3035958_3035958 | 82 | Caucasian | F | Blood |
| GSM6425119_6425119 | 82 | African_American | M | Blood |
| GSM3035472_3035472 | 83 | Caucasian | M | Blood |
| GSM3035475_3035475 | 83 | Caucasian | M | Blood |
| GSM3035486_3035486 | 83 | Hispanic | M | Blood |
| GSM3035554_3035554 | 83 | Caucasian | M | Blood |
| GSM3035839_3035839 | 83 | Caucasian | M | Blood |
| GSM3035868_3035868 | 83 | Caucasian | F | Blood |
| GSM3035950_3035950 | 83 | Caucasian | F | Blood |
| GSM3035440_3035440 | 84 | Caucasian | F | Blood |
| GSM3035459_3035459 | 84 | Caucasian | F | Blood |
| GSM3035568_3035568 | 84 | Caucasian | M | Blood |
| GSM3035657_3035657 | 84 | Caucasian | M | Blood |
| GSM3035692_3035692 | 84 | Caucasian | F | Blood |
| GSM3035694_3035694 | 84 | Caucasian | F | Blood |
| GSM3035705_3035705 | 84 | Caucasian | M | Blood |
| GSM3035718_3035718 | 84 | Caucasian | M | Blood |
| GSM3035775_3035775 | 84 | Caucasian | F | Blood |
| GSM3035779_3035779 | 84 | Caucasian | M | Blood |
| GSM3035876_3035876 | 84 | Caucasian | F | Blood |
| GSM3035961_3035961 | 84 | Caucasian | M | Blood |
| GSM3035433_3035433 | 85 | Caucasian | M | Blood |
| GSM3035456_3035456 | 85 | Caucasian | F | Blood |
| GSM3035575_3035575 | 85 | Caucasian | M | Blood |
| GSM3035654_3035654 | 85 | Caucasian | F | Blood |
| GSM3035828_3035828 | 85 | Caucasian | M | Blood |
| GSM3035888_3035888 | 85 | Caucasian | M | Blood |
| GSM3035447_3035447 | 86 | Caucasian | F | Blood |
| GSM3035523_3035523 | 86 | Caucasian | F | Blood |
| GSM3035570_3035570 | 86 | Caucasian | M | Blood |
| GSM3035573_3035573 | 86 | Caucasian | M | Blood |
| GSM3035598_3035598 | 86 | Hispanic | M | Blood |
| GSM3035605_3035605 | 86 | Caucasian | M | Blood |
| GSM3035698_3035698 | 86 | Caucasian | M | Blood |
| GSM3035880_3035880 | 86 | Caucasian | M | Blood |
| GSM3035891_3035891 | 86 | Caucasian | F | Blood |
| GSM3035966_3035966 | 86 | Caucasian | M | Blood |
| GSM3035559_3035559 | 87 | Caucasian | F | Blood |
| GSM3035600_3035600 | 87 | Caucasian | M | Blood |
| GSM3035834_3035834 | 87 | Caucasian | F | Blood |
| GSM3035874_3035874 | 87 | Caucasian | M | Blood |
| GSM3035477_3035477 | 88 | Caucasian | M | Blood |
| GSM3035944_3035944 | 88 | Caucasian | F | Blood |
| GSM3035871_3035871 | 89 | Caucasian | M | Blood |
| GSM3035894_3035894 | 89 | Caucasian | M | Blood |
| GSM3035813_3035813 | 90 | Caucasian | M | Blood |
| GSM3035816_3035816 | 90 | Caucasian | F | Blood |
| GSM6425150_6425150 | 91 | African_American | F | Blood |
| GSM3035595_3035595 | 92 | Caucasian | F | Blood |
| GSM1704996_1704996 | 0 * | Mexican | F | Cord_blood |
| GSM1704997_1704997 | 0 * | Mexican | M | Cord_blood |
| GSM1704998_1704998 | 1 * | Mexican | F | Cord_blood |
| GSM1704999_1704999 | 0 * | Mexican | M | Cord_blood |
| GSM1705003_1705003 | 0 * | Mexican | F | Cord_blood |
| GSM1705004_1705004 | 0 * | Mexican | F | Cord_blood |
| GSM1705005_1705005 | 0 * | Mexican | F | Cord_blood |
| GSM1705006_1705006 | 2 * | Mexican | M | Cord_blood |
| GSM1705007_1705007 | 0 * | Mexican | F | Cord_blood |
| GSM1705012_1705012 | 1 * | Mexican | M | Cord_blood |
| GSM1705013_1705013 | 1 * | Mexican | M | Cord_blood |
| GSM1705014_1705014 | 1 * | Mexican | M | Cord_blood |
| GSM1705015_1705015 | 1 * | Mexican | F | Cord_blood |
| GSM1705018_1705018 | 0 * | Mexican | M | Cord_blood |
| GSM1705020_1705020 | 0 * | Mexican | M | Cord_blood |
| GSM1705021_1705021 | 0 * | Mexican | M | Cord_blood |
| GSM1705022_1705022 | 1 * | Mexican | F | Cord_blood |
| GSM1705023_1705023 | 1 * | Mexican | M | Cord_blood |
| GSM1705027_1705027 | 0 * | Mexican | M | Cord_blood |
| GSM1705028_1705028 | 1 * | Mexican | M | Cord_blood |
| GSM1705029_1705029 | 1 * | Mexican | F | Cord_blood |
| GSM1705030_1705030 | 1 * | Mexican | M | Cord_blood |
| GSM1705031_1705031 | 1 * | Mexican | F | Cord_blood |
| GSM1705036_1705036 | 0 * | Mexican | M | Cord_blood |
| GSM1705037_1705037 | 0 * | Mexican | F | Cord_blood |
| GSM1705038_1705038 | 1 * | Mexican | F | Cord_blood |
| GSM1705039_1705039 | 0 * | Mexican | M | Cord_blood |
| GSM1705042_1705042 | 2 * | Mexican | M | Cord_blood |
| GSM1705044_1705044 | 1 * | Mexican | F | Cord_blood |
| GSM1705045_1705045 | 1 * | Mexican | M | Cord_blood |
| GSM1705046_1705046 | 1 * | Mexican | F | Cord_blood |
| GSM1705047_1705047 | 1 * | Mexican | M | Cord_blood |
| GSM1705052_1705052 | 0 * | Mexican | M | Cord_blood |
| GSM1705053_1705053 | 1 * | Mexican | F | Cord_blood |
| GSM1705054_1705054 | 1 * | Mexican | F | Cord_blood |
| GSM1705055_1705055 | 1 * | Mexican | F | Cord_blood |
| GSM1705056_1705056 | 1 * | Mexican | M | Cord_blood |
| GSM1705057_1705057 | 0 * | Mexican | M | Cord_blood |
| GSM1705058_1705058 | 1 * | Mexican | M | Cord_blood |
| GSM1705059_1705059 | 0 * | Mexican | M | Cord_blood |
| GSM1705062_1705062 | 0 * | Mexican | F | Cord_blood |
| GSM1705063_1705063 | 1 * | Mexican | F | Cord_blood |
| GSM1705065_1705065 | 0 * | Mexican | M | Cord_blood |
| GSM1705066_1705066 | 0 * | Mexican | M | Cord_blood |
| GSM1705067_1705067 | 0 * | Mexican | F | Cord_blood |
| GSM1705069_1705069 | 1 * | Mexican | F | Cord_blood |
| GSM1705070_1705070 | 1 * | Mexican | M | Cord_blood |
| GSM1705072_1705072 | 0 * | Mexican | M | Cord_blood |
| GSM1705073_1705073 | 1 * | Mexican | F | Cord_blood |
| GSM1705074_1705074 | 1 * | Mexican | M | Cord_blood |
| GSM1705075_1705075 | 1 * | Mexican | M | Cord_blood |
| GSM1705076_1705076 | 0 * | Mexican | F | Cord_blood |
| GSM1705079_1705079 | 1 * | Mexican | F | Cord_blood |
| GSM1705080_1705080 | 1 * | Mexican | M | Cord_blood |
| GSM1705081_1705081 | 1 * | Mexican | F | Cord_blood |
| GSM1705082_1705082 | 0 * | Mexican | F | Cord_blood |
| GSM1705083_1705083 | 0 * | Mexican | F | Cord_blood |
| GSM1893070_1893070 | 75 * | Chinese | M | Blood |
| GSM1893071_1893071 | 52 * | Chinese | M | Blood |
| GSM1893072_1893072 | 52 * | Chinese | M | Blood |
| GSM1893073_1893073 | 47 * | Chinese | M | Blood |
| GSM1893074_1893074 | 32 * | Chinese | M | Blood |
| GSM1893075_1893075 | 73 * | Chinese | M | Blood |
| GSM1893076_1893076 | 46 * | Chinese | M | Blood |
| GSM1893077_1893077 | 48 * | Chinese | M | Blood |
| GSM1893078_1893078 | 74 * | Chinese | M | Blood |
| GSM1893079_1893079 | 54 * | Chinese | M | Blood |
| GSM1893080_1893080 | 28 * | Chinese | M | Blood |
| GSM1893081_1893081 | 71 * | Chinese | M | Blood |
| GSM1893082_1893082 | 64 * | Chinese | M | Blood |
| GSM1893083_1893083 | 61 * | Chinese | M | Blood |
| GSM1893084_1893084 | 50 * | Chinese | M | Blood |
| GSM1893085_1893085 | 51 * | Chinese | M | Blood |
| GSM1893086_1893086 | 59 * | Chinese | M | Blood |
| GSM1893087_1893087 | 61 * | Chinese | M | Blood |
| GSM1893088_1893088 | 75 * | Chinese | M | Blood |
| GSM1893089_1893089 | 68 * | Chinese | M | Blood |
| GSM1893090_1893090 | 64 * | Chinese | M | Blood |
| GSM1893091_1893091 | 35 * | Chinese | M | Blood |
| GSM1893092_1893092 | 49 * | Chinese | M | Blood |
| GSM1893093_1893093 | 72 * | Chinese | M | Blood |
| GSM1893094_1893094 | 49 * | Chinese | M | Blood |
| GSM1893095_1893095 | 67 * | Chinese | M | Blood |
| GSM1893096_1893096 | 77 * | Chinese | M | Blood |
| GSM1893097_1893097 | 51 * | Chinese | M | Blood |
| GSM1893098_1893098 | 71 * | Chinese | M | Blood |
| GSM1893099_1893099 | 57 * | Chinese | M | Blood |
| GSM1893100_1893100 | 40 * | Chinese | M | Blood |
| GSM1893101_1893101 | 45 * | Chinese | M | Blood |
| GSM1893102_1893102 | 34 * | Chinese | M | Blood |
| GSM1893103_1893103 | 35 * | Chinese | M | Blood |
| GSM1893104_1893104 | 67 * | Chinese | M | Blood |
| GSM1893105_1893105 | 64 * | Chinese | M | Blood |
| GSM1893106_1893106 | 58 * | Chinese | M | Blood |
| GSM1893107_1893107 | 44 * | Chinese | M | Blood |
| GSM1893108_1893108 | 63 * | Chinese | M | Blood |
| GSM1893109_1893109 | 49 * | Chinese | M | Blood |
| GSM1893110_1893110 | 31 * | Chinese | M | Blood |
| GSM1893111_1893111 | 53 * | Chinese | M | Blood |
| GSM1893112_1893112 | 52 * | Chinese | M | Blood |
| GSM1893113_1893113 | 62 * | Chinese | M | Blood |
| GSM1893114_1893114 | 67 * | Chinese | M | Blood |
| GSM1893115_1893115 | 44 * | Chinese | M | Blood |
| GSM1893116_1893116 | 47 * | Chinese | M | Blood |
| GSM1893117_1893117 | 54 * | Chinese | M | Blood |
| GSM1893118_1893118 | 59 * | Chinese | M | Blood |
| GSM1893119_1893119 | 57 * | Chinese | M | Blood |
| GSM1893120_1893120 | 61 * | Chinese | M | Blood |
| GSM1893121_1893121 | 28 * | Chinese | M | Blood |
| GSM1893122_1893122 | 38 * | Chinese | M | Blood |
| GSM1893123_1893123 | 32 * | Chinese | M | Blood |
| GSM1893124_1893124 | 33 * | Chinese | M | Blood |
| GSM1893125_1893125 | 48 * | Chinese | M | Blood |
| GSM1893126_1893126 | 80 * | Chinese | M | Blood |
| GSM1893127_1893127 | 53 * | Chinese | M | Blood |
| GSM1893128_1893128 | 78 * | Chinese | M | Blood |
| GSM1893129_1893129 | 52 * | Chinese | M | Blood |
| GSM1893130_1893130 | 71 * | Chinese | M | Blood |
| GSM1893131_1893131 | 27 * | Chinese | M | Blood |
| GSM1893132_1893132 | 35 * | Chinese | M | Blood |
| GSM1893133_1893133 | 32 * | Chinese | M | Blood |
| GSM1893134_1893134 | 54 * | Chinese | M | Blood |
| GSM1893135_1893135 | 38 * | Chinese | M | Blood |
| GSM1893136_1893136 | 34 * | Chinese | M | Blood |
| GSM1893137_1893137 | 25 * | Chinese | M | Blood |
| GSM1893138_1893138 | 54 * | Chinese | M | Blood |
| GSM1893139_1893139 | 42 * | African | M | Blood |
| GSM1893140_1893140 | 2 * | African | M | Blood |
| GSM1893141_1893141 | 35 * | African | M | Blood |
| GSM1893142_1893142 | 48 * | African | M | Blood |
| GSM1893143_1893143 | 9 * | African | M | Blood |
| GSM5931643_5931643 | 56 * | Hawaiian | M | Monocyte |
| GSM5931644_5931644 | 38 * | Hawaiian | F | Monocyte |
| GSM5931645_5931645 | 62 * | Hawaiian | M | Monocyte |
| GSM5931646_5931646 | 50 * | Hawaiian | F | Monocyte |
| GSM5931647_5931647 | 56 * | Hawaiian | M | Monocyte |
| GSM5931648_5931648 | 44 * | Hawaiian | M | Monocyte |
| GSM5931649_5931649 | 35 * | Hawaiian | F | Monocyte |
| GSM5931650_5931650 | 58 * | Hawaiian | M | Monocyte |
| GSM5931651_5931651 | 55 * | Hawaiian | M | Monocyte |
| GSM5931652_5931652 | 38 * | Hawaiian | F | Monocyte |
| GSM5931653_5931653 | 59 * | Hawaiian | M | Monocyte |
| GSM5931654_5931654 | 53 * | Hawaiian | F | Monocyte |
| GSM5931655_5931655 | 56 * | Hawaiian | M | Monocyte |
| GSM5931656_5931656 | 57 * | Hawaiian | M | Monocyte |
| GSM5931657_5931657 | 55 * | Hawaiian | F | Monocyte |
| GSM5931658_5931658 | 57 * | Hawaiian | M | Monocyte |

* Age Predicted using Horvath Server
